# ﻿Type specimens of recent mammals in Naturalis Biodiversity Center. Part 1 Monotremata, Didelphimorphia, Dasyuromorphia, Peramelemorphia, Diprotodontia, Afrosoricida, Macroscelidea, Hyracoidea, Proboscidea, Scandentia, Primates, Rodentia (Mammalia)

**DOI:** 10.3897/zookeys.1242.117019

**Published:** 2025-06-20

**Authors:** Steven van der Mije, Chris Smeenk, Pepijn Kamminga

**Affiliations:** 1 Naturalis Biodiversity Center, P.O. Box 9517, 2300 RA, Leiden, Netherlands Naturalis Biodiversity Center Leiden Netherlands; † Deceased Naturalis Biodiversity Center Leiden Netherlands

**Keywords:** Naturalis Biodiversity Center, nomenclature, Rijksmuseum van Natuurlijke Historie, Temminck, types, Zoölogisch Museum Amsterdam

## Abstract

This is the first part of a catalogue containing all known types in the mammal collection of Naturalis Biodiversity Center, Leiden, The Netherlands, covering the orders Monotremata to Rodentia in the sequence according to Wilson and Reeder (2005). The remaining orders will be treated in the second part later. The catalogue began in the early 1990s as a basic inventory using historic catalogues. Chris Smeenk, then curator of the mammal collection, researched the types until his death in 2017. The current authors continued his work, resulting in the present publication. We discuss in this first part 427 names. For 341 of these, Naturalis holds name-bearing types. For *Sciuruserythrogenys* Schlegel, 1863 and *Macroxusschlegelii* Gray, 1867 we formally designate the lectotypes.

## ﻿﻿Introduction

This is the first part of a series of publications presenting the type specimens in the mammal collection of Naturalis Biodiversity Center, Leiden, The Netherlands. We intend to publish the catalogue of mammalian types in two parts, starting in this part with the Monotremata to Rodentia and publishing the Lagomorpha to Cetacea in the second part (following Wilson and Reeder 2005). This catalogue covers recent species of Mammalia, meaning extant or extinct since historical times (following Burgin et al. 2020). This agrees with the organisation of collections in Naturalis, where the fossil mammals are part of the paleontological collection.

These catalogues are based on an inventory assembled in the 1990s by a dedicated team of students and temporary staff, and CS continued researching the mammal types until his death in 2014 and left his manuscripts unfinished, especially the chapters on primates and bats that had been extensively researched. PK and SvM continued his work, adding newly discovered taxa and specimens, removing some names or specimens, and completing the parts not elaborated upon by CS. The reader will notice that the parts written by CS are usually more elaborate.

Naturalis Biodiversity Center holds the collections of the former ‘s Rijks Museum van Natuurlijke Historie (**RMNH**) and the Zoölogisch Museum Amsterdam (**ZMA**) after the merger of these two institutions in 2010. The types of the ZMA collection have been published previously by Bergmans (2011), and we also include them in the current publications. Thus we are presenting an overview of the complete Naturalis type collection, which is especially rewarding in the cases where the original type series have been split up between the institutions and are now reunited. Furthermore, we have found a few more taxa and type specimens from the former ZMA collection not included in Bergmans (2011).

## ﻿﻿History of the mammal collection

The collections of Naturalis Biodiversity Center have undergone several name changes in the past (see overview below), but for reasons of consistency we will use Naturalis throughout this publication, also in historical situations when the institutions had different names (Table 1).

**Table 1.** Previous names of Naturalis Biodiversity Center.

1820	‘s Rijks Museum van Natuurlijke Historie (RMNH)	Leiden	foundation
1838	Genootschap ‘Natura Artis Magistra’ (NAM)	Amsterdam	foundation
1892	Zoölogisch Museum Amsterdam (ZMA)	Amsterdam	merger of the collections of NAM and the City of Amsterdam
1989	Nationaal Natuurhistorisch Museum Naturalis (NNM)	Leiden	new name for merger RMNH and RGM (National Geological Museum)
2010	Nederlands Centrum voor Biodiversiteit Naturalis (NCB)	Leiden	new name for NNM, foundation of renewed research institute
2013	Naturalis Biodiversity Center	Leiden	merger of NCB, ZMA and National Dutch Herbaria

Naturalis Biodiversity Center has a long history through its oldest constituent, ‘s Rijks Museum van Natuurlijke Historie. This institution was founded in 1820 as the Dutch national museum for natural history, following the example of the other ‘great nations’. It was based on the collections of Coenraad Jacob Temminck (1778–1858), ‘s Lands Kabinet, the zoological collection of the Leiden University, and what remained of the Kabinet des Stadhouders. Unfortunately, we have no clear picture of the specimens that formed the beginning of the Rijks Museum.

Temminck was the great promoter of a national museum for natural history and became its first director based on his international scientific stature. The fact that he donated his world-famous collection to the state certainly played an important role. The actual move of this collection from his home in Amsterdam to Leiden lasted until 1830 and in the meantime Temminck continued exchanging specimens from this collection. There are however two catalogues of the Temminck collection, the published catalogue from 1807 (Temminck 1807) and an unpublished catalogue in Temminck’s hand dating from approximately 1803 housed in the archives of Naturalis (inv. 49.47). The catalogue of 1807 describes his world famous collection, the 1803 catalogue is clearly its forerunner. Comparing the two catalogues provides an insight in the way Temminck collected (see Jansen 2017), but both catalogues are a list of the species present in the collection, not specific specimens. If the changes between 1803 and 1807 are indicative, we must assume that until 1820 the composition of this collection continued to be very dynamic.

The 1807 catalogue lists 1072 entries of which the majority are birds. But Temminck’s collection also contained mammals. In fact, the first chapters of his catalogue cover “*Simia*” and “*Lemur*”, of which he listed 38 species. In the exchange list we can find monkeys marsupials, squirrels, and a *Viverra*. This might indicate that the catalogue is foremost intended to describe his collection of birds, as the mammals are not extensively treated.

The other founding collection of the ‘s Rijks Museum was the zoological cabinet of the Leiden University. Started as an anatomical collection, under the care of Jean Nicolas Sébastien Allamand (1713–1787) and later Sebald Justinus Brugmans (1763–1819), it also became a noteworthy natural history collection. The most important collection in the Netherlands, the Kabinet des Stadhouders, was confiscated in 1795 by the French occupying forces and shipped to Paris. Brugmans was sent to Paris after the defeat of Napoleon to retrieve this collection. Lamarck resisted and together with Cuvier and von Humboldt convinced Brugmans to accept 10,000 specimens from the so-called *doubles* (exchange) collection of the Paris Museum. Temminck, although critical at first, later admitted that this was the best deal possible. He could not say otherwise, as he was witness to the deal as a cavalry officer in the occupation of Paris (Annual Report in Naturalis archives, inv. 40.6). In 1815 the acquired specimens were shipped to the Netherlands and were donated by king Willem I to the Leiden University (see also Pieters 1980).

From its beginning the Leiden Museum played a major role in the research of the natural riches of the former Dutch colonies, especially in the Dutch East Indies (Indonesia). This effort was boosted by the formation of the Natuurkundige Commissie voor Nederlandsch-Indië on the instigation of Temminck and operating from 1820 until 1850. Several young scientists were sent to the east, and only a few returned, but in the meantime large collections were gathered from all over the colonies and shipped to Leiden. The processing of this material, containing many new species, fell to Temminck and a few of his colleagues and associates. Until now we have found 186 names in total for mammals published by Temminck. Müller (who was a prominent member of the Natuurkundige Commissie) and Schlegel, who succeeded Temminck as director, together described 77 new mammal taxa in total.

Another major contribution to the collection was made by Philipp Franz von Siebold (1796–1866) who worked in Japan from August 1823 to December 1829 in the service of the Vereenigde Oostindische Compagnie (VOC, Dutch East India Company). He was based in the Dutch trading post on the artificial island of Deshima in Nagasaki harbour (Kyūshū). Heinrich Bürger (1804 or 1806–1858) arrived in Japan in 1825 as von Siebold’s assistant. He continued collecting when von Siebold departed from Japan in December in 1829. Bürger left for Java in 1832, returned to Deshima in 1834, and finally left Japan in 1835 (Holthuis and Sakai 1970: 25–39). Apart from the journey to Edo in 1826, during which von Siebold obtained specimens from various places, von Siebold and Bürger were restricted to Deshima and Nagasaki. Thus, most of the material they acquired would have come from that area, although von Siebold in particular received many specimens from other parts of Japan, and even from Korea. There are hardly any specifications of von Siebold’s shipments of mammals, but Bürger’s inventories, preserved in the archives of Naturalis (inv. 232.1440), are very specific (see also von Siebold 1897; Holthuis and Sakai 1970).

Efforts were made by Temminck to promote the study of the natural world in the coastal areas of modern day Ghana and South Africa, where the Dutch conveniently held trading posts. In these regions Temminck relied on interested individuals, an apothecary named Hubertus Benedictus van Horstok (1794–1838) in South Africa and a government official named Hendrik Severinus Pel (1818–1876) in Ghana. Pel’s activities in the Gold Coast (coastal Ghana) are extensively described by Holthuis (1968), who also specifies his collecting localities. Most inventories of Pel’s shipments are preserved (Naturalis archives, inv. 147.114). Temminck’s (1853) account of the mammals of the Guinea coast is based on the collections that Pel brought together.

Beginning in 1875, Fredericus Anna Jentink (1834–1913) was the first dedicated mammalogist employed by the Leiden Museum where, until then, the study of mammals was conducted by Temminck and Schlegel who were primarily ornithologists. Jentink modernised the vertebrate collections, he introduced the practise of preparing specimens as study skins and the old fashioned idea of the collection as a cabinet of curiosities was abandoned in favour of the collection as a basis for scientific research. In his time as a curator and later director until his death in 1913 he described several new mammal species; we found 91 names introduced by Jentink. Jentink was also one of the founders of the ICZN in 1895 and remained an active member of this committee until his death.

After the death of Jentink his position as director, and with it the care of the mammal collection, was taken over by the ornithologists Eduart Daniël Van Oort and later George Christoffel Alexander Junge. It took until 1950 for the museum to hire another dedicated mammalogist, A.M. Husson. Pater (father) Husson, as he was known because of his priesthood, was an expert on Chiroptera in general and the mammals of Suriname and the Dutch Antilles. He retired in 1978.

With the death of Chris Smeenk on 23 March 2017 Naturalis lost its last scientific curator for the mammal collection. Smeenk began his work in Naturalis in 1976, when he succeeded A.M. Husson, after a PhD based on the ecology of birds of prey in Tsavo National Park, Kenya (1970–1973) and managing Pandam Wildlife Park in Nigeria (1974–1976). Originally an ecologist, Smeenk introduced nature conservation as a topic in the museum. He worked on the protection of whales and bats, not only in the Netherlands, but also abroad (see for an account of his life and work Broekhuizen et al. 2017).

## ﻿﻿Zoological Museum of Amsterdam (ZMA)

The origin of the Zoological Museum Amsterdam (ZMA) can be mainly attributed to the work of Max Weber (1852–1937), the first zoological professor at the Amsterdam University. He renovated and combined the collections of the Amsterdam city council and Natura Artis Magistra (Amsterdam Zoo), into a modern scientific zoological collection. Due to his own expeditions to Svalbard, Indonesia, and South Africa together with his wife A. Weber-Van Bosse (a renowned phycologist), he enriched the collection with new species for science, among which were several mammals. In 2011 the ZMA was incorporated into Naturalis Biodiversity Center. See Reitsma (2012) for a full account of the history of the ZMA.

Two mammalogists have greatly contributed to physical and scientific enrichment of the mammal collection of the ZMA. Peter J. H. van Bree (1927–2011) became curator of the mammal collection of the ZMA in 1960, a post he held until his retirement in 1992. He was the first mammal curator who worked on recent mammals, as his predecessor Pieter Jacobus van der Feen mainly worked on fossil mammals. Van Bree’s scientific work mainly focused on sea mammals and he described *Delphinuscapensistropicalis* Van Bree, 1971.

After the retirement of Van Bree, the position of mammal curator remained unoccupied. Wim Bergmans (1940–2018) however took over the care of the mammal collection. Working on Chiroptera, he enlarged this part of the collection with his many field trips to Africa and Asia. He described several new species and with his revisions contributed to our knowledge of this group.

## ﻿﻿Methods and instructions for the reader

The move and reorganisation of the combined mammal collections of ZMA and RMNH have enabled us to examine the specimens and discover new types. We have reviewed all type specimens (primary and secondary) for their presence, completeness, the associated information, and they are stored separately from the main collection. We have digitised the specimens and the dataset, with images of most specimens, is available on GBIF and on the collection portal of Naturalis (http://bioportal.naturalis.nl).

We follow the sequence of families of Wilson and Reeder (2005). Each entry starts with the current species name in bold according to the Illustrated Checklist of the Mammals of the World (Burgin et al. eds 2020), in alphabetic order within the families. This is followed by the names for which we hold types in the original spelling with the complete reference to the description. Subsequently, we cite the type locality as given by the author. Primary types are given with full data, secondary types are generally listed only with their registration number, except in those cases where no primary type is present in the Naturalis collection. We have included names by former staff members even if Naturalis does not hold the types or names of which the types are no longer present in Naturalis.

The label information is given with the listed specimen, and interpretations and additions by us are given between square brackets. General structure of the listed specimen information is: type status, registration number, Jentink’s catalogue entries, sex and stage, preservation type, collecting location, collector and date of collection, donator, and date of accession. Not all of this information is present in all cases, missing information is not indicated. More detailed information on collectors and surveys or expeditions is added where relevant or available. A great help in the recovery of this information was the extensive work of Van Steenis-Kruseman (1950 and online database: https://www.nationaalherbarium.nl/FMCollectors), who brought together information on the itineraries of many collectors in Indonesia and surrounding areas.

There have been several attempts to catalogue the Naturalis mammal collections. A first attempt was made by Schlegel (1876), who catalogued the primates. Schlegel used a numbering system per species and parts of a specimen were given separate numbers, cross referencing them in the specimen description. Jentink catalogued the Manidae (Jentink 1882) and Sciuridae (Jentink 1883a) using a similar method of numbering. Cataloguing the whole mammal collection commenced in 1887 with the publication of the first of three catalogues by Jentink in the ‘Revue méthodique et critique’, containing two so-called systematic catalogues (1888; 1892) covering the skins and one osteological catalogue (1887). In these catalogues the specimens are arranged per species and alphabetically numbered. Cross referencing to the several parts of a specimen listed in the other catalogues (Schlegel 1876; Jentink 1882, 1883a) is given. However, due to the differences in publication dates of these catalogues and the long time between accession of the specimens and the publication of the catalogues, in some cases the given relation between the different parts is erroneous or questionable.

In 1904 Jentink introduced a numeric system to register new accessions in accession books and to retrospectively register the existing collection. This formed the basis of our current numbering system and many of the numbers from the accession registers are still in use. With the introduction of a central registration database in 2012, these numbers are made unique for the whole Naturalis collection by adding two prefixes separated by a dot, RMNH or ZMA denoting the original collection and MAM indicating the mammal collection. To indicate the several parts of a specimen a suffix in the form of a letter is added separated by a dot, for example “RMNH.MAM.17301.a”. However we do not list the individual numbers for the different parts in this catalogue, since all parts belong to the type specimen.

We have not actively pursued specimens from type series in other collections; however, we occasionally refer to specimens when encountered in literature. Being a mere catalogue of the Naturalis collection and not a taxonomic review we hope the mention of these specimens helps the reader. We apologise in advance for the omission of many more to the curators who take care of them. We do encourage these curators to also publish the types in their collections for the benefit of taxonomists around the world.

## ﻿﻿Taxonomic issues

During the research of the type collection we have identified several taxonomic issues. However, as this work is meant to be a catalogue and not a revision, we have indicated the issue at hand, gave our opinion, but merely as a suggestion or recommendation. See the account of the name for a more detailed description (Table 2).

**Table 2.** Identified taxonomic issues.

Taxon	Issue
*Didelphisazarae* Temminck, 1824	Has priority over *Didelphisaurita* Wied, 1826.
*Hyraxsylvestris* Temminck, 1853	Has priority over *Hyraxdorsalis* Fraser, 1854 [=*Dendrohyraxdorsalis* (Fraser, 1854)].
*Onychogaleafraenata* (Gould, 1841)	The correct publication date is 1840, and name published as *Macropusfrenatus*.
*Osphranterrobustus* (Gould, 1841)	The correct publication date is 1840.
*Tarsiussangirensis* Meyer, 1896	The correct publication date is October 1896, not 1897.
*Callithrixdonacophilus* D’Orbigny, 1835	The correct publication date is October 1835 not 1836
*Simiachuva* Humboldt, 1812	The first publication was not *Ateleschuva*Schlegel 1876: 175
*Inuusfuscatus* Blyth, 1875	The first publication was not Gray, 1871
*Macacusbrachyurus* Kuhl, 1820	Has priority over *Macacusmaurus* Schinz, 1825 [=*Macacamaura* (Schinz, 1825)].
*Petauristaelegans* (Temminck, 1836)	The first publication was not Müller, 1840
*Petinomyshageni* (Jentink, 1889)	The correct publication date is 1889, not 1888.
*Sciurusrafflesiiborneoensis* Müller & Schlegel, 1844	The correct publication date is1844, not 1842.
*Sciurusrafflesiiindica* Müller & Schlegel, 1844	The correct publication date is 1844, not 1842.
*Nannosciurusmelanotis* (Mülle, 1841)	The correct publication date is 1841, not 1840, and the type series comprises several subspecies.
*Sciurusmodestus* Müller, 1840	The type series represents two taxa.
*Tachyoryctessplendens* (Rüppell)	The correct publication date is 1836, not 1835.
*Melomysleucogaster* (Jentink, 1909)	The correct publication date is 1909, not 1908.
*Paramelomyslorentzii* (Jentink, 1909)	The correct publication date is 1909, not in 1908.
*Apodemusargenteus* (Temminck, 1844)	The earlier lectotype designation was based on different species.
*Musratticolor* Jentink, 1909	The correct publication date is 1909, not 1908.
*Hystrixbrandtii* Jentink, 1879	The type locality is restricted to Suriname.
*Chinchillabrevicaudata* Waterhouse, 1848	First published as *Chinchillabrevicauda.*

## ﻿﻿Acknowledgements

Compiling this catalogue has been a collective effort. The authors are grateful to all staff members of Naturalis and researchers from every corner of the world that have worked in the mammal collection in the last 200 years, cataloguing and labelling specimens and describing new species. We especially would like to thank Lars van den Hoek-Ostende (Naturalis), who compiled the first list of mammal types in Naturalis; Jan van Tol (Naturalis) for nomenclatural advice; Mrs. N. Smeenk for support in the continuation of her late husband’s work; Karien Lahaise and Lieneke Nijkamp (Naturalis) for assistance with all archival questions; the Naturalis project team comprising Liza Lankhaar, Frank Loggen, Roos van der Helm, Sjoerd Groos, Dagmar Appelman, and Rob Westerduin who helped digitise and photograph the specimens. Furthermore, we would like to thank Guido Keijl (Naturalis), Robert Voss (AMNH), and Jelle Zijlstra for proofreading the manuscript. And last but not least we would like to thank the reviewers of the manuscript, Simon Engelberger (University of Vienna) and Kris Helgen (Australian Museum Research Institute) for their insights and valuable suggestions.

## ﻿﻿Abbreviations

**AMNH** American Museum of Natural History, New York, USA

**ICZN** International Commission on Zoological Nomenclature

**KBIN** Koninklijk Belgisch Instituut voor Natuurwetenschappen, Brussels, Belgium

**KNAG** Koninklijk Nederlands Aardrijkskundig Genootschap

**MfN**Museum für Naturkunde, Berlin (with ZMB as registration number acronym), Germany

**MNHN** Muséum national d’Histoire naturelle, Paris, France

**MTKD** Museum für Tierkunde, Dresden, Germany

**MZB** Museum Zoologicum Bogoriense, Bogor, Indonesia

**NAM** Genootschap Natura Artis Magistra (Amsterdam Zoo), Amsterdam, The Netherlands

**NHM/NHMUK** Natural History Museum, London, UK

**NMNH** Smithsonian, National Museum of Natural History, Washington, USA

**NMW**Naturhistorisches Museum Wien, Vienna, Austria.

**NRM**Naturhistoriska Riksmuseet, Stockholm, Sweden.

**RMCA**Royal Museum for Central Africa, Tervuren, Belgium.

**RMNH** Naturalis Biodiversity Center, Leiden, The Netherlands.

**SMF**Senckenberg Museum Frankfurt, Frankfurt, Germany.

**ZMA** Zoölogisch Museum Amsterdam, The Netherlands (this collection is part of Naturalis since 2010)

**ZSM** Zoologische Staatssammlung München, Munich, Germany

## ﻿﻿List of type specimens

### ﻿﻿MONOTREMATA Bonaparte, 1837

#### ﻿﻿Tachyglossidae Gill, 1872

***Zaglossusattenboroughi* Flannery & Groves, 1998**

*Zaglossusattenboroughi* Flannery & Groves, 1998: 367.

Holotype, RMNH.MAM.17301, sex unknown, skin and fragments of skull. Loc.: eastern top of Mt. Rara at 1600 m, Cyclops Mts., New Guinea, Indonesia. Leg.: P. van Royen, 4 July 1961. Received 6 March 1962.

***Zaglossusbartonismeenki* Flannery & Groves, 1998**

*Zaglossusbartonismeenki* Flannery & Groves, 1998: 367.

Paratype, RMNH.MAM.23319, adult male, skull. Loc.: Mai’iu II, Mount Suckling, Papua New Guinea. Leg.: J.F. Veldkamp, Lae-Leiden Mt. Suckling expedition, 15 July 1972.

This taxon was named after the late Dr Chris Smeenk, conservator of the mammal collection in Naturalis. Flannery and Groves (1998) nominated a specimen in the AMNH (M96822 error for M.9682, see Parnaby et al. (2017: 300)) as the holotype.

### ﻿﻿DIDELPHIMORPHIA Gill, 1872

#### ﻿﻿Didelphidae Gray, 1821

***Didelphisaurita* Wied, 1826**

*Didelphisazaræ* Temminck, 1824b: 30.

Syntype, RMNH.MAM.24239 (Jentink 1888: 219 *a*), male, mounted skin, skull in situ. Loc.: Brazil. Leg.: 1823.

Temminck’s description is based on several specimens (1824b: 32), and only one of those is still present in Naturalis.

Hershkovitz (1969: 54) pointed out that *D.azarae*, “currently used for white-eared opossums [= *Didelphisalbiventris* Lund, 1840] is based on several black-eared opossums”. This is in agreement with Temminck’s description (p. 31). Our syntype too, has a mainly dark ear (one ear is missing).

Gardner (1993: 16; 2005: 5) synonymised *Didelphisaurita* Wied, 1826 with *Didelphisazarae* Temminck, 1824. Although he recognised that Temminck’s name takes priority, he opted for the name *D.aurita*, since *D.azarae* “had been misapplied to *D.albiventris* for over 160 years”. This is followed by Cerqueira and Tribe (2007: 18–21) and Voss (2022: 35) who concluded that in the interest of stability, usage of *Didelphisaurita* Wied, 1826 should be maintained.

***Marmosaparaguayana* Tate, 1931**

*Didelphiscinerea* Temminck, 1824b: 46.

Paralectotype, RMNH.MAM.17924 (Jentink 1888: 221 *a*), female, mounted skin, skull in situ. Loc.: Brazil.

The state and provenance of this specimen are unclear. Temminck’s description (1824b: 46) is based on animals sent to him by Wied and by the NMW. There is no record whether all this material was returned, or whether Temminck may have retained one specimen for Naturalis, as a gift or in exchange.

Wied (1826: 406–411) described one male from his own collection, stating (p. 411) that he himself had not seen any female from this species, for which he referred to Temminck’s description regarding the absence of a pouch. Wied confirmed (p. 409) that he had put his animal at Temminck’s disposal under the (manuscript) name *D.cinerea*, but added (p. 411) that Temminck had studied several specimens since, obviously including one or more females. The inscription on the pedestal of the Naturalis specimen, in Temminck’s handwriting, does not record its provenance. However, Jentink (1888: 221) stated somewhat enigmatically: “un des types du Musée de Vienne. Brésil. Des collections du Prince von Wied”, thus misinterpreting Temminck’s (1824b) specification of provenances, as those two sources are mutually exclusive. If Jentink is correct in assuming that it is one of the types, then the animal, being a female, cannot have come from the Prince of Wied, so would have been received from Vienna and, given Temminck’s remark, would have been collected by Natterer. However, according to Engelberger (in litt., 21 July 2010) the card-file of Natterer’s collection in Vienna gives only one specimen of *D.cinerea*: the one listed in von Pelzeln (1883: 114), which was not collected by Natterer, but by Friedrich Sellow and acquired through the collection of Johann Kammerlacher; there is no record of an animal sent to Temminck, neither in the NMW, nor in the Naturalis archives. Moreover, von Pelzeln (1883) did not refer to Temminck’s description, attributing the name *D.cinerea* to Wied. Pending further evidence, however, we follow Jentink (1888).

Natterer travelled in Brazil between 1817 and 1835 (von Pelzeln 1871, 1883; Vanzolini 1993). If indeed collected by Natterer, RMNH.MAM.17924 must have come into Temminck’s possession not later than 1824, so must have been obtained during one of Natterer’s first four journeys in southeastern Brazil, undertaken between October 1817 and September 1822, in the area between Rio de Janeiro and Paranaguá (Vanzolini 1993: 19–27).

Allen (1900: 190), in discussing the type material of this species, wrote that “the mounted specimen sent to Temminck by Wied…for description”, which is now in the New York Museum (AMNH 845 *Marmosaparaguayana* or *Marmosademerarae* Thomas, 1905.

The name *Didelphiscinerea* Temminck, 1824 is preoccupied by *Didelphiscinerea* Goldfuss, 1809.

***Metachirusmyosuros* (Temminck, 1824)**

*Didelphismyosuros* Temminck, 1824b: 38.

Paralectotype, RMNH.MAM.26072 (Jentink 1888: 220 *a*), female, mounted skin and incomplete skull. Loc.: Brazil.

Paralectotype: RMNH.MAM.26073.

Temminck (1824b: 40) distinguished this new species from *Didelphisnudicaudatus* É. Geoffroy Saint-Hilaire. His description is based on material in various collections, with several specimens of both sexes in Naturalis. The two specimens in Naturalis catalogued as types by Jentink (1888: 220) are both from Brazil, but Temminck had also seen animals from Suriname.

The status and specific identity of the Leiden animals are discussed by Hershkovitz (1959: 343; 1976: 301–302; and, more extensively, 1997: 45–49). He misinterpreted some of Temminck’s statements, doubted the identity and type status of the Leiden and some other specimens, and finally (1997: 49) arranged *D.myosuros* with *Didelphisopossumquica* Temminck, 1824. Husson (1978: 29) and more particularly Gardner and Dagosto (2007: 38) have corrected his erroneous conclusions. Hershkovitz also overlooked that Pohle (1927: 242) had already designated a lectotype for *Metachirusnudicaudatusmyosurus* [= *myosuros*] (Temminck, 1824): a female in the Vienna Museum (NMW B2589), collected by J. Natterer at Ipanema (Bacaetava), São Paulo, Brazil.

***Philanderopossum* (Linnaeus, 1758)**

*Didelphis Opossum* Linnaeus, 1758: 55.

Lectotype, RMNH.MAM.25421.a (Jentink 1888: 220 *j*), adult female, alcohol, skull in situ. Loc.: Suriname. Ex: T.G. van Lidth de Jeude, 1866.

Paralectotypes: RMNH.MAM.25421.b–25421.d.

The description of *Didelphis Opossum* Linnaeus, 1758 is exclusively based on Seba (1734: 56–57, pl. XXXVI figs 1, 2), who depicted a male and a female with three pouch young. The male was reported to be from Brazil, the female with young from Suriname. Hershkovitz (1976: 297) designated the adult female figured in fig. 2 of Seba’s plate the lectotype of the species, but apparently did not realise at the time that this specimen still existed. They were also overlooked by Jentink (1888: 220) and Husson (1978: 24). Hershkovitz (1997: 38) was the first to recognise these specimens as the Seba specimens. One of the young, hidden in the pouch, was overlooked by Jentink (1888).

The history of Seba’s collection, part of which was eventually acquired by the Utrecht zoologist T.G. van Lidth de Jeude, is discussed by Thomas (1892) and Boeseman (1970). After Van Lidt de Jeude’s death, a selection from his collection was purchased by Naturalis in January 1866, before the remaining part was auctioned in 1867.

Recently a specimen in the Zoological Museum of the Zoological Institute of the Russian Academy of Sciences in St. Petersburg was attributed to the Seba collection (Hendriks, pers.com. 11 June 2020). This is a mounted skin, with what looks like young in the pouch. The Seba collection bought by Tsar Peter in 1716 was Seba’s first collection; the specimens of the plates in Seba (1734) are generally based on Seba’s second collection (Bauer et al. 2024: 10). The specimen in St. Petersburg could also be a paralectotype, but information is lacking.

***Philanderquica* (Temminck, 1824)**

*Didelphisquica* Temminck, 1824b: 36.

*Didelphislarvata* Jentink, 1888: 220 (in synonymy).

Paralectotype, RMNH.MAM.12848 (Jentink 1888: 220 *d*), subadult male, mounted skin, skull in situ. Loc.: [Southeastern] Brazil. Leg.: J. Natterer, [October 1817–February 1821]. Ex: NMW, 1821.

Paralectotypes: RMNH.MAM.12839, 12840, 24304.

Temminck (1824b: 37) received a considerable number of animals of this species. Jentink (1888) only listed two mounted skins as types, but there is confusion regarding these specimens. The pedestals bear original inscriptions in Temminck’s handwriting, very faded and difficult to read and sometimes contradictory to Jentink (1887, 1888) or subsequent labels.

Jentink (1888: 220, cat. a) specified RMNH.MAM.12846 as: “Femelle adulte montée, un des types de l’espèce. Brésil. Des collections de M[onsieur] Natterer. *Didelphislarvata*”. However the pedestal reads: “Did. larvata Surynam”. Obviously, the locality Suriname was overlooked by subsequent workers and, to make matters worse, the labels, and consequently the entries in Jentink’s catalogue, have become mixed up. Temminck (1824b: 37) explicitly stated that the animals called “Quica” are from Brazil. Nowhere in these paragraphs is there any reference to specimens from Suriname. This can only mean that Jentink’s skin a (RMNH.MAM.12846) has become mislabelled (apart from the subsequent confounding of labels) as being from Brazil, and is not a type of *Didelphisquica* Temminck, 1824. Of Temminck’s “plusieurs individus montés” in Naturalis, apparently only one is left.

Specimen RMNH.MAM.12848 is described by Jentink (1888: 220; cat d) as: “Mâle à-peu-près adulte monté, un des types de l’espèce. Brésil. Du Cabinet de M[onsieur] Temminck. *Didelphiscrassicaudata* Desmarest”. Jentink’s specification “Du Cabinet de M. Temminck” can be explained by the fact that in 1821 Temminck’s private collection, though already state-owned at the time, had not yet been transferred to the newly founded Leiden Museum; it was gradually moved to Leiden during later years, after extension of the museum building (Gijzen 1938: 33–34; Holthuis 1995: 17–18). The information on its stand reads: “Did. Crassicaudata et Cayapollin Brésil”. Temminck (1824b: 45–46) discussed the various names then in use for these opossums, among which “Cayapollin”, which he regarded as unidentifiable. Although Temminck’s writing on the pedestal does not give Natterer as its collector, it is beyond doubt from that source, since according to Temminck (1824b: 37) this specimen was collected by Natterer and sent from the NMW under the name *Quica*. According to Engelberger (in litt. 2010) the card-file of Natterer’s collection in the NMW confirms that one animal, a male, was sent to Temminck. In a list of exchanges between Vienna and Leiden in August 1821 (Naturalis archives inv. 241.952) one of the entries is “*Didelphiscrassicaudata*. Desm.”, which can only be RMNH.MAM.12848, given Temminck’s inscription.

Natterer collected in Brazil between 1817 and 1835 (von Pelzeln 1871, 1883; Vanzolini 1993). Von Pelzeln (1883: 110) specified the localities and months where Natterer collected this species, stating that 15 animals (one of them alive) were sent to Vienna. Given the date of the exchange in 1821, the Naturalis specimen must have been collected during one of Natterer’s first three journeys in Brazil, between October 1817 and February 1821, in the area between Rio de Janeiro and Paranaguá (Vanzolini 1993: 19–26).

Hershkovitz (1959: 342) designated a lectotype for *Didelphisquica* Temminck, 1825 [=1824]: an animal collected on 3 March 1818 by Natterer at Sapitiba [Sepetiba], Rio de Janeiro, Brazil. This lectotype selection was in all likelihood based on von Pelzeln (1883: 110), without seeing the actual specimen. He failed to specify in which collection it is housed; it cannot be found in the Vienna Museum and its whereabouts are unknown (Engelberger 2010; Voss et al. 2018).

### ﻿﻿DASYUROMORPHIA Gill, 1872

#### ﻿﻿Thylacinidae Bonaparte, 1838

***Thylacinuscynocephalus* (Harris, 1808) (extinct)**

*Thylacinusharrisii* Temminck, 1824c: 63, pl. VII figs 1–4.

*ThylacinusTemminckii* Brookes, 1828: 30 (nomen nudum).

Syntype, RMNH.MAM.39000 (Jentink 1888: 226 a), male, mounted skin. Loc.: Van Diemen’s Land [Tasmania], Australia.

Syntype, RMNH.MAM.63835 (Jentink 1887: 305 b), male, skull. Loc.: Van Diemen’s Land [Tasmania], Australia.

Temminck (1824c) based his description on two specimens. On p. 63 Temminck emphasised that he had only seen males and therefore had been unable to verify whether the female of this species had a pouch. However, only RMNH.MAM.39000 is a male. Another specimen (RMNH.MAM.39001) is a much smaller female. The incomplete skull RMNH.MAM.63835 figured in Temminck’s pl. VII is undoubtedly a male. It has, however, been associated with RMNH.MAM.39001 by Jentink (1887: 305 *a*), though it is obvious that it cannot have been extracted from the latter. The label (not original) of this skull reads “1828, Londres”, which cannot be correct, since it was figured by Temminck in 1824. The second male skin mentioned by Temminck, probably the one corresponding with this skull, is no longer present in Naturalis. The female must have been acquired at a later date (perhaps in 1828) and become misconnected with the male skull figured by Temminck.

A fragmented skull of a female, also catalogued by Jentink (RMNH.MAM.63838), is probably the one really belonging to the skin RMNH.MAM.39001, so this may be the specimen obtained in London in 1828 at the auction of the Brookesian Museum of Comparative Anatomy. Temminck visited this collection during an earlier stay in London and we know from the Museum Annual Report of 1828 (Naturalis archives inv. 40.9) that several specimens were bought at the auction. In his description of *harrisii* Temminck mentioned a specimen in the museum of “Brooks” [sic] (1824c: 65), this could have been the female acquired in 1828. In the catalogue of the sale of 1828 (Brookes 1828: 30, Fifth Day’s Sale. Mammalia, lot 16–18) three specimens of *Thylacinus* were offered for sale: “16 - stuffed specimen, 17 - Foetus, stuffed, 18 - Cranium”. Remarkably in the catalogue this species is listed as *Peracyon Harrisii* and *ThylacinusTemminckii* so with the name given by Temminck and naming it after Temminck. The latter name was published by Brookes (1828) as a nomen nudum.

In the catalogue of the sale of 1830 (Twentieth Day, page 106, Comp. E.I. Comparative Crania, chiefly exotic, in Bottles, lot 13) “the cranium of a young Dog-headed *Dasyurus* (*Peracyon Harrissi* Brookes)” is offered. Whether this is a remainder of the 1828 sale, or a specimen acquired by Joshua Brookes after 1828 is unclear.

#### ﻿﻿Dasyuridae Goldfuss, 1820

***Dasyurusalbopunctatus* Schlegel, 1880**

*Dasyurusalbopunctatus* Schlegel, 1880: 51.

Holotype by monotypy, RMNH.MAM.37157 (Jentink 1887: 304 *a*; 1888: 225 *a*), female, mounted skin and skull. Loc.: Sapua, [at the foot of the] Arfak Mts., New Guinea, Indonesia. [Leg. W.H. Woelders]. Received from G.A. Frank, Sr., 1879.

The collector of this specimen is not recorded. According to Schlegel (1880: 53), it was “a Dutch missionary collecting in the range of the Arfak-mountains”. This can only have been W.H. Woelders, at the time living near Andai at the foot of the Arfak Mts, who was on leave in The Netherlands around 1879, bringing his collections with him (Adriani 1895).

***Murexialongicaudata* (Schlegel, 1866)**

*Phascogalelongicaudata* Schlegel, 1866c: 356.

Holotype by monotypy, RMNH.MAM.35135 (Jentink 1887: 303 *a*; 1888: 223 *a*), subadult male, relaxed mount and incomplete skull. Loc.: Wonumbai, Kobroör Island, Aru Islands, Indonesia. Leg.: C.B.H. von Rosenberg, [4 June] 1865.

This specimen almost certainly is no. 81 in von Rosenberg’s field catalogue, preserved in the archives of Naturalis (inv. 255.486). It was collected on 4 June 1865 and entered as *Mijioictis wallaceë*.

***Myoictismelas* (Müller, 1840)**

*Phascogalemelas* Macklot, 1830: 174 (nomen nudum).

*Phascogalemelas* Müller, 1840b: 20.

Lectotype, RMNH.MAM.25750 (Jentink 1887: 304 *a*; 1888: 223 *a*), male, mounted skin and incomplete skull. Loc.: Lobo, Triton Bay, New Guinea, Indonesia. Leg.: S. Müller [and H.C. Macklot], August 1828.

Macklot (1830) published a list of specimens in his account of the Triton expedition to New Guinea. He introduced several new names, but without providing descriptions.

Müller (1840b) was the first to publish a brief description, later followed by the more extensive treatise by Müller and Schlegel (1843: pl. 25 figs 1–3; 1845: 149); see Husson and Holthuis (1955) for the dates of publication. Müller gave no indication of the number of specimens available to him. Jentink (1887: 304) designated the lectotype.

The material collected around Triton Bay is usually attributed to S. Müller and H.C. Macklot, without distinction. The expedition of the Dutch navy vessel “Triton” was stationed in this bay between 4 July and 29 August 1828 (Macklot 1829: 306). The animal was brought on board by a local hunter (Müller and Schlegel 1845: 152). For the locality see Müller (1840b: 14, map).

*Phascogaleathorbeckiana* Schlegel, 1866b: 257.

Lectotype, RMNH.MAM.25749 (Jentink 1887: 304 *c*; 1888: 224 *c*), male, mounted skin and skull. Loc.: Sailolof, Salawati, Indonesia. Leg.: H.A. Bernstein, [February 1865].

Paralectotype: RMNH.MAM.36166.

Schlegel (1866b: 257) based his description on two specimens of *Phascogale[a*] collected by Bernstein in February 1865. However, there is no *Phascogalea* mentioned in Bernstein’s field notes (see Van Musschenbroek 1883), nor in his notebook of collected specimens, preserved in the archives of Naturalis (inv. 232.517). Only a “*Perameles*” obtained by his hunters near Sailolof is listed in the entry for 22 February 1865 (Van Musschenbroek 1883: 152), which caused Tate (1940: 2) to erroneously suggest this date as the date of collection. However, there is no *Perameles* collected by Bernstein in the Naturalis collection, nor is any such specimen mentioned by Schlegel (1866c: 353–354) in his notes on *P.doreyanus*. Probably Bernstein had misidentified the animal(s). He died on 19 April 1865 on the neighbouring island of Batanta (Van Musschenbroek 1883: 104) where he had received some additional material from Salawati shortly before his death: see his notebook and Schlegel (1866c: 358), discussed further under *Pseudochiruluscanescens*. See also Bernstein’s incomplete and confusing records of kangaroos, discussed under *Dorcopsismuelleri*.

Tate (1940: 2; 1947: 140) erroneously included specimens *a* and *b* in Jentink (1888: 223) in the type series; however, these were collected in 1867 by Hoedt. Jentink (1887: 304) designated the lectotype, unnecessarily repeated by Woolley (2005: 333), who mistakenly recorded it under RMNH 25749c, the paralectotype under RMNH 25749d.

***Neophascogalelorentzii* (Jentink, 1911)**

*PhascogaleLorentzii* Jentink, 1911a: 234.

Holotype by monotypy, RMNH.MAM.36163, female, skin and skeleton, 4 pouch young in alcohol. Loc.: Hellwig Mts at 2600 m (bivouac 8), south of Wilhelmina (Trikora) Peak, New Guinea, Indonesia. Leg.: H.A. Lorentz, Dutch New Guinea Expedition 1909/1910 (329), 24 October 1909.

For the locality, see Lorentz (1913: map).

***Phascolosorexdoriae* (Thomas, 1886)**

*PhascogaleNouhuysii* Jentink, 1911a: 235.

Holotype by monotypy, RMNH.MAM.36174, female, skin and skeleton. Loc.: Went Mts at c. 1050 m (bivouac 4), south of Wilhelmina (Trikora) Peak, New Guinea, Indonesia. Leg.: H.A. Lorentz, Dutch New Guinea Expedition 1909/1910, 12 October 1909.

In the original description Jentink (1911a) gave as collecting locality “Bivak Island” and not “Bivak 4”. This was a mistake he clarified in a later publication (Jentink 1911b: 181). For the locality see Lorentz (1913: 92, map).

***Phascomurexianaso* (Jentink, 1911)**

*Phascogalenaso* Jentink, 1911a: 236.

Holotype by monotypy, RMNH.MAM.35134, male, skin and skeleton. Loc.: Hellwig Mts at c. 2000 m. (bivouac 7), south of Wilhelmina (Trikora) Peak, New Guinea, Indonesia. Leg.: H.A. Lorentz, Dutch New Guinea Expedition 1909/1910 (326), 16 October 1909.

For the locality, see Lorentz (1913: map).

### ﻿﻿PERAMELEMORPHIA Ameghino, 1889

#### ﻿﻿Peramelidae Gray, 1825

***Peroryctesraffrayanarothschildi*** (Förster, 1913)

*Peramelesrothschildi* Förster, 1913: 177.

Holotype by monotypy, RMNH.MAM.553, female, skin, skull, and vertebrae. Loc.: Mt. Bolan (Bangeta) at 3600 m, Saraweget Range, Papua New Guinea. Leg.: 1912. Ex: F. Förster, 8 November 1915.

Förster himself did not collect in New Guinea. He received his material from local collectors and sent it to various museums in Europe. The present specimen was among the mammals obtained by Förster in 1912 and was offered to Jentink in a letter dated 29 January 1913 (Naturalis archives inv. 230.213–214). After some disagreement about the price, Naturalis finally received it on 8 November 1915.

*Peramelesmainois* Förster, 1913: 178.

Syntype, RMNH.MAM.291, female, skin and skull. Source area of Bulung River at 1800–2000 m, Huon Peninsula, Papua New Guinea. Leg.: [1912]. Ex: F. Förster, 22 February 1913.

See *Peramelesrothschildi* (above) for details on the provenance of this specimen. The skeleton accompanying the skin as specified by Förster turned out to be incomplete and only the skull has been preserved. Another syntype is located in the MfN (A.45,23; see Tate 1948).

### ﻿﻿DIPROTODONTIA Owen, 1866

#### ﻿﻿Phalangeridae Thomas, 1888

***Ailuropsursinus* (Temminck, 1824)**

*Phalangistaursina* Temminck, 1824a: 10, pl. I figs 1–3, pl. II figs 1–5, pl. IV.

Syntype, RMNH.MAM.39005 (Jentink 1888: 236 *a*), male, mounted skin, skull extracted but not in collection. Loc.: [Gorontalo], Sulawesi, Indonesia. Leg.: C.G.C. Reinwardt, [17–23 September 1821].

Syntype, RMNH.MAM.39006 (Jentink 1888: 236 *b*), female, mounted skin, skull in situ. Loc.: [Gorontalo], Sulawesi, Indonesia. Leg.: C.G.C. Reinwardt, [17–23 September 1821].

Syntype, RMNH.MAM.39007 (Jentink 1888: 236 *c*), female, mounted skin, skull extracted but not in collection. Loc.: [Gorontalo], Sulawesi, Indonesia. Leg.: C.G.C. Reinwardt, [17–23 September 1821].

Syntype, RMNH.MAM.39008 (Jentink 1887: 313 *a*), sex unknown, skeleton with incomplete skull. Loc.: [Gorontalo], Sulawesi, Indonesia. . Leg.: C.G.C. Reinwardt, [17–23 September 1821]. Figured in Temminck (1824a: pl. I figs 1–3, pl. IV).

Syntype, RMNH.MAM.39009 (Jentink 1887: 314 *c*), juvenile, sex unknown, incomplete skull. Loc.: [Gorontalo], Sulawesi, Indonesia. Leg.: C.G.C. Reinwardt, [17–23 September 1821].

Temminck (1824a: 12) attributed the specimens he had before him to Reinwardt, specifying them as two large individuals, two adult skeletons and several juveniles both as skins and skeletons; another specimen from the same origin is in the MNHN. However, Naturalis has three adult skins from Sulawesi that give Reinwardt as the collector, written on the pedestals in Temminck’s handwriting. Reinwardt’s inventory of the collection that he brought with him to Leiden in 1822, preserved in the archives of Naturalis (inv. 255.139–141), enumerates two skeletons of the “koeskoes van de Celebes” [Cuscus from Sulawesi] for crate no. 4, and two “Koeskoes vellen van Celebes” [Cuscus skins from Sulawesi] for crate no. 11. These must relate to this species, as Temminck had no other cuscus material from Sulawesi. These discrepancies cannot be solved. In crate no. 10, there were two specimens (obviously skins) from the “Celebes-[koeskoes]”, but to those Temminck has added in pencil “B[alantia]. chrysorrhos - Cul d’or”, so they must have been included in that form and cannot have come from Sulawesi (see below).

Skull *a* (RMNH.MAM.39008), figured by Temminck (1824a) in pls I and IV, is now damaged; the skull figured in pl. II is no longer present in the collection. Tate (1940: 4) gave an erroneous list of “Co-types” and erroneously recorded Müller and Macklot as the collectors.

Reinwardt visited northern Sulawesi between 17 September and 14 November 1821 (Reinwardt 1858: 505, 604). He wrote that he found the species near Gorontalo (p. 515), where he stayed between 17 and 23 September (pp. 505, 522). He did not record it from other localities, which renders Gorontalo the most likely provenance of this series.

***Phalangerorientalis* (Pallas, 1766)**

*Phalangistacavifrons* Temminck, 1824a: 17, pl. I figs 7–9, pl. II figs 7–10.

Lectotype, RMNH.MAM.59143 (formerly 39002a) (Jentink 1887: 310 *m*^3^), juvenile, sex unknown, cranium. Loc.: [Banda or Ambon Island], Moluccas, Indonesia. Leg.: [C.G.C. Reinwardt, May–August 1821].

Paralectotypes: RMNH.MAM.16433, 39371, 59144, 59660.

Temminck (1824a: 19) gave as the origin for this species the islands of Banda and Ambon, and recorded a series of skins and skeletons of all ages for the Naturalis collection; the collector was not recorded. On the same page, Temminck referred to Reinwardt’s activities in the Malay Archipelago, but in a general way only, not specifically in connection with this material. Our specimens are badly documented and lack original labels. We follow Jentink (1887), who attributed RMNH.MAM.59143 and 16433 to Reinwardt, and Smeenk, who did the same for RMNH.MAM.39371 and 59660 on their labels, and that they all belong in the type series of *Phalangistacavifrons* Temminck, 1824.

Reinwardt visited the Banda Islands between 18 May and 26 June, Ambon and surrounding islands between 27 June and 12 August 1821 (Reinwardt 1858: 374, 424, 476). In this travel account, he recorded “*Didelphisorientalis*” for Ambon only (p. 435, see also below), so at first sight it would seem likely that he had obtained the species on that island. However, the inventory of the collection that he brought with him to Leiden in 1822 (Naturalis archives inv. 255.139–141) gives the following entries apparently relating to this species: two skeletons of the “koeskoes van Banda” [cuscus from Banda] in crate no. 4; seven “witte koeskoes” [white cuscus] in crate no. 10, to which Temminck has added in pencil “Balantia naevia” (obviously males) and three “Graauwe koeskoes” [Grey cuscus], to which Temminck has added “B[alantia]. fasciata” (probably females), both unpublished manuscript names. In crate no. 11, there were five “Koeskoes vellen van Banda” [Cuscus skins from Banda], to which has been added in pencil: “verteerd” [decayed], so these have not been preserved. Finally, for the same crate Temminck has added in pencil: “2 Balantia Cavifrons”. The loss of several specimens, however, does not explain the discrepancy between Temminck’s reported “série d’individus” and the present lack of skins among the types. Further in his travel account, Reinwardt (1858: 515) again briefly referred to the occurrence of cuscuses on Ambon, and in a footnote gave a description that includes two species: “De Ambonsche soorten zijn wit en rosachtig of wit met zwarte vlekken” [The Ambon species are white and reddish or white with black spots]. The former must refer to *Ph.orientalis* (= *cavifrons*), the latter to *Spilocuscusmaculatus*, which Temminck specifically distinguished from his *Ph.chrysorrhos* (see below).

Probably following Jentink (1887: 310), Tate (1940: 3) wrongly regarded the juvenile skull RMNH.MAM.59143 as “the type”, thereby inadvertently designating it the lectotype of *Phalangistacavifrons* Temminck, 1824. This is very unfortunate, particularly since Tate correctly observed that “The mandible associated with the skull does not belong to it”. The lectotype therefore comprises the cranium only, the mandibles are here listed as paralectotypes (RMNH.MAM.59144).

***Spilocuscusmaculatus* (E. Geoffroy, 1803)**

*Phalangistachrysorrhos* Temminck, 1824a: 12.

Lectotype, RMNH.MAM.13484 (Jentink 1887: 312 *n*; 1888: 234 *l*), female, mounted skin and skull. Loc.: Ambon, Moluccas, Indonesia. Leg.: C.G.C. Reinwardt, [27 June–12 August 1821]. Skull figured in Temminck (1824a: pl. I figs 4–6).

Paralectotypes: RMNH.MAM.13485, 39391.

Temminck (1824a: 13) attributed the two specimens and an unspecified number of skulls he had before him to Reinwardt. Since the skull of RMNH.MAM.13485 has not been extracted, there must have been at least one more, separate, skull collected by Reinwardt. The only such skull now present in Naturalis is RMNH.MAM.39391, which therefore should be included in the type series, a fact overlooked by Jentink (1887) and Tate (1940: 3).

Temminck (1824a: 14) gave as its origin the Moluccas, the island unknown. The pedestals, however, give Ambon as the locality, in Temminck’s own handwriting. The inventory of the collection that Reinwardt brought to Leiden in 1822 (Naturalis archives inv. 255.139–141) contains an entry of two specimens (obviously skins) of the “Celebes-[koeskoes]” for crate no. 10, to which Temminck has added in pencil: “B[alantia]. chrysorrhos - Cul d’or”. This vernacular designation agrees perfectly with the colouration of the skins and with Temminck’s description on p. 13. They must be the two types mentioned by Temminck and so cannot have come from Sulawesi (see under *Ailuropsursinus*). Reinwardt worked on Ambon between 27 June and 12 August 1821 (see under *Phalangerorientalis*).

The lectotype was designated by Husson (1955: 294).

***Spilocuscuswilsoni* Helgen & Flannery, 2004**

*Spilocuscuswilsoni* Helgen & Flannery, 2004: 826.

Holotypе, RMNH.MAM.12727, juvenile male, skin and skeleton. Loc.: Biak Island, New Guinea, Indonesia. Leg.: L.D. Brongersma, April 1955. Received 17 April 1906.

Paratype: RMNH.MAM.64.

#### ﻿﻿Pseudocheiridae Winge, 1893

***Petauroidesvolansincanus* Thomas, 1923**

*Petauroidesvolansincanus* Thomas, 1923a: 247.

Paratype, ZMA.MAM.5380, male, skin and skull. Loc.: Eidsvold, Australia. Leg.: T.V. Sherrin, 18 January 1922. Ex: NHM, 1923.

Thomas (1923a; 247) designated NHMUK 22.12.29.19 in the NHM as the type, making it the holotype in accordance with ICZN Article 73.1.1.. See also Bergmans (2011: 836).

***Petropseudesdahlii* (Collett, 1895)**

*Pseudochirusdahlii* Collett, 1895: 464.

Syntype, RMNH.MAM.13392, female, skin and skull with incomplete left mandible. Loc.: Mary River (13°30'S, 131°30'E), Northern Territory, Australia. Leg.: K. Dahl, 7 May 1895. Ex: Naturhistorisk museum (Oslo), R. Collett, 1897.

***Pseudocheirusperegrinus* (Boddaert, 1785)**

*DidelphisPeregrinus* Boddaert, 1785: 78.

*Didelphiscaudivolvula* Kerr, 1792: 196.

*DidelphisNovae Hollandiae* Bechstein, 1799: 348, 685.

*PhalangistaBanksii* Gray, 1838: 107.

Holotype for *D.peregrinus and D.novae hollandiae*, syntype for *D.caudivolvula* and *P.banksii*, RMNH.MAM.33660 (Jentink 1888: 239 *i*), female, mounted skin, skull in situ. [Loc.: Endeavour River, Queensland, Australia. Leg.: J. Banks, 26 July 1770, the first expedition by J. Cook. Received from Bullock’s Museum, London, 1819].

Smeenk (2009) extensively discussed the history of this specimen and the taxonomy of this species.

***Pseudocheirusperegrinusconvolutor* (Schinz, 1821)**

*PhalangerViverrina* Ogilby, 1838a: 131.

Syntype, RMNH.MAM.39011 (Jentink 1887: 315 *c*; 1888: 239 *j*), female, mounted skin and skull. Loc.: Van Diemen’s Land [Tasmania], Australia, 1837, no further documentation; acquired in London, 1838.

The pedestal of RMNH.MAM.39011 bears the inscription by Temminck: “Phalangista indiv. décrit par Ogilby”. Jentink (1885: 23) mentioned it as “One of the specimens described by Ogilby in 1837 s.n. *Phalangistaviverrina*”. Ogilby’s description, read for the Zoological Society on 28 November 1837, appeared in the following year (Ogilby, 1838a).

Tate (1945: 26) referred to a specimen in the NHM (NHMUK 1855.12.24.213) as “Type”. In the online database of the NHMhttps://data.nhm.ac.uk/object/3ec0da83-e2f8-437e-a3c1-25f1115e33a1/1733788800000) this specimen is listed as holotype. These are not valid lectotype designations and all specimens should be listed as syntypes.

***Pseudochiruluscanescens* (Waterhouse, 1846)**

*Phalangistabernsteinii* Schlegel, 1866c: 357.

Lectotype, RMNH.MAM.13389 (Jentink 1887: 315 *a*; 1888: 238 *a*), female, mounted skin and incomplete skull. Loc.: Salawati Island, Indonesia. Leg.: H.A. Bernstein, [April 1865]. Received 20 January 1866.

Paralectotype: RMNH.MAM.13390.

Bernstein collected on Salawati between 21 February and 31 March 1865 (see Van Musschenbroek 1883), and his notebook of collected specimens is preserved in the archives of Naturalis (inv. 232.517). In these, however, there is no record of this species (Van Musschenbroek 1883: 152 nor under *Phascogaleathorbeckiana* above and *Dorcopsismuelleri* below). Schlegel (1866c: 358) therefore supposed that Bernstein had received the specimens shortly before his death; he died on 19 April 1865 on the neighbouring island of Batanta. His diary from the final weeks of his life is very brief (Van Musschenbroek 1883: 102–105), but during his stay on Batanta he did receive additional material from Salawati. The last entry in his notebook is of 14 April, recording three birds from Salawati, but this is his only record from the period of 9 April until his death. The year 1866 mentioned by Jentink (1888) is the date when the collection was received in Leiden.

The lectotype was designated by Husson (1955: 296).

***Pseudochirulusschlegelii* (Jentink, 1884)**

*Pseudochirusschlegelii* Jentink, 1884: 110.

Holotype, RMNH.MAM.13388 (Jentink 1887: 315 *a*; 1888: 238 *a*), male, mounted skin and skull. Loc.: Arfak Mts, New Guinea, Indonesia. [Leg. W.H. Woelders]. Ex: G.A. Frank, Sr., 1879.

The collector of this specimen is not recorded. Almost certainly this was the Dutch missionary W.H. Woelders, who lived near Andai at the foot of the Arfak Mts and was back on leave in The Netherlands around 1879 (Adriani 1895). The animal was acquired in April 1879 through the dealer G.A. Frank, Sr. in Amsterdam.

#### ﻿﻿Potoroidae Gray, 1821

***Bettongialesueur* (Quoy & Gaimard, 1824)**

*Hypsiprymnus Graii* Gould, 1840a: 178.

Syntype, RMNH.MAM.63735 (Jentink 1888 253: *d*), juvenile female, mounted skin, skull in situ. Loc.: Swan River, Australia. Ex: J. Gould.

The population or form described as *graii* by Gould (1840a) is extinct (Burbridge and Short 2023).

***Potoroustridactylustridactylus* (Kerr, 1792)**

*Hypsiprymnusmyosurus Ogilby*, 1838e: 62.

Syntype, RMNH.MAM.63737 (Jentink 1888: 252 *d*), adult, sex unknown, mounted skin, skull in situ. Loc.: Australia. Ex: W. Ogilby.

On the stand of RMNH.MAM.63737 is written in Temminck’s hand: “decri individu par Ogelby” [Described specimen by Ogilby]. We therefore list this specimen as a syntype. We do not include another specimen (RMNH.MAM.64141, with the same data) in the type series, as it lacks this information.

In Calaby and Richardson (1988) and the online database of the NHM a specimen (NHMUK 1855.12.24.66; https://data.nhm.ac.uk/object/7f5ae9a3-b6b9-4c86-a241-f1eac8a014e9/1733961600000) is listed as holotype. Ogilby did not mention how many specimens he has before him, nor where they are kept, therefore according to ICZN art. 74.5 this does not constitute a valid lectotype designation and all types should be listed as syntypes.

#### ﻿﻿Macropodidae Gray, 1821

***Dendrolagusinustus* Müller, 1840.**

*Hypsiprymnusartus* Macklot, 1830: 174 (nomen nudum).

*Dendrolagusinustus* Müller, 1840b: 20.

Lectotype, RMNH.MAM.13483 (Jentink 1887: 324 *a*; 1888: 250 *d*), female, mounted skin and skeleton. Loc.: Mt Lamantsjieri [Lamanciri], Triton [Lobo] Bay, New Guinea, Indonesia. Leg.: S. Müller and H.C. Macklot, August 1828.

Macklot (1830) published a list of specimens collected in his account of the Triton expedition to New Guinea. He introduced several new names, but without providing a description.

Müller (1840b) provided a short description, followed by a more extensive description by Schlegel and Müller (1841: pls 20, 22–24; 1845c: 143). Müller (1840b) did not indicate the number of specimens available to him. Jentink (1887: 324) designated the lectotype.

The animal was caught by a local hunter in the forest of Mt Lamanciri and kept alive for some time on board the Dutch naval vessel “Triton” (Schlegel and Müller 1845c: 145–146). In the archives of Naturalis (not catalogued) there is a drawing of its head by P. van Oort. The expedition stayed in Triton Bay between 4 July and 29 August 1828 (Macklot 1829: 306). See Müller (1840b: 14, pl. 3, map) for the locality.

***Dendrolagusursinus* (Temminck, 1836)**

*Hypsiprymnusursinus* Macklot, 1830: 174 (nomen nudum).

*Hypsiprimnusursinus* Temminck, 1836: vi.

Lectotype, RMNH.MAM.13507 (Jentink 1887: 323 *a*; 1888: 249 *b*), female, mounted skin and skeleton. Loc.: Lobo (Triton) Bay near Mt Lamantsjieri [Lamanciri], New Guinea, Indonesia. Leg.: S. Müller and H.C. Macklot, July 1828.

Paralectotypes: RMNH.MAM.16450, 39012–39013.

Macklot (1830) published a list of specimens collected in his account of the Triton expedition to New Guinea. He introduced several new names, but without providing descriptions. Temminck (1836) was the first author to provide a description.

The four animals were caught by local hunters and kept alive on board the Dutch naval vessel “Triton”. Three of them were slaughtered and eaten by the crew in relief of the many sick members of the expedition. One adult female was taken as a pet to Ambon and from there to Timor, where it lived for some time (Schlegel and Müller 1845b: 142–143); this must have been RMNH.MAM.39012. In the archives of Naturalis (not catalogued) there are two drawings of this tree-kangaroo by P. van Oort: one showing the entire animal, dated 6 August 1828, the other a detailed portrait of the head, dated August 1828. These were copied in Schlegel and Müller’s pls 19 and 22 fig. 1, respectively. Jentink (1888: 249) refers in these plates to skin *b* (RMNH.MAM.13507), but that specimen has a whitish tip to the tail (now damaged), which is lacking in the drawing and plate. The expedition was in Triton Bay between 4 July and 29 August 1828 (Macklot 1829: 306). See Müller (1840b: 14, pl. 3, map)for the locality.

The lectotype was designated by Mees (1957: 209); see also Husson and Rappard (1958: 10).

***Dorcopsisluctuosaphyllis* Groves & Flannery, 1989**

*Dorcopsisluctuosaphyllis* Groves & Flannery, 1989: 125.

Holotype, ZMA.MAM.5243, female, skin and incomplete skeleton. Loc.: Near Merauke, New Guinea, Indonesia. Leg.: A.J.N. Monsanto, end of 1959–early 1960 (captured alive) died in NAM, 31 December 1962.

Paratype: RMNH.MAM.17239.

The animal was sold to NAM in 1959/1960, where it died after about two years. Other paratypes are in AMNH (AMNH 105982 and 105998) and the Western Australian Museum (WAM M2808). See Bergmans (2011: 836–837) for further details.

***Dorcopsismuellerimuelleri* (Schlegel, 1866)**

*Hypsiprymnusdoreo* Macklot, 1830: 174 (nomen nudum).

*MacropusMülleri* Schlegel, 1866c: 353.

Lectotype, RMNH.MAM.13473 (Jentink 1887: 322 *a*; 1888: 250 *b*), female, mounted skin and skeleton. Loc.: [Southern foot of Mt Lamantsjieri (Lamanciri)], Lobo (Triton) Bay, New Guinea, Indonesia. Leg.: S. Müller and H.C. Macklot, July 1828. Figured in Schlegel and Müller (1841: pl. 21, pl. 23 figs 7–8, pl. 24 figs 7–9).

Paralectotypes: RMNH.MAM.13474–13482.

Macklot (1830) published a list of specimens collected in his account of the Triton expedition to New Guinea. He introduced several new names, but without providing descriptions.

Schlegel and Müller (1841: pls 21–24) figured this small kangaroo from New Guinea under the name *Hypsiprymnusbrunii* (Schreber, 1778), later (Schlegel and Müller 1845b: 131) followed by the text. In this species, the authors included the animals from New Guinea and the Aru Islands, though leaving open the possibility that these might eventually prove distinct species (p. 133). Schlegel (1866c: 350–353) demonstrated that the two forms are very different indeed and showed that Schreber’s *Didelphisbrunii* applies to the species occurring in the Aru Islands. He abandoned the generic name *Dorcopsis* and named the animals from New Guinea and Salawati *Macropusmuelleri*. Husson (1955: 296) used the name *Dorcopsisveterum* (Lesson, 1827) for the New Guinea species, but Groves and Flannery (1989: 117) regarded this as a nomen dubium.

The lectotype of *Macropusmuelleri* Schlegel, 1866 was designated by Husson (1955: 300), following the suggestion by Tate (1940: 4). There can be no doubt that this is the female carrying a young in her pouch, which Müller and Macklot shot at the southern foot of Mt. Lamanciri, as recorded in Schlegel and Müller (1845b: 138); see Müller (1840b: 14, pl. 3, map) for the locality. The juvenile in alcohol (RMNH.MAM.13476) is her young. The other animals were caught alive by local hunters and brought on board the Dutch naval vessel “Triton”. Jentink (1888) also attributed the head, figured in Schlegel and Müller (1841b: pl. 22 fig. 3), to the female with young. This is probably incorrect: the figure is based on a drawing by G. van Raalten (not P. van Oort as stated by Schlegel and Müller 1845b: 147) of a live animal dated Lobo, August 1828, so probably represents one of the other individuals, which were not all captured in July as stated by Jentink. In his field notes from Lobo in the Naturalis archives (not catalogued), Müller recorded this species for “Julij–August”. The expedition in Triton Bay was between 4 July and 29 August 1828 (Macklot 1829: 306).

Jentink (1887, 1888) did not mention that the specimens collected by Bernstein were types, although Schlegel (1866c: 351) included collections from “Salawatti et sur différents points de la côte occidentale de la Nouvelle-Guinée”. At the time, Naturalis had just received Bernstein’s collection on 20 January 1866. Bernstein collected in New Guinea in the surroundings of Sorong and outlying islets between 22 November 1864 and 19 February 1865, and on Salawati from 21 February to 31 March 1865 (apart from a brief visit on 19–20 November 1864); see Van Musschenbroek (1883) and his notebook of collected specimens (Naturalis archives inv. 232.517). One of the Sorong kangaroos was obtained by Bernstein’s hunters on 27 December 1864; the other, a larger male, on 2 January 1865, some distance up the Ramui River above Sorong (see Bernstein’s notebook and Van Musschenbroek 1883: 69, 71, 137, 139). Strangely, Bernstein recorded both kangaroos as *Dendrolagus*, but there are no tree kangaroos collected by him in Naturalis, only *Dorcopsis*. This enigma cannot be solved. It seems difficult to believe that Bernstein would have confused the two genera, since for Salawati, he does mention two *Dorcopsis* collected at Kalwal on 17 March 1865 (see his notebook and Van Musschenbroek 1883: 159). There is another discrepancy here: Bernstein’s notes recorded two fairly small animals from Salawati (probably RMNH.MAM.13478–13479), but there are four in Naturalis. Probably, he had received additional specimens from Salawati during his stay on the neighbouring island of Batanta, where he died on 19 April 1865 (Van Musschenbroek 1883: 104). Adding to the confusion, Jentink (1887; 1888) has interchanged the two animals from Sorong. The smaller juvenile (skull *p*, RMNH.MAM.13482) is the one collected on 27 December 1864; the adult male (skin *q*, skull *o*, RMNH.MAM.13481), dated by Jentink 17 December 1865 (a lapsus for 27 December 1864), is the one obtained on 2 January 1865. The year 1866 given by Jentink for the remaining specimens refers to the date when the collection was received in Leiden (20 January 1866; Naturalis archives inv. 232.517).

Groves (2005a: 62) erroneously listed this species as *Dorcopsismuelleri* (Lesson, 1827), confounding the authors Schlegel and Lesson.

***Dorcopsismuellerilorentzii* Jentink, 1909**

*DorcopsisLorentzii* Jentink, 1909a: 10, pl. I figs a–d.

Lectotype, RMNH.MAM.24254, male, flat skin and skull. Loc.: [“Geluksheuvel” near] Alkmaar, New Guinea, Indonesia. Leg.: Dutch New Guinea Expedition 1907, H.A. Lorentz, 13 August 1907. Figured in Jentink (1909: pl. I figs a–d).

Paralectotypes: RMNH.MAM.24253, 24255, 24264.

Some sources (Groves 2005a: 62; Eldridge and Coulson 2020: 92) dated the publication of this name in 1908, based on the print date October 1908 in the header of the article. However the first issue of Nova Guinea vol. 9 was published in 1909 (see title page).

The lectotype was designated by Groves and Flannery (1989: 122), following an unpublished note on the label by A.M. Husson. See Van Nouhuys (1910: map, “Geluksheuvel”) and Lorentz (1913: map, “Glückshügel”) for maps of the collecting locality of the lectotype.

***Macropusfuliginosus* (Desmarest, 1817)**

*Kangurusfuliginosus* “Péron et Lesueur” Desmarest, 1817b: 35.

Syntype, RMNH.MAM.19689 (Jentink 1887: 318 *j*; 1888: 243 *g*), subadult female, mounted skin and skull. Loc.: Ile Decrès [Kangaroo Island], Australia. Leg.: C.-A. Lesueur, [2 January–1 February 1803], Baudin Expedition. Ex: MNHN.

We follow Jackson et al. (2021a) by listing Lesueur as collector and not Péron and Lesueur. However, Jackson et al. (2021a) are in error by listing a specimen in the MNHN as holotype (MNHN-ZM-MO-1990-396) and another (MNHN-ZM-MO-2014-17) as paratype, probably following De Beaufort (1966: 549) and Julien-Laferrière (1994: 25). Desmarest (1817b) had at least a male and female of the species, so all material available to him should be considered syntypes.

***Petrogalelateralislateralis* (Gould, 1842)**

*MacropusLateralis* Gould, 1840b: 685.

Paralectotype, RMNH.MAM.64134 (Jentink 1887: 322 *a*; 1888: 248 *a*), adult female, mounted skin and skull. Loc.: York, Western Australia, Australia. Ex: J. Gould.

Paralectotype, RMNH.MAM.64135.

McAllen and Bruce (1989) already pointed out that the original publication for this species is in the journal The Atheneum (Gould 1840b) and not in his ‘Monograph of the Macropodidae’ (Gould 1842: pt 2, 24). The latter date is still followed by Eldridge and Coulson (2020: 98). Thomas (1922: 128) designated a lectotype in the NHM (NHMUK 1842.5.26.3).

***Notamacropuseugenii* (Desmarest, 1817)**

*KangurusEugenii* Desmarest, 1817b: 38.

Syntype, RMNH.MAM.59662 (Jentink 1888: 246 *d*), subadult male, mounted skin, skull in situ. Loc.: Ile Eugene [Iles of St. Peter], Australia. Leg.: F. Péron [10 February 1803], Baudin expedition, [1802–1803]. Ex: MNHN.

Desmarest (1817b) based his description on specimens collected by C.A. Lesueur on Ile Eugène (Péron 1816: 117). This was probably during the visit of St. Peter Island by the ship ‘Le Géographe’ on 10 February 1803 (Jackson et al. 2021a: 413).

*Halmaturus Houtmannii* Gould, 1844: 31.

Paralectotype, RMNH.MAM.63740 (Jentink 1887: 320 *a*; 1888: 246 *a*), subadult male, mounted skin and skull. Loc.: Houtman Abrolhos, E. Wallabi Island. Ex: J. Gould. Received 18 January 1843.

Paralectotype: RMNH.MAM.63741.

The information on the labels with these specimens is confusing: the collecting date, 18 January 1843, and locality are in conflict with the listing of Gould as the collector, ‘Voyage Gould’. Gould returned from Australia in 1840 and never visited Western Australia. This is presumably the date of arrival of the specimens in Naturalis.

It is possible that these specimens were collected by Benjamin Bynoe, surgeon on board of the ‘Beagle’, which visited these islands in May 1840 during its third voyage. Bynoe (in Stokes 1846: 156–161) described how he found and collected specimens of this species.

A specimen in the NHM (NHMUK 1844.2.15.10) was designated the lectotype by Thomas (1922: 128).

***Osphranterrobustus* (Gould, 1841)**

[*Macropus*] *Robustus* Gould, 1840b: 685.

Paralectotype, RMNH.MAM.63733 (Jentink 1887: 318 *a*; 1888: 244 *a*), adult female, mounted skin and skull. Loc.: Liverpool Range, Australia. Leg.: J. Gould, [September 1838–April 1840].

Paralectotype: RMNH.MAM.63734.

Until now (Groves 2005a: 65; Eldridge and Coulson 2020: 104) the publication of this name was dated from the Proceedings of the Zoological Society of London (Gould 1841: 92). However the first mention of this name appeared in The Atheneum (Gould 1840b). In the first article no type locality is given; in the later publication (Gould 1841) the type locality is described as “mountain ranges in the interior of New South Wales”.

Thomas (1922: 128) designated a specimen (NHMUK 1841.1099) in the NHM as the lectotype.

***Osphranterrufus* (Desmarest, 1822)**

*Kanguruslaniger* Gaimard, 1823: 481.

Holotype by monotypy, RMNH.MAM.63732 (Jentink 1887: 318 *a*; 1888: 243 *a*), subadult male, mounted skin and skull. Loc.: Port Macquarie, Australia. Leg.: C. Fraser, [1819]. Received by J.P. Gaimard, Uranie expedition. Ex: MNHN.

Gaimard received this specimen from Fraser during the stay of the expedition of the ‘Uranie’ at Port Jackson (13 November–25 December 1819). The animal was shot by Fraser near Port Macquarie (Gaimard 1823: 481), probably during the Oxley Expedition to Port Macquarie in 1819. Gaimard (1823) later named this species *Kangaroos laniger*, without any reference to the name introduced by Desmarest.

***Onychogaleafrenata* (Gould, 1840)**

*M. [acropus] frenatus* Gould, 1840b: 685.

Syntype, RMNH.MAM.63738 (Jentink 1887: 321 *a*; 1888: 249 *a*), adult male, mounted skin and skull. Loc.: Liverpool Plains, Australia. Leg.: J. Gould, [September 1838–April 1840].

Until now (Groves 2005a: 66; Eldridge and Coulson 2020: 100) the publication of this name is dated from in the Proceedings of the Zoological Society of London (Gould 1841: 92). However the first mention of this name appeared in The Atheneum (Gould 1840b). Also note the spelling of the specific epithet in Gould 1840b: *frenatus* and not *fraenatus*. A specimen in the NMH (NHMUK 1841.1130) was listed by Thomas (1888b: 77) as “Type”. This was followed in Jenkins and Knutson (1983), Calaby and Richardson (1988), and the NHM online database (https://data.nhm.ac.uk/object/05cac01c-61c1-434a-9b2c-9c8c26f3b9db/1737590400000), that lists this specimen as the holotype. This does not qualify as a valid lectotype designation according to ICZN art. 74.5 and all types should be treated as syntypes.

***Thylogalebillardierii* (Desmarest, 1822)**

*Macropusrufiventer* Ogilby, 1838d: 23.

Syntype, RMNH.MAM.63742 (Jentink 1887: 320 *a*; 1888: 245 *c*), subadult female, mounted skin and skeleton. Loc.: Tasmania, Australia. Leg.: J. Gould, [September 1838–April 1840].

Ogilby (1838d) based his description of this new species on material in the collection of Gould. Thomas (1888b: 60) listed a specimen in the NHM as “type” for *rufiventer*. This was followed by Jenkins and Knutson (1983: 25) and Calaby and Richardson (1988) who listed NMHUK 1853.8.29.58 as the holotype. However, from the data it is not clear that this specimen came from Gould: its provenance only lists the Zoological Society. As Ogilby (1838d) did not indicate that the specimen was in the collection of the Zoological Society, nor specified the number of specimens available to him, there is no certainty this is the only type. Therefore according to ICZN art. 74.5 these are not valid lectotype designations and all type specimens should be treated as (possible) syntypes.

We do not include two other specimens in Naturalis (RMNH.MAM.63743–63744, also collected by Gould in Tasmania), in the type series. Only the stand of RMNH.MAM.63742 bears a reference to the name *rufiventer*.

##### ﻿Incertae sedis

**Macropus (Halmaturus) psilopus Gould, 1840b: 685.**

In a list of specimens sent by Gould to Naturalis preserved in the archives (inv. 208.298–299) a specimen with the name M. (Halmaturus) psilopus is mentioned. This name was published by Gould in 1840, but has never been accurately ascribed to any valid species. Finding the specimen exchanged with Naturalis under that name could resolve this issue. However, we have not been able to locate this particular specimen in the Naturalis collection.

### ﻿﻿AFROSORICIDA Stanhope et al., 1998

#### ﻿﻿Tenrecidae Gray, 1821

***Hemicentetesnigriceps* Günther, 1875**

*Hemicentetesvariegatus*, **var. buffoni** Jentink, 1879f: 150.

Holotype by monotypy, RMNH.MAM.39003 (Jentink 1887: 246 *a*; 1888: 124 *a*), sex unknown, subadult, mounted skin and incomplete skull. Loc.: Madagascar. Leg.: A. Crossley, [1872–1875]. Ex: G.A. Frank, Sr., 1875.

Crossley collected in Madagascar in 1860, again in 1869, and finally from 1872 until early 1875 (see Tattersall 2022). Many of his specimens were sold through natural history dealers. RMNH.MAM.39003 was acquired in August 1875 from the dealer G.A. Frank, Sr. in Amsterdam. It is thus likely that it was collected during the 1870s.

***Potamogalevelox* Du Chaillu, 1860**

*Potamogale Allmani* Jentink, 1895: 236.

Jentink’s new species is based on the description of a specimen by Allman (1866: 4). The holotype (NMS Z.1864.23) is at the National Museums of Scotland (Herman et al. 1990).

#### ﻿﻿Chrysochloridae Gray, 1825

***Calcochlorisobtusirostris* (Peters, 1851)**

*Chrysochlorisobtusirostris* Peters, 1851: 467.

Syntype, RMNH.MAM.39015 (Jentink 1887: 248 *a*; 1888: 128 *b*), subadult, sex unknown, relaxed mount and skull. Loc.: Inhambane, Mozambique. Leg.: W.C.H. Peters, [1843–1847]. Ex: MfN, 25 May 1851.

This new species was published in a preliminary communication by Peters (1851) that preceded the extensive description published in his definitive work the following year (Peters 1852: 70–75, pls XVIII, XXII). Although in both publications he gave the measurements of one animal only, he mentioned five additional specimens (p. 74), which he had all available in the previous year and so they all belong in the type series. An undated note (Naturalis archives inv. 247.168–169) lists species to be received from Peters includes “Chrysochloris n. sp.”, to which is added “mosambicensis” by Temminck. The shipment was received in Leiden on 25 May 1851 and includes “1 Chrysochloris mosambicensis Jeune indiv”. There can be no doubt that the Naturalis specimen is one of the five mentioned by Peters. The label by Jentink reads “1842–1847”, but Peters arrived in Mozambique in 1843; he returned to Germany in 1848 (Peters 1852: viii–ix).

Turni et al. (2007: 7) listed other syntypes in the MfN (ZMB 719, 720, 12945, 31020, 85341).

### ﻿﻿MACROSCELIDEA Butler, 1956

#### ﻿﻿Macroscelididae Bonaparte, 1838

***Petrodromustetradactylus* Peters, 1846**

*Petrodromustetradactylus* Peters, 1846: 258.

Syntype, RMNH.MAM.39312 (Jentink 1887: 243 *a*; 1888: 119 *a*), subadult, sex unknown, mounted skin and skull. Loc.: Tete, Mozambique. Leg.: W.C.H. Peters, [1843–1846]. Ex: MfN, 25 May 1851.

This species was first described in a preliminary communication, which preceded the extensive description in his definitive work (Peters 1852: 92–100, pl. XX). In his first note Peters gave the measurements of one animal only. In his later work, Peters (1852: 100) again did not specify numbers, but wrote that he found this species in several places; on pp. 98–99, he gave the measurements of three animals: two females and one male. RMNH.MAM.39312 is from Tete and, given the date of Peters’ first communication, cannot have been collected later than in 1846. It is mentioned in a list preserved in the archives of Naturalis (inv. 247.168) specifying animals from Mozambique received in exchange with the MfN in June 1850; the specimen was then in alcohol. It arrived probably the following year, since the specimen (there is only one in Naturalis) also appears in a list of mammals received from Peters on 25 May 1851 (Naturalis archives inv. 247.169). Peters arrived in Mozambique in 1843 and returned to Germany in 1848 (Peters 1852: viii–ix).

***Rhynchocyoncirneicirnei* Peters, 1847**

*RhynchocyonCirnei* Peters, 1847: 37.

Lectotype, RMNH.MAM.39313 (Jentink 1888: 120 *a*), male, relaxed mount, skull extracted but not in collection. Loc.: [near Quelimane], Bororo district, Mozambique. Leg.: W.C.H. Peters, [1843–1846]. Ex: MfN, 1851.

This species was also first described in a preliminary communication (Peters 1847) preceding the extensive description published in his definitive work (Peters 1852: 106–110, pls XXI–XXIV). There can be no doubt that RMNH.MAM.39313 is one of the types. From the description from 1847 and in 1852 (p. 110) it is clear Peters had collected two specimens, a male and a female, and Peters (1852: 110) added that the male was sent in exchange to Leiden. It is included in the list of specimens received in Leiden on 25 May 1851, as “1 Rhynchorgos [lapsus for Rhynchocyon] Cirnei en peau” (Naturalis archives inv. 247.168–169).

Peters (1847) states that he spent two months on Mr. Cirne’s family estate “im Distrikt Bororo (Quellimane)”, but did not specify the period. This could well have been in 1846, as Peters (1852: 47) recorded that he collected bats in his house “im März 1846, in Boror, etwa 12 Meilen nordwestlich von Quellimane”.

The lectotype was designated by Corbet and Hanks (1968: 57).

***Rhynchocyoncirneireichardi* Reichenow, 1886**

*Rhynchocyon Reichardi* Reichenow, 1886: 316.

Syntype, RMNH.MAM.39314 (Jentink 1887: 243 *a*; 1888: 120 *b*), female, mounted skin and skull. Loc: Kwa Mpesa, Marungu, Democratic Republic of Congo. Leg.: R. Böhm [and P. Reichard], 4 August 1883. Ex: Reichenow, 1886.

Reichenow (1886) gave the measurements of two specimens, a male and a female collected by Böhm and Reichard.

### ﻿﻿HYRACOIDEA Huxley, 1869

#### ﻿﻿Procaviidae Thomas, 1892

***Dendrohyraxdorsalissylvestris* (Temminck, 1853)**

*Hyraxsylvestris* Temminck, 1853: 182.

Syntype, RMNH.MAM.39236 (Jentink 1887: 160 *a*; 1892: 189 *b*), female, mounted skin and skull. Loc: Gold Coast (coastal Ghana). Leg.: H.S. Pel, [1841–1849].

Syntype, RMNH.MAM.39237 (Jentink 1887: 160 *b*, pl. 4 figs 5–6; 1892: 189 *c*), female, mounted skin and skull. Loc: Coastal Ghana. Leg.: H.S. Pel, [1841–1849].

Syntype, RMNH.MAM.39238 (Jentink 1892: 189 *d*), juvenile male, mounted skin, skull in situ. Loc: Dabocrom (Dabo Krom), coastal Ghana. Leg.: H.S. Pel, [February 1843].

Syntype, RMNH.MAM.39239 (Jentink 1892: 189 *e*), juvenile female, mounted skin, skull in situ. Loc: Dabo Krom, coastal Ghana. Leg.: H.S. Pel, [1841–1849].

Although Temminck (1853) did not specify how many specimens he had before him, from his description (p. 184) it is clear that there are at least one adult and one or more juveniles. Most inventories of Pel’s shipments are preserved in the archives of Naturalis (Inv. 247.112–114). In these, the following specimens of *Hyraxarboreus* are mentioned: a male from Sekondi dated November 1841; a male from Dabo Krom dated February 1843; a female from Apam sent in October 1844; and an unspecified animal from Dabo Krom sent on 6 March 1849. The only Naturalis specimen that bears a date is RMNH.MAM.39238. Its pedestal reads “voy Pel Dabocrom feb 1841” in Temminck’s handwriting. The village of Dabocrom (Dabo Krom) is situated between Butri and Sekondi and was one of Pel’s favourite collecting localities (Holthuis 1968: 9, 22–23). However, in February 1841 Pel was at Elmina (letter from Pel of 5 April 1841 in Holthuis 1968: 10); the collection date of this specimen must be February 1843 when Pel visited Dabo Krom (Holthuis 1968: 10) and which would agree with Pel’s shipping inventory of October 1844, where he mentions a “*Hyraxarboreus*. Mas. feb 1843”. It is also possible that one or more of Pel’s specimens listed above were collected during his second sojourn, so were not included in Temminck’s treatise, and therefore are not types, but this cannot be traced.

The species is usually referred to as *Dendrohyraxdorsalis* (Fraser, 1854). Fraser’s *Hyraxdorsalis* was described in the Proceedings of the Zoological Society of London for 1852, but that volume appeared on 23 May 1854 (Allen 1939: 443) contra Sclater (1893: 439) who dates the publication on 7 December 1852; Shoshani (2005: 87) and Shultz and Roberts (2013: 155) date it from 1855. If Allen is correct Temminck’s name *Hyraxsylvestris* should have priority.

***Hyraxstampflii* Jentink, 1886b: 209.**

Holotype by monotypy, RMNH.MAM.39240 (Jentink 1887: 160 *a*, pl. 4 figs 1–4; 1892: 190 *a*), female, mounted skin and skeleton. Loc: Chap hill, Schieffelinsville on Junk River, Liberia. Leg.: F.X. Stampfli, 3 February 1886.

For the locality, see Büttikofer (1888: 60, pl. 5; 1890a: map). The animal was obtained by a local hunter (Büttikofer 1890b: 390).

### ﻿﻿PROBOSCIDEA Illiger, 1811

#### ﻿﻿Elephantidae Gray, 1821

***Elephasmaximussumatranus* (Temminck, 1847)**

*ElephasSumatranus* Temminck, 1846: 328 (nomen nudum).

*ElephasSumatranus* Temminck, 1847: 90.

Syntype, RMNH.MAM.39234 (Jentink 1887: 169 *a*), male, skeleton. Loc: Palembang, Sumatra, Indonesia. Received from J.C. Baud, 1845.

Syntype, RMNH.MAM.39235 (Jentink 1887: 169 *b*), female, skeleton. Loc: Palembang, Sumatra, Indonesia. Received from J.C. Baud, 1845.

Temminck (1846) introduced this name without a description, and the formal description appeared one year later (Temminck 1847) without a specification of the type series. Jentink (1887: 169) catalogued the Sumatran elephants under *Elephassumatranus* Temminck, but did not list the above two skeletons as types.

Temminck tried to resolve the debate about the taxonomic status of the Sumatran elephant and sent a request for specimens to Baud, the Dutch Minister for the colonies at the time (Van Wingerden 2023: 104). Three specimens (two from Lampong, one from Palembang) were sent to Temminck; however, according to Temminck all of them were incomplete, so a second request followed. It is unknown whether this second request resulted in more specimens and to what shipment the two remaining types in the Naturalis collection belong.

Although listed by Jentink (1887: 169) as female, RMNH.MAM.39235 is likely a male (pers. comm. H. van Essen and A. Lister, 2023).

### ﻿﻿SCANDENTIA Wagner, 1855

#### ﻿﻿Tupaiidae Gray, 1825

***Dendrogalemurina* (Müller, 1840)**

*Hylogalemurina* Müller, 1840a: 25, 50.

Lectotype, RMNH.MAM.36120 (Jentink 1887: 241 *a*; 1888: 118 *a*), male, relaxed mount and incomplete skull. Loc: “Pontianak, Borneo” [error for Cochin, Vietnam]. Leg.: P.-M. Diard, [May 1821–1824].

Müller’s description (1840a) preceded the more extensive account (as *Hylogaleamurina*) by Schlegel and Müller (1843: pls 26–27; 1845c: 160, 167); see Husson and Holthuis (1955) for the dates of publication. Müller gave no indication of the number of specimens available to him. The lectotype was designated by Jentink (1887: 241).

Schlegel and Müller (1845c: 167) gave as type locality Pontianak, Borneo. Lyon (1913: 130–131) is the first author to question this provenance. He pointed out that the species had never again been found in Borneo and that the type specimen is very similar to *Dendrogalefrenata* (Gray, 1860) from Cambodia and Annam. He therefore suspected that the type is wrongly labelled. This is very likely, as Diard collected in Indochina from May 1821–1824 (Dorai and Low 2021: 35). The present specimen apparently became mixed up with the material that Diard collected in Borneo in 1826.

***Tupaiadorsalis* Schlegel, 1857**

*Tupajadorsalis* Schlegel, 1857: 59, pl. III fig. 31.

Syntype, RMNH.MAM.35995 (Jentink 1887: 240 *f*; 1888: 116 *b*), subadult, sex unknown, relaxed mount and skull. Loc: [Lower] Kapuas [Bohang] River, Borneo, Indonesia. Leg.: C.A.L.M. Schwaner, [28 January–2 February 1848].

Syntype, RMNH.MAM.35996 (Jentink 1888: 116 *g*), subadult, sex unknown, relaxed mount, skull in situ. Loc: [Lower] Kapuas [Bohang] River, Borneo, Indonesia. Leg.: C.A.L.M. Schwaner, [28 January–2 February 1848].

There are two rivers in Borneo named Kapuas: the Kapuas Bohang in western Borneo, and the Kapuas Murung in the southeast. Schwaner visited both rivers, the latter on 21–22 November 1847. Between 28 January and 2 February 1848 he travelled down the Kapuas Bohang, from Sintang to Pontianak (Schwaner 1854: 39–44, 188–200). Since Schlegel (1857) described this species only briefly, Jentink (1890b: 228–230) redescribed the species extensively, giving the locality as “North Western Borneo, in the neighbourhood of the Kapoeas-river.”

***Tupaiaglis* (Diard, 1820)**

*Tupaiaglis phœniura* Thomas, 1923b: 255.

Paratype, ZMA.MAM.11310, juvenile female, alcohol. Loc: Deli Estate near Medan, Sumatra, Indonesia. Leg.: L.P. Cosquino de Bussy, [1905–1917]. Ex: NHM, 1923.

The holotype is in the NHM (NHMUK 23.1.2.30). See also Bergmans (2011: 837).

***Tupaiajavanica* Horsfield, 1822**

*Tupaiajavanicabogoriensis* Sody, 1937: 213.

Holotype, RMNH.MAM.34090, female, skin and skull. Loc: Kota Batu, Bogor, Java, Indonesia, at 300 m. Leg.: H.J.V. Sody, 3 June 1924.

Paratypes: RMNH.MAM.34091–34093, 34096–34097.

Sody (1937) examined six specimens for his description. See Becking (1989: 47, 80–81) for details on the locality and collector.

*Tupaiajavanicatjibuniensis* **Sody, 1937: 213.**

Holotype, RMNH.MAM.34207, male, skin and skull. Loc: Tjiboeni (Cibuni) Estate, Mt Patuha, 1350 m, Java, Indonesia. Leg.: M. Bartels, Jr., 15 August 1932 (Tjib. 68).

Paratypes: RMNH.MAM.34205–34206, 34208–34219.

Sody (1937) examined 14 specimens for his description. See Becking (1989: 47, 80–81) for details on the locality and collector.

***Tupaiaminorhumeralis* Robinson & Kloss, 1919: 257.**

Holotype, RMNH.MAM.12654, female, skin and skull. Loc.: Rimbupengadang (Lebong), Barisan Mts at c. 1000 m, Bengkulen (Bengkulu) District, Sumatra, Indonesia. Leg.: E. Jacobson, 26 June 1916 (72).

Paratypes: RMNH.MAM.12652–12653.

Robinson and Kloss (1919: 266, 282) studied four animals, although in their table on p. 282 one additional specimen is listed. Three of these are present in Naturalis.

***Tupaiatanatana* Raffles, 1821**

*Tupaiatananainggolani* **Sody, 1936a: 54.**

Holotype, RMNH.MAM.34120, male, skin and skull. Loc.: Perlak [Peureulak], Sumatra, Indonesia. Leg.: F.J. Nainggolan, 16 October 1930. Ex: H.J.V. Sody (F.109).

Paratypes: RMNH.MAM.34119, 34121.

The holotype was obtained by a native collector through F.J. Nainggolan, who collected for Sody. See Becking (1989: 46, 87, 229) for details on the locality and collector.

### ﻿﻿Dermoptera Illiger, 1812

#### ﻿﻿Cynocephalidae Simpson, 1945

***Galeopterusvariegates* Audebert, 1799**

*Galeopithecusmarmoratus* Temminck, 1828: 66.

Temminck (1828) published this name as a nomen nudum.

##### ﻿Incertae sedis

***Galeopithecusmacrourus* Temminck, 1836: ix.**

Temminck (1836) published this name as a nomen nudum. Temminck wrote that he had a skeleton without a skull; however, this specimen is no longer in Naturalis.

### ﻿﻿PRIMATES Linnaeus, 1758

#### ﻿﻿Cheirogaleidae Gray, 1873

***Microcebusmurinus* (J.F. Miller, 1777)**

*Lemurpusillus* É. Geoffroy Saint-Hilaire, 1791: 89.

This name was published by É. Geoffroy Saint-Hilaire (1791) in a description based on three animals in the MNHN collected by Poivre and Sonnerat and he further referred to a drawing in Commerson’s manuscripts and to a live animal in Paris, seen by Buffon.

Schlegel (1876: 326) and Jentink (1887: 67; 1892: 78) listed a specimen in Naturalis (RMNH.MAM.39050) as belonging to the type series. The label (not original) of our specimen does not mention the locality or collector, but says: “obt: en 1815. du Musée de Paris”. The pedestal of the mounted skin bears an inscription in Temminck’s handwriting, most of which has become illegible. Over this is written, in another hand: “1815. Sganzin O Madagascar”; probably, the year 1815 has been added later as the pedestal of the skull reads only “Sganzin. Madagascar”. Year and collector are mutually exclusive. Sganzin worked in Madagascar during 1831 and 1832; he was the military commander based on Sainte Marie Island off NE Madagascar (Sganzin 1840: 2–3; Pollen 1863), from where he made expeditions through the adjacent mainland. Specimens were brought to him by local hunters. He donated most of his collections to the MNHN.

Schlegel (1876: 326) wrote that the specimens seen by him are from NE Madagascar, opposite of Nosy Boraha (l’île de St. Marie). This is the area covered by Sganzin, so the inscription on the pedestal “O” (= Ouest, possibly added later) to “Madagascar’’ must be erroneous. The MNHN did indeed send a large collection to Leiden in 1815, in compensation for the Kabinet des Stadhouders that had been taken by Napoleon in 1795 (Gijzen 1938: 23–28; Holthuis 1995: 11–14). Sganzin’s animal, of a much later date, must inadvertently have become catalogued with this 1815 consignment. Jentink (1887; 1892) copied Schlegel’s data, but it is clear that the Naturalis specimen cannot be one of Geoffroy’s types.

***Mirzazaza* Kappeler & Roos, 2005 in Kappeler et al. 2005: 18**

*Microcebuscoquereli* Schlegel, 1867: 419 (nomen nudum).

*MicrocebusCoquereli* Schlegel & Pollen, 1868: 12, pls 6, 7 (nec Grandidier 1867).

*Mirzazaza* Kappeler & Roos in Kappeler et al. 2005: 18.

Holotype for *M.zaza*, syntype for *M.coquereli*, RMNH.MAM.39377 (Jentink 1887: 66 *d*; 1892: 77 *c*), male, mounted skin and skull. Loc.: Kongony, Madagascar. Leg.: F.P.L. Pollen and D.C. van Dam, 25 September 1865.

Syntype for *M.coquereli*, paratype for *M.zaza*, RMNH.MAM.39376 (Jentink 1887: 66 c; 1892: 77 b), subadult female, mounted skin and skull. Loc.: Pasandava Bay [Ampasindava], Madagascar. Leg.: D.C. van Dam. Received 1868.

Syntype for *M.coquereli*, paratype for *M.zaza*, RMNH.MAM.54999 (formerly 39375) (Jentink 1887: 66 b; 1892: 77 a), adult female, mounted skin and skull. Loc.: Pasandava Bay [Ampasindava], Madagascar. Leg.: D.C. van Dam. Received 1868.

Schlegel (1867) introduced this new name, without description, in an account of mammals and birds of Madagascar collected by Pollen and Van Dam, which were to be fully described in a forthcoming publication (Schlegel and Pollen 1868). The description only mentioned the holotype; two specimens received later by Van Dam are listed in an appendix to this work (p. 172). We could not ascertain if the appendix was published separately in a later issue and for the moment list all three as syntypes, following ICZN art. 72.4.1. Kappeler et al. (2005: 18) erroneously gave Pollen and van Dam as the collectors and 1868 as collecting date for the latter specimens. Jentink (1887; 1892) did not list any specimens as types.

In the meantime, however, Grandidier (1867: 85) had described a lemur from Morondava, western Madagascar, as *CheirogalusCoquereli*; his description appeared in March 1867. The authors clearly were not aware of each other’s actions and their publications appeared independently (see Schlegel and Pollen 1868: ix). The animals from W and NW Madagascar have since been regarded as conspecific, with Grandidier’s name taking priority, until Kappeler et al. (2005) recognised the population of NW Madagascar as a separate species, *Mirzazaza*. Kappeler et al. (2005) were unaware of the fact that RMNH.MAM.39377 is also a syntype of *Microcebuscoquereli* Schlegel & Pollen, 1868.

Jentink (1887; 1892) attributed the other two specimens to Pollen and Van Dam 1868. However, their joint expedition to Madagascar lasted from November 1863 to July 1866. Van Dam returned later to Madagascar alone and collected there between 1867 and 1873. The specimens collected jointly by Pollen and Van Dam are carefully documented, whereas many of those obtained by Van Dam alone bear less precise labels. In the appendices to their work, Schlegel and Pollen (1868: 172) already referred to the animals collected by Van Dam in 1867, recording the total number of *M.Coquereli* then in Naturalis as three (p. 164). The year 1868 given by Jentink must therefore refer to the year when this material arrived in Leiden (erroneously used as the year of collection by Fransen et al. 1997: 281).

***Phanerpallescens* Groves & Tattersall, 1991**

*Phanerfurciferpallescens* Groves & Tattersall, 1991: 40.

Paratype, RMNH.MAM.36343 (Jentink 1887: 65 *h*; 1892: 77 *i*), male, mounted skin and skull. Loc.: Morondava River, Madagascar. Leg.: D.C. van Dam, August 1870.

Paratypes: RMNH.MAM.36344–36348.

Groves and Tattersall (1991) described this form from SW Madagascar, based on 18 skins and 15 skulls. In their table of skull measurements (p. 40), the authors specify 13 skulls, including six in Naturalis.

***Phanerparienti* Groves & Tattersall, 1991**

*Phanerfurciferparienti* Groves & Tattersall, 1991: 40.

Holotype, RMNH.MAM.36338 (Jentink 1887: 65 *c*; 1892: 77 *d*), male, mounted skin and skull with damaged mandible. Loc.: Sjangoi [Djangoa], Madagascar. Leg.: F.P.L. Pollen and D.C. van Dam, 5 September 1865.

Paratypes: RMNH.MAM.36339–36341.

Groves and Tattersall (1991) described this form from NW Madagascar, based on a study of eight skins and six skulls, which are not all specified. In their table of skull measurements (p. 40), the authors strangely do not include the holotype although its skull is undamaged.

#### ﻿﻿Lemuridae Gray, 1821

***Eulemurcollaris* (É. Geoffroy Saint-Hilaire, 1812)**

*Prosimiamelanocephala* Gray, 1863: 137, pl. XVIII.

Gray (1863) described *P.melanocephala* without indicating the number and provenance of the specimens he had before him, but referred to specimens in the NHM. Since he described both the adult and juvenile pelage, he must have had at least one animal of each category. Jenkins (1987: 11), however, gives an adult female in the NHM (NHMUK 1855.12.24.55) as the holotype of this form.

A specimen in Naturalis (RMNH.MAM.39066, collected by Crossley at Tannanariva, Madagascar, received through Frank Sr. in 1875) has a remark on the label: “un des types de *Pr.melanocephalus*, Gray”, a statement also given by Schlegel (1876: 307–308) and Jentink (1887: 60; 1892: 70). This is almost certainly incorrect, as there is no indication that this specimen has been part of the NHM collection.

***Eulemurfulvus* (É. Geoffroy Saint-Hilaire, 1796)**

*Lemurbruneus* Van der Hoeven, 1844: 35 (nomen novum).

Van der Hoeven (1844) introduced this new name as in his opinion the name *fulvus* did not agree with its appearance and with the French vernacular name of “Maki brun”. The two specimens mentioned in his description (RMNH.MAM.39051, 39052) and listed as types in Jentink (1892: 70) are therefore not types (ICZN art. 72.7).

*Lemurmayottensis* Schlegel, 1866a: 76.

Syntype, RMNH.MAM.39054 (Jentink 1887: 60 *bb*), sex unknown, skull (skin probably RMNH.MAM.39063). Loc.: Jongoni [Longoni] Bay, Mayotte, Comoro Archipelago. Leg.: F.P.L. Pollen and D.C. van Dam, 8 May 1864.

Syntype, RMNH.MAM.39055 (Jentink 1887: 60 *s*; 1892: 70 *b*^3^), male, mounted skin and skull. Loc.: Jongoni Bay, Mayotte. Leg.: F.P.L. Pollen and D.C. van Dam, 19 May 1864.

Syntype, RMNH.MAM.39056 (Jentink 1887: 60 *t*; 1892: 70 *c*^3^), subadult male, mounted skin and skull. Loc.: Jongoni Bay, Mayotte. Leg.: F.P.L. Pollen and D.C. van Dam, 9 June 1864.

Syntype, RMNH.MAM.39057 (Jentink 1887: 60 *u*; 1892: 70 *d*^3^), male, mounted skin and skull. Loc.: Jongoni Bay, Mayotte. Leg.: F.P.L. Pollen and D.C. van Dam, 7 June 1864.

Syntype, RMNH.MAM.39058 (Jentink 1887: 60 *v*; 1892: 70 *e*^3^), male, mounted skin and skull. Loc.: Jongoni Bay, Mayotte. Leg.: F.P.L. Pollen and D.C. van Dam, 8 May 1864.

Syntype, RMNH.MAM.39059 (Jentink 1887: 60 *w*; 1892: 70 *f*^3^), male, mounted skin and skull. Loc.: Jongoni Bay, Mayotte. Leg.: F.P.L. Pollen and D.C. van Dam, 8 May 1864.

Syntype, RMNH.MAM.39060 (Jentink 1887: 60 *x*; 1892: 70 *g*^3^), male, mounted skin and skull. Loc.: Jongoni Bay, Mayotte. Leg.: F.P.L. Pollen and D.C. van Dam, 8 May 1864.

Syntype, RMNH.MAM.39061 (Jentink 1887: 60 *y*; 1892: 70 *h*^3^), subadult male, mounted skin and skull. Loc.: Jongoni Bay, Mayotte. Leg.: F.P.L. Pollen and D.C. van Dam, 8 May 1864.

Syntype, RMNH.MAM.39062 (Jentink 1887: 60 *z*; 1892: 70 *i*^3^), adult female, mounted skin and skull. Loc.: Jongoni Bay, Mayotte. Leg.: F.P.L. Pollen and D.C. van Dam, 19 May 1864.

Syntype, RMNH.MAM.39063 (Jentink 1892: 70 *j*^3^), female, mounted skin, skull probably RMNH.MAM.39054. Loc.: Jongoni Bay, Mayotte. Leg.: F.P.L. Pollen and D.C. van Dam, 10 May 1864.

Syntype, RMNH.MAM.39064 (Jentink 1887: 60 *aa*; 1892: 70 *k*^3^), adult female, mounted skin and skull. Loc.: Jongoni Bay, Mayotte. Leg.: F.P.L. Pollen and D.C. van Dam, 8 May 1864.

Schlegel (1866a) and Schlegel and Pollen (1868: 5) mentioned a series of ten specimens collected in Mayotte in May and June 1864, seven males and three females, which agrees with the material listed above except for the skull RMNH.MAM.39054, without an associated skin. It probably belongs to RMNH.MAM.39063, which lacks an accompanying skull.

The dates given on the labels do not agree in all respects with Pollen’s account of his voyage in Mayotte (Pollen 1868: 110, 133–135), where he described having collected three specimens on a hunting trip on 8 May 1864. Naturalis holds five specimens bearing that date and a further two specimens labelled 19 May. On that day, no mention is made of any lemurs being collected. It is at the moment impossible to correct these inconsistencies.

***Eulemurrubriventer* (I. Geoffroy Saint-Hilaire, 1850)**

*Lemurflaviventer* I. Geoffroy Saint-Hilaire, 1850: 876.

Schlegel (1876: 311) listed RMNH.MAM.39053 (a female collected by Bernier in NE Madagascar, received from the MNHN in 1835) as one of the types. However, I. Geoffroy Saint-Hilaire (1850) based his description on material in the MNHN he had hitherto overlooked. As RMNH.MAM.39053 had already left the MNHN in 1835 we do not consider it part of the type series. It is specified in a list of mammals received in exchange in June 1835 (Naturalis archives inv. 241.915), as “Maki roux à gorge blanche”. Its pedestal reads “Espèce nouvelle, voyage Bernier”, in Temminck’s handwriting.

*Prosimiarufipes* Gray, 1871b: 339.

Gray (1871b) described this species based on material in the NHM collected by Crossley; he does not mention the collecting date nor the precise locality, and does not specify the number of specimens he had before him. Jenkins (1987: 20) listed two type specimens in the NHM (NHMUK 1870.5.5.35 and 1870.5.5.36). Schwarz (1931: 417) designated NHMUK 1870.5.5.35 the lectotype of *Prosimiarufipes* Gray, 1871.

Schlegel (1876: 311), listed RMNH.MAM.39067–39068 (collected by Crossley and received through Frank in 1875 and 1873 respectively) as types of *Prosimiarufipes*. There is no indication these specimens have been part of the NHM collection, so they cannot have been among Gray’s types.

***Eulemurrufus* (Audebert, 1799)**

*Lemurrufus* Audebert, 1799: 12, pl. II.

Syntype, RMNH.MAM.39065 (Jentink 1892: 72 d^5^), female, mounted skin, skull in situ. Loc.: Madagascar. Ex: MNHN, 1815.

Rode (1939: 437), probably unaware of the specimen in Naturalis, listed a specimen in the MNHN (MNHN-ZM-MO-1993-4247) as holotype. According to ICZN art. 74.5 this is not a valid lectotype designation.

#### ﻿﻿Indriidae Burnett, 1828

***Avahilaniger* Gmelin, 1788**

*LichanotusAvahi* Van der Hoeven, 1844: 44, pl. I, fig. 6, pl. III.

Syntype, RMNH.MAM.63664 (Jentink 1887: 57 *e*;1892: 64 *a*), adult male, mounted skin and skull. Loc.: Northeast Madagascar. Leg.: A.C.J. Bernier, [1831]. Ex: MNHN, 1838.

According to Schlegel (1876: 299–300) No. 1 is the specimen depicted by Van der Hoeven with skull No. 7. This was the only specimen in Naturalis at the time of description (Van der Hoeven 1844: 28) and the only specimen seen by him. Van der Hoeven also referred to the description by Jourdan (1834: 232).

*Avahiunicolor* Thalmann & Geissman, 2000: 915.

Holotype, RMNH.MAM.40031 (Jentink 1887: 57 *h*; 1892: 64 *d*), female, mounted skin and skull. Loc.: Kakamba [on the peninsula of Ampasindava, northwestern Madagascar, approximately 1335S, 4757E], Madagascar. Leg.: D.C. van Dam, 1868.

Paratypes: RMNH.MAM.40032–40034, 23118.

During 1863–1866, F.P.L. Pollen and D.C. van Dam made a joint expedition to Madagascar. In 1867 Van Dam returned to Madagascar alone, where he collected until 1873 (see under *Mirzazaza*). Many of his specimens are not precisely documented, and the year 1868 may also refer to the time when the material arrived in Leiden.

***Indriindri* (Gmelin, 1788)**

*Lichanotusmitratus* Peters, 1872: 360.

Schlegel (1876) and Jentink (1887: 53; 1892: 61) recorded RMNH.MAM.39069 (a female from Saralalano, Madagascar, collected by A. Crossley and purchased from G.A. Frank, Sr in 1875) as a type. However Peters (1872) described this form based on a single male specimen in the MfN (ZMB 4008; see Turni 2024: 154), which is therefore the holotype by monotypy for this name.

***Propithecusdeckenii* Peters, 1870**

*PropithecusDamanus*Sclater 1870: 112 (nomen nudum).

*PropithecusDamonis* Gray, 1871a: 137.

*PropithecusDamanus* “Pollen” Schlegel, 1876: 293.

Syntype, RMNH.MAM.39070 (Jentink 1887: 54 *a*; 1892: 64 *a*), adult male, mounted skin and skull. Loc.: southern shore of Bombetoka Bay, Madagascar. Leg.: D.C. van Dam, 1869.

Syntype, RMNH.MAM.39071 (Jentink 1887: 54 *b*; 1892: 64 *b*), adult male, mounted skin and skull. Loc.: Southern shore of Bombetoka Bay, Madagascar. Leg.: D.C. van Dam, 1869.

Syntype, RMNH.MAM.39072 (Jentink 1887: 54 *f*; 1892: 64 *c*), adult male, mounted skin and skull. Loc.: Southern shore of Bombetoka Bay, Madagascar. Leg.: D.C. van Dam, 1869.

Syntype, RMNH.MAM.39073 (Jentink 1887: 54 *c*; 1892: 64 *d*), adult female, mounted skin and skull. Loc.: Southern shore of Bombetoka Bay, Madagascar. Leg.: D.C. van Dam, 1869.

Syntype, RMNH.MAM.39074 (Jentink 1887: 54 *e*; 1892: 64 *e*), adult female, mounted skin and skull. Loc.: Southern shore of Bombetoka Bay, Madagascar. Leg.: D.C. van Dam, 1869.

Syntype, RMNH.MAM.39075 (Jentink 1887: 54 *d*; 1892: 64 *f*), adult female, mounted skin and skull. Loc.: Southern shore of Bombetoka Bay, Madagascar. Leg.: D.C. van Dam, 1869.

Syntype, RMNH.MAM.39076 (Jentink 1892: 64 *g*), juvenile female, mounted skin, skull extracted but not in collection. Loc.: Southern shore of Bombetoka Bay, Madagascar. Leg.: D.C. van Dam, 1869.

Van Dam collected in Madagascar between 1867 and 1873, see under *Mirzazaza*. He failed to document his specimens accurately, so the date of collection cannot be traced.

Schlegel (1876) referred to Pollen as the author of *PropithecusDamanus*, but as far as we could determine Pollen never formally published this name. Sclater (1870: 112) showed a specimen of this new lemur from Pollen to the Zoological Society of London in 1870, received through the dealer G.A. Frank Jr. Sclater (1870) does not give a description. Where this specimen went afterwards is not known. Gray (1871a) published this name for the first time validly as *Propithecusdamonis* (1871: 846) and attributed it to Pollen. Gray referred to specimens in the collection of the NHM, but also to Pollen; therefore, we consider the material mentioned above also as part of the type series. The publication date of the Catalogue of monkeys, lemurs, and fruit-eating bats in the collection of the British Museum is usually dated 1870. However, according to Sherborn (1934) this publication should be dated 1871.

***Propithecusverreauxi* Grandidier, 1867**

*PropithecusVerreauxi* Grandidier, 1867: 84.

Syntype, RMNH.MAM.39077 (Jentink 1887: 55 *b*; 1892: 63 *e*), female, mounted skin and skull. Loc.: Cape Sainte Marie [Cape Vohimena], Madagascar. Leg.: A. Grandidier, [June–November 1866]. Ex: Grandidier, 1867.

Syntype: RMNH.MAM.39078 (Jentink 1887: 55 c; 1892: 63 f), immature female, mounted skin and skull. Loc.: Cape Sainte Marie [Cape Vohimena], Madagascar. Leg.: A. Grandidier, [June–November 1866]. Ex: Grandidier, 1867.

Grandidier (1867) described this species based on a series of eleven adults and seven juveniles collected during his first expedition to western and southern Madagascar, which lasted from June to November 1866 (Dorr 1997: 185). Grandidier presented the specimens above to Naturalis in 1867 (Schlegel 1876: 296), the year given on the labels and pedestals, and thus belong in the type series (see also Voisin et al. 1999: 536).

Rode (1939: 442), probably unaware of the specimens in Naturalis, listed a specimen in the MNHN (MNHN-ZM-MO-1867-580) as holotype. According to ICZN art. 74.5 this is not a valid lectotype designation.

#### ﻿﻿Lorisidae Gray, 1821

***Perodicticuspotto* (Statius Müller, 1776)**

*LemurPotto* Statius Müller, 1776: 12.

*galagoguineensis* Desmarest, 1820: 104.

Neotype, RMNH.MAM.39375 (Jentink 1887: 52 *a*; 1892: 60 *a*), female, mounted skin and skeleton. Loc.: Dabocrom, Gold Coast [Ghana]. Leg.: H.S. Pel [March–September 1849].

The author is mostly cited as P.L.S. Müller, which is incorrect: his family name was Statius Müller. His description of this species is based on an 18^th^-century account and figure of pottos observed at Elmina and surroundings in the Gold Coast, the present-day coastal Ghana (Bosman 1704: 32–33, pl. facing p. 35 fig. 4); none of those animals have been preserved. Smeenk et al. (2006) discussed the need for a neotype and designated RMNH.MAM.39375 the neotype of *Lemurpotto* Statius Müller, 1776 and *Galagoguineensis* Desmarest, 1820.

#### ﻿﻿Galagidae Gray, 1825

***Galagoidesdemidoff* (G. Fischer, 1806)**

*Octolicnus* [sic] *peli* Temminck, 1853: 42.

Syntype, RMNH.MAM.39079 (Jentink 1887: 68 *b*; 1892: 80 *c*), adult male, mounted skin and skull. Loc.: [Dabo Krom], Gold Coast [Ghana]. Leg.: H.S. Pel, [1843–1850].

Syntype, RMNH.MAM.39080 (Jentink 1887: 68 *c*; 1892: 80 *d*), subadult female, mounted skin and cranium. Loc.: [Dabocrom], Ghana. Leg.: H.S. Pel, [1843–1850].

Syntype, RMNH.MAM.39081 (Jentink 1887: 68 *d*; 1892: 80 *e*), sex unknown, juvenile, mounted skin and skull. Loc.: Dabocrom, Ghana. Leg.: H.S. Pel, [1843–1850].

Syntype, RMNH.MAM.25166 (Jentink 1892: 80 *i*), male, alcohol, skull in situ. Loc.: Coastal Ghana. Leg.: H.S. Pel, [1843–1850].

Schlegel (1876: 331) and Jentink (1887; 1892) both overlooked the type status of these specimens, probably because they identified them with Fischer’s *Galagodemidoff*. Temminck (1853: 42–43) at first came to the same conclusion by comparing an undocumented juvenile specimen in Naturalis with Fischer’s description. However, after receiving Pel’s material, he described the animals from the Gold Coast as *Octolinus peli*. Temminck’s original juvenile specimen is no longer present in Naturalis, so the type series only consists of the animals collected by Pel in the Gold Coast, present-day coastal Ghana

Temminck (1853: 45) gave Dabocrom (Dabo Krom) as the collecting locality. Of the Naturalis specimens, only the pedestal of RMNH.MAM.39081 bears this locality, but not in Temminck’s handwriting. In Pel’s letters and incomplete shipping inventories (Naturalis archives inv. 247.112–114) the species is mentioned only once in a letter from Dabocrom dated February 1843 in which he announced that he had received a small specimen which he believed to be *Otolicnus*; this appears in Pel’s shipment inventory of 12 March 1843 as “*Otolicnus*? Mas. feb 1843. van Dabocrom. zeer zeldzaam” [Male. February 1843. from Dabocrom. very rare]. It cannot be determined whether this is the adult male RMNH.MAM.39079 (which was sent in alcohol) or the one still in alcohol, RMNH.MAM.25166. Temminck (1853: 43) only states that he had recently received an old male and old female, which does not quite agree with a specimen that arrived as early as 1843. It cannot be excluded either that one or more of the specimens were collected during Pel’s second sojourn in the Gold Coast (1852–1855), which were not included in Temminck’s treatise and hence are not types, but this cannot be determined.

Another syntype is in the MfN (ZMB 322: see Turni 2024: 155).

#### ﻿﻿Tarsiidae Gray, 1825

***Tarsiuspelengensis* Sody, 1949**

*Tarsiusfuscuspelengensis* Sody, 1949b: 143.

Holotype, RMNH.MAM.9851, male, skin and skull. Loc.: Pulau Peleng, Banggai Archipelago, Indonesia. Leg.: J.J. Menden, 17 August 1938. Ex: MZB (MZB 6605), 12 January 1950.

Sody (1949b) examined eight more specimens for his description; these are not in Naturalis.

***Tarsiussangirensis* Meyer, 1896**

*Tarsiussangirensis* Meyer, 1896: v, 9.

Paralectotype, RMNH.MAM.28641 (Jentink 1892: 81 *g*), female, mounted skin and skull. Sangihe Island, Indonesia, 6 January 1866. Leg.: D.S. Hoedt.

Meyer (1896) described this species from specimens in the MTKD (SNSD B497, B321; see Feiler 1990: 405) and Naturalis. He apparently had not seen the latter himself, but referred to it in the original description. Groves designated a specimen in the MTKD (SNSD B497 (2243)) the lectotype.

Meyer’s description is often dated 1897 (Groves 2005b: 128; Shekelle et al. 2020: 172). However this should be 1896 (Engelberger 2010).

#### ﻿﻿Cebidae Bonaparte, 1831

***Leontocebusnigricollis* (Spix, 1823)**

*Midasnigricollis* von Spix, 1823: 28, pl. XXI.

Syntype, RMNH.MAM.39103 (Jentink 1892: 57 *a*), adult, sex unknown, mounted skin, skull in situ. Loc.: Between Solimões (Amazonas) and Içá Rivers, Brazil. Leg.: J.B. von Spix, [January 1820].

Von Spix (1823: 29) encountered this species near d’Olivença in the neighbourhood of a black water river named Parana-Bijuna, between the Solimõens (Amazonas) and Içá, where he was in January 1820 (von Spix and von Martius 1823–1831: 1186–1190, map).

A list of animals received from von Spix in October 1824, preserved in the archives of Naturalis (inv. 257.853), has two entries: “12) Mydas nigricollis ♂”, and “13) – ♀”. Only one of these specimens is still present in Naturalis, the other had already left the collection in 1876 as Schlegel only listed one specimen in his catalogue (Schlegel 1876: 265).

Kraft (1983: 437) listed two syntypes in the ZSM.

***Leontopithecuschrysomelas* (Kuhl, 1820)**

*Midaschrysomelas* “Wied” Kuhl, 1820a: 51.

Syntype, RMNH.MAM.17691 (Jentink 1892: 55 *a*), female, mounted skin, skull in situ. Loc.: [Ribeirão das Minhocas], upper Ilhéus [Colônia] River, Brazil. Leg.: M. zu Wied [11–16 January 1817].

Kuhl (1820a) described this species from specimens in the MfN, Temminck’s cabinet, and the Wied collection, all collected by Wied. Wied (1826: 159) found this species near “Sertam von Ilhéos”, where he collected between 12 and 16 January 1817 (Wied in Joost 1987: 181–182). De Ávila-Pires (1965: 12) restricted the type locality to Ribeirão das Minhocas, a small tributary of the upper Ilhéus River; see Engländer (1995: 256–257) for the location. The collecting date is from Moraes (2009: 30). The year 1820 given by Jentink is the year when Temminck received the specimen.

Elliot (1913a: 212) gave a description of this species, “taken from the male example in Prince Max.’s Collection purchased by the New York Museum and presumably the type, as it is not supposed that the type of Kuhl’s description would be permitted to leave the collection.” De Ávila-Pires (1965: 11–12) misquoted É. Geoffroy Saint-Hilaire (1851: 62) by stating that “the type was a male specimen in the collection of the Museum National d’Histoire Naturelle”. However, É. Geoffroy Saint-Hilaire (1851) listed the specimen in the MNHN as “one of the types”. Although Kuhl studied the Paris collection, he does not mention this animal, so it must have been acquired after he had completed his synopsis. Apparently, this specimen has not been found again. De Ávila-Pires was unable to locate the New York animal either, and regarded our specimen as the only syntype still in existence (erroneously giving its catalogue number as 17961). Engländer (1995: 252) too, writes: “Typ: verloren; Syntyp: Leiden”.

Other syntypes are in the MfN (ZMB 306) and in the AMNH (MS 252) (see Turni 2024: 157).

***Leontopithecuschrysopygus* (Mikan, 1823)**

*Jacchuschrysopygus* Mikan, 1823: 2, pl.

Paralectotype, RMNH.MAM.39100 (Jentink 1892: 55 *a*), male, mounted skin, skull in situ. Loc.: Ipanema, São Paulo, Brazil. Leg.: J. Natterer, [1819–1820].

Paralectotype: RMNH.MAM.39101.

Mikan (1823) had a number of specimens before him, though he does not specify how many. Jentink (1892: 55) listed RMNH.MAM.39100 and 39101 as types.

Natterer worked in the surroundings of Ipanema between 2 February 1819 and 20 March 1820, between 22 April and 15 July 1820, and again from 2 September 1821 to 7 October 1822 (von Pelzeln 1871: iii–vi; 1883: 125–126; Vanzolini 1993: 22–27).

Engelberger (2010) located five syntypes, all collected in 1819. He also specified the material received from Johann Natterer’s expeditions, based on Johann and Joseph Natterer’s notes preserved in the Vienna archives; these do not agree in every detail with von Pelzeln (1883), and not at all with Hershkovitz (1977). Regarding the animals collected in 1819, Engelberger wrote that six specimens came to Vienna with Natterer’s third shipment from Brazil, which arrived in November 1819. On the accompanying document “[♀] N3 an Temminck gegeben” is written; this note is not dated, so the date of despatch is unknown. Five of these six animals are still in Vienna; the sixth one, apparently sent to Temminck, is a female collected at Ipanema on 5 September 1819; this must be RMNH.MAM.39101. Temminck’s collection was still privately owned at the time; in 1820 it became incorporated into the newly established ‘s Rijks Museum. The Naturalis archives (inv. 241.952) contain a list of material received in exchange from the Vienna Museum in August 1821, which has an entry “Hapale chrysopygos Natt.” This might be the female from 1819, but it could also be the other specimen. That animal must have been collected in 1819 or 1820, after Natterer’s first shipment had been sent to Vienna. Hence, one cannot be absolutely sure whether one or both of our animals have indeed been seen by Mikan, but there is no proof of the contrary. Therefore, following Jentink (1892), they are both included in the type series here. According to Engelberger (2010), no other material of this species arrived in Vienna before Natterer’s twelfth shipment was received in 1836. Garbino et al. (2016: 249) selected a lectotype in the NMW (ST1577).

***Micochrysoleucos* (Wagner, 1842)**

*Hapalechrysoleucos* Wagner, 1842: 357.

Syntype, RMNH.MAM.39099 (Jentink 1892: 59 *a*), female, mounted skin, skull in situ. Loc.: [Borba], lower Madeira River, Brazil. Leg.: J. Natterer, [December 1829–June 1830].

Wagner (1842) did not specify how many animals he had before him when describing this species, although he gave only one set of measurements. Schlegel (1876: 278) recorded that the species “a été découverte par I. Natterer près de Borba sur le Rio-Madeira inférieur; il y a recueilli des individus dans les mois de Décembre, Janvier et Mai”, which was later corrected by Von Pelzeln (1883: 22) into: “Borba, December, Januar, Juni (nicht Mai, Mus. Pays-bas)”. Schlegel specified our specimen as “1. Femelle adulte, voyage de Natterer, bois des bords du confluent du Madeira et de l’Amazone”, which was copied by Jentink (1892). This seems a somewhat free interpretation of the locality as given by Wagner (1848: 467): ”Natterer entdeckte diese Art bei Borba gegen die Ausmündung des Madeira in den Amazonenstrom”. Borba, however, is considerably further upstream the river Madeira; see also Von Pelzeln (1871: xi–xiii, map; 1883b: 127) and Vanzolini (1993: 42–43).

There is no record when our specimen was received. In the archives of Naturalis (inv. 241.952) there is a letter from J. Heckel of the NMW to Schlegel, dated 5 December 1855, which contains a list headed “Zum Auswahl Schöne Säugethier-bälge aus Brasilien jedoch ohne Schädel die Frühling 1848 verbrannt sind”. In this, there is an entry “Hapala chrijsoleuca Natt”. This may refer to our skin, though it still has its skull in situ. See Engelberger (2010) and Turni (2024: 156) for the complete list of type specimens.

***Saguinusmystax* (Spix, 1823)**

*Midasmystax* von Spix, 1823: 29, pl. XXII.

Syntype, RMNH.MAM.39102 (Jentink 1892: 56 *a*), sex unknown, mounted skin, skull in situ. Loc.: [Parana-Bijuma River near São Paulo de Olivença], between Solimõens (Amazonas) and Içá Rivers, Brazil. Leg.: J.B. von Spix, [January 1820].

Von Spix (1823: 29) encountered this species near d’Olivença in the neighbourhood of a black water river named Parana-Bijuna, between the Solimõens (Amazonas) and Içá, where he was in January 1820 (von Spix and von Martius 1823–1831: 1186–1190, map).

A list of animals received from von Spix in October 1824 (Naturalis archives inv. 257.853) has an entry: “14) Midasmystax.”

Kraft (1983: 437) listed another syntype in the ZSM (ZSM 29) and Turni (2024: 157) a syntype in the MfN (ZMB 286).

***Saimiriboliviensis* I. Geoffroy Saint-Hilaire & de Blainville, 1834**

*CallithrixBoliviensis* “d’Orbigny” I. Geoffroy Saint-Hilaire & de Blainville, 1834: 89.

*Calitrix* [sic] *entomophagus* D’Orbigny, 1835: pl.4.

Syntype, RMNH.MAM.63698 (Jentink 1892: 54 *a*), adult male, mounted skin, skull in situ. Loc.: Guarayas, Bolivia. Leg.: A. d’Orbigny, [Dec. 1831–1832]. Received in Paris 1834.

*Saimiriboliviensis* is based on the specimens D’Orbigny collected in Bolivia. This publication precedes the publication of the plate of *Calitrix* [sic] *entomophagus* by D’Orbigny (1835: pl. 4).

According to Jentink (1892: 54) and Hershkovitz (1984: 191) the specimens were collected in 1834, which cannot be true as D’Orbigny embarked on his return voyage to France the 25^th^ of July 1833. D’Orbigny collected in the Santa Cruz area from October 1830 until the end of 1831 (D’Orbigny 1839–1843: 471–659), and visited the area of the Guarayos from December 1831 until January 1832. The year 1834 refers to when the MNHN received the specimens (Rode 1938: 235).

Rode (1938: 235) listed a holotype (MNHN-ZM-2005-930) and paratype (MNHN-ZM-2005-931) in the MNHN; this does not constitute a valid lectotype designation (ICZN art. 74.5). All types should be listed as syntypes.

***Sapajusapella* (Linnaeus, 1758)**

*Cebusfallax* Schlegel, 1876: 210.

Holotype by monotypy, RMNH.MAM.39091 (Jentink 1887: 42 *a*; 1892: 48 *a*), subadult male, mounted skin and skeleton. From Rotterdam Zoo, died 1 May 1875.

The taxonomic identity of this specimen is not clear. A label given by A. Remington Kellogg in 1949 says: “*Cebusapella* subsp. Probably from eastern Colombia”. Groves (2005b: 137) included this name in the *Cebusapella* species group.

*Cebuslunatus* Kuhl, 1820a: 37.

Lectotype, RMNH.MAM.39092 (Jentink 1892: 47 *d*), subadult, sex unknown, mounted skin, skull extracted but not present in collection. Ex: Heidelberg Museum.

Kuhl (1820a) described this species from an undocumented skin in the Heidelberg Museum. In the copy of Kuhl’s publication in the Naturalis library (RBR C00259:1) there is an annotation in Temminck’s handwriting saying he received the specimen. Schlegel (1876: 207) catalogued the specimen under *Cebusfrontatus* Kuhl, 1820, specifying: “3. Jeune individu, type du *Cebuslunatus* de Kuhl, échangé du Musée de Heidelberg”. As Kuhl gives no indication how many specimens he has seen, we consider this last remark by Schlegel as a lectotype designation.

The taxonomic identity of this specimen is not clear. Like Schlegel (1876), A. Remington Kellogg identified it in 1949 on the label with *C.frontatus*. Groves (2005b: 137) has included the names *lunatus* in the *Cebusapella* species group.

***Cebusvariegatus* É. Geoffroy Saint-Hilaire, 1812: 111.**

Schlegel (1876: 210) and Jentink (1892: 47) catalogued a specimen in Naturalis (RMNH.MAM.39093, juvenile female, Ex: Heidelberg Museum) as one of the types for *Cebusvariegatus* Kuhl, 1820. Kuhl (1820a: 32), however, did not describe this form as a new species, but correctly records it as “Cebusvariegatus Geoff.”, seen in the MNHN.

***Sapajusxanthosternos* Wied, 1820**

*Sapajusxanthosternos* Wied, 1820a: 368.

Lectotype, RMNH.MAM.17689 (Jentink 1892: 47 *a*), female, relaxed mount, skull in situ. Loc.: [Bôca d’Obú], Belmonte [Jequitinhonha] River, Brazil. Leg.: M. zu Wied, [18 August 1816]. Received 1823.

Wied travelled on the Rio Belmonte from 17 August to 28 September 1816 (Joost 1987: 12; Wied in Joost 1987: 125–155; Vanzolini and Myers 2015). Based on Wied (1820a: 327), De Ávila-Pires (1965: 11) writes that the type locality is “not far from Bôca d’Obu”, the mouth of a small tributary of the lower Belmonte River. Wied visited this place on 18 August (Wied in Joost 1987: 126; Wied 1826: 95).

A label given by A. Remington Kellogg in 1949 reads: “One of original Co-types - Possibly only one extant. RK”. Apparently, no other syntypes were located by De Ávila-Pires (1965: 11), who therefore designated RMNH.MAM.17689 the lectotype of the species.

De Ávila-Pires (1965) erroneously attributed the name to Kuhl (1820a: 35, wrongly given as p. 51) who, however, records the species as “*Cebusxantosternos* [sic] Max.” Garbino and Costa (2015: 22) demonstrated that Wied (1820a) was available by 29 March 1820, while Kuhl (1820a) was not published before April 1820.

#### ﻿﻿Pitheciidae Mivart, 1865

***Cacajaocalvusrubicundus* (I. Geoffroy Saint-Hilaire & Deville, 1848)**

*Brachyurusrubicundus* I. Geoffroy Saint-Hilaire & Deville, 1848: 498.

Syntype, RMNH.MAM.63658 (Jentink 1887: 45 *a*; 1892: 51 *a*), adult male, mounted skin and skull. Loc.: “Rive meridionale de l’Iça” Brasil?. Leg.: F. Castelnau, E. Deville, 1847.

It is unclear why Rode (1938: 233) listed a specimen in the MNHN (MNHN-ZM-2005-898) as the holotype; the type series clearly consists of several specimens. Therefore this is not a valid lectotype designation according to ICZN art. 74.5. All types should be listed as syntypes.

***Cacajaomelanocephalus* (Humboldt, 1812)**

*Brachyurus Ouakary* von Spix, 1823: 12, pl. VIII.

Syntype, RMNH.MAM.39085 (Jentink 1887: 44 *a*; 1892: 50 *a*), male, mounted skin and skull. Loc.: Between Solimões [Amazonas] and Içá Rivers, Brazil. Leg.: J.B. von Spix, [January 1820].

Von Spix travelled in this area in January 1820 (von Spix and von Martius 1823–1831: 1186–1190, map); see under *Saguinusmystax* and *S.nigricollis* above. A list of animals received from von Spix in October 1824 has an entry: “1) Brachyurus dunkary” [sic] (Naturalis archives inv. 257.853).

Kraft (1983: 434) listed another syntype in the ZSM. Barnett (2005: 1) referred to this specimen as the holotype, which is not a valid lectotype designation according to ICZN art. 74.7.1.

***Callicebusmelanochir* (Wied, 1820)**

*Callithrixmelanochir* Wied, 1820a: 256.

*Callithrixincanescens* “Lichtenstein” Kuhl, 1820a: 40 (in synonymy).

Lectotype, RMNH.MAM.17690 (Jentink 1887: 45 *a*; 1892: 51 *a*), sex unknown, mounted skin and skull. Loc.: [Morro d’Arara] Belmonte [Jequitinhonha] River, Brazil. Leg.: Maximilian, Prinz zu Wied, [August–September 1816].

Paralectotype, RMNH.MAM.39090.

Wied travelled on the Rio Belmonte from 17 August to 28 September 1816 (Joost 1987: 12, 125–155); according to Wied he stayed at Morro d’Arara or Fazenda Arara.

De Ávila-Pires (1965: 10) erroneously attributed the name to Kuhl (1820a: 40) who, however, records the species as “*Callithrixmelanochir* Pr. Max.” [= Wied]. On p. 41 he mentions the two Naturalis specimens: “In Museo Temminkiano [sic] 2”. Elliot (1913a: 245) also overlooked the brief description by Wied (1820a). De Ávila-Pires misquoted I. Geoffroy Saint-Hilaire (1851: 40) by stating that he “listed the type (adult male) as being in the collection of the Museum National d’Histoire Naturelle, which is probably the same specimen described as the type by Elliot (1913a)”. The same applies to De Ávila-Pires’s citation of Rode (1938: 235), who correctly speaks of a paratype in the MNHN, and of Elliot (1913a: 245), who also regards the Paris male as a “co-type”. Apparently, this specimen could not be found at that time. Finally, De Ávila-Pires confounded the two Naturalis skins, recording RMNH.MAM.17690 as specimen *c* from Rio Belmonte and marked as “type”, the other one (RMNH.MAM.39090) as *a*, without locality and type notation; however, both are labelled Rio Belmonte and both are listed as types by Jentink (1887; 1892). Engländer (1995: 252), strangely, writes: “Typ oder Syntyp: Leiden”. Basing himself on Wied (1820a: 224), De Ávila-Pires restricted the type locality to the lower Belmonte River.

Hershkovitz (1990: 70) correctly listed our two specimens and designated RMNH.AVES.17690 the lectotype. According to him the specimen in the MNHN (MNHN-ZM-2007-1504) has been located.

Kuhl (1820a) published Lichtenstein’s manuscript name *incanescens* in the synonymy of *melanochir.* Therefore this is not an available name.

***Chiropoteschiropotes* (Humbold, 1812)**

*Brachyurus Israelita* von Spix, 1823: 11.

Syntype, RMNH.MAM.53007 (Jentink 1887: 44 *f*), skull, sex unknown. Leg.: J.B. von Spix.

See Schlegel (1867: 223) for a discussion on the type locality for this species. Another syntype in the ZSM was listed by Kraft (1983: 434).

***Pitheciachrysocephala* I. Geoffroy Saint-Hilaire, 1850**

*Pitheciachrysocephala* I. Geoffroy Saint-Hilaire, 1850: 875.

Neotype, RMNH.MAM.63700 (formerly 1845), adult male, mounted skin and skeleton. Loc.: Manacapurú, Amazonas, Brazil. Leg.: 15 August 1924. Ex: Schlüter and Mass, 25 May 1930.

Marsh (2014) designated the neotype since the original types are presumed missing.

***Pitheciainusta* (Spix, 1824)**

*Pitheciainusta* Spix, 1823: 15, pl. X.

Paralectotype, RMNH.MAM.39096 (Jentink 1892: 49 *a*), male, mounted skin, skull in situ. Loc.: Tabatinga, Brazil. Leg.: J.B. von Spix, [early January 1820]. Ex: J.B. von Spix, October 1824.

Von Spix did not mention the number of specimens he used for his description. RMNH.MAM.39096 agrees with the entry in the list of animals received from von Spix in October 1824 (Naturalis archives inv. 257.853). Jentink (1892) erroneously catalogued the animal as “un des types du *Pitheciahirsuta* Spix”.

According to Hershkovitz (1979: 15) the specimen figured by von Spix is the type. Later, Hershkovitz (1987: 423) designated the same specimen as lectotype. Kraft (1983: 433) stated no type specimen is present in the ZSM, which was confirmed by him in Marsh (2014: 49).

Von Spix arrived on 9 January 1820 at Tabatinga, situated on Solimões (Amazonas) River at the Peruvian border, and collected there during at least four days (von Spix and von Martius 1823–1831: 1187–1189, map); see under *Callicebuscupreus*.

***Pitheciapithecia* (Linnaeus, 1766)**

*Pitheciaochrocephala* Kuhl, 1820a: 44.

Syntype, RMNH.MAM.39097 (Jentink 1892: 49 d), subadult male, mounted skin, skull in situ. Loc.: “Suriname” [error for Cayenne = French Guiana]. Ex: Cabinet Temminck.

The pedestal of the type specimen bears the locality “Surinam”, written in an unknown hand; the label also gives this locality, and so did Schlegel (1876: 219, nr 4). The colouration of this animal agrees very well with the description by Kuhl (1820a), although Schlegel, strangely, wrote that the description does not in all respects match this specimen, attributing it to a mix up of Kuhl’s notes. Kuhl gave the provenance of his animal as “In Cajanna”, which is Cayenne or French Guiana. There can be no confusion on Kuhl’s side, as on the same page he gave the locality of his *Pitheciarufibarbata* (see below) as “In Surinama”. It seems likely that the provenance of two specimens has since been mixed up. Schlegel’s catalogue nr 3 (Jentink’s catalogue *c*), a very black adult male, bears the locality “Cayenne”, on the pedestal (in Schlegel’s handwriting) and on the label. Its colouration agrees in no respect with Kuhl’s description. Since Kuhl is the more original source, it seems best to assume that RMNH.MAM.39097 is indeed from French Guiana.

***Pitheciarufibarbata* Kuhl, 1820a: 44.**

Lectotype, RMNH.MAM.39098 (Jentink 1892: 49 *l*), juvenile female, mounted skin, skull in situ. Loc.: Suriname. Ex: Cabinet Temminck.

Jentink (1892: 49) designated the lectotype.

***Plecturocebuscupreus* (Spix, 1823)**

*Callithrixcuprea* von Spix, 1823: 23, pl. XVII.

Paralectotype, RMNH.MAM.39086 (Jentink 1892: 52 *a*), adult female, mounted skin, skull in situ. Loc.: [Tabatinga], Solimões River near the Peruvian border, Brazil. Leg.: J.B. von Spix, [early January 1820].

Schlegel (1876: 237) gave the locality as “sur les bords du Solimoëns au Pérou”. Von Spix was in this area in early January 1820; he went up the river as far as Tabatinga on the Brazilian/Peruvian border, where he arrived on 9 January, but did not proceed into Peru; he collected there during at least four days (von Spix and von Martius 1823–1831: 1187–1189, map). Hershkovitz (1990: 61) restricted the type locality to “Rio Solimões, opposite Tabatinga’’ as the species does not occur on the north bank of the river.

A list of specimens received from von Spix in October 1824 (Naturalis archives inv. 257.853) has the entries: “9) Callitrix cuprea ♀” and “16) Calitrix [sic] cuprea ♂”. Only the female is still present in our collection.

Hill (1960: 122) designated a female (ZSM 10) in München as the lectotype (see Kraft 1983: 432). There are also three paralectotypes in the ZSM; another is located in the MfN (Turni 2024: 159).

***Plecturocebusdiscolor* (I. Geoffroy & Deville, 1848)**

*Callithrixdiscolor* I. Geoffroy & Deville, 1848: 498.

According to Jentink (1892: 52) one of the catalogue entries for *Callithrixornata* Gray is a type for “*Callithrixdiscolor* Verreaux” (RMNH.MAM.39087, male from Nouvelle Granade received from Verreaux in 1873). Jentink’s 1887 catalogue of skeletal material does not mention this name and supposed type status, nor does Schlegel (1876: 238–239).

The name *Callithrixdiscolor* Verreaux cannot be found in the literature. This name was published by I. Geoffroy Saint-Hilaire and Deville in 1848, from the type locality Tabatinga, Brazil. The name does not appear in the lists of material acquired from Verreaux (Naturalis archives inv. 56.10). Obviously RMNH.MAM.39087 cannot be a type of *Callithrixdiscolor* I. Geoffroy Saint-Hilaire & Deville, 1848, of which the holotype is in the MNHN (Rode 1938: 233).

***Plecturocebusdonacophilus* (d’Orbigny, 1835)**

*Callithrixdonacophilus* d’Orbigny, 1835: pl. 5.

Syntype, RMNH.MAM.39088 (Jentink 1892: 53 *c*), subadult female, mounted skin, skull in situ. Loc.: Bolivia. Leg.: A. d’Orbigny, [January–September 1832]. Acquired 1834.

Syntype, RMNH.MAM.39089 (Jentink 1892: 53 d), juvenile, sex unknown, mounted skin, skull in situ. Loc.: Bolivia. Leg.: A. d’Orbigny, [January–September 1832].

This name was first validly published by D’Orbigny (1835) in the caption of plate 5, not 1836 as in most publications (Groves 2005b: 142; Rylands and Mittermeier 2020: 196). For the dating of d’Órbigny’s work see Sherborn and Griffin 1934: 130. The description followed much later (D’Orbigny 1847: 10).

Rode (1938: 234) listed one of the MNHN specimens as holotype. This was followed by Hershkovitz (1990: 49), who reported the specimen missing. In the online database of the MNHN a holotype is listed (MNHN-ZM-2007-1505), alongside three paratypes (MNHN-ZM-2007-1506, 1525, 1527). It is unclear why Rode (1938) listed this particular specimen as holotype; clearly the type series consists of several specimens. Therefore this is not a valid lectotype designation according to ICZN art. 74.5.

According to D’Orbigny (1844: 81) this species was found in Moxos district, Bolivia, where he stayed several times from January to September 1832.

#### ﻿﻿Atelidae Gray, 1825

***Alouattabelzebul* (Linnaeus, 1766)**

*Mycetesrufimanus* Kuhl, 1820a: 31.

Syntype, RMNH.MAM.39095 (Jentink 1892: 37 *a*), female, mounted skin, skull in situ. Ex: Bullock’s Museum, 7 May 1819.

Bullock’s collection was auctioned in London during a 26-days sale in April–June 1819. Temminck and his assistant Kuhl were present, and Temminck’s copy of the catalogue (Bullock 1819) is preserved in the archives of Naturalis (inv. 49.44). In this, Temminck has marked the specimens that he bought with a “T” and noted the price he paid. On p. 38 of the catalogue, there is an entry: “65 Preacher Monkey, *S*[*imia*] *Beelzebub*”. Temminck has marked this item as very good, and bought it for £ 3. Schlegel (1876: 151) and Jentink (1892: 37) both listed this specimen as “one of the types”; however it is unclear from the original description how many specimens were available.

***Alouattacaraya* (Humboldt, 1812)**

*Mycetesbarbatus* von Spix, 1823: 46.

Syntype, RMNH.MAM.64136 (Jentink 1892: 37: *a*), adult male, mounted skin, skull extracted but not in collection. Loc.: interior of Bahia, Brazil. Leg.: J.B. von Spix, [1817–1820].

Syntype, RMNH.MAM.64137 (Jentink 1892: 37: *b*), adult female, mounted skin, skull extracted but not in collection. Loc.: interior of Minas-Geraës, Brazil. Leg.: J.B. von Spix, [1817–1820].

Another syntype (no. 9) is in the ZSM (Kraft 1983: 434).

***Atelesbelzebuth* É. Geoffroy Saint-Hilaire, 1806**

*S[imia].chuva* Humboldt, 1812: 362.

*Atelesproblema* “Schlegel” Jentink, 1887: 42 (in synonymy).

Von Humboldt introduced this species initially only by its vernacular name “le chuva de Bracamorros”, without providing a description (von Humboldt 1805: 8, cited from second edition, 1812). Later (von Humboldt 1812: 340), after having read the description by É. Geoffroy Saint-Hilaire, Humboldt adopted the name *Atelesmarginatus* and (p. 341) described the species under É. Geoffroy Saint-Hilaire’s name. However in a footnote listing the new names introduced by Bonpland and himself, von Humboldt (1812: 362) published the name *S[imia]chuva* with a reference to p. 354, where “Chuva” is listed as a junior synonym for *Simiamarginata*. These pages were all published in the same issue (Sherborn 1899). Although this is probably a lapsus by von Humboldt, the name *Simiachuva* was validly published. Schlegel (1876: 174–175) treated *Atelesmarginatus* and *A.chuva* as distinct species and this was seen by most authors (Kellogg and Goldman 1944; Groves 2005b) as the valid publication of *Ateleschuva*. However in accordance with ICZN art. 11.6.1 this name should be attributed to von Humboldt (1812) and the type series consists of the specimens mentioned in that publication (ICZN art. 74.4.3). Therefore, RMNH.MAM.31771 and 59661 are not types. Jentink (1887, 1892) did not list these specimens as types since he correctly attributed the name *Ateleschuva* to von Humboldt.

On the pedestal of RMNH.MAM.59661 is written “*Atelesproblema* Schl. n. sp.” and this name is also mentioned in Jentink’s Catalogue (1892: 42). We have not been able to find any valid publication of this name, therefore this name is not available.

***Atelesgeoffroyi* Kuhl, 1820**

*Atelespan* Schlegel, 1876: 180.

Syntype, RMNH.MAM.39082 (Jentink 1887: 38 *c*; 1892: 43 *a*), male, mounted skin and skull. Loc.: Cobán, Guatemala. Leg.: 1874. Ex: G. Schneider, 1875.

Syntype, RMNH.MAM.39083 (Jentink 1887: 38 *d*; 1892: 43 *b*), female, mounted skin and skull. Loc.: Cobán, Guatemala. Leg.: 1874. Ex: G. Schneider, 1875.

Syntype, RMNH.MAM.39084 (Jentink 1887: 38 *e*; 1892: 43 *c*), female, mounted skin and skull. Loc.: Cobán, Guatemala. Leg.: 1874. Ex: G. Schneider, 1875.

These specimens were obtained from the dealer G. Schneider in Basel (curator of the museum in Basel at that time), who sent them to Leiden in April 1875. A letter from Schneider to Schlegel dating 8 April 1875 (Naturalis archives inv. 257.240) has an entry: “3 Ateles variegatus 2♂ 1♀? Vera Paz” (see Schlegel 1876: 180). They had been collected in 1874 near Cobán, Vera Paz, Guatemala, by an unnamed German collector. In the same year also some birds were acquired through Schneider from the same location collected by F. Sarg. It is likely that these mammals were also from this source.

***Brachyteleshypoxanthus* Wied, 1820**

*Ateleshypoxanthus* Wied, 1820a: 91.

Lectotype, RMNH.MAM.17688 (Jentink 1892: 44 a), male, mounted skin, skull in situ. Loc.: [As Barreiras, Rio Belmonte] Bahia, Brazil. Leg.: M. zu Wied, [August 1816].

De Ávila-Pires (1965: 9) designated the lectotype and restricted the type locality to “As Barreiras”, based on Wied (1820a: 267). Date and locality are based on Moraes (2009: 29).

De Ávila-Pires (1965) and Rylands et al. (2020: 218) attributed this name to Kuhl (1820a); however, Garbino and Costa (2015: 22) demonstrated that Wied (1820a) was available by 29 March 1820, while Kuhl (1820a) was not published before April 1820.

***Lagothrixlagotricha* (Humboldt, 1812)**

*Gastrimargusinfumatus* von Spix, 1823: 41, pl. XXIX.

Syntype, RMNH.MAM.39094 (Jentink 1887: 36 *a*; 1892: 40 *a*), male, mounted skin and skull. Loc.: Lower Içá River, Brazil. Leg.: J.B. von Spix, [last week of January 1820].

Von Spix travelled on the lower Içá River during the last week of January 1820 (von Spix and von Martius 1823–1831: 1190, map).

The list of animals received from von Spix in October 1824 (Naturalis archives inv. 257.853) has an entry: “11) *Gastrimarchusmustelinus*”, which must be the present specimen. This name refers to the French and German name von Spix used for this species (von Spix 1823: 41; 1823: pl. XXIX): martin-coloured woolly monkey.

Kraft (1983: 435) listed three syntypes in the ZSM.

#### ﻿﻿Cercopithecidae Gray, 1821

***Cercocebuslunulatus* (Temminck, 1853)**

*Cercopithecuslunulatus* Temminck, 1853: 37.

Syntype, RMNH.MAM.39104 (Jentink 1892: 25 *a*), female, mounted skin, skull in situ. Loc.: [Butri (Ankobra) River or Dabo Krom], Gold Coast [Ghana]. Leg.: H.S. Pel, [February–March 1843 or 1849/50].

Syntype, RMNH.MAM.39105 (Jentink 1887: 22 *a*; 1892: 25 *b*), female, mounted skin and skull. Loc.: [Butri River or Dabo Krom], Ghana. Leg.: H.S. Pel, [February–March 1843 or 1849/50].

Syntype, RMNH.MAM.39106 (Jentink 1892: 25 *c*), female, mounted skin, skull in situ. Loc.: [Elmina], Ghana. Leg.: H.S. Pel, January [1841].

Syntype, RMNH.MAM.39107 (Jentink 1892: 25 *d*), subadult male, mounted skin, skull in situ. Loc.: Dabocrom (Dabo Krom), Ghana. Leg.: H.S. Pel, [February–March 1843].

Schlegel (1876: 95–96) and Jentink (1887: 22; 1892: 25) did not mention these specimens as types, probably because they synonymised *Cercopithecuslunulatus* Temminck, 1853 with *Cercocebusaethiops* (= *Cercocebusatys*).

Temminck’s (1853) account of the mammals of the Guinea coast is based on the collections that Pel brought together during his first period in the Gold Coast (p. viii). All specimens collected by Pel between 1841 and 1850 had arrived in Leiden when Temminck published his description, and therefore belong in the type series.

Temminck (p. 38) gave Butri River as the locality. However, only RMNH.MAM.39107 bears an precise locality, and only RMNH.MAM.39106 is dated. It is not always possible to reconstruct the precise localities and dates of Pel’s material. In his correspondence and shipping inventories (Naturalis archives inv. 247.112–114) this species is mentioned three times. The first shipment, dispatched in September 1841, gives a “*Cercopithecuslunulatus*? Elmina Bosch. Jan. 1841. Iris bruin Mas” (Elmina Forest. January 1841. Iris brown Male). Pel already used the name *C.lunulatus* here, perhaps coined by himself. However, the label of RMNH.MAM.39106 is dated January 1842, when Pel also was at Elmina (Holthuis 1968: 19). Since there is no record of a *Cercopithecus* from Elmina in Pel’s second shipment inventory sent in March 1843, we have to assume that 1842 is an error for 1841. To make things worse, the inscription on the pedestal, of later date, says January 1849, which is obviously a mistake, as Pel was not at Elmina at that time (Holthuis 1968: 19). In a letter sent from Butri dated 10 March 1849, Pel wrote that he was now sending three specimens of *Cercopithecuslunulatus*; these appear in his shipment inventory of 16 March: “3 e[xx.] Dabocrom”. One more animal is mentioned in the inventory of 7 May 1850, but without locality or date. So at least three specimens were collected at Dabocrom (Dabo Krom), which is situated between Butri town (the present Princes’ Town) and Sekondi (Holthuis 1968: 9, 22–23); this is indeed close to Butri (Ankobra) River, a small tidal stream. Dabo Krom was Pel’s favourite collecting locality and he was here on various occasions during 1843–1850. The three animals sent in March 1843 correspond with his first visits to this place in February and March of that year (Holthuis 1968: 22). The pedestal of RMNH.MAM.39107 bears the inscription “Dabocrom” in Temminck’s handwriting, so this must be one of those three. In 1849 and 1850, Pel was intermittently at Butri and Dabo Krom (Holthuis 1968: 22). Although one may suppose that the remaining two specimens also would have been collected at Dabo Krom in 1843, it cannot be excluded that one of those may be from Butri.

***Cercopithecusmitismitis* Wolf, 1822**

*CercopithecusDilophos* Ogilby, 1838c: 343.

Syntype, RMNH.MAM.39119 (Jentink 1892: 21 *a*), male, mounted skin, skull in situ. Loc.: Coastal Angola.

Syntype, RMNH.MAM.39120 (Jentink 1892: 21 *b*), female, mounted skin with false tail, skull extracted but not in collection. Loc.: Angola.

Ogilby (1838c) based his description of *Cercopithecusdilophos* on two specimens in Naturalis and a specimen described by F. Cuvier from the Paris menagerie. Schlegel (1876: 83) and Jentink (1892: 21) apparently overlooked Ogilby’s publication and have not catalogued these skins as types. A skeleton of the same provenance (RMNH.MAM.63701, Jentink, 1887: 19 *a*) is not mentioned by Ogilby and therefore does not belong in the type series. The possibility that this is the skeleton of RMNH.MAM.39120 cannot be confirmed.

The pedestal of RMNH.MAM.39119 bears an inscription in Temminck’s handwriting, identifying the animal as ”fem ad” and “femelle”, and giving its locality as “Cote d’Angola”. Although its genitals are lost, it is clearly a male. The tail of RMNH.MAM.39120 has been replaced during preparation.

*CercopithecusTemminckii* Ogilby, 1838c: 343.

According to Ogilby there was a single specimen in Naturalis, acquired in Amsterdam in 1824 from Guinea, on which he based this new name. Both Schlegel (1876) and Jentink (1887) overlooked this name and did not list any specimen in their catalogue with the data mentioned in the description. This specimen seems to be lost, since it wasn’t recently found in the collection.

According to Schwarz (1928: 96) Ogilby’s name is based on *Colobustemminckii* Kuhl. However the description by Ogilby differed in many aspects with this species (e.g., black arms and legs). Based on comparison with other species we tentatively identify this specimen as *Cercopithecusmitismitis*.

***Cercopithecusmitiserythrarchus* Peters, 1852**

*Cercopithecuserythrarchus* Peters, 1852: 1, pl.I.

Paralectotype, RMNH.MAM.39121 (Jentink 1887: 17 *a*; 1892: 19 *a*), male, subadult, mounted skin and skull. Loc.: Mozambique. Leg.: W.C.H. Peters, [1843–1847]. Ex: MfN, 25 Mai 1851.

Peters (1852) did not state how many specimens he had before him. He gave the measurements of one animal only, but mentioned two localities: “Inhambane, Quellimane, a 17° ad 24° Lat. Austr.”.

The shipment from Peters was received on 25 May 1851 and contained a skin and skull of *Cercopithecuserythrarchus* (Naturalis archives inv. 247.169). Schwarz (1927: 152) mentioned a specimen in the MfN (No. 16059) as the type of *C.erythrarchus*, Peters, 1850, thereby designating it the lectotype (see Turni 2024: 163).

Peters collected in Mozambique during 1843–1847 (Peters 1852: viii–ix).

***Cercopithecusmitislabiatus* I. Geoffroy Saint-Hilaire, 1842**

*Cercopithecussamango* Sundevall, 1844: 160.

*Cercopithecuschimango* Temminck, 1853: 32 (lapsus for *samango*).

Syntype, RMNH.MAM.39123 (Jentink 1887: 18 *a*; 1892: 20 *a*), female, mounted skin, skull extracted but missing, only pedestal found. Loc.: [Inland of Durban], South Africa. Leg.: J.A. Wahlberg, [6–7 June 1841]. Ex: NRM, C.J. Sundevall.

From the description by Sundevall (1844: 160–161) it is clear that he had several specimens before him; he wrote that Wahlberg had collected this species inland from Port Natal (Durban) in May or June 1841. Wahlberg recorded in his diary for 6–7 June 1841 the shooting of 6 “Samangos” around the kraal Umgeni (Hummel 1994: 49).

*Cercopithecusneglectus* Schlegel, 1876: 70.

Schlegel (1876) based his description of this species on the description by Gray (1871a: 22) of *Cercopithecusleucocampyx* from the White Nile, Sudan. The online database of the NHM lists a holotype for *Cercopithecusneglectus* (NHMUK 1860.4.20.7, (https://data.nhm.ac.uk/object/0737d1e8-1903-4683-8d4f-994ce83602f8/1738195200000) although from Gray (1871a) it is not clear how many specimens were available.

***Cercopithecusnictitansstampflii* Jentink, 1888**

*Cercopithecusstampflii* Jentink, 1888a: 10.

Holotype, RMNH.MAM.39125 (Jentink 1892: 24 *a*), male, mounted skin and skeleton. Loc.: Pessy Country, Liberia. Leg.: J. Büttikofer and F.X. Stampfli (142), 24 March 1887.

Paratype: RMNH.MAM.58929.

In 1887 Büttikofer and Stampfli worked in the coastal districts of Liberia and did not reach Pessy Country (Büttikofer 1888: 59–62, pl. 5). A single animal of this species was obtained from their hunters (Büttikofer 1890b: 358). Jentink (1888a) mentioned in the beginning of his description that he had “one specimen”, namely the one collected by Büttikofer and Stampfli and later (p. 12) referred to this specimen as the type. However, he also described in detail a second specimen as also belonging to this new species (RMNH.MAM.58929).

***Cercopithecusnictitans* (Linnaeus, 1766)**

*Cercopithecussignatus* “Schlegel” Jentink, 1886a: 55.

Holotype by monotypy, RMNH.MAM.39124 (Jentink 1887: 20 *a*; 1892: 23 *a*), male, mounted skin and skeleton. Loc.: ?Banana, Democratic Republic of Congo. Ex: Rotterdam Zoo, died 6 January 1877, received 1 February 1887.

Oates (1985: 41) suggested that this specimen represents a hybrid between *C.nictitans* and a member of the *Cercopithecuscephus* (Linnaeus, 1758) species group.

***Cercopithecuspetauristabuettikoferi* Jentink, 1886**

*Cercopithecus büttikoferi* Jentink, 1886a: 56.

Syntype, RMNH.MAM.39109 (Jentink 1887: 20 *b*), male, skull. Loc.: Bavia on St. Paul River, Liberia. Leg.: J. Büttikofer and C.F. Sala, 9 February 1880.

Syntype, RMNH.MAM.39110 (Jentink 1887: 20 *d*), sex unknown, juvenile, skull. Loc.: Soforeh-Place on St. Paul River, Liberia. Leg.: J. Büttikofer and C.F. Sala, [April–October] 1880.

Syntype, RMNH.MAM.39111 (Jentink 1892: 22 *a*), male, mounted skin, skull in situ. Loc.: Soforeh-Place, Liberia. Leg.: J. Büttikofer and C.F. Sala, 1 August 1880.

Syntype, RMNH.MAM.39112 (Jentink 1892: 22 *b*), male, mounted skin, skull in situ. Loc.: Soforeh-Place, Liberia. Leg.: J. Büttikofer and C.F. Sala, 31 July 1880.

Syntype, RMNH.MAM.39113 (Jentink 1892: 22 *c*), male, mounted skin, skull extracted but not in collection. Loc.: Soforeh-Place, Liberia. Leg.: J. Büttikofer and C.F. Sala, 11 August 1880.

Syntype, RMNH.MAM.39114 (Jentink 1892: 23 *d*), juvenile female, mounted skin, skull in situ. Loc.: Soforeh-Place, Liberia. Leg.: J. Büttikofer and C.F. Sala, 12 September 1880.

Syntype, RMNH.MAM.39115 (Jentink 1887: 20 *c*; 1892: 23 *e*), subadult female, skin and skull. Loc.: Bavia, Liberia. Leg.: J. Büttikofer and C.F. Sala, 15 March 1880.

Syntype, RMNH.MAM.39116 (Jentink 1887: 20 *a*; 1892: 23 *f*), male, mounted skin and skeleton. Loc.: Bendoo on Fisherman Lake (Lake Pisa), Liberia. Leg.: J. Büttikofer and C.F. Sala, 30 November 1880.

Syntype, RMNH.MAM.39117 (Jentink 1892: 23 *h*), juvenile male, mounted skin, skull in situ. Buluma near Fisherman Lake [Lake Pisa], Liberia, 12 December 1880. Leg.: J. Büttikofer and C.F. Sala.

Syntype, RMNH.MAM.39118 (Jentink 1892: 23 *g*), juvenile female, mounted skin, skull in situ. Loc.: Bendoo, Liberia. Leg.: J. Büttikofer and C.F. Sala, 24 November 1880.

According to Jentink (1886a) he had a series of eight specimens; however, Naturalis holds ten specimens received from Büttikofer and Sala. We consider all these to form part of the type series. RMNH.MAM.39111 is erroneously labelled 1878, but correctly catalogued by Jentink (1892: 22) as 1880; in 1878 Büttikofer and Sala had not yet arrived in Liberia. They stayed at Soforeh-Place from 1 April to 15 October 1880 (Büttikofer 1885: 143). RMNH.MAM.39116 is wrongly dated 1881 by Jentink (1887: 20; 1892: 23), which is the year when the specimens arrived in Naturalis. See for the localities and collecting periods Schlegel (1881) and Büttikofer (1884: 96–119, 125, map; 1885: 142–147, pl. 7; 1890a: map).

***Cercopithecuspogonias* Bennett, 1833**

*Cercopithecuspetronellae* Büttikofer, 1911: 1.

Syntype, RMNH.MAM.244, juvenile male, skin and skeleton. Loc.: Upper Congo [Haut-Congo], Democratic Republic of Congo, imported December 1910 and died 23 June 1911. Ex: Rotterdam Zoo (J. Büttikofer).

According to Büttikofer (1911), then director of the Rotterdam Zoo, he had seen three specimens of this new species: the Naturalis specimen, a living animal in the Antwerp Zoo, now in the RMCA (RMCA 3428; Wendelen in litt. 2014), and a third in possession of the dealer Ruhe (Alfeld). The animal was said to be imported from the “Upper Congo”. In his description, its sex is erroneously recorded as female.

***Colobusguereza* Rüppell, 1835**

*ColobusGuereza* Rüppell, 1835: 1, Taf. 1.

*ColobusRüppellii* Gray, 1870: 19 (nomen novum).

Paralectotype for *C.guereza*, syntype for *C.rueppellii*, RMNH.MAM.39127 (Jentink 1887: 7 *b*; 1892: 6 *b*), female, mounted skin and incomplete skull. Loc.: Kulla district, Ethiopia. Leg.: E. Rüppell, [October 1832–January 1833].

Schlegel (1876: 26) mentioned the presence of three specimens from Ethiopia, of which he only attributed the female (nr. 2) to Rüppell, which is in agreement with the labels. However, the name Rüppell has been added on the pedestals of the skin and skull of the male from Shoa (nr 1), but not in Schlegel’s handwriting. In his osteological catalogue, Jentink (1887: 6 *a*) did not give Rüppell as the collector of this male (RMNH.MAM.39126), but in his skin catalogue (1892: 6, cat *a*) he does; the latter must be erroneous.

Rüppell (1835: 1) collected these specimens in Gojam und Kulla. He departed from Gondar for the Kulla area further north in October 1832 and returned to Gondar on 18 January 1833 (Mertens 1949: 78–79, pl. 15). Rüppell never proceeded as far south as Shoa, so an animal from that area cannot have been collected by him. The confusion may have arisen from the correspondence between Rüppell and Schlegel. In a list of specimens sent to Naturalis in exchange, enclosed with his letter of 15 October 1837, Rüppell mentioned “2 *Colobusguereza*, (einer zurück)” (Naturalis archives inv. 255.576). This clearly means that only one of these animals was retained by Naturalis.

Gray (1871a: 19) listed Rüppell’s *Colobusguereza* under the new name *Guerezarueppellii*, and referred to the description by Rüppell (1835), thus including all original specimens in the type series of this new name.

Jentink (1887: 7; 1892: 6) did not mention these specimens as types, although the one from Kulla clearly belongs in the type series. Mertens (1925: 37) designated a specimen in the SMF (Nr 1856, from Damot, Gojam) the lectotype of the species.

***Colobusvellerosus* (I. Geoffroy, 1834)**

*ColobusLeucomeros* Ogilby, 1838c: 288.

Syntype, RMNH.MAM.63707 (Jentink 1892: 7 f), juvenile, sex unknown, alcohol. Loc.: Guinea.

Ogilby (1838c) based his description on a skin in the Zoological Society, a specimen in the MNHN, and a juvenile in Naturalis from Guinea. Schlegel (1876: 26 cat. 5) and Jentink (1892: 7 cat. f) list two juveniles, one collected by Pel, which cannot be the specimen to which Ogilby referred, since Pel only arrived in Ghana in 1840. The other juvenile, stored in alcohol, must be the specimen Ogilby examined.

Napier (1985: 6) listed a specimen in the NHM (NHMUK 1855.12.24.405) as holotype, which does not qualify as a valid lectotype designation (ICZN art. 74.5).

***Lophocebusaterrimus* (Oudemans, 1890)**

*Cercopithecusaterrimus* Oudemans, 1890: 267.

Holotype by monotypy, RMNH.MAM.39108 (Jentink 1892: 26 *f*), subadult female, mounted skin and skeleton. Loc.: Stanley [Boyoma] Falls, Democratic Republic of Congo, “25° 10’ O. L. Greenw. 0° Br”. Leg.: A. Greshoff, imported June 1890, died 8 October 1890. Ex: The Hague Zoo.

According to Oudemans (1890) this animal was bought at the Stanley Falls, but the actual collecting locality is unknown.

***Macacafascicularisfascicularis* (Raffles, 1821)**

*Macacairussubmordax* Sody, 1949b: 133.

Holotype, RMNH.MAM.34332, male, skin and skull. Loc.: Mt. Agung, Bali, Indonesia. Leg.: [A. Samat], 24 July 1930. Ex: H.J.V. Sody (E.85).

Paratypes: RMNH.MAM.34329–34331, 34333, 34334.

Sody (1949b) examined ten specimens, six from his own collection. The latter are all in Naturalis.

***Macacafasciculariskarimondjawae* Sody, 1949**

*Macacairuskarimondjawae* Sody, 1949b: 132.

Holotype, RMNH.MAM.10608, male, skin and skull. Loc.: Karimunjawa Islands, Indonesia. Leg.: W. Romswinckel, 28 November 1930. Ex: MZB (2719), 27 June 1950.

Sody (1949b) examined nine specimens for his description; only the holotype is in Naturalis. The collecting date was erroneously given by Sody (1949b) as “28.VI.1930”.

***Macacafuscata* (Blyth, 1875)**

*Inuusfuscatus* Gray, 1871a: 32 (in synonymy).

*Inuusfuscatus* Blyth, 1875: 6.

Syntype, RMNH.MAM.39404 (Jentink 1887: 27 *f*), female, skull. Loc.: Japan. Leg.: P.F. von Siebold, [1823–1829].

Syntype, RMNH.MAM.39405 (Jentink 1887: 27 *g*), female, skull. Loc.: Japan. Leg.: P.F. von Siebold, [1823–1829].

Syntype, RMNH.MAM.39406 (Jentink 1887: 27 *h*), sex unknown, juvenile skull. Loc.: Japan. Leg.: P.F. von Siebold, [1823–1829]. Figured in Temminck (1842: pl. II figs 5, 6).

Syntype, RMNH.MAM.45486 (formerly 39397) (Jentink 1892: 31 *c*), female, mounted skin, skull in situ. Loc.: Japan. Leg.: P.F. von Siebold, [1823–1829].

Syntype, RMNH.MAM.54921 (formerly 39395) (Jentink 1887: 27 *d*; 1892: 31 *a*), very old male, mounted skin and skull. Loc.: Japan. Leg.: P.F. von Siebold, [captured alive 1824, died in captivity after 1829]. Figured in Temminck (1842: pl. I, figs 2, 5–7).

Syntype, RMNH.MAM.54922 (formerly 39396) (Jentink 1892: 31 *b*), male, mounted skin, skull extracted but not in collection. Loc.: Japan. Leg.: P.F. von Siebold, [1823–1829].

Syntype, RMNH.MAM.54924 (formerly 39398) (Jentink 1892: 31 *d*), female, mounted skin, skull extracted but not in collection. Loc.: Japan. Leg.: P.F. von Siebold, [1823–1829].

Syntype, RMNH.MAM.54925 (formerly 39399) (Jentink 1887: 27 *c*; 1892: 31 *e*), juvenile female, mounted skin and skeleton. Loc.: Japan. Leg.: P.F. von Siebold, [1823–1829].

Syntype, RMNH.MAM.54926 (formerly 39400) (Jentink 1892: 31 *f*), juvenile female, mounted skin, skull in situ. Loc.: Japan. Leg.: P.F. von Siebold, [1823–1829].

Syntype, RMNH.MAM.54927 (formerly 39401) (Jentink 1887: 27 *a*), male, skeleton. Loc.: Japan. Leg.: H. Bürger, [1830–1834].

Syntype, RMNH.MAM.54928 (formerly 39402) (Jentink 1887: 27 *b*), sex unknown, skeleton. Loc.: Japan. Leg.: P.F. von Siebold, [1823–1829].

Syntype, RMNH.MAM.54929 (formerly 39403) (Jentink 1887: 27 *e*), male, skull. Loc.: Japan. Leg.: H. Bürger, [1830–1834].

The complicated nomenclatural history of this species is extensively discussed by Fooden and Aimi (2005: 79–82). The name *Inuusfuscatus* was coined by Reinwardt and used by him, Temminck, and by von Siebold as a manuscript name for the specimens that were sent to Leiden from Japan. In the Fauna Japonica, however, Temminck (1842: 9–10, pls I, II) used the published name *Inuusspeciosus*, attributed by him to F. Cuvier, but in fact already given by I. Geoffroy Saint-Hilaire in 1826 (as *Macacusspeciosus*). The name *Macacusspeciosus* was then used by Schlegel (1876: 114) and Jentink (1887: 27, 1892: 31) for the Japanese macaques. However, despite Temminck’s explanation, it has been widely misapplied to the bear macaque *Macacaarctoides* (I. Geoffroy Saint-Hilaire, 1831) from Indochina. Since this has caused considerable confusion, Fooden (1967) proposed conservation of the name *Macacafuscata* (Blyth, 1875). Following this, the name *speciosus* I. Geoffroy Saint-Hilaire, 1826 was suppressed and *fuscatus* Blyth, 1875 was validated by Opinion 920 of the ICZN (Melville and China 1970: 77).

The name *Inuusfuscatus* was first published by Gray (1871a: 32) as a junior synonym of *M.speciosus* (here correctly applied to the Japanese macaque, but again attributed to F. Cuvier). Blyth (1875: 6) again misapplied the name *I.speciosus* to the bear macaque and like many authors, overlooked the fact that the name *M.speciosus* was already given to the Japanese macaque by I. Geoffroy Saint-Hilaire in 1826. Both Fooden (1967) and the ICZN (Melville and China 1970) attributed the name *I.fuscatus* to Blyth (1875), but Fooden and Aimi (2005: 80–82) correctly pointed out that technically, Gray (1871a) was the author. In accordance with ICZN art. 50.7 the name *fuscatus* should be attributed to Gray, 1871. The publication date of the Catalogue of monkeys, lemurs, and fruit-eating bats in the collection of the British Museum is usually dated from 1870. However, according to Sherborn (1934) this publication should be dated for 1871.

Since Gray exclusively referred to the specimens from Japan in Naturalis, those constitute the type series of *Inuusfuscatus* Gray, 1871a. The animals sent to Leiden by Reinwardt are no longer present in Naturalis, leaving only the collections received from von Siebold and Bürger. Most material collected by von Siebold and Bürger is only labelled “Japon”, and their precise provenance cannot be reconstructed. From shipment inventories (Naturalis archives inv. 232.1438–1440) it appears that von Siebold dispatched a skeleton of *I.fuscatus* in May 1827 and in 1828; the mammals in his last shipment, which he took with him in 1829, are not specified. There is a specification of live mammals that he brought with him to The Netherlands, which contained four monkeys. In a manuscript on the mammals of Japan by von Siebold (but copied in another hand) (Naturalis archives inv. 232.1439), used by Temminck as a source for the mammal section of the Fauna Japonica, he says: “Der große Affe, den ich lebend mit nach Leiden gebracht, wurde bereits im Jahre 1824 auf Japan gefangen. Schon damals hatte er sein volles Wachstum und zwar in freier Natur erreicht, daher er auch bis an sein Ende unbändig geblieben. Demnach mag dieser Affe ein Alter von 20 Jahren erreicht haben”. This animal (RMNH.MAM.54921) is figured in the Fauna Japonica (Temminck 1842: 11, pl. I figs 2, 5–7). The fate of the other animals is not known. The shipment inventories sent by Bürger list one mounted skin for 1831, one mounted skin and one skeleton for 1832, and one mounted animal for 1834. None of these can be associated with certainty with any of the types listed above. Since the collections of von Siebold and Bürger were not always kept apart and no original labels are preserved, it cannot be excluded that one or more specimens assumed to be from von Siebold, are in fact acquired through Bürger.

***Macacamaura* (Schinz, 1825)**

*Inuusbrachyurus* “Temminck” Kuhl, 1820a: 17 (in synonymy).

Holotype by monotypy, RMNH.MAM.39407 (Jentink 1892: 31 *b*), male, albino, mounted skin and incomplete skull. Loc.: “India”. Ex: Cabinet Temminck.

The name *Macacusbrachyurus* is generally attributed to Hamilton Smith (1842: 103, pl.1; see Groves 2005b: 162). However, Kuhl (1820a: 17) was the first to publish this name for this white variety in the synonymy of *Inuusleucophaeus* Cuvier. In his copy of Kuhl’s monograph (Naturalis library inv. RBR C00258), Temminck added a remark confirming that he believed it to be a new species. Hamilton Smith (1842) made this name available by using it as a valid name. Following the ICZN (art. 11.6) Kuhl is still the author and the original type series still applies. This also implies that *M.brachyurus* Kuhl, 1820 has priority over *M.maura* (Schinz, 1825).

***Macacanemestrina* (Linnaeus, 1766)**

*Macacanemestrinanucifera* Sody, 1936a: 42.

Holotype, RMNH.MAM.34291, male, skin of head and skull. Loc.: Bangka Island, Indonesia. Leg.: Mr Langen or W.C. van Oosterhout, November 1930–September 1932. Ex: H.J.V. Sody (Bk 75).

Sody (1936a) did not provide any information about localities, dates, or collectors for the mammals collected for him in Bangka. According to Becking (1989: 85–86, 232) they were obtained by forestry officers, in November 1930 by Mr. Langen (initials not recorded) and between May 1931 and September 1932 by W.C. van Oosterhout (most material).

***Macacanigrescens* (Temminck, 1849)**

*papionigrescens* Temminck, 1849: 111.

Lectotype, RMNH.MAM.39136 (Jentink 1892: 33 *f*), female, mounted skin, skull extracted but not in collection. Loc.: Gorontalo, Sulawesi, Indonesia. Leg.: E.A. Forsten, [12 October 1841].

Paralectotypes: RMNH.MAM.39135, 39137, 63706 (possible paralectotype).

Identifying the type material of this species is complicated; see Fooden (1969: 101–102). Temminck (1849: 111–112) described two species of macaque from Sulawesi. He did not specify the distribution areas of these neighbouring forms, nor any details on the number or provenance of the specimens he had before him. Moreover, his “*papioniger*” was not a new species, but had already been described by Desmarest (1822) as *Macacanigra*.

Schlegel (1876: 120–122) enumerated the material in Naturalis. The mounted skins with catalogue numbers 1–5 and 10 (Jentink 1892: 32–33 cat. *a*–*e*) were all collected by Forsten and labelled “Menado” (Manado). The next skins (catalogue numbers 6–9) were collected by von Rosenberg in 1864, therefore cannot have been among Temminck’s types of 1849 (Matschie 1901: 254–255; Büttikofer 1917: 19; Fooden 1969: 101). This would leave us with only two possible types collected by Forsten at Gorontalo and Tomini, respectively, in the western part of the northern peninsula: nr 6 (RMNH.MAM.39136) and nr 10 (RMNH.MAM.39137).

RMNH.MAM.39135 (Schlegel’s skin nr 2, Jentink’s skin b, with skull nr 19, Jentink’s skull *f*) must be added to the series. It was erroneously labelled Menado, though the pedestal reads Gorontalo. Its characters agree with those given for *M.nigrescens*. These three types are also specified as such by Büttikofer (1917) and Fooden (1969). Unfortunately, the skull *f* cannot be found. The skull RMNH.MAM.63706 might be the skull belonging to skin RMNH.MAM.39135 since this specimen matches the description of Büttikofer (1917: 19) of a skull of an adult female where the fifth molar didn’t break through. Elliot (1913b: 163) remarked on the specimens listed by Schlegel: “Of these, only No. 6 can be a type” which, though erroneous, may be regarded as a lectotype designation. Fooden (1969: 102) formally designated that specimen (RMNH.MAM.39136) as the lectotype.

Forsten was in northern Sulawesi from March 1840 until April 1842, interrupted by a stay on Ternate Island. He worked mainly in Minahasa district in the northeastern tip of Sulawesi (Manado and other places), but collected in Gorontalo and surroundings between 20 September and 1 November 1841, and around Pagowat (Paguat) further west between 4 and 12 November of that year; see his diary in the Naturalis archives (not catalogued). The entry for 12 October 1841 tells how his hunters brought four specimens of “Cijnocephalus niger” (3 females, 1 juvenile male) and describes how they differ markedly from those of Manado. There are no other records of specimens collected near Gorontalo or Paguat. The two females RMNH.MAM.39135 and 39136 must be of this lot. The juvenile male RMNH.MAM.39137 is labelled “Tomini” and is recorded as such by all subsequent authors. This is strange, since Forsten did not visit that place, Paguat being the westernmost locality that he reached. There can be little doubt that this youngster is the juvenile male recorded above. Gorontalo is situated at the entrance of Tomini Bay as mentioned by Forsten in his diary, and “Tomini” almost certainly stands for that, not for the town at the westernmost end of the northern peninsula. This would agree with Fooden’s (1969: 103) suspicion that the young animal was not from Tomini town that is within the range of *Macacahecki* (Matschie, 1901), but from further east. The year 1842 given on the labels and by Schlegel (1876) and Jentink (1887; 1892) is the year when the material was sent to Leiden.

***Macacatonkeana* (Meyer, 1899)**

*Cynopithecustogeanus* Sody, 1949b: 137.

Holotype, RMNH.MAM.10609, male, skin and skull. Loc.: Malenge Island, Togian Islands, Indonesia. Leg.: J.J. Menden, 3 December 1939. Ex: MZB, 27 June 1950 (MZB 6545).

Sody (1949b) examined 12 specimens for his description of which only the holotype is in Naturalis.

***Papioanubis* (Lesson, 1827)**

*Papiorubescens* Temminck, 1853: 39.

Syntype, RMNH.MAM.39138 (Jentink 1892: 35 *a*), subadult female, mounted skin, skull in situ. Loc.: Côte d’Or [Ghana]. Leg.: 1820.

Temminck (1853) gave no indication of the number of specimens available to him. A specimen in Naturalis (RMNH.MAM.63704) originating from the Kabinet des Stadhouders could have been known to Temminck, but it only entered Naturalis in 1867 through the collection of Lidth de Jeude.

***Piliocolobusbadiustemminckii* (Kuhl, 1820)**

*Colobustemminkii* [sic] Kuhl, 1820a: 7.

*Colobusrufo-fuliginus* Ogilby, 1838c: 270 (nomen novum).

Holotype by monotypy, RMNH.MAM.39128 (Jentink 1892: 7 a), adult, sex unknown, mounted skin, skull extracted but not in collection. Ex: Bullock Museum, 1819.

In the original description by Kuhl (1820a) the name is written as *temminkii*, clearly a lapsus for *temminckii.*Ogilby (1838c) introduced *rufo-fuliginus* as a substitute name for *C.fuliginosus* Ogilby, 1835 and *C.temminckii* Kuhl, 1820.

***Presbytischrysomelaschrysomelas* (Müller, 1838)**

*Semnopithecuschrysomelas* Müller, 1838: 138.

Syntype, RMNH.MAM.39140 (Jentink 1892: 12 *a*), male, mounted skin, skull extracted but not in collection. Loc.: Pontianak, Borneo, Indonesia. Leg.: P.-M. Diard, [1826]. Figured in Müller and Schlegel (1840: pl. 10 fig. 1).

Syntype, RMNH.MAM.39141 (Jentink 1892: 12 *b*), female, mounted skin, skull extracted but not in collection. Loc.: Pontianak, Borneo, Indonesia. Leg.: P.-M. Diard, [1826].

Syntype, RMNH.MAM.39142 (Jentink 1892: 12 *c*), female, mounted skin, skull extracted but not in collection. Loc.: Pontianak, Borneo, Indonesia. Leg.: P.-M. Diard, [1826]. Figured in Müller and Schlegel (1840: pl. 10 fig. 2).

Syntype, RMNH.MAM.39143 (Jentink 1892: 12 *d*), juvenile female, mounted skin, skull in situ. Loc.: Pontianak, Borneo, Indonesia. Leg: P.-M. Diard, [1826]. Figured in Müller and Schlegel (1840: pl. 11 fig. 2).

Possible syntype, RMNH.MAM.54940 (Jentink 1887: 11 *a*), sex unknown, skull. Pontianak, Borneo, Indonesia. Leg.: P.-M. Diard, [1826].

Possible syntype, RMNH.MAM.54941 (Jentink 1887: 11 *b*), sex unknown, skull. Pontianak, Borneo, Indonesia. Leg.: P.-M. Diard, [1826].

The description by Müller (1838) predated the extensive account by Müller and Schlegel (1840: 61, 71–73, pls 10, 11). According to Müller and Schlegel (1840: 71) these specimens were collected by Diard 15 years before the publication. Diard spent most of 1826 in Borneo. In his description Müller mentioned three specimens by Diard (2 females, 1 male) and one specimen (an adult female) donated to him by Dr. Fritze, the chief medical officer of the Dutch East Indies, received from Pontianak. However in the catalogues by Schlegel (1876: 47) and Jentink (1892: 12) all specimens are listed as collected by Diard.

The status of the two skulls is unknown. Both Schlegel (1876) and Jentink (1887) listed them as separate individuals and not belonging to mounted skins in the collection. Müller (1838) made no mention of these skulls in his description and all the skins have their skulls extracted. This might suggest that the skulls belong to the skins and for this reason we tentatively include them in the type series.

***Presbytiscomatafredericae* Sody, 1930**

*Pithecusaygulafredericae* Sody, 1930c: 68.

Syntype, RMNH.MAM.34316, adult male, skin and skull. Loc.: Tjoeroegilang [Curug Ilang], Java, Indonesia. Leg.: A. Samat, 28 September 1929. Ex: H.J.V. Sody (9c).

Syntype, RMNH.MAM.34318, adult male, skin and skull. Loc.: Tjoeroegilang [Curug Ilang], Java, Indonesia. Leg.: A. Samat, 30 September 1929. Ex: H.J.V. Sody (19c).

Sody (1930c) gave no information on the specimens available to him. Becking (1989: 50) erroneously listed RMNH.MAM.34316 as the holotype. Based on the collecting date this specimen must have been collected at Curug Ilang (Becking 1989: 79).

***Presbytisfemoralis* (Martin, 1838)**

*Semnopithecusneglectus* Schlegel, 1876: 45.

Syntype, RMNH.MAM.39154 (Jentink 1887: 11 a; 1892: 12 a), adult male, mounted skin and skull. Loc.: Singapore. Leg.: G.A. Frank, 1869.

Syntype, RMNH.MAM.39155 (Jentink 1887: 11 c; 1892: 13 b), adult, remnants of mounted skin and skull. Loc.: Singapore. Leg.: P. Diard, 1859.

Syntype, RMNH.MAM.39156 (Jentink 1887: 11 b; 1892: 13 c), juvenile, mounted skin and skull. Loc.: Singapore. Leg.: G.A. Frank, 1869.

***Presbytisfrontata* (Müller, 1838)**

*Semnopithecusfrontatus* Müller, 1838: 136.

Syntype, RMNH.MAM.39147 (Jentink 1887: 8 *b*; 1892: 9 *a*), adult female, mounted skin and skull. Loc.: Pamattan [Pematang], Borneo, Indonesia. Leg.: S. Müller, October 1836.

Syntype, RMNH.MAM.39148 (Jentink, 1887: 8 *a*; 1892: 9 *b*), adult male, mounted skin and skeleton. Loc.: Poulo-Lampy [Pulau Sari], Borneo, Indonesia. Leg.: S. Müller, 1836.

Syntype, RMNH.MAM.39149 (Jentink 1892: 9 *c*), juvenile male, mounted skin, skull extracted but not in collection. Loc.: Pamattan [Pematang], Borneo, Indonesia. Leg.: S. Müller, 1836.

Syntype, RMNH.MAM.39150 (Jentink 1887: 8 *c*; 1892: 9 *d*), juvenile male, mounted skin and skull. Loc.: Pamattan [Pematang], Borneo, Indonesia. Leg.: S. Müller, 1836.

***Presbytismelalophos* (Raffles, 1821)**

*Semnopithecusferrugineus* Schlegel, 1876: 42.

Syntype, RMNH.MAM.39144 (Jentink 1887: 9 *a*), adult male, skeleton, missing. Loc.: Padang, Sumatra, Indonesia. Leg.: S. Müller, 1835.

Syntype, RMNH.MAM.39145 (Jentink 1887: 10 *b*), adult female, skeleton. Loc.: Batang-Singalang, Sumatra, Indonesia. Leg.: S. Müller, August 1835.

Syntype, RMNH.MAM.39146 (Jentink 1887: 10 *c*), subadult, sex unknown, skeleton. Loc.: Padang, Sumatra, Indonesia. Leg.: S. Müller, September 1835.

Syntype, RMNH.MAM.42339 (formerly 39180) (Jentink 1892: 11 *r*), adult male, mounted skin, skull extracted but not in collection. Loc.: Padang, Sumatra, Indonesia. Leg.: S. Müller, 1835.

Syntype, RMNH.MAM.42399 (formerly 39180) (Jentink 1892: 11 *r*), juvenile, sex unknown, skull. Loc.: Padang, Sumatra, Indonesia. Leg.: S. Müller, 1835.

Syntype, RMNH.MAM.42400 (formerly 39181) (Jentink 1892: 11 *s*), adult male, mounted skin and skull. Loc.: Padang, Sumatra, Indonesia. Leg.: S. Müller, 1835.

Syntype, RMNH.MAM.52983 (formerly 39175) (Jentink 1887: 10 *d*), subadult, skull. Loc.: Padang, Sumatra, Indonesia. Leg.: S. Müller, 1835.

Syntype, RMNH.MAM.52984 (formerly 39176) (Jentink 1887: 10 *e*), subadult, sex unknown, skull. Loc.: Padang, Sumatra, Indonesia. Leg.: S. Müller, 1835.

Syntype, RMNH.MAM.52985 (formerly 39177) (Jentink 1892: 11 *q*), adult female, mounted skin and skull. Loc.: Padang, Sumatra, Indonesia. Leg.: S. Müller, 1835.

Syntype, RMNH.MAM.52986 (formerly 39178) (Jentink 1892: 11 *o*), adult female, mounted skin and skull. Loc.: Padang, Sumatra, Indonesia. Leg.: S. Müller, 1835.

Syntype, RMNH.MAM.52987 (formerly 39179) (Jentink 1892: 11 *p*), adult female, mounted skin and skull. Loc.: Padang, Sumatra, Indonesia. Leg.: S. Müller, 1835.

Syntype, RMNH.MAM.52988 (formerly 39182) (Jentink 1892: 11 *t*), juvenile female, mounted skin and skull. Loc.: Padang, Sumatra, Indonesia. Leg.: S. Müller, 1835.

The skull originally associated with the mounted specimen RMNH.MAM.42339 is clearly from a juvenile animal. As the skin is an adult specimen, we assume that Hooijer (1962: 13, no. 5) has mistakenly combined the skin and skull as being from the same specimen. This skull is hereby separated from the skin (RMNH.MAM.42339) and listed separately (RMNH.MAM.42399).

***Presbytisnatunae* (Thomas & Hartert, 1894)**

*Semnopithecusnatunae* Thomas & Hartert, 1894: 652.

Paratype, RMNH.MAM.39153, adult, sex unknown, study skin and skull. Loc.: Bunguran [Natuna Besar Island], Indonesia. Leg.: A. Everett, 4 October 1893. Ex: G.A. Frank, July 1900, from W. Rothschild (Tring).

Thomas and Hartert (1894) designated a specimen in the NHM (NHMUK 1894.9.28.1) the type. However, in the introduction they refer to the specimens in the collection of Rothschild as “co-types”, which we consider paratypes.

***Presbytissumatrana* (Müller & Schlegel, 1841)**

*Semnopithecussumatranus* Müller & Schlegel, 1841a: 61.

Syntype, RMNH.MAM.39169 (Jentink 1887: 11 *c*; 1892: 12 *a*), adult male, mounted skin and skull. Loc.: Mount Ophir [Gn. Talakmau], Sumatra, Indonesia. Leg.: L. Hörner, May 1838. Figured in Müller and Schlegel, plate 10b, fig. 1.

Syntype, RMNH.MAM.39170 (Jentink 1887: 11 *d*; 1892: 12 *b*), adult male, mounted skin and skull. Loc.: Mount Ophir, Sumatra, Indonesia. Leg.: L. Hörner, May 1838.

Syntype, RMNH.MAM.39171 (Jentink 1887: 11 *a*; 1892: 12 *c*), adult female, mounted skin and skeleton. Loc.: Mount Ophir, Sumatra, Indonesia. Leg.: L. Hörner, May 1838.

Syntype, RMNH.MAM.39172 (Jentink 1887: 11 *e*; 1892: 12 *d*), juvenile female, mounted skin and skull. Loc.: Mount Ophir, Sumatra, Indonesia. Leg.: L. Hörner, May 1838.

Syntype, RMNH.MAM.39173 (Jentink 1887: 11 *b*; 1892: 12 *e*), adult female, mounted skin and skeleton. Loc.: Mount Ophir, Sumatra, Indonesia. Leg.: L. Hörner, May 1838.

Syntype, RMNH.MAM.42367 (Jentink 1887: 11 *f*), adult male, skull. Loc.: Mount Ophir, [Kampong Sawa], Sumatra, Indonesia. Leg.: L. Hörner, [10] May 1838.

L. Hörner, a Swiss geologist in service of the Natuurkundige Commissie voor Nederlandsch-Indië climbed Mount Ophir [Gunung Talamau] in May 1838 (Hörner 1839: 609). In his description of the ascent of the mountain Hörner mentioned shooting a male of a new species of monkey in “kampong Sawa 920’ above sea level” on the 10^th^ of May, but that the damp weather destroyed the skin. This is probably the skull RMNH.MAM.42367. He did not mention any other specimens.

*Presbytisaygulamargae* Hooijer, 1948: 234.

Holotype, RMNH.MAM.39139 (Jentink 1892: 12 f), subadult female, mounted skin and mandible. Loc.: Deli, Serdang, Sumatra, Indonesia. Leg.: B. Hagen, 25 March 1887. Received 1889.

Paratypes: RMNH.MAM.42404, 1854.

***Presbytisrubicunda* (Müller, 1838)**

*Semnopithecusrubicundus* Müller, 1838: 137.

Syntype, RMNH.MAM.39158 (Jentink 1887: 8 *a*), adult female, skeleton. Loc.: Mt. Sakoumbang, Borneo, Indonesia. Leg.: S. Müller, [11 November] 1836.

Syntype, RMNH.MAM.39159 (Jentink 1892: 9 *a*), adult female, mounted skin, skull extracted but not in collection. Loc.: Tanah-Lawout, Borneo, Indonesia. Leg.: S. Müller, [21–23 October] 1836. Mother of RMNH.MAM.39164.

Syntype, RMNH.MAM.39160 (Jentink 1892: 9 *b*), adult female, mounted skin, skull extracted but not in collection. Loc.: Mt. Sakoumbang, Borneo, Indonesia. Leg.: S. Müller, [11 November] 1836.

Syntype, RMNH.MAM.39161 (Jentink 1887: 8 *b*; 1892: 9 *c*), adult male, mounted skin, skull in situ. Loc.: Kertingan, Borneo, Indonesia. Leg.: S. Müller, November 1836.

Syntype, RMNH.MAM.39162 (Jentink 1892: 9 *d*), subadult male, mounted skin, skull in situ. Loc.: Mt. Sakoumbang, Borneo, Indonesia. Leg.: S. Müller, [11] November 1836.

Syntype, RMNH.MAM.39163 (Jentink 1887: 9 *c*; 1892: 9 *e*), subadult, sex unknown, mounted skin and skull. Loc.: Borneo, Indonesia. Leg.: S. Müller, 1836.

Syntype, RMNH.MAM.39164 (Jentink 1887: 9 *d*; 1892: 9 *f*), juvenile female, mounted skin and skull. Loc.: Tanah-Lawout, Borneo, Indonesia. Leg.: S. Müller, [21–23 October] 1836. Young of RMNH.MAM.39159.

Syntype, RMNH.MAM.42332 (Jentink 1887: 8 *a*), adult male, skull. Loc.: Borneo, Indonesia. Leg.: S. Müller, 1836.

Syntype, RMNH.MAM.42333 (Jentink 1887: 8 *a*), adult female, skull. Loc.: Borneo, Indonesia. Leg.: S. Müller, 1836.

Syntype, RMNH.MAM.42334 (Jentink 1887: 8 *a*), adult female, skull. Loc.: Borneo, Indonesia. Leg.: S. Müller, 1836.

***Presbytissiamensis* (Müller & Schlegel, 1841)**

*Semnopithecussiamensis* Müller & Schlegel, 1841a: 60.

Syntype, RMNH.MAM.39166 (Jentink 1892: 11 *f*), adult male, mounted skin, skull extracted but not in collection. Loc.: Malaysia. Leg.: P. Diard [1818–1824].

Syntype, RMNH.MAM.39167 (Jentink 1892: 11 *g*), adult male (originally identified as female), mounted skin, skull extracted but not in collection. Loc.: Malaysia. Leg.: P. Diard [1818–1824].

Syntype, RMNH.MAM.39168 (Jentink 1892: 11 *h*), adult female, mounted skin, skull extracted but not in collection. Loc.: Malaysia. Leg.: P. Diard [1818–1824].

Diard accompanied T.S. Raffles to Malakka (Penang) and Sumatra in the years 1818–1819. He collected in the Malay Peninsula until 1824, when he went to Java (Fransen et al. 1997: 228).

According to Schlegel (1876: 39) the skins of these types show yellowing due to preservation in alcohol. The whereabouts of the skulls is unknown; in their description Müller and Schlegel (1841a) mentioned skulls and listed four specimens from Diard. In the collection and in the catalogues by Schlegel (1876: 39) and Jentink (1892: 11) there are only three specimens listed and there is no mention of skulls collected by Diard.

***Rhinopithecusroxellana* (Milne-Edwards, 1870)**

*SemnopithecusRoxellana* Milne-Edwards, 1870: 341.

Syntype, RMNH.MAM.39157 (Jentink 1892: 17 *a*), adult female, mounted skin, skull extracted but not in collection. Loc.: Moupin [Baoxing], China. Leg.: A. David. Ex: MNHN, 1875.

***Semnopithecusschistaceus* Hodgson, 1840**

*Semnopithecusschistaceus* Hodgson, 1840: 1212.

Paralectotype, RMNH.MAM.39165 (Jentink 1892: 16 *b*), adult female, mounted skin, skull in situ. Loc.: Nepal. Leg.: B.H. Hodgson.

Hodgson (1840) assigned this new name to a species until then listed in his unpublished catalogue as *nipalensis* (see Hodgson 1842: 212). Thomas (1918:370) designated a specimen in the NHM (NHMUK 1843.1.12.1) as the lectotype.

***Semnopithecusvetulus* (Erxleben, 1777)**

*SimiaLatibarba* Temminck, 1807: 3.

*Cercopithecuslatibarbatus* É. Geoffroy Saint-Hilaire, 1812: 94 (incorrect subsequent spelling).

Syntype, RMNH.MAM.42199 (Jentink 1892: 14 *e*), male, mounted skin. Loc.: Ceylon [Sri Lanka]. Leg.: unknown, 1800. Ex: Cabinet Temminck.

Temminck (1807: 3) listed this species in the catalogue of his collection as *Simialatibarba* but gave no description. However, by including a reference to the “Guenon a face pourpre” in De Buffon (1789: 117, pl. 21), according to the code (ICZN art. 12.2.1) this name was validly published by indication.

É. Geoffroy Saint-Hilaire (1812: 94) attributed the name *latibarbatus* to Temminck (1807), which we consider to be an incorrect subsequent spelling.

*Semnopithecuskelaartii* Schlegel, 1876: 30, 52.

Syntype, RMNH.MAM.42213 (Jentink 1892: 14: *a*), adult male, mounted skin, skull extracted but not in collection. Loc.: South Ceylon, Sri Lanka. Leg.: P. Diard, 1859.

Schlegel (1876) introduced this name based on the description of ‘*Presbytispriamus*’ in Kelaart (1852: 3) from Sri Lanka and a single specimen in Naturalis. Diard visited Sri Lanka sometime between 1857 and 1859.

Groves (2005b: 178) erroneously spelled this name as *kelaarti*.

***Theropithecusgeladagelada* (Rüppell, 1835)**

*MacacusGelada* Rüppell, 1835: 5, Taf. 2.

*GeladaRüppellii* Gray, 1843: 77 (nomen novum).

Paralectotype, RMNH.MAM.39174 (Jentink 1887: 25 *a*; 1892: 29 *a*), adult male, mounted skin and skull. Loc.: Simēn Mountains, Ethiopia. Leg.: E. Rüppell, [June–September 1832]. Received 15 October 1835.

Rüppell was in the Simēn Mountains from June until the first week of October 1832 (Mertens 1949: 68–75). RMNH.MAM.39174 was sent by Rüppell in exchange on 15 October 1835. It is mentioned as “1 Macaca gelada R” in a list of specimens enclosed with Rüppell’s letter (Naturalis archives inv. 255.577).

Gray (1843: 77) listed Rüppell’s *Macacusgelada* under the new name *Geladarüppellii* and referred to the description by Rüppell (1835), thus including all original specimens in the type series of this new name.

Mertens (1925: 36) designated a specimen in the SMF (Nr 1011) as the lectotype of *Theropithecusgelada* (Rüppell, 1835).

***Theropithecusgeladaobscurus* Heuglin, 1863**

*Theropithecusobscurus* von Heuglin, 1862: 427 (nomen nudum).

*Theropithecusobscurus* von Heuglin, 1863: 10.

Syntype, RMNH.MAM.39130 (Jentink 1887: 25 *e*), subadult female, skull. Loc.: Sources of Tekeze River, Ethiopia. Leg.: T. von Heuglin, [March 1862]. Received 1865.

Syntype, RMNH.MAM.39131 (Jentink 1887: 25 *a*; 1892: 29 *a*), male, mounted skin and skull. Loc.: Sources of Tekeze River, Ethiopia. Leg.: T. von Heuglin, [March 1862]. Received 1865.

Syntype, RMNH.MAM.39132 (Jentink 1887: 25 *b*; 1892: 29 *b*), female, mounted skin and skull. Loc.: [Sources of] Tekeze River, Ethiopia. Leg.: T. von Heuglin, [March 1862]. Received 1865.

Syntype, RMNH.MAM.39133 (Jentink 1887: 25 *c*; 1892: 29 *c*), female, mounted skin and skull. Loc.: [Sources of] Tekeze River, Ethiopia. Leg.: T. von Heuglin, [March 1862]. Received 1865.

Syntype, RMNH.MAM.39134 (Jentink 1887: 25 *d*; 1892: 30 *d*), juvenile male, mounted skin and skull. Loc.: [Sources of] Tekeze River, Ethiopia. Leg.: T. von Heuglin, [March 1862]. Received 1865.

This name was first published by von Heuglin (1862) as a nomen nudum. The formal description appeared the following year (von Heuglin 1863).

The labels and pedestals of all skins, as well as that of the separate skull, bear the locality “Takassie”, the pedestals of RMNH.MAM.39130 and 39131 specifying this further, in Schlegel’s handwriting, as “Bronnen” [sources] of this river. The exact locality where the Naturalis animals were collected cannot be traced. Von Heuglin (1863: 10) wrote about a presumed second species of *Theropithecus* near the sources of the Takasseh river. He found this species on a trip from Begemeder to Wollo-Galla in great numbers. This is further south than the main sources of Tekeze River, although some smaller tributaries originate from those highlands at the other side of the watershed. Von Heuglin stayed in this area 13–28 March 1862, but did not mention a visit to the sources of the Tekeze (von Heuglin 1862: 424–425), and the locality where the specimens were collected may in fact have been near the southern edges of these highlands.

Schlegel (1876) recorded that the Naturalis series was received in 1865; this year is also given by Jentink (1887; 1892), but only for his skin and skull RMNH.MAM.39131. This is not in agreement with the correspondence between von Heuglin and Schlegel (Naturalis archives inv. 209.435). In a letter dated Chartum, 5 January 1863, von Heuglin announced the despatch of “Schädel von *Theropithecusobscurus* Heugl. 1 sts” and “Bälge von *Theropithecusobscurus* 2. sts (1 sehr altes Männchen u. 1. junges)”. These must be RMNH.MAM.39130, 39131 and 39134, respectively. These two skins appear in a specification of payments to von Heuglin dated 1 March 1864 (Naturalis archives inv. 209.435); the separate skull is not mentioned here. The remaining specimens may indeed have been received in 1865, but there is no record. Given the locality on the labels and pedestals, they must all belong in the same series and thus are to be regarded as types. Hill (1970: 603) erroneously located the type series in the NMW; see the correction by Engelberger (2010).

Dieterlen et al. (2013) listed three syntypes in the Museum of Natural History Stuttgart (SMNS 581, 1032, 1033, 1034).

***Trachypithecusauratus* (E. Geoffroy, 1812)**

*Pithecuspyrrhuskohlbruggei* Sody, 1931b: 349.

Holotype, RMNH.MAM.34328, adult female, skin and skull. Loc.: Sendang, Bali, Indonesia. Leg.: A. Samat, 15 July 1930 (E.42).

Paratypes: RMNH.MAM.26118, 34323–34327.

The series was collected for Sody by A. Samat, preparator at the Bogor Museum (Becking, 1989: 94).

***Trachypithecusobscurus* (Reid, 1837)**

*Semnopithecusleucomystax* Müller & Schlegel, 1841a: 59.

Syntype, RMNH.MAM.39151 (Jentink 1892: 13 *f*), subadult male, mounted skin, skull extracted but not in collection. Loc.: Malacca, Malaysia. Leg.: P.-M. Diard [1818–1824].

Syntype, RMNH.MAM.39152 (Jentink 1892: 13 *g*), subadult female, mounted skin, skull extracted but not in collection. Loc.: Malacca, Malaysia. Leg.: P.-M. Diard [1818–1824].

Müller and Schlegel (1841a) tentatively described these animals as a new species, already suggesting that they could be identical with *Semnopithecusobscurus* Reid, 1837. Later Schlegel (1876: 49) included them in that species. According to Schlegel the skins were kept in alcohol.

Diard accompanied T.S. Raffles to Malakka (Penang) and Sumatra in the years 1818–1819. He collected in the Malay Peninsula until 1824, when he went to Java (Fransen et al. 1997: 228).

*Trachypithecuspopa*Roos et al., 2020: 9

Paratype, RMNH.MAM.59807 (Jentink 1892: 13 l), male, skin, skull in situ. Loc.: Yado, Mt. Cariani, E. di. Tounghoc, Myanmar, 800–1000 m. Leg. L. Fea, December 1887.

Based on molecular, morphological, and biogeographical data Roos et al. (2020) introduced this new species, which was until then included in *Trachypithecusphayrei* (Blyth, 1847). This new name was published shortly after Zinner et al. (2020) and is therefore used as the preferred name here.

#### ﻿﻿Hylobatidae Gray, 1871

***Hylobatesmoloch* Audebert, 1798**

*Hylobateslarpongoalsoni* Sody, 1949b: 122.

Holotype, RMNH.MAM.34322, adult female, study skin and skull. Loc.: Kali Kidang, Mt Slamet, Central Java, 800 m. Leg.: A. Samat, 18 October 1929. Ex H.J.V. Sody (C. 132).

Sody (1949b) restricted *Hylobatesmoloch* to West Java and renamed the population from Central Java as *Hylobateslarpongoalsoni*. He mentioned five specimens of which only the holotype is in Naturalis.

*Hylobatesmuelleri* Martin, 1841: 444.

Syntype, RMNH.MAM.42117 (Jentink 1887: 5 *b*; 1892: 5 *f*), juvenile female, mounted skin and skull. Loc.: Southeast Borneo, Indonesia. Leg.: S. Müller, [28 July 1836–17 December 1836]. Received 1837.

Syntype, RMNH.MAM.42134 (Jentink 1892: 5 *a*), adult male, mounted skin, skull extracted but not in collection. Loc.: Mount Sakoumbang [Sekumbang], Southeast Borneo. Leg.: S. Müller, [28 July 1836–17 December 1836]. Received 1837.

Syntype, RMNH.MAM.42135 (Jentink 1892: 13 *b*), adult male, mounted skin, skull extracted but not in collection. Loc.: Pamattan [Pematang], Southeast Indonesia. Leg.: S. Müller, October 1836.

Syntype, RMNH.MAM.42144 (Jentink 1892: 4 *f*), subadult female, mounted skin, skull in situ. Loc.: “Java Occ.” [= Borneo], Indonesia. Leg.: S. Müller.

Martin (1841) referred to Müller’s description of the gibbons he collected in Borneo under the heading of *Hylobatesconcolor* (Müller, 1840a: 48). According to Martin these animals constitute a new species he named *Hylobatesmuelleri*. Müller did not mention any particular specimens but he clearly based his description on his own material. The above-listed specimens are catalogued by Jentink (1887: 5; 1892: 5) as *Hylobatesmuelleri*, but he failed to recognise their type status.

RMNH.MAM.42144 was originally labelled as originating from “Java Occ.”. As this species does not occur there, probably the labels have been mixed up.

Lyon (1911: 142) restricted the type locality to southeastern Borneo.

### ﻿﻿RODENTIA

#### ﻿﻿Sciuridae Fischer de Waldheim, 1817

***Aeromystephromelas* (Günther, 1873)**

*Petauristabartelsi* Sody, 1936b: 146.

Holotype, RMNH.MAM.24076, adult male, skin and skull. Loc.: Pagar Djawa, Pematang Siantar, Deli, Sumatra, Indonesia (500 m.). Leg.: H. Bartels, 1934. Ex: M. Bartels Jr.

Paratype: RMNH.MAM.64148.

***Belomyspearsoniikaleensis* (Swinhoe, 1863)**

*Sciuropteruskaleënsis*Swinhoe 1863: 359.

Syntype, RMNH.MAM.39333 (Jentink 1887: 183 *a*; 1888: 5 *a*), adult, sex unknown, relaxed mount and skull. Loc.: Formosa [Taiwan]. Leg.: R. Swinhoe. Ex: G.A. Frank, 1865.

Swinhoe (1863) described how he received two immature specimens of this new species through a local. Although RMNH.MAM.39333 is identified as an adult, we follow Jentink (1887: 183; 1888: 5), who referred to this specimen as one of the types.

According to Illar Muul (in litt., 1972) this specimen has been wrongly identified and it belongs to *Hylopetesphayrei* (Blyth, 1859). However, this species is not known from Taiwan, so if the locality is correct it would represent the first record of this species from that area. Since Swinhoe did not collect these specimens from the wild, human translocation of these specimens from another location is more likely. Considering the re-identification by Ilar Muul, it either is a mixed type series for *S.kaleensis* or this form should be a synonym of *Hylopetesphayrei* instead of *Belomyspearsonii*.

***Callosciuruscanicepsbimaculatus* (Temminck, 1853)**

*Sciurusbimaculatus* Temminck, 1853: 251.

Holotype by monotypy, RMNH.MAM.13363 (Jentink 1887: 188 *c*; 1888: 20 *d*), adult male, mounted skin and skull. Loc.: Malacca, Malaysia.

***Callosciurusfinlaysoniicinnamomeus* (Temminck, 1853)**

*Sciuruscinnamomeus* Temminck, 1853: 250.

Syntype, RMNH.MAM.13367 (Jentink 1888: 19 c; 1887: 188 a), adult, sex unknown, mounted skin and skull. Loc.: Cambodia. Leg.: P. Diard, [May–August 1822].

Syntype, RMNH.MAM.13368 (Jentink 1888: 19 d; 1887: 188 b), adult male, mounted skin and skull. Loc.: Cambodia. Leg.: P. Diard, [May–August 1822].

Syntype, RMNH.MAM.13369 (Jentink 1888: 19 e), adult, sex unknown,mounted skin, skull in situ. Loc.: Cambodia. Leg.: P. Diard, [May–August 1822].

Diard collected in Cambodia from May–August 1822 (Van Wingerden 2023: 131).

***Callosciurusnigrovittatusnigrovittatus* (Horsfield, 1823)**

*Callosciurusnigrovittatusmadsoedi* Sody, 1929a: 163.

Syntype, RMNH.MAM.10053, adult female, skin and skull. Loc.: Gn. Moeria, Kp. Gingsir, Desa Rahtowu, Java, Indonesia. Leg.: H.J.V. Sody (22), 16 December 1928.

Syntype, RMNH.MAM.63703, adult female, skin and skull. Loc.: Pangonan, Gn. Moeria (E. slope, 550 m.), Java, Indonesia. Leg.: H.J.V. Sody (M 36), 18 December 1928.

Sody (1929a) introduced this new name in a footnote, with a very short description. He gave no indication of the number of specimens available to him. In a later publication Sody (1949a: 101) mentioned having measured four skulls; only two are present in the Naturalis collection.

*Callosciurusnigrovittatussalakensis* Sody, 1949a: 98.

Holotype, RMNH.MAM.9829, adult female, skin and skull. Loc.: Tjianten, Mt. Salak, Java, Indonesia. Leg: P. Franck, 29 December 1931. Ex: MZB (MZB 3278), 12 January 1950.

Paratypes: RMNH.MAM.24532–24543, 24553, 24555–24559, 24670.

*Callosciurusnigrovittatusbantamensis* Sody, 1949a: 99.

Holotype, RMNH.MAM.9830, adult male, skin and skull. Loc.: Tjikudjang [Cikujang], Bantam, Java, Indonesia. Leg.: P.F. Franck, 28 July 1932. Ex: MZB (MZB 3636), 12 January 1950.

*Callosciurusnigrovittatusphoenicurus* Sody, 1949a: 99.

Holotype, RMNH.MAM.9831, adult male, skin and skull, baculum. Loc.: Mt. Tjerimai (1000 m.), Cheribon, Java, Indonesia. Leg.: J.J. Menden, 23 February 1931. Ex: MZB (MZB 2677), 12 January 1950.

Sody (1949a) examined 12 specimens; only the holotype is in Naturalis.

*Callosciurusnigrovittatustenggerensis* Sody, 1949a: 99.

Holotype, RMNH.MAM.9828, adult female, skin and skull. Loc.: Ranu Pani, Tengger Mts., Java, Indonesia. Leg.: A.C.V. van Bemmel, 08 July 1939. Ex: MZB (MZB 3627; 74/39), 12 January 1950.

Sody (1949a) examined 10 specimens of which only the holotype is in Naturalis.

***Callosciurusnotatusdiardii* (Jentink, 1879)**

*Sciurus Diardii* Jentink, 1879b: 38.

Holotype by monotypy, RMNH.MAM.13366 (Jentink 1887: 189 *a*; 1888: 21 *a*), adult female, mounted skin and skull. Loc.: Nusa Kambangan, Indonesia. Leg.: C.L. Blume, [3 November 1824–1 December 1824]. Ex: P. Diard.

Blume (also written as Blüme) collected on Nusa Kambangan sometime between 3 November and 1 December 1824 (Van Steenis-Kruseman 1950: 64).

*Callosciurusnotatusvanheurni* Sody, 1929b: 327.

Holotype, RMNH.MAM.22906, adult male, skin and skull. Loc.: Tjipanas [Cipanas] near Garoet, Java, Indonesia. Leg.: W.C. van Heurn, 6 November 1927. Ex: H.J.V. Sody.

Possible paratypes: RMNH.MAM.2850, 13377, 63729, 63730.

Sody (1929b) based his description on four males in his own collection, which were sent to him on alcohol by Van Heurn. Naturalis holds five specimens from Tjipanas, collected by Van Heurn in 1927, possibly all seen by Sody. On the label of RMNH.MAM.22906 “type” is written by Sody and the data agrees with the description.

The type status of the other four specimens is unclear. On the label of RMNH.MAM.13377 (same data as RMNH.MAM.22906) is added “Lectotype - Tate - 1951”. No publication of this lectotype designation is found. Besides, there is no need for a lectotype designation, as Sody clearly designated a type. However, RMNH.MAM.13377 could be a paratype and RMNH.MAM.2850, 63729–63730 could also be paratypes. Unfortunately, there is no indication that these were part of Sody’s collection, something that Sody specifically stated. Additionally, there is also a female (RMNH.MAM.64142) collected by Van Heurn with similar data as the males, but Sody did not refer to any females in his description.

*Callosciurusnotatusverbeeki* Sody, 1929b: 330.

Holotype, RMNH.MAM.13375, adult female, skin and skull. Loc.: Rembang, Bandar, Java, Indonesia. Leg.: H.J.V. Sody, 10 December 1927.

Paratypes: RMNH.MAM.24926, 24929–24940, 24943, 24971, 24972.

Sody (1929b) examined 23 specimens, of which the above types are in Naturalis.

*Callosciurusnotatusprinsulae* Sody, 1949a: 89.

Holotype, RMNH.MAM.9834, adult male, skin and cranium, baculum. Loc.: Prinsen Island [Panaitan], Indonesia. Leg.: K.W. Dammerman, 23 July 1929. Ex: MZB (MZB 712), 12 January 1950.

*Callosciurusnotatusmagnificus* Sody, 1949a: 92.

Holotype, RMNH.MAM.9832, adult male, skin and skull. Loc.: Nusa Barung, Indonesia. Leg.: A. Hoogerwerf, 03 July 1939. Ex: MZB (MZB 6096; 47/39), 12 January 1950.

Of the seven specimens examined by Sody (1949a) only the holotype is in Naturalis.

***Callosciurusnotatussuffusus* Bonhote, 1901**

*Callosciurusnotatusvinocastaneus* Sody, 1949a: 86, 93.

Holotype, RMNH.MAM.9833, adult female, skin and skull. Loc.: Kali Tjempaga, Borneo, Indonesia. Leg.: J.J. Menden, 17 June 1935. Ex: MZB (MZB 6140; 132/35), 12 January 1950.

Of the 18 specimens used by Sody (1949a) for his description only the holotype is in Naturalis.

***Callosciurusnotatusvittatus* (Raffles, 1821)**

*Callosciurusnotatusnicotianae* Sody, 1936c: 217.

Holotype, RMNH.MAM.13376, adult female, skin and skull. Loc.: Deli, Silalas, Sumatra, Indonesia. Leg.: H.J.V. Sody (22), 06 December 1927.

Paratypes: RMNH.MAM.25026–25028, 25032–25033, 25036, 25039–25041, 25043–25044, 25047.

Sody (1936c) examined 14 specimens; we have been able to locate 13 in the Naturalis collection.

*Sciurusnotatuspercommodus* Chasen, 1940a: 489.

Holotype, RMNH.MAM.9835, adult female, skin and skull. Loc.: Lesten, Atjeh, Sumatra, Indonesia. Leg.: A. Hoogerwerf, N. Sumatra Expeditie Atjeh I–V, 1937 (113), 21 March 1937. Ex: MZB (MZB 6046; 416/37), 12 January 1950.

Chasen (1940a) examined 10 specimens, only the holotype is in Naturalis

*Callosciurusnotatuskalianda* Sody, 1949a: 88.

Holotype, RMNH.MAM.9836, adult female, skin and skull. Loc.: Kalianda, Lampongs, Indonesia. Leg.: J.J. Menden, 02 August 1934. Ex: MZB (MZB 6065; 108/34), 12 January 1950.

Sody (1949a) examined 4 specimens; only the holotype is in Naturalis.

***Callosciurusnotatus* (Boddaert, 1785)**

Sciurus (Rheithrosciurus) microtis Jentink, 1879b: 41.

Lectotype, RMNH.MAM.13349 (Jentink 1888: 29 *e*), female, mounted skin and incomplete skull. Loc.: Salayar, Indonesia. Leg.: J.E. Teysmann, [16 November–11 December 1877]. Received 1878.

Paralectotypes: RMNH.MAM.13345–13348, 13350.

Teysmann (also spelled Teijsmann) worked on Selayar from 16 November to 11 December 1877 (Van Steenis-Kruseman 1950: 524). The year 1878 given by Jentink (1887: 193; 1888: 29) thus refers to the year when the material was received in Naturalis. The species is almost certainly introduced on Selayar from elsewhere in the archipelago (Musser 1987: 80; Musser et al. 2010: 197–198).

The lectotype has been designated by Musser et al. (2010: 196).

***Callosciurusprevostiiprevostii* (Desmarest, 1822)**

Sciurusrafflesiivar.indica Müller & Schlegel, 1844: 86 (nec Erxleben).

Syntype, RMNH.MAM.60456. (Jentink 1888: 26 *a*) adult male, mounted skin, skull extracted but not in collection. Loc.: Malacca. Leg.: P.-M. Diard.

Syntype, RMNH.MAM.60457 (Jentink 1888: 26 *b*) adult male, mounted skin, skull extracted but not in collection. Loc.: Malacca. Leg.: P.-M. Diard.

Syntype, RMNH.MAM.60458 (Jentink, 1888: 26 *c*) adult female, mounted skin, skull extracted but not in collection. Loc.: Malacca. Leg.: P.-M. Diard.

For the publication date, see *Sciurusrafflesii var. borneoensis*.

***Callosciurusprevostiiatricapillus* (Schlegel, 1863)**

*Sciurusatricapillus* Schlegel, 1863: 27, pl. 2 fig. 1.

*Macroxusatrocapillus* Gray, 1867: 278 (incorrect subsequent spelling).

Syntype, RMNH.MAM.13383 (Jentink 1888: 26 *s*), male, mounted skin and skull. Loc.: [Lower] Kapuas [Bohang] River, Borneo, Indonesia. Leg.: C.A.L.M. Schwaner, [28 January–2 February 1848].

Syntype, RMNH.MAM.13384 (Jentink 1888: 26 *r*), male, mounted skin and skull. Loc.: [Lower] Kapuas [Bohang] River, Borneo. Leg.: C.A.L.M. Schwaner, [28 January–2 February 1848].

Syntype, RMNH.MAM.13385 (Jentink 1888: 26 *t*), female, mounted skin, skull in situ. Loc.: [Lower] Kapuas [Bohang] River, Borneo. Leg.: C.A.L.M. Schwaner, [28 January–2 February 1848].

Syntype, RMNH.MAM.13386 (Jentink 1888: 26 *u*), juvenile female, mounted skin and skull. Loc.: [Lower] Kapuas [Bohang] River, Borneo. Leg.: C.A.L.M. Schwaner, [28 January–2 February 1848].

Syntype, RMNH.MAM.13387 (Jentink 1888: 26 *v*), male, mounted skin and skull. Loc.: [Upper] Duson [Barito] River, Borneo. Leg.: C.A.L.M. Schwaner, [1843–1846].

There are two rivers in Borneo named Kapuas: the Kapuas Bohang in western Borneo, and the Kapuas Murung in the southeast. Schwaner visited both rivers, but the latter only very briefly, on 21–22 November 1847. Between 28 January and 2 February 1848 he travelled down the Kapuas Bohang, from Sintang to Pontianak (Schwaner 1854: 39–44, 188–200). Schlegel (1863) gave the locality as ”le district de Kapouas, situé, dans l’Intérieur, à quatre degrés environ à l’est de la ville de Pontianak”, which is the Kapuas Bohang. The fifth specimen was collected, according to Schlegel, “sur les bords du haut Douson, à peu près dans le centre de l’île.” The precise locality or date cannot be traced; it must have been in the period 1843–1846.

Gray (1867: 278) used *atrocapillus* but without any justification. This is an incorrect subsequent spelling and therefore not an available name (ICZN art. 33.3).

***Callosciurusprevostiiborneoensis* (Müller & Schlegel, 1842)**

*Sciurusrafflesii var. borneoensis* Müller & Schlegel, 1844: 86.

Syntype, RMNH.MAM.13380 (Jentink 1887: 192 *c*; 1888: 26 *p*), female, mounted skin and skull. Loc.: Pontianak, Borneo, Indonesia. Leg.: P.-M. Diard (32), [1826].

Syntype, RMNH.MAM.13381 (Jentink 1887: 192 *b*; 1888: 26 *o*), male, mounted skin and skull. Loc.: Pontianak, Borneo. Leg.: P.-M. Diard (31), [1826].

Syntype, RMNH.MAM.13382 (Jentink 1887: 192 *d*; 1888: 26 *q*), female, mounted skin and skull. Loc.: Pontianak, Borneo. Leg.: P.-M. Diard (33), [1826].

The date for this publication was given by most authors as 1842 (Thorington and Hoffmann 2005: 779; Koprowski et al. 2020: 592); however, the date of publication of issue 10 of the Verhandelingen in which the pages 85–100 of the Mammalia chapter appear were published on 22 March 1844 (Husson and Holthuis 1955: 23).

Diard collected in Borneo during the second half of 1826 (Veth 1879: 62; Van Steenis-Kruseman 1950: 136).

*Callosciurusprevostiicoomansi* Sody, 1949a: 103.

Holotype, RMNH.MAM.9838, adult female, skin and skull. Loc.: Pematang Tudjuh, Borneo, Indonesia. Leg.: Madzoed, 14 March 1931. Ex: MZB (MZB 2898), 12 January 1950.

Sody (1949a) examined three specimens; only the holotype is in Naturalis.

***Callosciurusprevostiirafflesii* (Vigors & Horsfield, 1828)**

*Sciurusredimitus* Van der Boon Mesch, 1829: 243.

*Sciurusprevostiisumatranus* Schlegel, 1863: 25.

Schlegel (1863) introduced this subspecies (“conspecies”) for the Sumatran form of *Sciurusprevostii* and placed the Sumatran *Sciurusrafflesii* and *Sciurusredimitus* in synonymy. The type series therefore consists of the holotype for *S.rafflesii* (a single specimen collected by Raffles, not in Naturalis) and *S.redimitus*.

Schlegel (1863: 26) mentioned having taken the type specimen for *Sciurusredimitus* from alcohol himself in 1825 and stated its origin as Sumatra. According to Van der Boon Mesch (1829) the specimen came from the collection of J.P. van Braam and originated from the East Indies, “India orientali”. Gray (1867: 278) used this name in the combination Macroxusrufogularisvar.redimitus, referring to Boon Mesch; however, he placed this taxon in Borneo.

We have not been able to locate this syntype.

*Callosciurusprevostiiwaringensis* Sody, 1949a: 103.

Holotype, RMNH.MAM.9837, adult male, skin and skull. Loc.: Riam, Borneo, Indonesia. Leg.: J.J. Menden, 06 December 1935. Ex: MZB (MZB 6188; 28/36), 12 January 1950.

Of the ten specimens examined by Sody (1949a) only the holotype is in Naturalis.

***Callosciurusprevostii* (Desmarest, 1822)**

*Sciurusprevostiibangkanus* Schlegel, 1863: 26, pl. 1 fig. 2.

Holotype by monotypy, RMNH.MAM.13373 (Jentink 1888: 26 *l*), sex unknown, mounted skin and skull. Loc.: Bangka, Indonesia. Leg.: J.F.R.S. van den Bossche, [1859–1860].

Van den Bossche worked on Bangka between 1859 and 1861; he did not provide details of provenance or collection dates of his specimens, which he obtained from local hunters. His first consignment, despatched in July 1860 and received in Naturalis in June 1861 (Naturalis archives inv. 232.973), included several mammals. The year 1861 given by Jentink (1888: 26) refers to the date when the collection arrived. Mees (1986: 9–10) assumed that the majority of this material was collected in the surroundings of Mentok, which was Van den Bossche’s residence.

*Sciuruserythromelas* Temminck, 1853: 248.

*Sciuruserythrogenys* Schlegel, 1863: 29, pl. 2 fig. 3 (nec Waterhouse, 1843).

*MacroxusSchlegelii* Gray, 1867: 278 (nomen novum).

*Sciurusrubicaudus* “Temminck” Jentink, 1887: 27 (in synonymy).

Lectotype for *S.erythrogenys* and *M.schlegelii*, RMNH.MAM.13378 (Jentink 1888: 27 *bb*), male, mounted skin and skull. Loc.: Kema, Sulawesi, Indonesia. Leg.: E.A. Forsten, [1840–1842]. New lectotype designation.

Paralectotype for *S.erythromelas* only: RMNH.MAM.13379.

Temminck (1853) did not state how many specimens he had before him, but his description makes clear he had more than two skins. The provenance is specified (p. 249) as Gorontalo and Kema. There are two specimens in Naturalis, from Manado and Kema, none from Gorontalo. The animal from Manado (RMNH.MAM.13379) is also described and illustrated by Schlegel (1863: 28–29, pl. 2 fig. 2) and he renamed the specimen from Kema (RMNH.MAM.13378) as *Sciuruserythrogenys*.

Forsten collected in NE Sulawesi between March 1840 and April 1842; see under *Prosciurillusleucomus* for more details. His diary, a copy (in an unknown hand) is preserved in Naturalis (not catalogued), sheds no light on the provenance and collection dates of these animals. The squirrels described here must have been introduced from Borneo (Musser 1987: 80; Musser et al. 2010: 201), perhaps as pets; the coastal towns of Manado, Kema, and Gorontalo have always been centres of trade. Corbet and Hill (1992: 290–91) place *erythromelas* in synonymy of *Callosciurusbaluensis* (Bonhote, 1901). We follow Thorington and Hoffmann (2005) and Musser et al. (2010) and identify these specimens as *Callosciurusprevostii*, one of the Bornean races.

Jentink (1888: 27) listed these specimens as types of the manuscript name *rubicaudus* by Temminck in the synonymy of *prevostii.* Jentink provided no description and this name has not been used as a valid name since, so this is not an available name.

Schlegel (1863) regarded the two squirrels from NE Sulawesi named *Sciuruserythromelas* by Temminck (1853: 248) as specifically distinct. He restricted the name *S.erythromelas* to the specimen from Manado (RMNH.MAM.13379) and described the animal obtained at Kema as *S.erythrogenys*.

Since that name is preoccupied by *Sciuruserythrogenys* Waterhouse, 1843 from Fernando Póo, Gray (1867: 278) has renamed this form, which he includes in his *Macroxusatrocapillus* (= *Sciurusatricapillus* Schlegel, 1863, see above), writing: “See also *Sc.Schlegelii* (with cheeks red), *Sc.erythrogenys*, Schlegel, *l.c.* t. 2. f. 3 (1863), not Waterhouse”, thus referring to the same specimen.

The lectotype for *Sciuruserythromelas* Temminck, 1853 has been designated by Musser et al. (2010: 198). They erroneously referred to RMNH.MAM.13378 as the “holotype” for *Sciuruserythrogenys* and *MacroxusSchlegelii* Gray, 1867. According to ICZN art. 74.7 this does not constitute a valid lectotype designation. In our opinion Musser et al. (2010) intended to designate RMNH.MAM.13378 as the lectotype. To rectify this error, we formally designate RMNH.MAM.13378 the lectotype for *Sciuruserythrogenys* and *Macroxusschlegelii* Gray, 1867.

***Callosciuruspygerythruslokroides* (Hodgson, 1836)**

*SciurusLokroides* Hodgson, 1836: 232.

Paralectotype, RMNH.MAM.13364 (Jentink 1887: 188 *a*; 1888: 19 *a*), adult, sex unknown, mounted skin and skull. Loc.: Nepal. Leg.: B.H. Hodgson.

Paralectotype: RMNH.MAM.13365.

Thomas (1918: 371) designated the lectotype in the NHM (NHMUK 1843.1.12.58).

***Dremomyslokriahlokriah* (Hodgson, 1836)**

*SciurusLokriah* Hodgson, 1836: 232.

Paralectotype, RMNH.MAM.39350 (Jentink 1888: 20 *a*), adult, sex unknown, mounted skin and skull. Loc.: Tibet. Leg.: B.H. Hodgson.

Paralectotype: RMNH.MAM.39351.

Thomas (1918: 371) designated the lectotype in the NHM (NHMUK 1843.1.12.58).

***Epixerusebiiebii* (Temminck, 1853)**

*Sciurusebii* Temminck, 1853: 129.

Lectotype, RMNH.MAM.19626 (Jentink 1888: 32 *a*; 1887: 194 *a*), adult male, mounted skin and skull. Loc.: Dabocrom, Côte d’Or [Ghana]. Leg.: H.S. Pel, June.

Paralectotype: RMNH.MAM.19627.

The name *Sciurusebii* was first published by Pel (1851). This *Sciurusebii* is a junior synonym of *Xeruserythropus* Geoffroy, 1803 and is clearly a different species from *Sciurusebii* Temminck, 1853. As the latter had been used widely for more than a century, Husson and Holthuis (1968) proposed to suppress *Sciurusebii*Pel 1851 in order to save *Sciurusebii* Temminck, 1853. They also designated the lectotype (Husson and Holthuis 1968: 126). The ICZN in Opinion 945 ruled accordingly (ICZN 1970: 224).

***Eupetauruscinereus* Thomas, 1888.**

*Eupetaurustibetensis* Jackson, Helgen, Q. Li & Jiang in Jackson et al., 2021: 6.

Holotype, RMNH.MAM.19524 (Jentink 1887: 180 *b*; 1888: 2 *b*), subadult male, relaxed mount and skull. Loc.: Tibet.

In his description of *Eupetauruscinereus*Thomas (1888a: 257) nominated two specimens as “cotypes”, but he also mentioned two specimens in Naturalis in his description as belonging to this species, one from Tibet (RMNH.MAM.19524) and a melanistic form from Kashmir (RMNH.MAM.55222). These are excluded from the type series and the latter is identified as *Petauristapetauristaalbiventer* Gray, 1834. For discussion of these specimens see also Jentink (1890c: 143–144 on *E.cinereus*) and Anderson (1879: 279, on the melanistic specimen from Kashmir).

Jackson et al. (2021b: 6) describe the *Eupetaurus* from Tibet as a new species and select RMNH.MAM.19524 as the holotype. The paratype is in the NHM (NHMUK 23.11.10.2).

***Eutamiassibericussibericus* (Laxman, 1769)**

*Myoxuslineatus* von Siebold, 1824: 13.

Syntype, RMNH.MAM.59667, adult, sex unknown, flat skin. Loc.: Jesso [Hokkaido], Japan. Leg.: [Ph.F. von Siebold.]

Von Siebold (1824) did not state how many specimens he had in his possession; only one flat skin is present in the Naturalis collection. This specimen was undetected by Jentink, since it has not been listed in any of his catalogues and there are no Naturalis labels attached to this specimen. This is fortunate because this way it escaped the practice by former curators of removing the “untidy” original labels. Thus the original label by von Siebold is preserved, stating “Myoxus lineata e Jesso relata” and in a later hand “Tamias”.

***Euxeruserythropus* (É. Geoffroy Saint-Hilaire, 1803)**

*Sciurusebii* Pel, 1851: 161 (nomen oblitum).

Lectotype, RMNH.MAM.19622 (Jentink 1888: 37 *g*), adult male, mounted skin, skull in situ. Loc.: Rio Boutry, Côte d’Or [Ghana]. Leg.: H.S. Pel, 1842.

Paralectotypes: RMNH.MAM.19623–19625.

For remarks on the taxonomic status of this name, see the remarks for *Sciurusebii* Temminck, 1853 (above). Husson and Holthuis (1968: 126) designated the lectotype.

***Exillisciurusexilis* (Müller, 1838)**

*Sciurusexilis* Müller, 1838: 148.

Lectotype, RMNH.MAM.13318 (Jentink 1887: 190 *b*; 1888: 23 *b*), adult, sex unknown, relaxed mount and skull. Loc.: Mt. Singgalang, Sumatra, Indonesia. Leg.: S. Müller, [May–November 1834].

Paralectotype: RMNH.MAM.13319.

The collecting locality for RMNH.MAM.13319 is described by Müller (1838) as the mountainous regions of the Laut countries on Borneo. Medway (1977: 99) interpreted this as ‘Tanah Laut [Pleihari] district, South Kalimantan. Jentink (1888: 23) listed an additional specimen (cat. d; RMNH.MAM.13320) as a cotype. This specimen was collected by Schwaner and is not mentioned by Müller in the original publication, which is therefore excluded from the type series. The mandibles and cranium of RMNH.MAM.13318 do not belong together. According to Kris Helgen (in lit., 5 December 2018) the mandibles belong to *Sundasciurus*, probably *lowi*. Heaney (1985: 18) designated RMNH.MAM.13318 as the lectotype, being the specimen from Sumatra. He doubted the correctness of the locality and followed Medway (1977) in selecting Tanah Laut, South Kalimantan as the type locality. Until recently no other specimens have been recorded from Sumatra and most authors follow Heaney (see for instance Chasen and Kloss 1928: 44; Medway 1963: 108). However, in 2015 an animal was photographed in Batang Toru, North Sumatra and later identified as belonging to *Exilisciurusexilis* (Meijaard et al. 2018).

*Nannosciurusexilissordidus* Chasen & Kloss, 1928: 44.

Holotype, RMNH.MAM.10117, adult female, skin and skull. Loc.: Long Temelen, Borneo, Indonesia. Leg.: H.C. Siebers, Midden Oost Borneo Exp. 1925 (23), 26 August 1925. Ex: MZB (MZB 1189), 19 April 1950.

Chasen and Kloss (1928) examined 28 specimens; only the holotype is in Naturalis.

***Funambulussublineatus* (Waterhouse, 1838)**

*Sciurusdelessertii* Gervais, 1841: 51.

Syntype, RMNH.MAM.60459 (Jentink 1887: 193 *a*; 1888: 30 *a*), adult, sex unknown, mounted skin and skull. Loc.: Ghats Mts., Bengal, India. leg. A. Delessert, [1834–1839].

Syntype, RMNH.MAM.60460 (Jentink 1887: 25 *b*), adult, sex unknown, skull. Loc.: Bengal, India. Leg A. Delessert, [1834–1839].

Delessert returned from his collecting trip in India in 1839, well before the description of this new species by Gervais (1841) so these specimens were in the possession of Gervais.

The first publication of this new taxon was by Gervais in 1841. A more detailed description with plates was published in Delessert (1843: 18, pls 3, 4).

***Funisciuruscongicus* (Kuhl, 1820)**

*SciurusPoolii* Jentink, 1906: 139.

Holotype by monotypy, RMNH.MAM.26429 (formerly 66), female, skin and skull. Loc.: Stanley [Boyoma] Falls, Democratic Republic of Congo. Leg.: G.C.A. Pool, August 1905. Received from A.A.W. Hubrecht, 22 April 1906.

***Funisciuruspyrropusleucostigma* Temminck, 1853**

*Sciurusleucostigma* Temminck, 1853: 133.

Syntype, RMNH.MAM.26431 (Jentink 1887: 196 *b*; 1888: 35 *e*), adult male, mounted skin and skull. Loc.: Dabocrom, Côte d’Or [Ghana]. Leg.: H.S. Pel, May 1842.

Syntype, RMNH.MAM.26432 (Jentink 1888: 35 *f*), adult male, mounted skin, skull in situ. Loc.: Dabocrom, Côte d’Or [Ghana]. Leg.: H.S. Pel, May 1842.

Syntype, RMNH.MAM.26433 (Jentink 1888: 35 *g*), adult female, mounted skin, skull in situ. Loc.: Dabocrom, Côte d’Or [Ghana]. Leg.: H.S. Pel, May 1842.

Syntype, RMNH.MAM.26434 (Jentink 1887: 196 *a*; 1888: 35 *h*), adult female, mounted skin and skull. Loc.: Dabocrom, Côte d’Or [Ghana]. Leg.: H.S. Pel, February 1843.

Syntype, RMNH.MAM.26435 (Jentink 1887: 196 *b*; 1888: 35 *i*), adult female, mounted skin and skull. Loc.: Dabocrom, Côte d’Or [Ghana]. Leg.: H.S. Pel, 1842.

Syntype, RMNH.MAM.26436 (Jentink 1888: 35 *j*), adult male, mounted skin, skull in situ. Loc.: Dabocrom, Côte d’Or [Ghana]. Leg.: H.S. Pel, 1842.

Syntype, RMNH.MAM.26437 (Jentink 1888: 35 *k*), adult male, mounted skin, skull in situ. Loc.: Dabocrom, Côte d’Or [Ghana]. Leg.: H.S. Pel, 1842.

***Geosciurusinauris* (E.A.W. Zimmermann, 1780)**

*SciurusLevaillantii* Kuhl, 1820b: 67.

*Sciurussetosus* Smuts, 1832: 33.

Syntype for *S.setosus*, possible syntype for *S.levaillantii*, RMNH.MAM.26465 (Jentink 1887: 198 *a*; 1888: 38 *a*), adult female, mounted skin and skull. Loc.: “Cap”, South Africa.

Syntype for *S.setosus*, RMNH.MAM.26466 (Jentink 1887: 198 *b*; 1888: 38 *b*), juvenile female, mounted skin and skull. Loc.: “Cap”, South Africa. Leg.: H.B. van Horstok, [1826–1835].

Smuts (1832) based his work on the collection in Leiden and both specimens were in all likelihood available to him. Kuhl (1820b) referred to specimens in several collections, including Temminck’s cabinet, which became part of the Naturalis collection. RMNH.MAM.26465 could be the specimen from Temminck’s collection, although it is not indicated as such on the label. RMNH.MAM.26266 was not available to Kuhl. Both names were written by Temminck on the bottom of the stand, so we include them in the type series for *setosus* Smuts, 1832; only RMNH.MAM.26465 could also be the syntype for *levaillantii* Kuhl, 1820.

***Heliosciurusgambianusmulticolor* (Rüppell, 1835)**

*Sciurusmulticolor* Rüppell, 1835: 38.

Paralectotype, RMNH.MAM.26356 (Jentink 1887: 196 *b*; 1888: 34 *f*), adult female, mounted skin and skull. Loc.: Ethiopia. Leg.: E. Rüppell.

Paralectotype: RMNH.MAM.26357.

Mertens (1925: 27) designated a specimen in the SMF (SMF 4329) the lectotype.

***Heliosciuruspunctatuspunctatus* (Temminck, 1853)**

*Sciuruspunctatus* Temminck, 1853: 138.

Syntype, RMNH.MAM.26366 (Jentink 1887: 196 *b*; 1888: 33 *d*), adult female, mounted skin and skull. Loc.: Dabocrom, Côte d’Or [Ghana]. Leg.: H.S. Pel, June 1843.

Syntype, RMNH.MAM.26367 (Jentink 1887: 196 *c*; 1888: 33 *e*), adult female, mounted skin and skull. Loc.: Dabocrom, Côte d’Or [Ghana]. Leg.: H.S. Pel, June 1843.

Syntype, RMNH.MAM.26368 (Jentink 1887: 196 *d*; 1888: 33 *f*), adult male, mounted skin and skull. Loc.: Dabocrom, Côte d’Or [Ghana]. Leg.: H.S. Pel.

Syntype, RMNH.MAM.26369 (Jentink 1887: 196 *e*; 1888: 33 *g*), adult female, mounted skin and skull. Loc.: Rio Boutry, Côte d’Or [Ghana]. Leg.: H.S. Pel.

Syntype, RMNH.MAM.26370 (Jentink 1888: 33 *h*), adult female, mounted skin, skull in situ. Loc.: Rio Boutry, Côte d’Or [Ghana]. Leg.: H.S. Pel, June 1843.

***Heliosciurusrufobrachiummaculatus* (Temminck, 1853)**

*Sciurusmaculatus* Temminck, 1853: 130.

Syntype, RMNH.MAM.26381 (Jentink 1887: 195 *b*; 1888: 32 *i*), adult male, mounted skin and skull. Loc.: Rio Boutry, Côte d’Or [Ghana]. Leg.: H.S. Pel.

Syntype, RMNH.MAM.26382 (Jentink 1888: 32 *j*), adult male, mounted skin, skull in situ. Loc.: Rio Boutry, Côte d’Or [Ghana]. Leg.: H.S. Pel.

Syntype, RMNH.MAM.26383 (Jentink 1887: 195 *c*; 1888: 32 *k*), adult female, mounted skin and skull. Loc.: Rio Boutry, Côte d’Or [Ghana]. Leg.: H.S. Pel.

Syntype, RMNH.MAM.26384 (Jentink 1887: 195 *d*; 1888: 33 *l*), adult male, mounted skin and skull. Loc.: Saccondee, Côte d’Or [Ghana]. Leg.: H.S. Pel, 25 April 1842.

Syntype, RMNH.MAM.26385 (Jentink 1888: 33 *m*), juvenile female, mounted skin, skull in situ. Loc.: Rio Boutry, Côte d’Or [Ghana]. Leg.: H.S. Pel.

Syntype, RMNH.MAM.26395, adult male, skull. Loc.: Dabocrom, [Ghana]. Leg.: H.S. Pel, February 1843.

***Hylopetesalbonigeralboniger* (Hodgson, 1836)**

*Sciuropterusalboniger* Hodgson, 1836: 231.

Paralectotype, RMNH.MAM.63846 (Jentink 1888: 5 *b*), unknown, mounted skin, skull in situ. Loc.: Nepal. Leg.: B.H. Hodgson.

Paralectotype: RMNH.MAM.63847.

According to Jentink (1888: 5) RMNH.MAM.63846 is a male. On the stand, however, female is written in Temminck’s hand. The specimen listed by Jentink (1888: 5) as cat. *c* is no longer present in Naturalis.

Naturalis holds another specimen (RMNH.MAM.63716) from Hodgson collected in “Sikkim/Tibet”. As Hodgson only mentioned specimens from Nepal in his description, we do not include this specimen in the type series.

Thomas (1918: 371) designated the lectotype in the NHM (NHMUK 1843.1.12.49).

***Hylopetesbartelsi* (Chasen, 1939)**

*Petinomysbartelsi* Chasen, 1939a: 185.

Syntype, RMNH.MAM.15680, adult male, skin and skull. Loc.: Mt. Pangrango, Tjilondong, Java, Indonesia. Leg.: M. Bartels, 19 April 1938. Ex: Bartels (no. 2000).

Syntype, RMNH.MAM.15539, adult female, skin, skull missing. Loc.: Mt. Pangrango, Java, Indonesia. Leg.: M. Bartels, 2 September 1902. Ex: Bartels.

***Hylopetesplatyurus* (Jentink, 1890)**

*Sciuropterusplatyurus* Jentink, 1890d: 145.

Holotype, RMNH.MAM.13315, adult female, relaxed mount and skull. Loc.: Deli, Sumatra, Indonesia. Leg.: B. Hagen. Received 1889.

***Hylopetesspadiceussumatrae* Sody, 1949**

*Hylopetis [sic] sagitta sumatrae* Sody, 1949a: 71.

Holotype, RMNH.MAM.9844, adult male, skin and skull. Loc.: Radelong, Sumatra, Indonesia. Leg.: Madzoed, 14 July 1936. Ex: MZB (MZB 3111), 12 January 1950.

***Hylopeteswinstoni* (Sody, 1949)**

*Iomyswinstoni* Sody, 1949a: 75.

Holotype, RMNH.MAM.9845, adult male, skin and skull. Loc.: Baleg, Atjeh, Sumatra, Indonesia. Leg.: Madzoed, 19 August 1930. Ex: MZB (MZB 3112), 12 January 1950.

***Lariscusinsignisinsignis* (F. Cuvier, 1821)**

*Lariscusinsignisdiversoides* Sody, 1949a: 114.

Holotype, RMNH.MAM.9839, adult male, skin and skull. Loc.: Sanggul, Sumatra, Indonesia. Leg.: J.J. Menden, 11 August 1936. Ex: MZB, 12 January 1950.

Sody (1949a) examined six specimens; only the holotype is in Naturalis.

*Lariscusinsignisatchinensis* Sody, 1949a: 115.

Holotype, RMNH.MAM.9841, adult male, skin and skull. Loc.: Atang Putar, Atjeh, Sumatra, Indonesia. Leg.: A. Hoogerwerf, 11 April 1937. Ex: MZB (MZB 472/37), 12 January 1950.

Paratypes: RMNH.MAM.24456, 24457.

For a preliminary report and map of this expedition, see Hoogerwerf (1939).

***Lariscusinsignisjavanus* Thomas & Wroughton, 1909**

*Lariscusinsignismurianus* Sody, 1937: 219.

Holotype, RMNH.MAM.13374, adult male, skin and skull. Loc.: Kampong Semliro, Gunung Muriah 800 m.), Java, Indonesia. Leg.: H.J.V. Sody, 13 December 1928 (M. 6).

Paratypes: RMNH.MAM.24519, 24520.

***Lariscusobscurusauroreus* Sody, 1949**

*Lariscusinsignisauroreus* Sody, 1949a: 114.

Holotype, RMNH.MAM.9840, adult female, skin and skull. Loc.: North Pagi Isl., Indonesia. Leg.: J.J. Menden, 23 January 1935. Ex: MZB (MZB 6363; 16/35), 12 January 1950.

Sody (1949a) examined ten specimens; only the holotype is in Naturalis.

***Marmotahimalayanahimalayana* (Hodgson, 1841)**

*Arctomyshimalayanus* Hodgson, 1841c: 777.

Syntype, RMNH.MAM.39323 (Jentink 1888: 43 *b*), adult, sex unknown, mounted skin, skull in situ. Loc.: Tibet. Leg.: B.H. Hodgson.

***Marmotahimalayanarobusta* (Milne-Edwards, 1872)**

*Arctomysrobustus* Milne-Edwards, 1872: 92.

Syntype, RMNH.MAM.39324 (Jentink 1887: 201 *a*; 1888: 43 *a*), adult male, mounted skin and skull. Loc.: Moupin (Baoxing), China. Leg.: J.P.A. David, July 1869. Ex: MNHN, 1875.

Rode (1945: 25) listed a holotype (MNHN 1870-43) and paratype (MNHN 1870-538) in the MNHN, which should be syntypes since Milne-Edwards does not specify any type specimens.

***Nannosciurusmelanotismelanotis* (Müller, 1841)**

*Sciurusmelanotis* Müller, 1840a: 35 (nomen nudum).

*Sciurusmelanotis* Müller, 1841: pl. 14 figs 4–7.

*Sciurussoricinus* Waterhouse, 1838: 46 (nomen nudum).

*Sciurussoricinus* “Waterhouse” Anderson, 1879: 265.

Lectotype for *S.melanotis* and syntype for *S.soricinus*, RMNH.MAM.13338 (Jentink 1887: 191 *b*; 1888: 25 *h*), adult female, relaxed mount, skull. Loc.: Java, Indonesia. Leg.: S. Müller.

Paralectotype for *S.melanotis* and syntype for *S.soricinus*, RMNH.MAM.13337 (Jentink 1888: 25 *g*), adult male, relaxed mount, skull in situ. Loc.: Java, Indonesia. Leg.: S. Müller.

Paralectotype for *S.melanotis* and syntype for *S.soricinus*, RMNH.MAM.39353 (Jentink 1887: 191 *d*) adult, sex unknown, cranium. Loc.: Java, Indonesia. Leg.: H. Boie and H.C. Macklot, [1826–1827](missing).

Paralectotype for *S.melanotis* and syntype for *S.soricinus*, RMNH.MAM.39354 (Jentink 1887: 191 *e*) adult, sex unknown, cranium. Loc.: Java, Indonesia. Leg.: H. Boie and H.C. Macklot, [1826–1827].

Paralectotype for *S.melanotis* and syntype for *S.soricinus*, RMNH.MAM.59653 (Jentink 1888: 25 *c*), adult male, relaxed mount, skull in situ. Loc.: Borneo, Indonesia. Leg.: S. Müller, [28 July 1836–17 December 1836]. Received 1837. (= *Nannosciurusmelanotisborneanus*).

Paralectotype for *S.melanotis* and syntype for *S.soricinus*, RMNH.MAM.13344 (Jentink 1888: 25 *d*), adult male, relaxed mount, skull. Loc.: Pontianak, Borneo, Indonesia. Leg.: P.-M. Diard, [1826]. (= *Nannosciurusmelanotisborneanus*).

Paralectotype for *S.melanotis* and syntype for *S.soricinus*, RMNH.MAM.39352 (Jentink 1887: 191 *c*), adult female, cranium. Loc.: Borneo, Indonesia. Leg.: P.-M. Diard, [1826]. (= *Nannosciurusmelanotisborneanus*).

Paralectotype for *S.melanotis* and syntype for *S.soricinus*, RMNH.MAM.59652 (Jentink 1888: 25 *a*), adult female, relaxed mount, skull in situ. Loc.: Padang, Sumatra, Indonesia. Leg.: S. Müller, [July 1833–December 1835]. (= *Nannosciurusmelanotispulcher*).

The name *Sciurusmelanotis* was first published by Müller (1840a: 35) without a description; he only gave locations and a description of the habitat. Koprowski et al. (2020: 588) considered this a valid publication and therefore dated this name in 1840. On 11 October 1841 the plate for this taxon was published (Müller 1841: pl. 14), accompanied by the scientific name, followed by a formal description in the issue of March 1844 (see for publication dates Husson and Holthuis 1955: 22–23).

This species was first described under the name *Sciurussoricinus* in 1838 by Waterhouse (1838: 46), publishing a manuscript name by Temminck. Waterhouse was uncertain about his reference to Temminck and we have not been able to find any publication by Temminck of this name. Waterhouse provided no description and only gave a location: “Java” (Heaney 1985: 25; Thorington and Hoffmann 2005: 784). The first valid publication of this name is by Anderson (1879: 265), who gave a formal description and referred to the specimen listed by Waterhouse in the NHM and the specimens in Müller (1841) and Müller and Schlegel (1844).

Heaney (1985: 25), who did not mention the first publication of Müller (1840a), designated RMNH.MAM.13338 as the lectotype based on the assumption that this specimen was depicted in Müller (1841) and followed Lyon (1906: 52), who restricted the type locality to Java.

Jentink (1883a, 1887, 1888) in his catalogues of the Naturalis mammal collection inconsistently listed specimens as types for *melanotis*. He omitted specimens collected by Müller himself and even lists specimens collected by Schwaner, who arrived in Indonesia in 1842, well after the publication. According to Jentink (1887: 191) the skull depicted on the plate (figs 6, 7) is RMNH.MAM.39354 (cat. *e*) and the two depicted specimens are RMNH.MAM.13337 and 13338. However, these specimens are both from Java and one is a female.

Largen (1985) listed a possible paralectotype for *Sciurusmelanotis* (LIVCM-D.392) in the World Museum Liverpool.

***Nannosciurusmelanotisborneanus* Lyon, 1906.**

*Nannosciurusmelanotispallidus* Chasen & Kloss, 1928: 43.

Holotype, RMNH.MAM.10116, adult male, skin and skull. Loc.: Long Poehoes, Borneo, Indonesia. Leg.: H.C. Siebers, Midden-Oost Borneo Expeditie 1925, 10 August 1925 (10). Ex: MZB (MZB 1178), 19 April 1950.

Chasen and Kloss (1928) examined four specimens; only the holotype is in Naturalis.

***Paraxerusboehmiemini* (Stuhlmann, 1894)**

*Sciurusemini* “Matschie” Stuhlmann, 1894: 320.

Syntype, RMNH.MAM.26455 (Jentink 1888: 36 *a*), adult female, mounted skin and skull. Loc.: Gadda [Uele River], Monbuttu [Mangbetu], Democratic Republic of Congo. Leg.: Emin Pasha [E.C.O.T. Schnitzer], 29 March 1884. Ex: NHM, March 1888.

***Paraxeruspoensis* (A. Smith, 1830)**

*Sciurusmusculinus* Temminck, 1853: 142.

Syntype, RMNH.MAM.26407 (Jentink 1888: 34 *d*), adult male, mounted skin, skull in situ. Loc.: Rio Boutry, Côte d’Or [Ghana]. Leg.: H.S. Pel, 1842.

Syntype, RMNH.MAM.26408 (Jentink 1888: 34 *e*), adult male, mounted skin, skull in situ. Loc.: Rio Boutry, Côte d’Or [Ghana]. Leg.: H.S. Pel, 1842.

Syntype, RMNH.MAM.26409 (Jentink 1887: 196 *b*; 1888: 34 *f*), adult female, mounted skin and skull. Loc.: Rio Boutry, Côte d’Or [Ghana]. Leg.: H.S. Pel, 1842.

Syntype, RMNH.MAM.26410 (Jentink 1888: 35 *g*), subdult, sex unknown, mounted skin, skull in situ. Loc.: Rio Boutry, Côte d’Or [Ghana]. Leg.: H.S. Pel, 1842.

Syntype, RMNH.MAM.26411 (Jentink 1888: 35 *h*), subadult male, mounted skin, skull in situ. Loc.: Rio Boutry, Côte d’Or [Ghana]. Leg.: H.S. Pel, 1842.

***Petauristaeleganselegans* (Temminck, 1836)**

*Pteromyselegans* Temminck, 1836: xii.

Syntype, RMNH.MAM.64144 (Jentink 1888: 4 *f*), adult, sex unknown, relaxed mount, skull extracted but not in collection. Loc.: Nusa Kambangan, Indonesia. Leg.: C.L. Blume, [3 November–1 December 1824].

Temminck (1836) only referred to the island Nusa Kambangan as the origin of this species, even explicitly stating that on Java itself this species had not been found. He did not specify how many specimens were available, but all types listed in Jentink (1887: 182; 1888: 4) from Java are here excluded from the type series. Temminck must have overlooked these specimens collected by Kuhl and Van Hasselt in 1820–1821 (RMNH.MAM.13310 and 60455) and by Müller in 1826–1832 (RMNH.MAM.60454).

RMNH.MAM.64144 was stored together with another relaxed mount collected by F.W. Junghuhn in 1864 (RMNH.MAM.64143) and the labels of both specimens had become disassociated, making identification of the correct specimen problematic. Fortunately, the collection holds another specimen from Junghuhn, still mounted and with the correct label (RMNH.MAM.64145). Comparing the specimens, in particular the type of glass eyes used, made it possible to identify the type specimen.

Blume (also written as Blüme) collected on Nusa Kambangan between 3 November and 1 December 1824 (Van Steenis-Kruseman 1950: 64).

***Petauristaeleganssumatrana* Kloss, 1921**

*Petauristapunctatasumatrana* Kloss, 1921a: 230, pl. III.

Holotype, RMNH.MAM.60452, adult female, skin and skull. Loc.: Padang Highlands, West Sumatra (probably near Fort de Kock), Indonesia, 29 May 1918. Leg.: E. Jacobson (398).

***Petauristagrandis* (Swinhoe, 1863)**

*Pteromysgrandis* Swinhoe, 1863: 358.

Syntype, RMNH.MAM.39325 (Jentink 1887: 181 *a*), adult, sex unknown, skull. Loc.: Formosa [Taiwan]. Leg.: R. Swinhoe. Received 1863.

Syntype, RMNH.MAM.39326 (Jentink 1887: 181 *b*; 1888: 3 *a*), adult, sex unknown, mounted skin and skull. Loc.: Formosa [Taiwan]. Leg.: R. Swinhoe. Ex: G.A. Frank, 1877.

Syntype, RMNH.MAM.39327 (Jentink 1888: 3 *b*), adult, sex unknown, mounted skin, skull in situ. Loc.: Formosa [Taiwan]. Leg.: R. Swinhoe, April 1862.

Syntype, RMNH.MAM.39328 (Jentink 1888: 3 *c*), juvenile, sex unknown, mounted skin, skull in situ. Loc.: Formosa [Taiwan]. Leg.: R. Swinhoe, April 1862.

Syntype, RMNH.MAM.39329 (Jentink 1888: 3 *d*), adult, sex unknown, mounted skin, skull in situ. Loc.: Formosa [Taiwan]. Leg.: R. Swinhoe. Ex: G.A. Frank, 1877.

Syntype, RMNH.MAM.39330 (Jentink 1888: 3 *e*), juvenile, sex unknown, mounted skin, skull in situ. Loc.: Formosa [Taiwan]. Leg.: R. Swinhoe. Ex: G.A. Frank, 1877.

The two specimens with the collecting date of April 1862 are clearly not the animals Swinhoe mentioned in his description of this new species. However, they were in his possession during the writing of the publication, so are part of the type series. When he acquired the other specimens is not known, they could be (in part) the original male, female, and juvenile. The NHM online database (https://data.nhm.ac.uk/object/42e7e09c-c5b9-40e3-9929-2dc28af368e7/1738108800000) lists one syntype (NMHUK 1862.12.24.11), a juvenile.

***Petauristaleucogenysleucogenys* (Temminck, 1827)**

*Pteromysleucogenys* Temminck, 1827a: xxvii.

Syntype, RMNH.MAM.13311 (Jentink 1887: 181 *a*; 1888: 4 *a*), adult, sex unknown, mounted skin and skull. Loc.: Japan. Leg.: H. Bürger.

Syntype, RMNH.MAM.13312 (Jentink 1887: 182 *b*; 1888: 4 *b*), juvenile, sex unknown, mounted (faded) skin and cranium. Loc.: Japan. Leg.: H. Bürger.

In his first very short introduction of this new species Temminck (1827a) gave no information of their origin. In a later publication, Temminck (1844) referred to specimens collected by Blomhof, von Siebold, and Bürger and gave as origin “les provinces de Figo et Fiuga”. The type locality has been restricted to “Higo, Kiusiu” [Kumamoto Prefecture, Kyushu, Japan] by Kuroda (1938: 50; not seen).

***Petauristamagnificus* (Hodgson, 1836)**

*Sciuropterus Magnificus* Hodgson, 1836: 231.

Syntype, RMNH.MAM.60453 (Jentink 1888: 2 *a*), adult male, mounted skin, skull in situ. Loc.: Nepal. Leg.: B.H. Hodgson.

***Petauristapetauristainterceptio* Sody, 1949**

*Petauristapetauristainterceptio* Sody, 1949a: 69.

Holotype, RMNH.MAM.9847, adult male, skin and skull. Loc.: Tjerimai [Ciremai], Cheriben, Java, Indonesia. Leg.: J.J. Menden, 11 July 1931. Ex: MZB (MZB 2666), 12 January 1950.

Sody (1949a) used 15 specimens for his description; only the holotype is in Naturalis.

*Petauristapetauristarufipes* Sody, 1949a: 68.

*Petauristapetauristarufipes* Sody, 1949a: 68.

Holotype, RMNH.MAM.9848, adult female, skin and skull. Loc.: Palembang, Kluang [Keluang], Sumatra, Indonesia. Leg.: Soekarno, 22 July 1933. Ex: MZB (MZB 5941; 1/33), 12 January 1950.

Sody (1949a) referred to three specimens in his description; only the holotype is in Naturalis.

***Petinomyshageni* (Jentink, 1889)**

*Sciuropterushageni* Jentink, 1889 [“1888”]: 26.

Syntype, RMNH.MAM.13313 (Jentink 1888: 6 *a*), adult, sex unknown, relaxed mount and skull. Loc.: Deli, Sumatra, Indonesia. Leg.: B. Hagen, 28 August 1887 (63). Received June 1888.

Syntype, RMNH.MAM.13314 (Jentink 1888: 6 *b*), adult, sex unknown, relaxed mount and skull. Loc. Deli, Sumatra, Indonesia. Leg.: B. Hagen, 1887.

Generally the date for this publication is given as 1888 (Thorington and Hoffmann 2005: 773; Koprowski et al. 2020: 604), based on the imprint date (November 1888). However this article (Note VI) was published in the first instalment of Volume XI of the Notes issued in January 1889 (Dickinson 2005).

*Petinomyshageniouwensi* Sody, 1949a: 74.

Holotype, RMNH.MAM.9846, adult female, skin and skull. Loc.: Koeboe, Pontianak, Borneo, Indonesia. Leg.: W. Brautigam, June 1917. Ex: MZB (MZB 3759), 12 January 1950.

***Petinomyssetosus* (Temminck, 1844)**

Pteromys (sciuropterus) setosus Temminck, 1844: 48.

Syntype, RMNH.MAM.13316 (Jentink 1887: 183 *a*; 1888: 6 *a*), adult female, relaxed mount and skull. Loc.: Padang, Sumatra, Indonesia. Leg.: L. Horner, [1835–1838].

Ludwig Horner arrived in Sumatra in 1835 and collected there (and other islands) until his death in 1838.

***Petinomysvordermanni* (Jentink, 1890)**

*Sciuropterusvordermanni* Jentink, 1890e: 150.

Holotype, RMNH.MAM.13317, adult male, relaxed mount and skull. Loc.: Billiton, Indonesia. Leg.: A. Vorderman, June 1888. Received 1890.

***Prosciurillusleucomusleucomus* (Müller & Schlegel, 1844)**

*Sciurusleucomus* Müller & Schlegel, 1844: 87.

Lectotype, RMNH.MAM.13344 (Jentink 1888: 24 *b*), male, mounted skin and skull. Loc.: Kema, Sulawesi, Indonesia. Leg.: E.A. Forsten, 1840.

Paralectotypes: RMNH.MAM.13343, 54910–54915, 54918 (= *Sciurusleucomusoccidentalis*).

Müller and Schlegel (1844) did not state how many specimens they had before them. The skulls of RMNH.MAM.13343 and 13344 were extracted at a later date. Jentink (1887: 191) also listed two skeletons and four separate skulls collected by Forsten as types. Although Müller and Schlegel (1844) did not include skeletal material in their description, they had these specimens available when writing their descriptions, and hence they are included in the type series here.

Forsten arrived in Manado in northeast Sulawesi on 22 March 1840; on 15 April he made his headquarters at Tondano, from where he explored Minahasa (Manado) District in the northeastern tip of the island. He arrived back in Manado on 24 April 1841, departed from Kema on 14 June, and landed in Ternate on 19 June. On 9 September 1841 he proceeded to Gorontalo in NE Sulawesi, where he arrived on 18 September. From there, he travelled further west along the coast as far as Paguat, and returned to Gorontalo on 14 November. He arrived back at Kema on 28 November and worked again in Minahasa District, until he finally left Sulawesi on 14 April 1842 (Veth 1879: 98, 107; Van Steenis-Kruseman 1950: 179; and Forsten’s unpublished diary in Naturalis archives (not catalogued)).

RMNH.MAM.13343 must be the animal collected on 6 April 1840 near Manado. In Forsten’s diary the entry for that day relates that his hunter brought “a Sciurus which I believe to be new”. In the same diary, Forsten recorded having collected a *Sciurus* at Pagowat (Paguat) during the week of 5–12 November 1841. This is almost certainly RMNH.MAM.54918, the pedestal of which reads “Sciurus leucomus Forst Pagowat Célebes” in Temminck’s handwriting, though Forsten was not mentioned as the collector. Jentink (1888: 25) must have overlooked Forsten’s diary note and so did not mention this specimen as a type; it is included in the type series here.

Musser et al. (2010: 62) designated the lectotype. In the subsequent details on the type series, these authors accidentally listed RMNH.MAM.13343 as the lectotype (p. 63).

***Prosciurillusleucomusoccidentalis* Meyer, 1898**

*Sciurusleucomusoccidentalis* Meyer, 1898: 2.

Paralectotype, RMNH.MAM.46075, (formerly 39401) (Jentink 1887: 191 *h*; 1888: 25 *l*), male, mounted skin and skull. Loc.: Panibi near Gorontalo, Sulawesi, Indonesia. Leg.: C.B.H. von Rosenberg, [7–15] September 1863.

Paralectotypes: RMNH.MAM.54916–54920 (skull RMNH.MAM.54918 is missing).

Meyer (1896: 25–26) discussed the differences in colouration between specimens of *Sciurusleucomus* from various localities in NE Sulawesi. He extensively examined the material in Naturalis and tentatively distinguished the animals from Gorontalo from the material collected in the Minahasa district. Meyer used the catalogue numbers given by Jentink (1883a: 130), including the corrections that Jentink had pointed out to him. He excluded the Naturalis specimens for which no precise locality was known. Later, after having received additional material from Sulawesi, Meyer (1898: 2) described the populations from the surroundings of Gorontalo as the subspecies *S.l.occidentalis*. Apart from the two specimens he had recently received, Meyer (1898) again referred to the Naturalis material which he had discussed earlier, these specimens therefore are part of the type series.

For the dates and localities of Von Rosenberg’s collecting trips in the surroundings of Gorontalo, see von Rosenberg (1865: 34–37, 60) and his notes preserved in the Naturalis archives (inv. 255.486).

The lectotype is one of the two specimens from Gorontalo in the MTKD (SNSD B 168), which Feiler (1998: 407) erroneously published as “Holotypus”. This does not constitute a valid lectotype designation (ICZN art. 74.5). However, this lectotype designation was confirmed and validated by Musser et al. (2010: 75–77).

***Prosciurillusmurinus* (Müller & Schlegel, 1844)**

*Sciurusmurinus* Müller & Schlegel, 1844: 87.

*Sciurusumbrinus* “Temminck” Jentink, 1883a: 126 (in synonymy).

Lectotype, RMNH.MAM.13213 (Jentink 1887: 190 *a*; 1888: 22 *a*), female, mounted skin and cranium. Loc.: North[east] Sulawesi, Indonesia. Leg.: E.A. Forsten, [1840–1842].

Paralectotypes: RMNH.MAM.13214, 13219.

For Forsten’s stay in NE Sulawesi and for the inclusion of the separate skull in the type series, see under *P.leucomus*; see also Musser et al. (2010: 134–135), who designated the lectotype.

Jentink (1883a: 126) listed two of these specimens (RMNH.MAM.13213–13214) as the types of the manuscript name *umbrinus* by Temminck in the synonymy of *Sciurusmurinus*. As far as we could establish, this name has never been validly used since and therefore is not available.

*Sciurillusmurinusgriseus* Sody, 1949a: 77.

Holotype, RMNH.MAM.9827, female, skin, skull missing. Loc.: Bumbulan, Sulawesi, Indonesia. Leg.: J.J. Menden, 24 October 1939. Ex: MZB (MZB 5974; 220/39), 12 January 1950.

Sody (1949a: 78) also described the skull of RMNH.MAM.9827, which was still present in Naturalis in 1951, when it was studied and photographed by Tate (Musser et al. 2010: 157); however, it is now missing.

***Prosciurillusrosenbergii* (Jentink, 1879)**

*SciurusRosenbergii* Jentink, 1879b: 37.

Lectotype, RMNH.MAM.13362 (Jentink 1888: 24 *l*), female, mounted skin and skull. Loc.: Siau, Sangihe Islands, Indonesia. Leg.: D. Hoedt, 27 October 1865.

Paralectotypes: RMNH.MAM.13351–13361.

Jentink (1879b: 36) examined twelve specimens, all of which are still present in Naturalis. Five skulls were extracted in Jentink’s days, the others were taken out later.

Jentink (1888: 24) did not mention the locality for the specimens collected by von Rosenberg, now paralectotypes. Their labels read “Sanghir”, which in this case probably means one of the islands in the Sangihe Archipelago, not Sangihe Island in particular. Von Rosenberg did not visit the Sangihe Islands himself but sent local hunters to collect on the islands during September–November 1864, when he worked in NE Sulawesi; see his notes preserved in the Naturalis archives (inv. 255.486).

Musser et al. (2010: 123) designated the lectotype.

***Prosciurillusweberi* (Jentink, 1890)**

*Sciurusweberi* Jentink, 1890a: 115, pl. VIII, X figs 1–3.

Lectotype, RMNH.MAM.13342, adult female, mounted skin and skeleton. Loc.: Palopo, Luwu District, Sulawesi, Indonesia. Leg.: M. Weber, [February 1889].

Paralectotypes: ZMA.MAM.11327, 11328.

Jentink (1890a) based this species on three skins, two skeletons and one skull. The mammals collected by Weber were deposited in Naturalis and the ZMA, so after the merger of these two collections these specimens were reunited. According to Musser et al. (2010: 60) NHM skin NHMUK 1894.7.4.6 is also one of the specimens upon which Jentink’s original description of *Sciurusweberi* is based and thus also a paralectotype. This could be the skin belonging to skeleton ZMA.MAM.11327; see also Bergmans (2011: 837).

Weber visited the Luwu District in February 1889 (Weber 1890: vii, map III). Musser et al. (2010: 103) designated the lectotype.

***Protoxerusaubinniisalae* (Jentink, 1881)**

*Sciurussalae* Jentink, 1881: 63.

Syntype, RMNH.MAM.26396 (Jentink 1888: 32 *a*), adult male, mounted skin and skull. Loc.: Sofore-Place, Liberia. Leg.: J. Büttikofer and J.A. Sala, 15 May 1880.

Syntype, RMNH.MAM.26397 (Jentink 1887: 195 *a*; 1888: 32 *b*), adult male, mounted skin and skull. Loc.: Sofore-Place, Liberia. Leg.: J. Büttikofer and J.A. Sala, 16 May 1880.

Syntype, RMNH.MAM.26398 (Jentink 1887: 195 *b*; 1888: 32 *c*), adult male, mounted skin and skull. Loc.: Sofore-Place, Liberia. Leg.: J. Büttikofer and J.A. Sala, 25 May 1880.

Syntype, RMNH.MAM.26399 (Jentink 1888: 32 *d*), adult female, mounted skin, skull in situ. Loc.: Sofore-Place, Liberia. Leg.: J. Büttikofer and J.A. Sala, 05 July 1880.

Syntype, RMNH.MAM.26400 (Jentink 1888: 32 *e*), adult female, mounted skin, skull in situ. Loc.: Bavia, Liberia. Leg.: J. Büttikofer and J.A. Sala, 13 March 1880.

According to Jentink (1881: 63) the type locality is Liberia, St. Paul’s River (Bavia, Sofore-Place). Therefore, another specimen (RMNH.MAM.26401), with the same collectors but from Bendo, is excluded from the type series.

***Protoxerusstangeritemminckii* (Anderson, 1879)**

*Sciuruscaniceps* Temminck, 1853: 127.

*Sciurustemminckii* Anderson, 1879: 229 (nomen novum).

*Sciurustemminckii* Jentink, 1881: 65 (nomen novum).

Syntype, RMNH.MAM.26418 (Jentink 1888: 31 *c*), adult male, mounted skin and skull. Loc.: Dabocrom, Côte d’Or [Ghana]. Leg.: H.S. Pel, May 1849.

Syntype, RMNH.MAM.26419 (Jentink 1888: 31 *d*), adult female, mounted skin, skull in situ. Loc.: Dabocrom, Côte d’Or [Ghana]. Leg.: H.S. Pel, May 1849.

Syntype, RMNH.MAM.26420 (Jentink 1888: 31 *e*), adult female, mounted skin, skull in situ. Loc.: Dabocrom, Côte d’Or [Ghana]. Leg.: H.S. Pel, May 1849.

Syntype, RMNH.MAM.26421 (Jentink 1887: 194 *f*), adult, sex unknown, cranium. Loc.: Côte d’Or [Ghana]. Leg.: H.S. Pel.

This taxon was first described under the name *Sciuruscaniceps* Temminck, 1853. This name is preoccupied by *Sciuruscaniceps* Gray, 1842, so Anderson (1879: 229) proposed *Sciurustemminckii* as a new name. Apparently not aware of this act by Anderson, Jentink (1881: 65) also renamed *caniceps* as *Sciurustemminckii* for the same reason without reference to Anderson (1879).

Although dated 1878, the work by Anderson (1879) was not published until 1879 (Forbes 1881: 2).

***Pteromysmomonga* Temminck, 1844.**

Pteromys (sciuropterus) momonga Temminck, 1844: 47, tab. XIV.

*Pteromysmomoga* Temminck, 1844: tab. XIV (incorrect original spelling).

Syntype, RMNH.MAM.39334 (Jentink 1887: 182 *a*), adult, sex unknown, mounted skeleton. Loc.: Japan. Leg.: Ph.F. von Siebold.

Syntype, RMNH.MAM.39335 (Jentink 1887: 182 *b*), adult, sex unknown, mounted skeleton. Loc.: Japan.

Syntype, RMNH.MAM.39336 (Jentink 1887: 182 *d*), subadult, sex unknown, skull. Loc.: Japan.

Syntype, RMNH.MAM.39337 (Jentink 1887: 182 *e*), subadult, sex unknown, skull. Loc.: Japan.

Syntype, RMNH.MAM.39338 (Jentink 1887: 182 *c*; 1888: 5 *a*), subadult sex unknown, relaxed mount and skull. Loc.: Japan. Leg.: H. Bürger, 1834.

Syntype, RMNH.MAM.39339 (Jentink 1888: 5 *b*), adult, sex unknown, mounted skin, skull missing. Loc.: Japan. Leg.: H. Bürger, 1834.

Syntype, RMNH.MAM.39340 (Jentink 1888: 5 *c*), subadult, sex unknown, mounted skin, skull missing. Loc.: Japan. Leg.: H. Bürger, 1834.

Syntype, RMNH.MAM.39341 (Jentink 1888: 5 *d*), subadult, sex unknown, relaxed mount, skull missing. Loc.: Japan. Leg.: H. Bürger, 1834.

In the caption of the plate (Temminck 1844: tab. XIV) the name is misspelt *momoga*. Temminck (1844) himself corrected this in the footnote on page 48. This name is therefore not available (ICZN art. 32.4). According to Jentink (1888: 182) the skull of RMNH.MAM.39336 is the one figured on plate XIV. Although Temminck specifically only referred to the material received from Bürger in his description, Jentink listed them all as types and they were at Temminck’s disposal at the time of writing. Bürger was responsible for the shipment of von Siebold’s collection to Leiden. So we consider all the specimens to be syntypes.

The type locality was restricted by Kuroda (1938: 51; not seen) to Kyushu, Japan.

***Ratufaaffinisbaramensis* Bonhote, 1900**

*Ratufaephippiumbaramensis* Bonhote, 1900: 496.

Paratype, RMNH.MAM.31118, adult male, skin and skull. Loc.: Batu Sang, Baram River, Borneo, Indonesia. Leg.: C. Hose, March 1892.

The holotype is in the NHM (NHMUK 99.12.9.40), but Bonhote also refers to the specimens listed by Hose (1893: 44) including RMNH.MAM.31118.

***Ratufaaffiniscothurnata* Lyon, 1911**

*Ratufacothurnata* Lyon, 1911: 93.

Paratype, RMNH.MAM.31097, adult female, skin and skull. Loc.: Sempang River, Borneo, Indonesia. Leg.: W.L. Abbott (5544), 6 September 1907. Ex: NMNH (USNM 145380).

The holotype is in the NMNH (USNM 145378; Fisher and Ludwig 2012: 19). RMNH.MAM.31097 is included in the table with the material Lyon (1911) examined.

***Ratufaaffinisephippium* (Müller, 1838)**

*Sciurusephippium* Müller, 1838: 147.

Holotype by monotypy, RMNH.MAM.39344 (Jentink 1888: 15 *n*), adult male, mounted skin and skull. Loc.: Borneo, Indonesia. Leg.: S. Müller, [28 July–17 December] 1836.

Jentink (1888: 15) included two other specimens in the type series (RMNH.MAM.39345 and 39346, males collected by Diard on Borneo, 1829, subsequently identified as *Ratufaaffiniscothurnata*), probably based on a later publication (Müller 1839) in which they are depicted. However in his original description Müller did not refer to any other specimens than the one collected by himself. We therefore exclude them from the type series. In the manuscript of this catalogue by C. Smeenk we find the holotype listed as lectotype and the Diard specimens as paralectotypes without specification who designated the lectotype. Lyon (1911: 93) refers to “the type” in Naturalis without further specification, but it is clear that the Müller specimen is intended. Due to its ambiguity and the fact that there is only one specimen that fits the original description, we do not see this as a valid or necessary lectotypification.

Müller was on Borneo from July 28 to 17 December 1836.

*Sciurusalbiceps* Jentink, 1897: 55 (nec Desmarest) (misidentification).

Although Jentink (1897) attributed this name to Desmarest, these specimens clearly represent a different taxon from a different location. So *S.albiceps* Jentink, 1897 is a junior synonym for *Ratufaaffinis* (Thorington and Hoffmann 2005: 756), whereas *S.albiceps* Desmarest is a synonym of *Ratufabicolorbicolor* (see also Thorington and Hoffmann 2005: 756). We consider this a misidentification, therefore this name is not available (ICZN art.49) and RMNH.MAM.31102–31110, 59745 and 63789 are not syntypes.

***Ratufavittata* Lyon, 1911: 94.**

Paratype, RMNH.MAM.31100, male, skin and skull. Loc.: Pulau Laut, Borneo, Indonesia. Leg.: W.L. Abbott (5633), 20 December 1907. Ex: NMNH (USNM 151759).

Lyon (1911) examined five specimens; the holotype is in NMNH (USNM 151758; Fisher and Ludwig 2012: 20).

***Ratufavittatula* Lyon, 1911: 95.**

Paratype, RMNH.MAM.31101, female, skin and skull. Loc.: Pulau Sebuku, Borneo, Indonesia. Leg.: W.L. Abbott (5722), 2 Januari 1908. Ex: NMNH (USNM 151764).

Lyon (1911) examined five specimens; the holotype is in the NMNH (USNM 151762; Fisher and Ludwig 2012: 20).

***Ratufabicolorgigantea* (McClelland, 1839)**

*SciurusMacruroides* Hodgson, 1841b: 220 (nomen nudum).

*SciurusMacruroides* Hodgson, 1849b: 775.

Paralectotype, RMNH.MAM.60461 (Jentink 1887:186 *a*; 1888:13 *a*), adult female, mounted skin and skull. Loc.: Himalaya. Leg.: B.H. Hodgson.

Hodgson (1841b: 220) first published this name without a description followed by a very short description later (Hodgson 1849b: 775). Thomas (1918: 371) designated the lectotype in the NHM (NHMUK 1843.1.12.76).

***Ratufabicolorpalliata* G.S. Miller, 1902**

*Sciurusbicolor var. Sondaica* Müller & Schlegel, 1844: 85.

Syntype, RMNH.MAM.63641 (Jentink 1888: 14 a), adult male, mounted skin, skull in situ. Loc.: Padang, Sumatra [Indonesia]. Leg.: S. Müller, [1833–1835]. Received 1836.

Syntype, RMNH.MAM.63642 (Jentink 1888: 14 b), adult male, mounted skin, skull in situ. Loc.: Padang, Sumatra [Indonesia]. Leg.: S. Müller, [1833–1835]. Received 1836.

Syntype, RMNH.MAM.63643 (Jentink 1888: 14 f), adult male, mounted skin, skull in situ. Loc.: Java [Indonesia]. Leg.: H. Kuhl and J.C. van Hasselt, [1820–1821].

Syntype, RMNH.MAM.63644 (Jentink 1888: 14 h), adult female, mounted skin, skull in situ. Loc.: Java. Leg.: C.G.C. Reinwardt [April 1816–March 1822].

Syntype, RMNH.MAM.63645 (Jentink 1888: 14 j), adult female, mounted skin, skull in situ. Loc.: Tjikao, Java. Leg.: H. Boie and H.C. Macklot [June 1826 – September 1827].

Syntype, RMNH.MAM.63646 (Jentink 1888: 14 k), juvenile, sex unknown, mounted skin, skull in situ. Loc.: Java [Indonesia]. Leg.: H. Kuhl and J.C. van Hasselt, [1820–1821].

Syntype, RMNH.MAM.63647 (Jentink 1888: 14 l), juvenile, sex unknown, mounted skin, skull in situ. Loc.: Java [Indonesia].

Syntype, RMNH.MAM.63648 (Jentink 1888: 14 m), juvenile, female, mounted skin, skull in situ. Loc.: Java [Indonesia]. Leg.: 1840.

Müller and Schlegel (1844: 85) introduced this new name of subspecific rank for the Javan and Sumatran form of *Sciurusbicolor* and gave a short description, but failed to mention any specimens. We have included specimens in the type series that agree with their description and have left out the specimens that were labelled *Sciurushypoleucos*.

Reinwardt visited Java several times between April 1816 and March 1822. Boie visited Java between June 1826 and September 1827. Müller visited West Sumatra from 1833–1835, so the date 1836 given by Jentink (1888: 14) must be the date the specimens arrived in Naturalis.

*Sciurusbicolor* var. *Indica* Müller & Schlegel, 1844: 85 (nec Erxleben, 1777).

Syntype, RMNH.MAM.63650, (Jentink 1888: 14 *a*), adult male, mounted skin (missing). Loc.: Malakka, Malaysia. Leg.: P.-M. Diard, 1828.

Syntype, RMNH.MAM.63649 (Jentink 1888: 14 *b*), adult female, mounted skin, skull extracted but not in collection. Loc.: Malakka, Malaysia. Leg.: P.-M. Diard, 1828.

***Rhinosciuruslaticaudatuslaticaudatus* (Müller, 1840)**

*Sciuruslaticaudatus* Müller, 1840: 34.

Syntype, RMNH.MAM.13370 (Jentink 1887: 197 *a*; 1888: 37 *a*), adult female, mounted skin and skeleton. Loc.: Pontianak, Borneo, Indonesia. Leg.: P. Diard, 1827.

Syntype, RMNH.MAM.13371, (Jentink 1887: 197 *a*; 1888: 37 *a*), adult female, mounted skin and skeleton. Loc.: Pontianak, Borneo, Indonesia. Leg.: P. Diard, 1827.

Syntype, RMNH.MAM.13372, (Jentink 1887: 197 *a*; 1888: 37 *a*), adult female, mounted skin, skull extracted but not in collection. Loc.: Pontianak, Borneo, Indonesia. Leg.: P. Diard, 1827.

Müller (1840a) based his short description on material collected by Diard. The species is later described and depicted in Müller and Schlegel (1841b: pl. 15 figs 1–3; 1844: 100). According to Jentink (1887: 197) the depicted skull belongs to the female RMNH.MAM.13370, which is also the depicted skin according to him; however, according to the index for the illustrations the depicted specimen is a male.

In the original manuscript of this catalogue this specimen was listed by Smeenk as a lectotype. We could not, however, find the lectotype designation and therefore list these specimens as a syntypes.

*Rhinosciuruslaticaudatussaturatus* Robinson & Kloss, 1919: 274

Holotype, RMNH.MAM.64146, adult female, study skin and skull. Loc.: Rimbo Pengadang, Barisan Mts, West Sumatera, Indonesia. Leg.: E. Jacobson, (65), 22 June 1916.

Robinson and Kloss (1919) examined two specimens for their description of this new species. Only the holotype is in Naturalis.

***Rubrisciurusrubriventer* (Müller & Schlegel, 1844)**

*Sciurusrubriventer* “Forsten” Müller & Schlegel, 1844: 86.

Lectotype, RMNH.MAM.13341 (Jentink 1888: 23 *a*), male, mounted skin, skull extracted but not in collection. Loc.: [Northeast] Sulawesi, Indonesia. Leg.: E.A. Forsten, [1840–1842].

Müller and Schlegel (1844) gave no indication of the number of specimens available to them. The listing by Jentink (1888: 23) of RMNH.MAM.13341 as “type de l’espèce’’ constitutes a lectotype designation. Musser et al. (2010: 33) erroneously list this specimen as holotype.

For Forsten’s stay in NE Sulawesi, see under *Prosciurillusleucomus*.

***Sciurusgranatensisvariabilis* I. Geoffroy St. Hilaire, 1832**

*Sciurus Lansbergei* “Temminck” Jentink, 1887: 11 (in synonymy).

Jentink (1887) published this manuscript name by Temminck in the synonymy of *Sciurusvariabilis*. We have found no subsequent use as a valid name, therefore this name is not available.

***Sciuruslis* Temminck, 1844**

*Sciuruslis* Temminck, 1844: 45, pl. XII figs 1, 2.

Syntype, RMNH.MAM.39347 (Jentink 1887: 188 *a*; 1888: 18 *a*), adult male, mounted skin and skull. Loc.: Jeddo, Japan. Leg.: Ph.F. von Siebold, [1823–1829].

Syntype, RMNH.MAM.39348 (Jentink 1887: 188 *b*; 1888: 18 *b*), adult female, mounted skin and skull. Loc.: Japan. Leg.: H. Bürger.

Syntype, RMNH.MAM.39349 (Jentink 1888: 18 c), adult, sex unknown, mounted skin, skull in situ. Loc.: Japan. Leg.: H. Bürger.

***Sciurusniger* Linnaeus, 1758**

*Sciurusanomalus* “Güldenstedt” Kuhl, 1820b: 68 (nec Gmelin, 1778).

According to Jentink (1883a: 95) RMNH.MAM.63817 (from Georgia, USA, acquired at the Bullock Museum auction) is the type of *Sciurusanomalus* Kuhl, 1820. Jentink later, under *Sciurussyriacus*, explained how the origin of this specimen was erroneously located in Georgia, Asia and the specimen, based on this origin, wrongly attributed to this species. As Kuhl had no intention to describe a new species and the application of *anomalus* by Kuhl is clearly a misidentification, we do not consider this an available name following ICZN art. 49.

***Sciurotamiasdavidianusdavidianus* (Milne-Edwards, 1867)**

*Sciurusdavidianus* Milne-Edwards, 1867: 196.

Syntype, RMNH.MAM.39343 (Jentink 1887: 189 *a*; 1888: 20 *a*), adult, sex unknown, mounted skin and skull. Loc.: ‘Environ de Pekin’, China. Leg.: J.P.A. David. Ex: MNHN, 1868.

***Sundasciurusphilippinensis* (Waterhouse, 1839)**

*Sciuruscagsi* Meyer, 1890: 600.

Paratype, RMNH.MAM.39342, adult, sex unknown, mounted skin and skeleton. Loc.: Davao, Philippines. Leg.: C.C. Platen, 28 August 1889. Ex: MTKD, 1891.

According to K. Helgen (pers. comm. June 2024) *Sciuruscagsi* is not a synonym of *Sundasciurusrabori* (as in Thorington and Hoffmann 2005: 788); it is a synonym of *S.philippinensis*.

***Sundasciurustenuismodestus* (Müller, 1840)**

*Sciurusmodestus* Müller, 1840a: 34.

Syntype, RMNH.MAM.13323 (Jentink 1888: 21 *c*), adult male, relaxed mount and skull. Loc.: [West] Sumatra, Indonesia. Leg.: S. Müller, [1833–1835]. Received 1837.

Syntype, RMNH.MAM.13324 (Jentink 1888: 21 *d*), juvenile male, relaxed mount and skull. Loc.: [West] Sumatra, Indonesia. Leg.: S. Müller, [1833–1835]. Received 1837.

Syntype, RMNH.MAM.13325 (Jentink 1887: 190 *a*; 1888: 22 *e*), adult male, relaxed mount and skull. Loc.: Mount Singgalang, Sumatra, Indonesia. Leg.: S. Müller, [May 1834–November 1834]. Received July 1837.

Syntype, RMNH.MAM.13326 (Jentink 1888: 22 *f*), adult female, relaxed mount and skull. Loc.: Padang, Sumatra, Indonesia. Leg.: S. Müller, [1833–1835]. Received July 1837.

Syntype, RMNH.MAM.13327 (Jentink 1888: 22 *g*), subadult, sex unknown, relaxed mount and skull. Loc.: [West] Sumatra, Indonesia. Leg.: S. Müller, [1833–1835]. Received July 1837.

Syntype, RMNH.MAM.13328 (Jentink 1888: 22 *h*), adult, sex unknown, relaxed mount, skull in situ. Loc.: [West] Sumatra, Indonesia. Leg.: S. Müller, [1833–1835]. Received July 1837.

Syntype, RMNH.MAM.13329 (Jentink 1888: 22 *i*), adult, sex unknown, relaxed mount and skull. Loc.: [West] Sumatra, Indonesia. Leg.: S. Müller, [1833–1835]. Received July 1837.

Syntype, RMNH.MAM.13330 (Jentink 1888: 22 *j*), adult, sex unknown, relaxed mount and skull. Loc.: West] Sumatra, Indonesia. Leg.: S. Müller, [1833–1835]. Received July 1837.

Syntype, RMNH.MAM.13331 (Jentink 1888: 22 *k*), adult, sex unknown, relaxed mount and skull. Loc.: Borneo, Indonesia. Leg.: S. Müller, [28 July 1836–17 December 1836]. (= *Sciurustenuisparvus*).

Jentink (1888a: 21) listed two more specimens collected in Malacca by Diard (RMNH.MAM.13321, 13322) and two from Borneo collected by Diard and Schwaner (RMNH.MAM.13332, 63717) as types. However, Müller (1840a: 34, 55) made no reference to specimens from Malacca and only referred to specimens collected by him or his travel companions.

The type series of *Sciurusmodestus* Müller, 1840 represents two taxa, *modestus* (RMNH.MAM.13323–13330) from Sumatra and *parvus* from Borneo (RMNH.MAM.13331). To fix the name *modestus* to the Sumatran form a lectotype should be designated.

Müller collected in West-Sumatra from 1833 until 1835, May to November 1834 he spent in the area around Mount Singgalang. From 28 July to 17 December 1836 Müller visited Borneo. Schwaner was in Borneo between 1843 and 1848.

*Sciurusminor* “Diard” Jentink, 1883a: 126 (in synonymy).

Jentink (1883a: 126) published this manuscript name by Diard in the synonymy of *Sundasciurustenuis*. We could not find any subsequent use validating this name. Therefore this name is not available and RMNH.MAM.63717 is not a type.

***Tamiopsmcclellandiileucotis* (Temminck, 1853)**

*Tamiasleucotis* Temminck, 1853: 252.

Syntype, RMNH.MAM.59664 (Jentink 1888: 31 *k*), male, mounted skin, skull in situ. Loc.: Malacca. Leg.: P.-M. Diard, “May”.

Syntype, RMNH.MAM.59665 (Jentink 1888: 31 *l*), male, mounted skin, skull in situ. Loc.: Malacca. Leg.: P.-M. Diard, “December”.

Diard collected in the Malay Peninsula in 1819 and 1824. The first trip was as assistant to Raffles and the collected material ended up in the Raffles collection. The specimens above were probably collected during Diard’s 1824 expedition (van Wingerden 2023; Dorai and Low 2021).

***Xerusrutilusrutilus* (Cretzschmar, 1826)**

*Sciurusrutilus* Cretzschmar, 1826: 59.

Paralectotype, RMNH.MAM.26467 (Jentink 1887: 197 *a*; 1888: 37 *a*), adult male, mounted skin and skull. Loc.: Abyssinia (Ethiopia). Leg.: E. Rüppell.

Paralectotypes: RMNH.MAM.26468, 26469.

Mertens (1925: 26) designated a specimen in the SMF (SMF 4328) as the lectotype.

#### ﻿﻿Gliridae Muirhead, 1819

***Glirulusjaponicus* (Schinz, 1845)**

*Myoxuselegans* Temminck, 1844: 52 [nec Ogilby, 1838].

*Myoxusjaponicus* Schinz, 1845: 530 (nomen novum).

*Myoxusjavanicus* Schinz, 1845: 530 (incorrect original spelling for *japonicus*).

*Myoxuslasiotis* Thomas, 1880: 40 (nomen novum).

Syntype, RMNH.MAM.39367 (Jentink 1887: 202 *a*; 1888: 45 *a*), adult, sex unknown, mounted skin and skull. Loc.: Awa province, Shikoku Island, Japan. Leg.: H. Bürger, 1834.

Syntype, RMNH.MAM.39368 (Jentink 1887: 202 *b*; 1888: 45 *b*), adult, sex unknown, mounted skin and skull. Loc.: Awa province, Shikoku Island, Japan. Leg.: H. Bürger, 1834.

Syntype, RMNH.MAM.39369 (Jentink 1888: 45 *c*), adult, sex unknown, mounted skin, skull in situ. Loc.: Awa province, Shikoku Island, Japan. Leg.: H. Bürger, 1834.

Schinz (1845: 530) renamed *Myoxuselegans* Temminck, 1844 as this name is preoccupied by *Myoxuselegans* (Ogilby, 1838). He gave the new name *Myoxusjavanicus* (Schinz 1845: 350), this is however conceived by later authors as a misprint for *japonicus* (Thomas 1905: 347).

Thomas (1880: 40) overlooked the Schinz name and renamed *Myoxuselegans* Temminck, 1844 as *Myoxuslasiotis*. In a later publication Thomas (1905: 347) accepted the validity and priority of Schinz’s name.

As proposed by Smeenk and Kaneko (2000: 36: case 3033) the ICZN (2001: opinion 1978) has ruled the conservation of *Glirulusjaponicus* (Schinz, 1845) as the correct spelling and that *elegans*, *javanicus*, and *lasiotis* are invalid synonyms.

***Graphiuruscrassicaudatus* (Jentink, 1888)**

*Clavigliscrassicaudatus* Jentink, 1888a: 41.

Holotype by monotypy, RMNH.MAM.26639 (Jentink 1888: 46 *a*), adult female, alcohol and cranium. Loc.: Hill-town, DuQueah river, Liberia. Leg.: J. Büttikofer; F.X. Stämpfli, 3 April 1887.

***Graphiuruskelleni* (Reuvens, 1890)**

*Eliomyskelleni*Reuvens 1890: 70.

Holotype, RMNH.MAM.26638, adult female, alcohol and skull. Loc.: Interieur de Mossamedes, Angola. Leg.: P.J. van der Kellen, Southwest Afrika Expedition 1884–1885, KNAG. Received 1888.

***Graphiurusnagtglasii* (Jentink, 1888)**

*Eliomysnagtglasii* Jentink, 1888a: 38.

Syntype, RMNH.MAM.26631 (Jentink 1888: 46 *a*), adult male, mounted skin and skull. Loc.: Côte d’Or [Ghana]. Leg.: C.J.M. Nagtglas. Received November 1862.

Syntype, RMNH.MAM.26632 (Jentink 1888: 46 *b*), adult, sex unknown, mounted skin, skull in situ. Loc.: Côte d’Or [Ghana]. Leg.: C.J.M. Nagtglas. Received 1862.

Syntype, RMNH.MAM.26633 (Jentink 1888: 46 *d*), adult female, alcohol. Loc.: Farmington river, Liberia. Leg.: F.X. Stämpfli, 1887.

Syntype, RMNH.MAM.26634 (Jentink 1888: 46 *e*), adult female, alcohol. Loc.: Farmington river, Liberia. Leg.: F.X. Stämpfli, 1887.

Syntype, RMNH.MAM.26635 (Jentink 1888: 46 *c*), adult male, alcohol and skull. Loc.: Hill-town, DuQueah river, Liberia. Leg.: J. Büttikofer, 2 April 1887.

#### ﻿﻿Heteromyidae Gray, 1868

***Perognathusfasciatusfasciatus* Wied, 1839**

*Perognathusfasciatus* Wied, 1839: 369.

Syntype, RMNH.MAM.63739 (Jentink 1887: 226 *b*; 1888: 94 *a*), juvenile, sex unknown, mounted skin and skull. Loc.: upper Missouri River, USA. Leg.: M. zu Wied, [1832]. Received 1846.

Wied travelled in the USA in 1832, so the date 1846 given by Jentink (1887, 1888) must be the date the specimen entered the Naturalis collection. According to Wied (1839), he collected these specimens (Wied gave measurements for two specimens) near the confluence of the Yellowstone and the upper Missouri rivers.

In view of the existence of this syntype, the designation of a neotype in the NMNH (USNM 168599) by Williams and Genoways (1979) is invalid according to ICZN art. 75.8. This fact was also overseen by Fisher and Ludwig (2012:52).

#### ﻿﻿Spalacidae Gray, 1821

***Cannomysbadius* (Hodgson, 1841)**

*Rhizomysbadius* Hodgson, 1841a: 60.

Jentink (1888: 92) recorded a specimen in Naturalis (RMNH.MAM.39360) as one of the types for *R.badius*. However Hodgson (1841a: 60) based his description on “a fine male specimen” from Nepal, which thus is the holotype by monotypy. The specimen in Naturalis is unsexed and from “Sikkim/Tibet”, so is probably not the type.

***Rhizomyssumatrensispadangensis* Brongersma, 1936**

*Rhizomyssumatrensispadangensis* Brongersma, 1936: 139.

Holotype, RMNH.MAM.54938 (formerly 1013a), female, skin and skull. Loc.: Kotagadang, Mt Singgalang at 1000 m, Sumatra, Indonesia. Leg.: E. Jacobson (382), 10 September 1917. Received 2 September 1920.

Paratypes: RMNH.MAM.52999–53000, 54937, 54939, 59666.

*Nyctocleptes Dékan* Temminck, 1832: 5 (nomen novum).

The specific epithet *dekan* by Temminck (1832) is a nomen novum for *sumatrensis* Raffles, 1821, which was placed by Temminck into a new genus *Nyctocleptes*. Temminck (1832: 3), who objected to names based on geographical names, explicitly identified his *N.dekan* with the species described by Raffles and gave a Dutch translation of the latter’s description (Temminck 1832: 5–6). Thomas (1915: 57) correctly gave *Nyctocleptessumatrensis* as the type species for *Nyctocleptes* Temminck. Brongersma (1936: 149–150) erroneously referred to the Naturalis animals as “co-types”. According to ICZN art. 72.7 “both the nominal taxa they denote have the same name-bearing type”, in this case the types for *sumatrensis* Raffles, 1821.

***Tachyoryctessplendens* (Rüppell, 1836)**

*Bathyergussplendens* Rüppell, 1836: 36, Taf. 12.

Paralectotype, RMNH.MAM.26673 (Jentink 1887: 224 *a*; 1888: 92 *a*), sex unknown, mounted skin and skull. Loc.: [Dembea province near Gondar], Ethiopia. Leg.: E. Rüppell, [1832–1833]. Received December 1837.

Rüppell (1836: 36) recorded this species in Dembea Province and Gondar. Although he gave the measurements of one animal only, he clearly had more specimens before him (p. 37).

Mertens (1925: 24) designated a specimen in the SMF (SMF 4317) the lectotype. Rüppell’s work is dated 1835 by most authors (including Musser and Carleton 2005: 925; Norris 2020: 328), but Mertens (1925: 19) specified the correct publication dates of the various instalments, which is 1836 in this case. Rüppell worked in the surroundings of Gondar between October 1832 and May 1833 (Mertens 1949: 78–80, fig. 15).

#### ﻿﻿Nesomyidae Major, 1897

*Brachyuromysramirohitra* Major, 1896: 323.

*Brachyuromysramirohitra* Major, 1896: 323.

Paratype, RMNH.MAM.26525, male, skin and skull. Loc.: Ampitambe Forest, Madagascar. Leg.: C.I.F. Major (M.1016), 17 March 1896.

Major (1896) gave measurements for three specimens in his original description, including the type (his M.429) and listed fossil material. He fails to indicate where his material is deposited. The NHM online database (https://data.nhm.ac.uk/object/c2ae898f-794e-448d-b7cd-f4a9dc96e20f/1739404800000) lists one specimen (NHMUK 1897.9.1.133) which is, according to Jenkins and Carleton (2005: 1781), the holotype. Largen (1985: 416) listed another possible paratype in the World Museum Liverpool (LIVCM-A19.4.98.24), who also erroneously considered the holotype in the NHM to be lost.

***Cricetomysgambianus* Waterhouse, 1840**

*Cricetomysgambiensis* Temminck, 1853: xvi, 168 (incorrect subsequent spelling).

Temminck (1853) had no intention to describe a new species, but misspelt *Cricetomysgambianus* Waterhouse, 1840.

***Gymnuromysroberti* Major, 1896**

*GymnuromysRoberti* Major, 1896: 324.

Paratype, RMNH.MAM.26523, female, skin and skull. Loc.: Ampitambe Forest, Madagascar. Leg.: C.I.F. Major (M.948), 15 March 1896.

Paratype: RMNH.MAM.26524.

The holotype is in the NHM (NHMUK 1897.9.1.140; https://data.nhm.ac.uk/object/de695a34-a26c-407e-9354-44fc417add03/1739404800000). Largen (1985: 416) listed another paratype in the World Museum Liverpool (LIVCM-A19.4.98.27).

***Nesomysaudeberti* (Jentink, 1879)**

*Hallomys Audeberti* Jentink, 1879e: 107.

Lectotype, RMNH.MAM.26527 (Jentink 1887: 217 *b*; 1888: 74 *a*), adult male, mounted skin, skull, postcranial skeleton and viscera in alcohol. Loc.: Savary, west of Mananare [Mananara Avaratra], Madagascar. Leg.: J.P. Audebert, February 1878.

Paralectotypes: RMNH.MAM.26528, 39356.

For an extensive discussion on the type locality see Carleton et al. (2014), who amended the type locality to “Madagascar, Toamasina Province (former), Analanjirofo Région, west of Antongil Bay towards the western frontier of Fivondronana Mananara Avaratra”.

Carleton et al. (2014: 429) also selected the specimen for which Jentink (1879e) gave the measurements in his description as lectotype. The skull of this specimen is also later depicted in Jentink 1887 (pl. VII).

***Saccostomuscampestriscampestris* Peters, 1846**

*Saccostomuscampestris* Peters, 1846: 258.

*Saccostomuslapidarius* Peters, 1852: 167, pl. XXXIV fig. 3, XXXV fig. 12.

Syntype, RMNH.MAM.26574 (Jentink 1887: 207 *a*; 1888: 53 *a*), sex unknown, mounted skin and skull. Loc.: Tete, Mozambique. Leg.: W.C.H. Peters, [1843–1847]. Ex: MfN, Berlin.

This name was first published in a preliminary communication, read in 1846 on behalf of Peters (still in Tete, Mozambique) for the Royal Prussian Academy of Sciences in Berlin. In his definitive work, Peters (1852) renamed the species *S.lapidarius.* In neither publication did he specify how many specimens he had before him; in 1852 he gave the measurements of only three animals: a male, a female, and an immature specimen. In the absence of more precise data, it seems best to regard all specimens collected by Peters as belonging in the type series for both names. Naturalis holds a specimen from Tete. It is included as “*Saccostomuscampestris*” in an inventory of animals from Mozambique offered in exchange by the MfN in June 1850 (Naturalis archives inv. 247.169); the specimen was then in alcohol. Probably the same animal (there is now only one in Naturalis) appears in a list of specimens received from Peters on 25 May 1851. Peters arrived in Mozambique in 1843 and returned to Germany in 1848 (Peters 1852: viii–ix).

***Steatomyspratensis* Peters, 1846**

*Steatomyspratensis* Peters, 1846: 258.

*Steatomysedulis* Peters, 1852: 163, pl. XXXIV fig. 2, XXXV fig. 11.

Syntype, RMNH.MAM.26491 (Jentink 1888: 52 *a*), sex unknown, mounted skin, skull in situ. Loc.: Tete, Mozambique. Leg.: W.C.H. Peters, [1843–1847]. Ex: MfN, Berlin.

This species was preliminarily described on behalf of Peters (1846) as *Steatomypratensis* and later renamed by him as *Steatomysedulis* (Peters 1852). The number of specimens that Peters had before him was not specified. Peters (1852: 164) gave the measurements of three animals: a male and two females. It seems best to regard all material collected by Peters as belonging in the type series for both names. The Naturalis specimen also occurs in the two lists in the archives (see *Saccostomuscampestris*), as “*Steatomyspratensis*”, and was originally in alcohol.

#### ﻿﻿Cricetidae Fischer, 1817

***Abrothrixjelskii* (Thomas, 1894)**

Hesperomys (Habrothrix) scalops Thomas, 1884: 455 (nec Gay, 1847) (misidentification).

*AcodonJelskii* Thomas, 1894: 360.

Paratype, ZMA.MAM.26246, male, alcohol, skull in situ. Loc.: Junín, Peru. Leg.: C. Jelski (2), [1870–1873].

Thomas (1884) misidentified the specimens collected by Jelski in Peru as *Hesperomysscalops* (Gay, 1847) (= *Chelemysmegalonyx* [Waterhouse, 1845]). Later, Thomas (1894) noted the differences between the specimens from Peru and Chile and renamed the specimens from Peru as *Acodonjelskii*.

Thomas (1894) selected a specimen in the NHM as the type (NHMUK 1885.4.1.44), but from the text it is clear he has more specimens before him. He also referred to his article on the collections made by Jelski in Peru (Thomas 1884: 455).

***Alexandromysoeconomusarenicola* (De Selys-Longchamps, 1841)**

*Arvicolaarenicola* De Selys-Longchamps, 1841: 236.

Syntype, RMNH.MAM.18873 (Jentink 1887: 222 *b*; 1888: 87 *b*), female, mounted skin and skull. Loc.: Lisse, The Netherlands. Leg.: [C.J. Temminck], 1836.

Syntype, RMNH.MAM.18874 (Jentink 1888: 87 *c*), sex unknown, mounted skin, skull extracted but missing. Loc.: Lisse, The Netherlands. Leg.: [C.J. Temminck], 1836.

Syntype, RMNH.MAM.18875 (Jentink 1888: 87 *d*), male, mounted skin and skull. Loc.: Lisse, The Netherlands. Leg.: [C.J. Temminck], 1836.

Syntype, RMNH.MAM.18876 (Jentink 1888: 87 *e*), juvenile, sex unknown, mounted skin and skull. Loc.: Lisse, The Netherlands. Leg.: [C.J. Temminck], June 1836.

Jentink (1880a) identified these specimens as *Arvicolaratticeps* Keyserling & Blasius, 1841 and lists them under this name in his catalogues (Jentink 1887; 1888). He overlooked the description by De Selys-Longchamps from the same year in which the Naturalis animals were tentatively described as *A.arenicola*, pending further examination. Later this article was brought to Jentink’s attention by G.S. Miller, who suggested that *A.arenicola* and *ratticeps* might be conspecific. After further study Jentink (1907b) agreed and recognised these specimens as the types of *Arvicolaarenicola* De Selys-Longchamps, 1841.

The pedestal of RMNH.MAM.18875 bears an inscription in Temminck’s handwriting: “Arvicola qui a dévasté les champs en Hollande dans les années 1836 et 1837”. Strangely, Jentink (1907b: 265) did not recognise this characteristic handwriting and suggested that the note had been written by De Selys-Longchamps. The latter, however, states that Temminck had collected these specimens. Temminck had a country house Wildlust near Lisse (The Netherlands), where he used to spend the summer months (Susanna 1858: 60).

***Calomyshummelincki* (Husson, 1960)**

*Baiomyshummelincki* Husson, 1960: 34, pls VI–VII.

Holotype, RMNH.MAM.15994, female, skin and skull. Loc.: Klein Santa Martha, Curaçao. Leg.: A.B. Bitter, 5 January 1947. Ex: P. Wagenaar Hummelinck (Mus 33).

Paratypes: RMNH.MAM.15988, 16003, 16009; ZMA.MAM.1564, 1570, 2480.

***Euryoryzomysnitidus* (Thomas, 1884)**

*Hesperomyslaticeps*, *var. nitidus* Thomas, 1884: 452, pl. XLII fig. 1.

Paralectotype, ZMA.MAM.16884, female, skull. Loc.: Junín, Junín Dept., Peru. Leg: C. Jelski (14), 1870–1873. Ex: NHM (O. Thomas).

Thomas (1884) mentioned 18 specimens in his description; however, he did not select a type. Thomas (1927: 549) designated a lectotype in the NHM (NHMUK 1885.4.1.41).

***Megalomysdesmarestii* (Fischer, 1829) (extinct)**

*Muspilorides* Desmarest, 1826: 483 (nec Pallas, 1778).

*M* (*us*) *Desmarestii* Fischer, 1829: 316 (nomen novum).

Syntype, RMNH.MAM.21287 (Jentink 1888: 80 *a*), male, mounted skin and incomplete skull. Loc.: Martinique. Leg.: A. Plée, [1820–1821]. Ex: MNHN, 1825.

Jentink (1888) recorded this specimen as “*Megalomyspilorides* Pallas”. Apparently, he overlooked Fischer’s synopsis, in which the species was identified with “*M.pilorides Desmar. in Nouv. Dict.* XLIV. p. 483”. Desmarest (1826) did not state whether he had more than one specimen and clearly stated Martinique as its provenance. Fischer (1829) recognised that Desmarest’s name is preoccupied by *Muspilorides* Pallas, 1778 (= *Capromyspilorides*), so he renamed the species *Musdesmarestii*. Fischer worked in Naturalis and obviously had this specimen available when compiling his work. It was received from the MNHN in 1825, so was among the material collected by Plée and seen by Desmarest some years earlier.

Plée collected in Martinique between May 1820 and February 1821, and again in 1825; he died on the island in November of that year. His collections were sent to the MNHN (Raymond 1957: 193–201). RMNH.MAM.21287 must have been collected during his first sojourn in Martinique.

Miljutin (2010: 219) mentioned two syntypes in the MNHN (CG 1979-385 and CG 2006-187).

***Neodonsikimensis* Horsfield, 1851**

*Neodonsikimensis* Horsfield, 1851: 146.

This name was first published by Horsfield (1849: 203) as a nomen nudum, and the description followed in 1851 (Horsfield 1851: 146). This description was based on a single specimen in the collection of the East India Company. This specimen, the holotype, is in the NHM (NHMUK 1879.11.21.395). Jentink (1888: 89) erroneously listed a specimen in Naturalis (RMNH.MAM.19144, mounted skin, skull in situ, from Tibet by B.H. Hodgson) as a type, but Horsfield described a specimen from Sikkim, not Tibet (see also Kaneko and Smeenk 1996).

#### ﻿﻿Muridae Illiger, 1811

***Acomysdimidiatusdimidiatus* (Cretzschmar, 1826)**

*Musdimidiatus* Cretzschmar, 1826: 37, tab. 13 fig. b.

Possible paralectotype, RMNH.MAM.26499 (Jentink,1887: 213 *a*; 1888: 68 *a*), sex unknown, mounted skin and skull. Loc.: Egypt. Leg.: E. Rüppell.

Possible paralectotypes: RMNH.MAM.26500, 26501.

Jentink (1887; 1888) did not list these specimens as types and their type status is uncertain. The species was described based on material collected by Rüppell in the Sinai and surroundings, later also in Nubia (Cretzschmar 1826: 38). The pedestals of two of the Naturalis animals read, in Temminck’s handwriting: “Rupp Atlas tab 13 f a Egypte”. They can only belong in the type series if they were collected during the first part of Rüppell’s first expedition, between January 1822 and July 1825; in 1822 Rüppell collected in Egypt and Sinai, and in January 1823 travelled further south into Sudan. Results of this part of the expedition were published by Cretzschmar before Rüppell’s return to Germany (Mertens 1949: 29–50). It cannot be reconstructed whether our specimens were indeed collected in Egypt in 1822 or are from later expeditions. They are not mentioned in the lists of exchanges between Naturalis and SMF.

Mertens (1925: 25) designated a specimen in the SMF (SMF 4321) as the lectotype.

***Apodemusargenteus* (Temminck, 1844)**

*Musargenteus* Temminck, 1844: 51.

Lectotype, RMNH.MAM.19688 (Jentink 1888: 63 b; 1887: 210 b), juvenile, sex unknown, mounted skin and skull. Loc.: Japan. Leg.: H. Bürger [1830–1834].

Paralectotype, RMNH.MAM.24211–24212 (= *Apodemusspeciosus*).

The lectotype of *Apodemusargenteus* is the only specimen of the type series which actually belongs to this species. The paralectotypes had been identified as juvenile specimens of *Apodemusspeciosus* (Temminck, 1844) by Smeenk et al. (1982: 126) who also designated RMNH.MAM.19688 the lectotype. However, they overlooked the earlier lectotype designation by Tate (1940: 7), who selected RMNH.MAM.24211 as the lectotype by listing it as the type. As Tate stated not having seen the other specimens, and RMNH.MAM.24211 does not belong to *Apodemusargenteus* as currently understood taxonomically, we follow the lectotype designation by Smeenk et al. (1982) pending resolution of this matter by application to the ICZN.

In the detailed cargo lists compiled by Bürger in the Naturalis archives (inv. 232.1440) several shipments with “*Mus*” specimens are recorded between December 1830 and November 1834 (see for the list Smeenk et al. 1982: pl. 4).

***Apodemusspeciosus* (Temminck, 1844)**

*Musspeciosus* Temminck, 1844: 52.

Syntype, RMNH.MAM.19686 (Jentink 1887: 210 *a*; 1888: 63 *a*), adult, sex unknown, mounted skin and skull. Loc.: Japan. Leg.: H. Bürger [1830–1834].

Syntype, RMNH.MAM.19687 (Jentink 1887: 210 *b*; 1888: 63 *b*), adult, sex unknown, mounted skin and skull. Loc.: Japan. Leg.: H. Bürger [1830–1834].

Smeenk, in his draft of this catalogue, listed RMNH.MAM.19686 as lectotype and RMNH.MAM.19687 as paralectotype. We have not been able to find the references for these designations and therefore list these specimens as syntypes.

***Arvicanthisabyssinicus* (Rüppell, 1842)**

*Musrufodorsalis* Heuglin, 1877: 70.

Syntype, RMNH.MAM.26513 (Jentink 1887: 214 a; 1888: 69 a), adult, sex unknown, mounted skin and skull. Loc.: “Egypt” [error]. Leg.: T. von Heuglin, 1861. Ex SMNS (Kraus), 1862.

Syntype, RMNH.MAM.26514 (Jentink 1888: 69 b), adult, sex unknown, mounted skin, skull in situ. Loc.: Wogara, Ethiopia. Leg.: T. von Heuglin, 1861. Ex SMNS (Kraus), 1862.

Von Heuglin (1877) described this new species based on his fieldnotes. The locality “Egypt”, in Jentink (1888) must be an error, as this species does not occur in Egypt. Another syntype is in the SMNS (SMNS 1052; Dieterlen et al. 2013: 297).

***Arvicanthisrufinus* (Temminck, 1853)**

*Musrufinus* Temminck, 1853: 163.

Lectotype, RMNH.MAM.19207 (Jentink 1888: 70 a; 1887: 214 a), adult, sex unknown, mounted skin and skull. Loc.: St.George d’Elmina, Côte d’Or [Ghana]. Leg.: H.S. Pel.

Temminck (1853) mentioned having two specimens; only one specimen is still present in Naturalis. Jentink (1887: 214) designated RMNH.MAM.19207 the lectotype.

***Bandicotabengalensis* (Gray, 1835)**

*Gunomysbengalenissundavensis* Kloss, 1921b: 116.

Syntype, RMNH.MAM.9825, adult male, skin and skull. Loc.: Olee-Lheuë, Atjeh, Indonesia. Leg.: “Gouv. veearts”, 22 September 1916. Ex: MZB (MZB 222).

Syntype, RMNH.MAM.9826, subadult female, skin and skull. Loc.: Olee-Lheuë, Atjeh, Indonesia. Leg.: “Gouv veearts”, 22 September 1916. Ex: MZB (MZB 221).

On the label of RMNH.MAM.39361 (adult female from Sumatra, collected 24 December 1914) is written “paratype”. Kloss in his description mentioned only two syntypes. The specimens were collected by a government veterinarian.

***Gerbilliscusafer* (Gray, 1830)**

*GerbillusAfra* Gray, 1830: 10.

*Mussericeus* “Temminck” Gray, 1830: 10 (in synonymy).

*Merionesschlegelii* Smuts, 1832: 41.

Syntype, RMNH.MAM.26610 (Jentink 1888: 49 e), adult, sex unknown, mounted skin, skull in situ. Loc.: ‘Cap’ (South Africa). Leg.: H.B. van Horstok.

Syntype, RMNH.MAM.26611 (Jentink 1888: 49 f), adult, sex unknown, mounted skin, skull in situ. Loc.: ‘Cap’ (South Africa). Leg.: H.B. van Horstok.

Syntype, RMNH.MAM.26612 (Jentink 1888: 49 g), juvenile, sex unknown, mounted skin, skull in situ. Loc.: ‘Cap’ (South Africa). Leg.: H.B. van Horstok.

Syntype, RMNH.MAM.26621 (Jentink 1887: 204 a), adult, sex unknown, skeleton. Loc.: ‘Cap’ (South Africa). Leg.: H.B. van Horstok.

Syntype, RMNH.MAM.26622 (Jentink 1887: 204 b), adult, sex unknown, skeleton. Loc.: ‘Cap’ (South Africa). Leg.: H.B. van Horstok.

Syntype, RMNH.MAM.39357 (Jentink 1888: 49 k), juvenile, sex unknown, alcohol. Loc.: ‘Cap’ (South Africa). Leg.: H.B. van Horstok.

In his description of *Gerbillusafra*, Gray (1830) referred to specimens in Naturalis with the manuscript name *Mussericeus* Temminck. We have found no subsequent use of *Mussericeus* to validate this name and it is therefore not available. However, by referring to the specimens in Naturalis with this name, they are included in the type series of *Gerbillusafra* Gray, 1830.

Smuts (1832: 44) also referred to the manuscript name by Temminck (cited as *cericeus*) as a synonym for *Gerbillusafra*Gray 1830. Smuts (1832) introduced his new species *Merionesschlegelii*, but was uncertain about its status, as in a footnote on p. 44 he seemed to question whether it might be the same species as *Gerbillusafra*. It is unclear to what part of the text this footnote refers to.

According to Smuts (1832: 45) the first reported specimen of *Merionesschlegelii* originated from Port Elisabeth. None of the specimens listed in Jentink (1887; 1888) are from Port Elisabeth and it is unclear whether this specimen is preserved.

***Gerbilliscusrobustus* (Cretzschmar, 1826)**

*Merionesmacropus* von Heuglin, 1864: 9.

Syntype, RMNH.MAM.26604 (Jentink 1888: 48 a), adult male, mounted skin, skull in situ. Loc.: Bongos, Bahr el Ghazal, South-Sudan. Leg.: Th. von Heuglin, January 1863. Received 1865.

Syntype, RMNH.MAM.26605 (Jentink 1888: 48 b), adult, sex unknown, mounted skin, skull in situ. Loc.: Bongos, Bahr el Ghazal, South-Sudan. Leg.: Th. von Heuglin, December 1863. Received 1865.

Dieterlen et al. (2013: 297) erroneously listed a specimen in the SMNS (SMNS 1098) as holotype, as von Heuglin (1864) did not nominate types and gave no indication of the number of specimens available to him. The mere use of the term holotype does not constitute a valid lectotype designation (ICZN art. 74.7). We therefore list these specimens as syntypes.

***Grammomysdolichurus* (Smuts, 1832)**

*Musdolichurus* Smuts, 1832: 38.

Lectotype, RMNH.MAM.19511 (Jentink 1888: 70 *a*), adult, sex unknown, relaxed mount, skull extracted (see remark). Loc.: ‘Cap’ (South Africa). Leg.: H.B. van Horstok.

Paralectotypes: RMNH.MAM.19512, 26543, 26544.

Jentink (1888: 70) listed RMNH.MAM.19511 as the type, thereby designating it the lectotype for *Musdolichurus* Smuts, 1832. The skulls of RMNH.MAM.19511 and 19512 are extracted, but not present in Naturalis. It is possible that the skull and skeleton listed as separate specimens are in fact derived from the two skins.

***Hydromyshussoni* Musser & Piik, 1982**

*Hydromyshussoni* Musser & Piik, 1982: 156.

Holotype, RMNH.MAM.29140, male, skin and skull. Loc.: Enarotali, Lake Paniai, Wissel Lakes, New Guinea, Indonesia. Leg.: H. Boschma (0126), 9 November 1939, K.N.A.G. Central New Guinea Expedition 1939.

Paratypes: RMNH.MAM.29141–29175, 29177–29179, 29740–29752.

See Boschma (1943: 507) for the localities. Most specimens were obtained by Boschma from local hunters. One of the specimens listed in Musser and Piik (1982) was exchanged with the Australian Museum in 1987 (RMNH.MAM.29176, a female from Enarotali from 1 October 1939, field nr. 1479/3853).

***Kadarsanomyssodyi* (Bartels, 1937)**

*Rattuscanussodyi* Bartels, 1937a: 45.

Holotype, RMNH.MAM.13595, adult female, skin and skull. Loc.: S.W. slopes of Mts. Pangrango-Gede, West Java, Indonesia. Leg.: M. Bartels Jr., 18 October 1933. Ex: coll. Bartels (580), June 1954.

In his description Bartels (1937a) listed nine specimens and mentioned having examined 17, all from the type locality, but he restricted the type series to the holotype, now in Naturalis, and two paratypes (MZB 5020 and 5083) in the MZB.

***Komodomysrintjanus* (Sody, 1941)**

*Rattusrintjanus* Sody, 1941: 310.

Holotype, RMNH.MAM.9801, adult male, skin and skull. Loc.: Lohoboeaja [Loh Buaya], Pulau Rintja, Indonesia. Leg.: J.K. de Jong, 8 November 1929. Ex: MZB (MZB 2405).

***Lemniscomysrosalia* (Thomas, 1904)**

*Musspinalis* “Sundevall” Jentink, 1888a: 70 (in synonymy).

Jentink (1888: 70) listed a specimen (RMNH.MAM.19509, from South Africa, collected by C.J. Sundevall) as one of the types of *Musspinalis* Sundevall in the synonymy of *Musdorsalis*. However we have not been able to find any reference to this name or subsequent use to validate this name. Later Thomas (1916: 69) introduced *Lemniscomysgriseldaspinalis* as a replacement for *Musdorsalis* A. Smith, 1845, preoccupied by *Sicistadorsalis* G. Fischer, 1814.

***Lenomysmeyeri* (Jentink, 1879)**

*Mus Meyeri* Jentink, 1879a: 12.

Holotype by monotypy, RMNH.MAM.18302 (Jentink 1887: 211 *a*; 1888: 65 *a*), sex unknown, mounted skin and skull. Loc.: Langoan, Sulawesi, Indonesia. Leg.: S.C.J.W. van Musschenbroek, September 1875.

***Leopoldamyssabanus* (Thomas, 1887)**

*Echimysmacrourus* “Temminck” Jentink, 1879d: 97.

Holotype by monotypy, RMNH.MAM.17215 (Jentink 1888: 101 *a*), juvenile, sex unknown, mounted skin, skull extracted, but not in collection. Loc.: “Suriname” [= West Sumatra, Indonesia. Leg.: S. Müller, 1833–1836].

The pedestal of this specimen in Temminck’s handwriting reads: “*Echimysmacrourus* Temm Sp Nov par Dippering Surinam”, but this manuscript name and provenance were published much later by Jentink (1879d).

Husson (1963: 37–40) identified this specimen with the Southeast Asian *Rattussabanus* Thomas, 1887. Apparently a specimen collected in Sumatra had become mixed up with material from Suriname collected by H.H. Dieperink (misspelt “Dippering” by Temminck), who worked in that country between 1816 and 1836. The animal most probably was collected by S. Müller, who travelled in western Sumatra between January 1833 and July 1836. Husson (1963: 40; repeated in Musser 1981: 257) pointed out that *macrourus* Jentink, 1879 has priority over *sabanus* Thomas, 1887.

***Lophuromyssikapusi* (Temminck, 1853)**

*Mussikapusi* Temminck, 1853: 160.

Syntype, RMNH.MAM.25734 (Jentink 1888: 52 a; 1887: 206 a), adult female, mounted skin and skull. Loc.: Dabocrom, Côte d’Or [Ghana]. Leg.: H.S. Pel.

Syntype, RMNH.MAM.25735 (Jentink 1888: 52 c), juvenile female, mounted skin, skull in situ. Loc.: Dabocrom, Côte d’Or [Ghana]. Leg.: H.S. Pel.

Syntype, RMNH.MAM.25736 (Jentink 1888: 52 d), juvenile, sex unknown, mounted skin, skull extracted but not in collection. Loc.: Dabocrom, Côte d’Or [Ghana]. Leg.: H.S. Pel.

***Lorentzimysnouhuysii* Jentink, 1911.**

*LorentzimysNouhuysii* Jentink, 1911b: 165, 174.

Holotype by monotypy, RMNH.MAM.29280, adult male, skin and skull. Loc.: “Bivak II”, New Guinea, Indonesia, alt. 400 m. Leg.: H.A. Lorentz (319), 10 October 1909, Second South New-Guinea Expedition 1909–1910.

Laurie and Hill (1954) located Bivak II on 4°38'S, 138°48'E.

***Margaretamysbeccarii* (Jentink, 1880)**

*Musbeccarii* Jentink, 1880b: 11 (nomen novum).

*Musleucopus* Jentink, 1879a: 8 (nec Gray, 1867) (misidentification).

Lectotype, RMNH.MAM.18305 (Jentink 1887: 212 *a*; 1888: 66 *a*), adult, sex unknown, relaxed mount and skull. Loc.: Menado-Langowan, Sulawesi, Indonesia. Leg.: S.C.J.W. van Musschenbroek, September 1875. Received 1877.

Paralectotype: RMNH.MAM.18306.

Jentink (1879a: 8) identified specimens in the Leiden collection as *Acanthomysleucopus* (Gray, 1867) and remarked on Alston (1877: 124), who in his view unnecessarily introduced a new name for this species. Later Alston (1879: 645) separated the Sulawesi specimens from the Australian material, followed by Jentink (1880b) who renamed the former as *Musbeccarii*.

Jentink (1888: 66; *terrae-reginae*, cat. *a*) listed a specimen from Aru collected by von Rosenberg as the type for *Musleucopus* Jentink. This specimen is mentioned in his 1880 description of *Musbeccarii*, but as agreeing with the description of *Musterrae-reginae* by Alston and used as an illustration of the wide distribution of species of *Mus.* Because this specimen does not feature in the original description of *Musleucopus* (Jentink 1879), it is not a type.

Musser (1971b: 153) designated the lectotype because of the better agreement of this specimen with the measurements given by Jentink (1879a).

*Rattusthysanurus* Sody, 1932b: 157.

Holotype, RMNH.MAM.21235, adult female, skin and skull. Loc.: Toemaratas, Sulawesi, Indonesia. Leg.: H. Witkamp, 1932. Ex: H.J.V. Sody (P.59).

***Mastomyserythroleucus* (Temminck, 1853)**

*Muserythroleucus* Temminck, 1853: 160.

Syntype, RMNH.MAM.19206 (Jentink 1887: 214 *a*; 1888: 70 *a*), adult, sex unknown, mounted skin and skull. Loc.: Côte d’Or [Ghana]. Leg.: H.S. Pel.

Temminck (1853) gave no indication of the specimens available to him.

***Mastomysnatalensis* (A. Smith, 1834)**

*Musillovoensis* Jentink, 1909b: 240, 248.

Holotype, RMNH.MAM.27425, adult male, skin (missing) and skull. Loc.: Natal, lower Illovo River, South Africa. Leg.: M. Weber (227), [1894].

Weber visited South Africa in 1894 (Pieters and De Visser 1993).

***Maxomysbartelsii* (Jentink, 1910)**

*Musbartelsii* Jentink, 1910: 69.

Holotype by monotypy, RMNH.MAM.18329, adult, sex unknown, skin and skull. Loc.: Pangerango, Java, Indonesia. Leg.: M. Bartels, April 1903. Received September 1909.

*Rattusbartelsi* [sic] *tjibuniensis* Sody, 1933: 430.

Holotype, RMNH.MAM.21343, adult male, skin and skull. Loc.: Tjiboeni (Cibuni), Bandung, Java, Indonesia. Leg.: M. Bartels Jr., 20 August 1932. Ex: H.J.V. Sody (Tjib.52).

Paratypes: RMNH.MAM.21344–21362.

Sody (1933) examined 22 specimens.

*Rattusbartelsiiobscuratus* Bartels, 1938: 323.

Holotype, RMNH.MAM.14147, adult male, skin. Loc.: G. Slamet, Java, Indonesia. Leg.: M. Bartels Jr. and P.J. Bouma (Sl.37), 26 August 1933. Ex: coll. Bartels, June 1954.

Paratypes: RMNH.MAM.13207, 13528, 13529, 13547, 13582, 13583, 13758, 13841, 13998, 14140, 14145, 14146, 14148, 14150–14151, 14200–14203, 22914–22919.

According to Bartels (1938) the holotype was preserved as a skin and skull. However, only the skin was received in June 1954, when the Bartels collection was acquired for the Naturalis collection.

***Maxomyshellwaldii* (Jentink, 1879)**

*MusHellwaldii* Jentink, 1879a: 11.

Lectotype, RMNH.MAM.18307 (Jentink 1887: 212 *a*; 1888: 65 *a*), sex unknown, mounted skin and skull. Loc.: Langoan, Sulawesi, Indonesia. Leg.: S.C.J.W. van Musschenbroek, September 1875.

Paralectotype: RMNH.MAM.18308.

Jentink (1887: 212) designated the lectotype.

*Rattushellwardigriseogenys* Sody, 1941: 305.

Holotype, RMNH.MAM.9792, adult female, skin and skull. Loc.: Masembo River, Sulawesi, Indonesia, alt. 550 m. Leg.: G. Heinrich, 26 January 1932 (Heinrich Expedition 1931, nr. 843). Ex: American Museum of Natural History. Ex: MZB (MZB 93/41; 4091).

Sody (1941) referred to two female specimens in his description. The paratype is not in Naturalis.

***Maxomysmusschenbroekii* (Jentink, 1879)**

*MusMusschenbroekii* Jentink, 1879a: 10.

Lectotype, RMNH.MAM.21070 (Jentink 1887: 212 *a*; 1888: 66 *a*), sex unknown, mounted skin and incomplete skull. Loc.: Langoan, Sulawesi, Indonesia. Leg.: S.C.J.W. van Musschenbroek, September 1875.

Paralectotype: RMNH.MAM.21071.

Jentink (1887: 212) designated the lectotype.

*Rattusmusschenbroekiilalawora* Sody, 1941: 305.

Holotype, RMNH.MAM.9791, adult female, skin and skull. Loc.: Tanke Salokko, Sulawesi, Indonesia. Leg.: G. Heinrich, 23 December 1931 (Heinrich Expedition 1931, nr. 682). Ex: American Museum of Natural History. Ex: MZB (MZB 94/41; 4093).

***Maxomysrajah* (Thomas, 1894)**

*Rattusrajahhidongis* Kloss, 1921b: 122.

Holotype, RMNH.MAM.9804, adult female, skin and skull. Loc.: Serasan, Natuna Isl., Indonesia. Leg.: posthouder, 12 August 1916 (185). Ex: MZB (MZB 285).

Kloss (1921b) referred to this specimen with the number 185, an error for 285. The “posthouder’ was the local representative of the government, and his name is not known.

***Maxomyssuriferravus* (H.C. Robinson & Kloss, 1916)**

*Rattusrajahverbeeki* Sody, 1930b: 130.

Holotype, RMNH.MAM.22452, adult male, skin and skull. Loc.: Gedangan, Java, Indonesia. Leg.: F.A.Th.H. Verbeek, 10 December 1929. Ex: H.J.V. Sody (I).

Paratype: RMNH.MAM.22453.

*Rattussurifersolaris* Sody, 1934: 170.

Holotype, RMNH.MAM.22478, adult female, skin and skull. Loc.: Situ Gunung, Gunung Gedeh, Java, Indonesia. Leg.: M. Bartels Jr. (SG.169), Jan.–June 1933. Ex: H.J.V. Sody.

Paratypes: RMNH.MAM.22479–22488.

***Maxomyswhiteheadi* (Thomas, 1894)**

*Rattuswhiteheadicoritzae* Sody, 1941: 299.

Holotype, RMNH.MAM.9795, adult female, skin and skull. Loc.: Kp. Riam distr., Kotawaringin, Borneo, Indonesia. Leg.: J.J. Menden, 1 November 1935. Ex: MZB (MZB 5682; 83/36), 12 January 1950.

Sody (1941) examined eight specimens in his description; only the holotype is in Naturalis. This specimen is a female and not a male (error on Sody’s label on skin in Naturalis).

***Melomysleucogaster* (Jentink, 1909)**

*Pogonomysleucogaster* Jentink, 1909a: 3.

Syntype, RMNH.MAM.25493, adult female, skin and skeleton. Loc.: Alkmaar, Papua, Indonesia. Leg.: H.A. Lorentz (119), Eerste Zuid Nieuw-Guinea Expeditie 1907, 11 August 1907.

Syntype, RMNH.MAM.29267, subadult male, skin and skeleton. Loc.: Sabang, Papua, Indonesia. Leg.: H.A. Lorentz (78), Eerste Zuid Nieuw-Guinea Expeditie 1907, June 1907.

Syntype, RMNH.MAM.29268, adult female, skin and skeleton. Loc.: Alkmaar, Papua, Indonesia. Leg.: H.A. Lorentz (106), Eerste Zuid Nieuw-Guinea Expeditie 1907, 30 Juli 1907.

Syntype, RMNH.MAM.29269, juvenile male, skin and skeleton. Loc.: Alkmaar, Papua, Indonesia. Leg.: H.A. Lorentz (106), Eerste Zuid Nieuw-Guinea Expeditie 1907, 3 Juli 1907.

Some sources (Musser and Carleton 2005: 1378; Denys et al. 2020: 502) date the publication of this name in 1908, based on the publication date in the header: “October 1908”. However the first issue of Nova Guinea 9 was published in 1909 (see title page).

Of the four specimens used by Jentink (1909a) for his description, Rümmler (1938: 103) listed RMNH.MAM.25493 as “Typus” and Menzies (1996: 405) referred to this specimen as “Holotype”. Both these actions do not constitute valid lectotype designations according to ICZN art. 74.5 and art. 74.6.

***Melomysrufescens* (Alston, 1877)**

*Pogonomyssexplicatus* Jentink, 1907a: 366.

Holotype by monotypy, RMNH.MAM.29281, adult pregnant female, flat skin and skeleton. Loc.: Sentani meer, Indonesia. Leg.: C.E.A. Wichmann, 2 April 1903, Noord Nieuw-Guinea Expeditie (1903) (Humboldt Bay Expedition).

***Melomysrufescenswisselensis* Menzies, 1996**

*Melomysrufescenswisselensis* Menzies, 1996: 404.

Holotype, RMNH.MAM.29205, adult male, skin and skull. Loc.: Arabu River [Araboe bivouac], New Guinea, Indonesia. Leg.: H. Boschma (1615/3833), 4 November 1939, K.N.A.G. Central New Guinea Expedition 1939.

Paratypes: RMNH.MAM.29184–29193, 29200–29204, 29206–29209, 29212, 29213, 29216.

***Muscaroli* Bonhote, 1902**

*Mus* (*musculus*?) *ouwensi* Kloss, 1921b: 120.

Holotype, RMNH.MAM.9820, adult male, skin and skull. Loc.: Probolinggo, Java, Indonesia. Leg.: Lim King Siang, 13 February 1915. Ex: MZB (MZB 237), 12 January 1950.

Paratypes: RMNH.MAM.39362–39364.

Kloss (1921b) mentioned 17 specimens, of which four are in Naturalis.

***Musmusculoides* Temminck, 1853**

*Musmusculoides* Temminck, 1853: 161.

Holotype, RMNH.MAM.26521 (Jentink 1888: 73 *a*), sex unknown, mounted skin, skull in situ. Loc.: “Côte de Guiné” [Ghana]. Leg.: H.S. Pel, [1841–1850].

***Musmusculuscastaneus* Waterhouse, 1843**

*Musmolossinus* Temminck, 1844: 51.

Syntype, RMNH.MAM.18824 (Jentink 1887: 209 *f*; 1888: 60 *o*), adult female, mounted skin and skull. Loc.: Japan.

Muswagnerivar.rotans Droogleever Fortuyn, 1912: 189.

Lectotype, ZMA.MAM.27233, sex unknown, alcohol and skull. Loc.: “Japan” (bred in captivity in NAM, Amsterdam). Leg.: A.B. Droogleever Fortuyn.

Paralectotypes:, ZMA.MAM.27234–27242.

Droogleever Fortuyn (1912: 184) provided measurements for 11 specimens. Bergmans (2011: 838) referred to 10 type specimens for this name (ZMA.MAM.27233–27242). They were found in a jar labelled ”Typen/ *Muswagneri* varietas *rotans* Droogl. Fort./Japansche Dansmuis”. However 19 more specimens (ZMA.MAM.27184–27203) are present in the collection; we do not include these in the type series. Cruz et al. (2024: 838) designated the lectotype.

The specimens available to Droogleever Fortuyn were bred in NAM from animals imported from Vienna, Austria by C. Kerbert (then director of the NAM) and one was bred by C.U.A. Zwaardemaker in Utrecht (Droogleever Fortuyn 1912). When and by whom their ancestors were imported from Japan is unknown.

*Musmusculusfredericae* Sody, 1933: 438.

Holotype, RMNH.MAM.22618, adult female, skin and skull. Loc.: Poso, Sulawesi, Indonesia. Leg.: H. Witkamp, 1932. Ex: H.J.V. Sody (PO.164).

Paratypes: RMNH.MAM.22619–22625.

***Niviventerbukit* (Bonhote, 1903)**

*Rattuslepturusbesuki* Sody, 1931a: 214.

Holotype, RMNH.MAM.21311, adult female, skin and skull. Loc.: Ongop-ongop, Java, Indonesia, alt. 1850 m. Leg.: K.W. Dammerman, 18 May 1924. Ex: H.J.V. Sody (Z.1). Ex: Museum Zoologicum Bogoriense.

Paratype: RMNH.MAM.21312.

*Rattusbukitbaturus* Sody, 1932a: 334.

Holotype, RMNH.MAM.21313, adult male, skin and skull. Loc.: G. Agoeng, Bali, Indonesia. Leg.: A. Samat, 30 July 1930. Ex: H.J.V. Sody (E. 101).

Paratypes: RMNH.MAM.21314, 21315.

Another specimen in Naturalis (RMNH.MAM.21316) is not included in the type series as it was only later identified as *Rattusbukitbaturus* (Becking 1989: 60).

*Rattusbukitlepturoides* Sody, 1934: 174.

Holotype, RMNH.MAM.21317, adult female, skin and skull. Loc.: G. Lawoe, Java, Indonesia. Leg.: M. Bartels Jr. and H.J.V. Sody (L.13), 14 June 1933.

Paratypes: RMNH.MAM.21318–21321.

*Rattusbukitjacobsoni* Bartels, 1937b: 121.

Holotype, RMNH.MAM.23540, adult male, skin and skull. Loc.: “De Giesting”, G. Tanggamoes, Sumatra, Indonesia. Leg.: M. Bartels Jr. and Ch. Berkholst, 18 June 1935. Ex: Coll. Bartels (S.30).

Paratypes: RMNH.MAM.13811, 23541.

Bartels (1937b) specifically mentioned two paratypes (his S 135 and S 140), at that time housed in the MZB; these paratypes are now in Naturalis. All other specimens mentioned in his description are present in Naturalis, but not part of the type series.

*Rattusbukitlieftincki* Chasen, 1939b: 208.

Holotype, RMNH.MAM.9798, adult female, skin and skull. Loc.: Atang Poetar, Atjeh, Indonesia. Leg.: A. Hoogerwerf, 12 April 1937, N. Sumatra Expedition Atjeh I–V 1937. Ex: MZB (MZB 5583; 478/37).

Chasen (1939b) examined 14 specimens for his description, but only the holotype is in Naturalis. For a report and map of this expedition see Hoogerwerf (1939).

***Niviventercremoriventer* (Miller, 1900)**

*Rattuscremoriventersumatrae* Bartels 1937b: 123.

Holotype, RMNH.MAM.23174, adult male, skin and skull. Loc.: Bandjarnegri, Sumatra, Indonesia. Leg.: M. Bartels Jr. and Ch. Berkholst, 2 July 1935. Ex: M. Bartels (S.180)

Paratype: RMNH.MAM.23175.

Bartels (1937b) indicated that he would send the paratype (S.161) to the MZB. It is, however, present in Naturalis.

***Niviventerfraternus* (Robinson & Kloss, 1916)**

*Rattusrapitatchinensis* Sody, 1941: 295.

Holotype, RMNH.MAM.9803, adult male, skin and skull. Loc.: Paja Toong Kalan, Atjeh, N. Sumatra, Indonesia. Leg.: Madzoed and H.R. Rookmaker, 9 September 1930. Ex: MZB (MZB 3156).

Sody (1941) examined six specimens; only the holotype is in Naturalis.

***Niviventerfulvescens* (Gray, 1847)**

*Muscaudatior* Hodgson, 1849a: 203.

Syntype, RMNH.MAM.19198 (Jentink 1888: 62 *a*), adult, sex unknown, mounted skin, skull in situ. Loc.: Tibet. Leg.: B.H. Hodgson.

***Niviventerlepturus* (Jentink, 1880)**

*Muslepturus* “Temminck” Jentink, 1880c: 17.

Holotype by monotypy, RMNH.MAM.18321 (Jentink 1887: 211 *a*; 1888: 64 *a*), adult, sex unknown, mounted skin and skull. Loc.: Java, Indonesia.

*Rattuslepturusfredericae* Sody, 1931a: 212.

Holotype, RMNH.MAM.21405, adult male, skin and skull. Loc.: G. Salak, Java, Indonesia. Leg.: A. Samat, 20 June 1931. Ex: H.J.V. Sody (A.188).

Paratypes: RMNH.MAM.21406–21416.

*Rattuslepturusmaculipectus* Sody, 1934: 173.

Holotype, RMNH.MAM.21445, adult female, skin and skull. Loc.: Gunung Cereme, Java, Indonesia. Leg.: H.J.V. Sody and M. Bartels Jr. (K.20), 7 June 1933.

Paratypes: RMNH.MAM.21446, 21467.

Sody’s material consisted of 25 specimens (Sody 1934); all specimens except two are in Naturalis.

***Papagomysarmandvillei* (Jentink, 1892)**

*Musarmandvillei* Jentink, 1892a: 79.

Holotype, RMNH.MAM.18301 (1024), adult female, skin and skeleton. Loc.: Flores, Sikka, Indonesia. Leg.: C.J.F. le Cocq d’Armandville, 1889. Ex: M. Weber.

The type was originally sexed as male, which was later corrected to female.

***Paramelomyslorentzii* (Jentink, 1909)**

*Pogonomyslorentzii* Jentink, 1909a: 3.

Holotype, RMNH.MAM.25494, adult female, skin and skeleton. Loc.: Resi kamp, Lorentz River, Papua, Indonesia. Leg.: H.A. Lorentz, September 1907, First South New Guinea Expedition 1907 (132).

Some sources (Musser and Carleton 2005: 1432; Denys et al. 2020: 504) date the publication of this name in 1908, based on the imprint date “October 1908”; however, the first livraison of Nova Guinea vol. 9 was published in 1909 (see title page).

Jentink described this specimen as a female, although the field label clearly says male. Rümmler (1938: 139), who stated to have studied the specimen, also lists this specimen as a female.

***Paramelomysplatyops* (Thomas, 1906)**

*Melomysplatyopsmamberanus* Sody, 1937: 218.

Holotype, RMNH.MAM.22491, adult male, skin and skull. Loc.: Pionier bivak, Mamberamo River, New Guinea, Indonesia. Leg.: W.C. van Heurn (28), 15 July 1920, N. Guinea Expedition 1920. Ex: H.J.V. Sody.

Paratypes: RMNH.MAM.22492, 22493.

***Paruromysdominator* (Thomas, 1921)**

*Taeromysdominatorursinus* Sody, 1941: 312.

Holotype, RMNH.MAM.9823, adult male, skin and skull. Loc.: Wawa Karaeng, Mt. Lompobatang, Sulawesi, Indonesia. Leg.: G. Heinrich, 15 April 1931, Heinrich Expedition 1931, nr. 101170. Ex: MZB (MZB 5593; 92/41).

***Psammomysobesusobesus* Cretzschmar, 1828**

*Psammomysobesus* Cretzschmar, 1828: 58, tab. 22, 23.

Paralectotype, RMNH.MAM.26593 (Jentink 1887: 205 *a*; 1888: 50 *a*), sex unknown, mounted skin and incomplete skull. Loc.: [Alexandria], Egypt. Leg.: E. Rüppell, [summer 1822]. Ex: SMF.

Paralectotypes: RMNH.MAM.26596–26599.

Rüppell sent several specimens of this new species to Cretzschmar (1828: 59). Rüppell stayed in Alexandria in January–February and again in the late summer of 1822, leaving in October of that year (Mertens 1949: 30, 34, 300–301). During his second expedition, he was in Alexandria in January 1831, where he made additional observations of this species and collected one more specimen (Mertens 1949: 54, 343). We can therefore assume that our animals were obtained in the summer of 1822. However, only two specimens are mentioned in a list of an exchange by the SMF to Naturalis dated 6 June 1828 (Naturalis archives inv. 241.880), so that the third specimen might be the animal collected in 1831. This cannot be traced and therefore all our skins are included in the type series here. Jentink did not mention the skulls without skins, labelled “Egypt”, as types, but it seems likely that these were taken from the two skins lacking skulls. Therefore, they are included in the type series here.

Mertens (1925: 26) designated a specimen in the SMF (SMF 4326) as the lectotype. See Mertens (1925: 19) for the publication dates of Cretzschmar’s work.

***Pseudomysaustralis* Gray, 1832**

*Muslineolatus* Gould, 1845: 77.

Paralectotype, RMNH.MAM.20347 (Jentink 1887: 213 *a*; 1888: 67 *b*), adult, sex unknown, mounted skin and skull. Loc.: Plains of Darling [Plains on the Darling Downs], Australia. Ex: J. Gould.

It is doubtful that Gould himself was the collector of this specimen (as presumed by Jentink 1887; 1888) as he never reached this area of Australia. The collector could have been John Gilbert, who visited this area in 1844.

The lectotype, a specimen in the NHM (NHMUK 58.11.24.4), was designated by Thomas (1921: 432).

***Rattusadustus* Sody, 1940**

*Rattusadustus* Sody, 1940: 397.

Holotype, RMNH.MAM.9811, adult female, skin and skull. Loc.: Kiojoh, Enggano, Sumatra, Indonesia. Leg.: Saan, 15 June 1936. Ex: MZB (MZB 304/36; 5827).

*Rattusrattusbali* Kloss, 1921b: 123.

Syntype, RMNH.MAM.9805, adult male, skin and skull. Loc.: Kloengkoeng, Bali, Indonesia. Leg.: 01 May 1915. Ex: MZB (MZB 199)

Syntype, RMNH.MAM.9806, adult female, skin and skull. Loc.: Laboean Amok, Bali, Indonesia. Leg.: 05 July 1915. Ex: MZB (MZB 200).

Kloss (1921b) designated two co-types but confused the associated data. In his description of the types he referred to a male from Laboean Amok, 5 July 1915, no. 99 and a female from Kloengkoeng, 1 May 1915, no. 100. The original labels from the MZB marked “co-type’’ describe a female, no. 200 from Laboean Amok, 5 July 1915 (RMNH.MAM.9806) and a male, no. 199 from Kloengkoeng, 1 May 1915 (RMNH.MAM.9805).

***Rattusrattussaturnus* Sody, 1941: 269.**

Holotype, RMNH.MAM.9808, adult female, skin and skull. Loc.: Melolo, Sumba, Indonesia. Leg.: G. Stein, 15 June 1932. Ex: MZB (MZB 4943; 265/35).

***Rattuselaphinus* Sody, 1941**

*Rattuselaphinus* Sody, 1941: 307.

Holotype, RMNH.MAM.9799, adult male, skin and skull. Loc.: Taliabu Island, Sulawesi, Indonesia. Leg.: J.J. Menden, 23 October 1938. Ex: MZB (MZB 4087; 347/38).

Sody (1941) based his description on 12 specimens, only the holotype is in Naturalis.

***Rattusexulans* (Peale, 1848)**

*Musephippium* “Temminck” Jentink, 1880c: 15.

Holotype by monotypy, RMNH.MAM.18345 (Jentink 1887: 211 *a*; 1888: 64 *a*), adult male, relaxed mount and skull. Loc.: Sumatra, Indonesia.

*Musjessook* Jentink, 1880c: 15.

Lectotype, RMNH.MAM.19202 (Jentink 1887: 213 *a*; 1888: 68 *a*), adult female, mounted skin and skull. Loc.: Vanuatu, ‘Nouvelles Hebrides’, Tanna. Leg.: unknown. Received 1860.

Paralectotype: RMNH.MAM.19203.

Jentink (1880c) mentioned having two specimens. In his catalogue (Jentink 1887: 213: cat. *a*) he listed the skull of RMNH.MAM.19202 as the type, thereby designating it the lectotype.

*Muswichmanni* Jentink, 1890a: 120.

Holotype, RMNH.MAM.37695, adult male, skull and alcohol. Loc.: Sikka, Flores, Indonesia. Leg.: M. Weber (518), [​​1–9 January] 1889.

Paratype: ZMA.MAM.27231.

Another specimen in Naturalis (ZMA.MAM.12671, immature male from Kotting, Flores collected by Weber in November/December 1888, no. 514) was not mentioned by Jentink (1890a) and is therefore not included in the type series. Weber visited Flores from 21 November 1888 until 9 January 1889.

*Epimysstragulum* Robinson & Kloss, 1916: 274.

Paratype, RMNH.MAM.23721, male, skin, skull missing. Loc.: Mt. Kerinci, Sumatra, Indonesia. Leg.: H.C. Robinson and C.B. Kloss (6366), 6 May 1914. Ex: H.J.V. Sody.

Robinson and Kloss (1916) examined ten specimens; the holotype is in the Federated Malay States Museums (no. 482/14).

*Rattusschuitemakeri* Sody, 1933: 431.

Holotype, RMNH.MAM.22586, adult female, skin and skull. Loc.: Pontianak, Borneo, Indonesia. Leg.: H.J.V. Sody (12), July 1932.

*Rattusconcolorlassacquèrei* Sody, 1933: 433.

Holotype, RMNH.MAM.22575, adult female, skin and skull. Loc.: Manokwari, Anggi Guyi, New Guinea, Indonesia. Leg.: G. de Lassacquère, 28 January 1933. Ex: H.J.V. Sody (N.G.51).

Paratypes: RMNH.MAM.22576–22578.

*Rattusconcolormanoquarius* Sody, 1934: 175.

Holotype, RMNH.MAM.22580, adult male, skin and skull. Loc.: Amberbaki, New Guinea, Indonesia. Leg.: Z.L. Litaay, December 1932. Ex: H.J.V. Sody (N.G.57).

Paratypes: RMNH.MAM.22581–22585.

*Rattusconcolormalengiensis* Sody, 1941: 281.

Holotype, RMNH.MAM.9793, adult male, skin and skull. Loc.: Malengi, Togian Islands, Indonesia. Leg.: J.J. Menden, 29 November 1939. Ex: MZB (MZB 4749; 51/40).

According to Sody (1941) he had eight specimens available; only the holotype is in Naturalis.

*Rattusconcolormeringgit* Sody, 1941: 283.

Holotype, RMNH.MAM.9794, adult male, skin and skull. Loc.: Batoe-Meringgit, Bali, Indonesia. Leg.: P.F. Franck, 9 October 1928. Ex: MZB (MZB 1981).

Sody (1941) examined three specimens; only the holotype is in Naturalis. Batoe-Meringgit cannot be found on any map but possibly refers to Baturinggit.

***Rattusfuscipes* (Waterhouse, 1839)**

*Musfuscipes* Waterhouse, 1839: 66, pl. 25.

Although Jentink in his catalogues (Jentink 1887: 213; 1888: 67) listed a specimen in Naturalis (RMNH.MAM.20354, from ‘King George Sound’ by J. Gould) as the type of *Musfuscipes*, this is most unlikely. Waterhouse had only one specimen at the time of description (the holotype). This specimen ended up in the British Museum and eventually was lost.

The specimen in Naturalis is most likely not this lost holotype as it does not agree with the plate. Furthermore, Jentink (1887; 1888) in all likelihood misinterpreted the reference to the title of the publication by Waterhouse (1839) as the collection event. This is corroborated by an undated document in the Naturalis archives (inv. 208.299) which lists birds and mammals received by Gould in 1840, among which: “*Musfuscipes* Waterh. Zool. of Beagle”. The reference is clearly to the publication and not to the origin of the specimen.

Taylor and Horner (1967) discussed the Naturalis specimen as “a topotypical mounted specimen”. They mentioned Gould’s undated list in the archives but overlooked the name “Gould” on the stand and did not discuss the information on the label. Although they did not exclude the possibility that RMNH.MAM.20354 was the lost holotype of *Musfuscipes*, they designated another specimen in the Western Australian Museum (M6634) as neotype, mainly because of the bad condition of the Naturalis skull.

***Rattushoffmanni* (Matschie, 1901)**

*Rattusbiformatus* Sody, 1941: 306.

Holotype, RMNH.MAM.9797, adult female, skin and skull. Loc.: Malengi, Togean Islands, Indonesia. Leg.: J.J. Menden, 14 December 1939. Ex: MZB (MZB 5889; 61/40).

Sody (1941) mentioned having seven specimens of this new taxon; only the holotype is in Naturalis.

***Rattushoogerwerfi* Chasen, 1939**

*Rattushoogerwerfi* Chasen, 1939b: 207.

Holotype, RMNH.MAM.9824, adult female, skin and skull. Loc.: Blang Kedjeren, Sumatra, Indonesia. Leg.: A. Hoogerwerf, 29 January 1937. Ex: MZB (MZB 4854; 311/37).

Chasen (1939b) referred to four specimens in his description; only the holotype is in Naturalis.

***Rattusleucopusratticolor* (Jentink, 1909)**

*Musratticolor* Jentink, 1909a: 3, 7.

Holotype by monotypy, RMNH.MAM.25743, subadult female, skin and skeleton. Loc.: Van Weel’s Kamp, Lorentz River, New Guinea, Indonesia. Leg.: H.A. Lorenz, 18 June 1907, Nieuw Guinea Expeditie 1907 (84).

Some sources (Musser and Carleton 2005: 1473; Denys et al. 2020: 478) date the publication of this name to 1908, based on the imprint date October 1908 in the header of the article; however, the first issue of Nova Guinea 9 was published in 1909 (see title page).

***Rattusmarmosurus* Thomas, 1921**

*Rattustondanus* Sody, 1932b: 158.

Holotype, RMNH.MAM.21234, adult female, skin and skull. Loc.: Tondano, Sulawesi, Indonesia. Leg.: H.J.V. Sody, March 1932.

***Rattusnitidus* (Hodgson, 1845)**

*Musaequicaudalis* Hodgson, 1849a: 203.

Jentink (1888: 63) listed a specimen in Naturalis (RMNH.MAM.19205, from Tibet by B.H. Hodgson) as one of the types. However, Hodgson (1849a) described this species based on specimens collected in India, therefore RMNH.MAM.19205 is not a type.

*Musruber* Jentink, 1880c: 18.

Holotype by monotypy, RMNH.MAM.26067 (Jentink 1887: 213 *a*; 1888: 67 *a*), adult female, mounted skin and skull. Loc.: Doreh, New Guinea, Indonesia. Leg.: C.B.H. von Rosenberg, January 1869.

For details of this specimen and taxonomic name see Calaby and Taylor (1980).

***Rattusvanheurni* Sody, 1933: 435.**

Holotype, RMNH.MAM.21704, female, skin and skull. Loc.: Manokwari, New Guinea, Indonesia. Leg.: Z.L. Litaay, October 1932. Ex: H.J.V. Sody (N.G.6).

Paratypes: RMNH.MAM.21705–21710.

Sody (1933) based his description on seven specimens. There is some confusion about who collected these specimens and when. According to Becking (1989: 68) at least the holotype was collected by Van Heurn during the Memberano Expedition of 1920. Becking was probably misled by the dedication by Sody of this species to Van Heurn, acknowledging Van Heurn’s important collecting work during this expedition and referring to duplicates in Sody’s own collection. But the original label attached to the specimen is a Sody collection label and the number, N.G.6, is part of the range of specimens collected in October 1932 by Z.L. Litaay in Manokwari (original data by Sody, Naturalis archives inv. 11) and received by Sody on 22 November 1932. We follow this.

***Rattuspelurus* Sody, 1941**

*Rattusforamineuspelurus* Sody, 1941: 308.

Holotype, RMNH.MAM.9800, adult female, skin and skull. Loc.: Peleng, Indonesia. Leg.: J.J. Menden, 19 August 1938. Ex: MZB (MZB 5620).

Sody (1941) examined two specimens for his description; only the holotype is in Naturalis.

***Rattusrattus* (Linnaeus, 1758)**

*Muserythronotus* Temminck, 1844: 50.

Syntype, RMNH.MAM.37249 (Jentink 1888: 64 *a*), adult [male], mounted skin and damaged skull. [Nagasaki, Kyūshū], Japan, [1840–1841]. Received from P.-M. Diard, 1841.

Temminck (1844: 50) had two specimens preserved in alcohol at his disposal. Of those, only one, now mounted, is still present in Naturalis. The specimens must have been among the material obtained by two unknown Javan taxidermists of the museum in Batavia (Jakarta). They were sent to Japan by Diard, to stay for a year at what he calls “Mangazaka”, no doubt meaning Nagasaki, the port in which the Dutch trading post of Deshima was situated. The shipment was forwarded to Leiden in May 1841 (see Diard’s letter to Temminck of 25 May 1841; Naturalis archives inv. 205.227), so the animals were most likely collected in 1840. Temminck suspected that the species had been introduced from China.

Tate (1940: 6), probably unaware of the fact that Temminck had two animals before him, referred to the Naturalis specimen as “Type”. This does not qualify as a valid lectotype designation according to ICZN art. 74.5 and all types should be treated as syntypes.

*Musalbiventer* Jentink, 1909b: 240.

*Musalbiventris* Jentink, 1909b: 247 (incorrect original spelling).

Holotype by monotypy, RMNH.MAM.27426, adult female, skull and alcohol. Loc.: Mosselbaai, South Africa. Leg.: M. Weber (184), [1894–1895].

Jentink (1909b) described this species as *Musalbiventer*, but later in the text spelled the name (once) as *albiventris*.

***Rattussteinifoersteri* Rümmler, 1935**

*Stenomysverecundusförsteri* Rümmler, 1935: 117.

Holotype, RMNH.MAM.54930, adult female, skin and skull. Loc.: Bulung River (source area), Papua New Guinea (1800–2000m). Leg.: F. Förster (292/2), 22 February 1913. Ex: Zoologisches Museum Berlin.

Paratype: RMNH.MAM.54931.

***Rattustanezumi* (Temminck, 1844)**

*Mustanezumi* Temminck, 1844: 51, pl. 15 figs 5–7.

Lectotype, RMNH.MAM.24207 (Jentink 1887: 210 *b*; 1888: 63 *b*), juvenile, sex unknown, mounted skin and damaged skull. Loc.: Japan. Leg.: [Ph.F.B. von Siebold or H. Bürger, 1823–1831]. Figured in Temminck (1844: pl. 15 figs 5–7).

Paralectotypes: RMNH.MAM.24206, 27421.

Jentink (1887; 1888) catalogued two skulls *a* and *b* as belonging to the respective skins. The identity of skull *a* (RMNH.MAM.27421) is unknown; it cannot have belonged with the above skin *a* (RMNH.MAM.24206), the skull of which was extracted in the 20^th^ century.

Jentink attributed both specimens to von Siebold. However, “Bürger” is written on the label and pedestal of RMNH.MAM.24207, whereas those of RMNH.MAM.24206 did not mention the collector. In the archives of Naturalis two consignments by Bürger are described (from 20 December 1831 and 31 December 1831) containing three specimens of this species. At least RMNH.MAM.24206 must have been one of those three. The precise provenance of the material cannot be traced.

The lectotype was designated by Tate (1940: 6).

*Musdiardii* Jentink, 1880c: 13.

*Musrattusbrevicaudatus* Horst & De Raadt, 1918: 69. (nomen novum)

Holotype by monotypy, RMNH.MAM.22891 (Jentink 1887: 211 *a*; 1888: 65 *a*), adult, sex unknown, mounted skin and skull. Loc.: W. Java, Indonesia. Leg.: P. Diard, 1864.

When the plague was diagnosed in Java in 1911, Dutch colonial health officials, led by J.J. van Loghem, tried to identify the particular species of rodents responsible for the transmission of the disease. To aid with these identifications Horst and De Raadt studied specimens of the Javan rodent species in the Naturalis collection.

They identified the type specimen of *Musdiardii* Jentink, 1880 as a Javan houserat, which until then was named *Musrattusgriseiventer* Bonhote, 1903. As Jentink’s name has priority over Bonhote, the valid name for the Javan houserat should in their opinion be *Musrattusdiardii* Jentink, 1880. This leaves the Javan field(sawah)rat (until then considered to be *diardii*, see De Beaufort 1913: 32) without a name, for which Horst and De Raadt (1918) proposed *Musrattusbrevicaudatus* as a new name.

Musser and Carleton (2005: 1465) listed *Musrattusbrevicaudatus* Horst & De Raadt, 1918 as a synonym of *Rattusargentiventer* (Robinson & Kloss, 1916). Being a nomen novum for *diardii* Jentink, 1880, this name should be a synonym for *Rattustanezumi*.

*Musneglectus* Jentink, 1880c: 14.

Syntype, RMNH.MAM.39365 (Jentink 1887: 211 *a*; 1888: 65 *a*), adult female, mounted skin and skull. Loc.: Batjan, Indonesia. Leg.: H.A. Bernstein, 1861.

Syntype, RMNH.MAM.39366 (Jentink 1887: 211 *b*; 1888: 65 *b*), adult, sex unknown, mounted skin and skull. Loc.: Banjermassing, Borneo, Indonesia. Leg.: S. Müller, [28 July–17 December 1836].

Chasen (1940b: 152) only mentioned Banjermassin, South Borneo as the locality, implying a restriction of the type locality. However, Chasen failed to validly designate a lectotype (ICZN art. 74.5). Therefore we list the two specimens mentioned by Jentink (1880c: 14) as syntypes.

*Rattusrattussamati* Sody, 1932b: 159.

Holotype, RMNH.MAM.22215, adult male, skin and skull. Loc.: Buleleng, Bali, Indonesia. Leg.: A. Samat, 6 August 1932. Ex: H.J.V. Sody (E.123).

Paratypes: RMNH.MAM.22216–22231.

Sody (1932b) examined 19 specimens, of which the specimens listed above are in Naturalis.

*Rattusrattussantalum* Sody, 1932b: 159.

Holotype, RMNH.MAM.22568, adult male, skin and skull. Loc.: Waingapoe, Sumba, Indonesia. Leg.: H.J.V. Sody, 01 February 1932.

Sody (1932b) had only one specimen of this taxon in his collection but also referred to four other specimens in the MZB listed in Dammerman (1928: 309).

*Rattusmasaretes* Sody, 1937: 217.

Holotype, RMNH.MAM.22446, adult male, skin and skull. Loc.: Buru, Molukken (Moluccas), Indonesia. Leg.: December 1932. Ex: H.J.V. Sody (Boeroe 10).

*Rattusrattusmoluccarius* Sody, 1933: 437.

Holotype, RMNH.MAM.22439, adult male, skin and skull. Loc.: Buru, Molukken (Moluccas), Indonesia. Leg.: December 1932. Ex: H.J.V. Sody (Boeroe 9).

Paratypes: RMNH.MAM.22440–22445.

Sody (1933) had seven specimens from the type-locality and 24 from other Moluccan islands; it is unclear from his description whether the latter are included in this new subspecies. The specimens were probably collected by a local government officer.

*Rattusrattussepticus* Sody, 1933: 437.

Holotype, RMNH.MAM.22409, adult female, skin and skull. Loc.: Banda-Neira, Indonesia. Leg.: November 1932. Ex: H.J.V. Sody (Banda-Neira 1).

Paratypes: RMNH.MAM.22410–22416.

Sody (1933) examined eight specimens from the type locality and sixteen from neighbouring islands. The specimens were probably collected by a local government officer. Only the specimens listed above are in Naturalis.

*Rattusrattusauroreus* Sody, 1941: 264, 266.

Holotype, RMNH.MAM.9810, adult female, skin and skull. Loc.: North Pagai, Mentawai Islands, Indonesia. Leg.: J.J. Menden, 16 January 1935. Ex: MZB (MZB 47/35, MZB 5236), 12 January 1950.

*Rattusrattusmakassarius* Sody, 1941: 264, 266.

Holotype, RMNH.MAM.9813, adult female, skin and skull. Loc.: Macassar [Ujung Pandang], Sulawesi, Indonesia. Leg.: Eykman Institute (668), 1940. Ex: MZB (MZB 447/40, MZB 4222), 12 January 1950.

*Rattusrattuspelengensis* Sody, 1941: 264, 267.

Holotype, RMNH.MAM.9819, adult female, skin and skull. Loc.: Peleng, Sulawesi, Indonesia. Leg.: J.J. Menden, 19 July 1938. Ex: MZB (MZB 147/38, MZB 4158), 12 January 1950.

*Rattusrattusobiensis* Sody, 1941: 264, 268.

Holotype, RMNH.MAM.23179, adult female, skin and skull. Loc.: Laiwui, Obi Island, Molukken (Moluccas), Indonesia. Leg.: C. Limaheluw, November 1932. Ex: H.J.V. Sody. (Obi 7).

Paratypes: RMNH.MAM.23180–23211.

Sody (1941) designated a single specimen as the type, but also referred to additional material from Obi, Ternate, and possibly Halmahera. There is a large series of specimens in his collection labelled *Rattusrattusobiensis* by Sody from the Obi islands and Ternate; those we list here as paratypes.

*Rattusrattusargyraceus* Sody, 1941: 270.

Holotype, RMNH.MAM.9816, adult female, skin and skull. Loc.: Mapanget, Sulawesi, Indonesia. Leg.: Klapperproefstation (18), 15 July 1939. Ex: MZB (MZB 350/39, MZB 4045), 12 January 1950.

Sody (1941) based his description on eight specimens; only the holotype is in Naturalis.

*Rattusrattustalaudensis* Sody, 1941: 275.

Holotype, RMNH.MAM.9814, adult female, skin and skull. Loc.: Lirung, Talaud Island, Indonesia. Leg.: Eri, 29 May 1926. Ex: MZB (MZB 1488), 12 January 1950.

Sody (1941) referred to six specimens in his description; only the holotype is in Naturalis.

*Rattusrattusbarussanoides* Sody, 1941: 276.

Holotype, RMNH.MAM.9807, adult female, skin and skull. Loc.: Macassar (Ujung Pandang), Sulawesi, Indonesia. Leg.: Eykman Institute (39), 1940. Ex: MZB (MZB 376/40; 4039), 12 January 1950.

Sody (1941) had three specimens available; only the holotype is Naturalis.

*Rattussapoensis* Sody, 1941: 306.

Holotype, RMNH.MAM.9790, adult male, skin and skull. Loc.: Malengi Island, Togian Islands, Indonesia. Leg.: J.J. Menden, 02 December 1939. Ex: MZB (MZB 50/40; 5624), 12 January 1950.

Sody (1941) had three specimens available; only the holotype is Naturalis.

*Rattustoxi* Sody, 1941: 309.

Holotype, RMNH.MAM.9802, adult female, skin and skull. Loc.: Wadjo, Sulawesi, Indonesia. Leg.: “controleur”, 12 April 1915. Ex: MZB (MZB 95/41; 5632), 12 January 1950.

Musser (1971c) restricted the type locality to the village of Wadjo (4°03'S, 120°09'E).

***Rattustiomanicus* (Miller, 1900)**

*Rattusrattusroquei* Sody, 1929a: 163.

Lectotype, RMNH.MAM.21403, adult male, skin and skull. Loc.: Gedangan, Semarang, Java, Indonesia. Leg.: F.A.Th.A. Verbeek, 16 August 1928. Ex: H.J.V. Sody.

Paralectotypes, 21480–21486, 21492, 21530–21545, 21547–21549.

The short first publication of this name is in a footnote to a list of mammals of Java, where Sody (1929a) did not mention any specimens. This was followed by an extensive description in 1930 (Sody 1930a), wherein he made it clear he had 29 specimens in his own collection and referred to many more in several other collections. He designated a type that, according to the code, should be considered the lectotype. Sody failed to give a collection number of this specimen and later (Sody 1941: 273) stated that the type was untraceable. RMNH.MAM.21403 is probably the lectotype, as geographic data agrees with the description of 1930 and the measurements agree very well with Sody’s lectotype (Musser in litt., 1969, verified by the authors).

However there remains some uncertainty, as the collecting date on the label is 16 August 1928, while Sody’s lectotype was collected on 16 August 1924 (Sody 1930a: 97). Furthermore, the collector is not mentioned by Sody, but an original label by Verbeek is associated with the skull. Becking (1989: 65) added to the confusion by listing RMNH.MAM.21548 (Sody’s number “III” collected by Verbeek on 16 August 1924 in the same locality as RMNH.MAM.21403) as the holotype. However there is no information on its label supporting this assumption (except for Sody’s number and the locality of Semarang) and the measurements do not agree with those given by Sody (1930a: 97).

*Rattusrattusvernalus* Sody, 1940: 395.

Holotype, RMNH.MAM.9818, adult female, skin and skull. Loc.: Kayaapu, Enggano Island, Sumatra, Indonesia. Leg.: J.K. de Jong, July 1936. Ex: MZB (MZB 316/36; 4130), 12 January 1950.

Sody (1940) based his description on six specimens; only the holotype is in Naturalis.

*Rattusrattusdelirius* Sody, 1941: 270, 274.

Holotype, RMNH.MAM.9817, adult male, skin and skull. Loc.: Bantam, Java, Indonesia. Leg.: V. von Plessen, 20 February 1932. Ex: MZB (MZB 5/32; 4114), 12 January 1950.

*Rattusrattusgeneratius* Sody, 1941: 270, 274.

Holotype, RMNH.MAM.9812, adult male, skin and skull. Loc.: Noesa Kambangan, Java, Indonesia. Leg.: Eykman Institute (8), 1940. Ex: MZB (MZB 294/40; 5846), 12 January 1950.

Sody (1941) based his description on thirty specimens; only the holotype is in Naturalis.

*Rattusrattuslarusius* Sody, 1941: 270, 274.

Holotype, RMNH.MAM.9809, adult male, skin and skull. Loc.: Meeuweneiland (Pulau Peucang), Bantam, Indonesia. Leg.: P.F. Frank, 04 August 1932. Ex: MZB (MZB 12/32; 4120), 12 January 1950.

Sody (1941) based his description on three specimens; only the holotype is in Naturalis.

*Rattusrattussebasianus* Sody, 1941: 270, 273.

Holotype, RMNH.MAM.9815, adult male, skin and skull. Loc.: Sebesi Island, Indonesia. Leg.: K.W. Dammerman, 28 April 1921. Ex: MZB (MZB 60), 12 January 1950.

Sody (1941) based his description on two specimens; only the holotype is in Naturalis.

***Rattusxanthurus* (Gray, 1867)**

*Musfaberi* Jentink, 1883b: 176.

Holotype by monotypy, RMNH.MAM.18300 (Jentink 1887: 212 *a*; 1888: 66 *a*), adult, sex unknown, mounted skin and skull. Loc.: Menado, Sulawesi, Indonesia. Leg.: F. von Faber, 1883.

*Taeromysparaxanthus* Sody, 1941: 313.

Lectotype, RMNH.MAM.60471 (formerly 9822), adult male, skin. Loc.: Toelap, Sulawesi, Indonesia. Leg.: J.W. van Braekel, August 1938. Ex: MZB (MZB 341/38; 4043), 12 January 1950. (=*Rattusxanthurus*).

Paralectotype: RMNH.MAM.60472 (=*Taeromyscallitrichus*).

This name is based on a composite specimen. The skull belongs to *Taeromyscallitrichus* and the skin belongs to *Rattusxanthurus*. Musser (1971a) designated the skin the lectotype.

***Sundamysmaxi* (Sody, 1932)**

*Rattusmaxi* Sody, 1932b: 157.

Holotype, RMNH.MAM.21477, adult male, skin and skull. Loc.: Mt. Patuha, 1350 m., Tjiboeni (Cibuni), Bandung, Java, Indonesia. Leg.: M. Bartels Jr., 09 August 1932. Ex: H.J.V. Sody (Tjib.13).

Paratype: RMNH.MAM.21478.

***Sundamysmuelleri* (Jentink, 1880)**

*Musmülleri* Jentink, 1880c: 16.

Holotype by monotypy, RMNH.MAM.18347 (Jentink 1887: 211 *a*; 1888: 64 *a*), subadult female, relaxed mount and skull. Loc.: Batang Singalan, Sumatra, Indonesia. Leg.: S. Müller, [May–July 1834].

Müller was on a collecting trip in the area of Mount Singgalang between May and November 1834.

*Rattusmuelleriwaringensis* Sody, 1941: 289.

Holotype, RMNH.MAM.9796, adult male, skin and skull. Loc.: Kp. Riam, Borneo, Indonesia. Leg.: J.J. Menden, 04 November 1935. Ex: MZB (MZB 52/36; 5002), 12 January 1950.

Sody (1941) gave measurements for six specimens of this new taxon; only the holotype is in Naturalis.

***Taeromyscallitrichus* (Jentink, 1879)**

*Muscallitrichus* Jentink, 1879a: 12.

Lectotype, RMNH.MAM.21275 (Jentink 1887: 212 *a*; 1888: 65 *a*), adult, sex unknown, mounted skin and cranium. Loc.: Menado-Kakas, Sulawesi, Indonesia. Leg.: S.C.J.W. van Musschenbroek, September 1875.

Paralectotypes: RMNH.MAM.21276–21277 (= *Taeromyscallitrichus*), 21278–21281 (= *Bunomysfratrorum*), 21282 (= *Paruromysdominator*), 21283–21285 (= *Bunomyschrysocomus*), 21286 (= *Rattushoffmanni*).

According to Musser (1969) several species are represented in the type series of *Muscallitrichus*. Musser (1969: 6) designated RMNH.MAM.21275 the lectotype.

***Taeromystaerae* (Sody, 1932)**

*Rattustaerae* Sody, 1932b: 158.

Holotype, RMNH.MAM.22569, adult male, skin and skull. Loc.: Lembean, Sulawesi, Indonesia. Leg.: Corinus Deeng, March 1932 via H. Witkamp. Ex: H.J.V. Sody (P 72).

Paratypes: RMNH.MAM.22570–22571.

Sody (1932b) examined five specimens for his description; only three are in Naturalis. According to Becking (1989: 89) the specimens were collected in the Lembean Forest reserve, east of Tondano.

*Taeromystatei* Sody, 1941: 313.

Lectotype, RMNH.MAM.54934 (9821), adult male, skin. Loc.: Toelap, Sulawesi, Indonesia. Leg.: J.W. van Braekel, 25 August 1938. Ex: MZB (MZB 340/38, MZB 5814), 12 January 1950.

Paralectotype: RMNH.MAM.54933 (= *Bunomysfratrorum*).

According to Musser (1971a) this name is based on a composite specimen: the skin belonging to *Taeromystaerae*, the skull to *Bunomysfratrorum*. He designated the skin (RMNH.MAM.54934) the lectotype. Sody (1941) also gave measurements of a female specimen, which is not in Naturalis.

***Thallomysnigricauda* (Thomas, 1882)**

*Musnigricauda* Thomas, 1882: 266.

According to Jentink (1888: 71) RMNH.MAM.26488 (female from Hountop River, South Africa collected by Andersson in 1862) is one of the types for *Musnigricauda* Thomas, 1882. However, according to Thomas (1882) he had only one specimen of this new species at his disposal. The online database of the NHM lists a specimen (NHMUK 1881.3.8.7, https://data.nhm.ac.uk/object/7eb31634-292d-4a7c-8dc9-d513b72a2bd4/1739750400000) that agrees with the data given by Thomas and has as old determination *Musnigricauda* Thomas. It has (later) been identified by ???? as *Thallomyspaedulcus* (Sundevall, 1846) and its type status is not indicated.

***Thallomyspaedulcus* (Sundevall, 1846)**

*Muspædulcus* Sundevall, 1846: 120.

Syntype, RMNH.MAM.26489 (Jentink 1887: 214 *b*; 1888: 71 *b*), subadult female, mounted skin and skull. Loc.: ‘Caffraria interior’ [South Africa]. Leg.: [J. Wahlberg]. Ex: NRM, C.J. Sundevall.

Syntype, RMNH.MAM.26490 (Jentink 1888: 71 *c*), juvenile, sex unknown, mounted skin, skull in situ. Loc.: ‘Caffraria interior’ [South Africa]. Leg.: [J. Wahlberg]. Ex: NRM, C.J. Sundevall.

According to Davis (1965: 127) the type locality was in the Magaliesberg area and has been provisionally fixed as “Crocodile Drift, Brits, Transvaal” [South Africa].

***Typomystrivirgatus* (Temminck, 1853)**

*Mustrivirgatus* Temminck, 1853: 159.

Lectotype, RMNH.MAM.19508 (Jentink 1888: 70 *a*), subadult, sex unknown, mounted skin, skull in situ. Loc.: Dabocrom, Côte d’Or [Ghana]. Leg.: H.S. Pel.

The lectotype was designated by Jentink (1888: 70).

***Uromyscaudimaculatusmultiplicatus* (Jentink, 1907)**

*Pogonomysmultiplicatus* Jentink, 1907a: 367.

Holotype by monotypy, RMNH.MAM.29315, adult male, flat skin and skeleton. Loc.: Sentani meer, New Guinea, Indonesia. Leg.: A. Wichmann, 18 April 1903, Noord Nieuw-Guinea Expeditie (1903) (Humboldt Bay Expedition).

#### ﻿﻿Anomaluridae Gervais, 1849

***Anomalurusbeecrofti* Fraser, 1853**

*Anomaluruslaniger* Temminck, 1853: 149.

Holotype by monotypy, RMNH.MAM.26757 (Jentink 1887: 180 *a*; 1888: 1 *a*), subadult female, relaxed mount and skull. Loc.: Guinea coast (coastal Ghana). Leg.: H.S. Pel.

***Anomaluruspeliipelii* (Schlegel & Müller, 1845)**

Pteromys (Anomalurus) pelii Schlegel & Müller, 1845a: 109.

Lectotype, RMNH.MAM.26761 (Jentink 1888: 1 *a*), adult male, mounted skin and skeleton. Loc.: Dabocrom, Côte d’Or [Ghana]. Leg.: H.S. Pel [February 1843].

Paralectotype: RMNH.MAM.26762.

According to Schlegel and Müller (1845a) Naturalis received three specimens from Pel; only two are still present. Schunke and Hutterer (2005: 326) designated the lectotype.

Largen (1985) listed a possible syntype (LIVCM 1981.270) in the World Museum Liverpool and referred to a specimen in the NHM (not in the online database) and MNHN (not in Rode 1943).

***Anomaluruspeliiperalbus* Schunke & Hutterer, 2005**

*Anomaluruspeliiperalbus* Schunke & Hutterer, 2005: 327.

Holotype, ZMA.MAM.21275, adult female, skin and skull. Loc.: Gueboua (5°59'N, 5°41'W), Ivory Coast. Leg.: L.J.R. Bellier (A9013), 20 October 1970.

Paratypes: ZMA.MAM.21264–21270, 21272–21274, 21276–21289, 21293–21315.

#### ﻿﻿Bathyergidae Waterhouse, 1841

***Heliophobiusargenteocinereusargentocinereus* Peters, 1846**

*Heliophobiusargento*-*cinereus* Peters, 1846: 259.

Syntype, RMNH.MAM.26672 (Jentink 1888: 93 *a*), sex unknown, mounted skin and skull. Loc.: Boror, Mozambique. Leg.: W.C.H. Peters, [1846]. Ex: MfN, W.C.H. Peters, 25 May 1951.

This species was first described in a preliminary publication, read in August 1846 on behalf of Peters for the Royal Prussian Academy of Sciences in Berlin. It preceded the extensive description in his definitive work (Peters 1852: 140–145, Taf. XXXI, XXXV). In neither publication is it specified how many animals Peters had before him, but both from the 1846 publication and in 1852 it is clear he collected several specimens.

Peters arrived in Mozambique in 1843 and returned to Germany in 1848 (Peters 1852: viii–ix); on p. 47 he writes that he was in Boror “etwa 12 Meilen nordwestlich von Quellimane” in March 1846, but does not say how long he stayed in the area. In the absence of more precise data, it seems best to regard all specimens collected by Peters as belonging in the type series. RMNH.MAM.26672 is mentioned in a list of specimens received from Peters on 25 May 1851, preserved in the archives of Naturalis (inv. 247.168), as “1 Heliophobius (Bathyergus) cinereus Jeune indiv”, although the animal looks quite adult.

#### ﻿﻿Hystricidae Fischer, 1817

***Atherurusmacrourus* (Linnaeus, 1758)**

*Atherurusretardatus* Mohr, 1964: 105.

Paratype, RMNH.MAM.19420, adult male, skin and skull. Loc.: imported from Singapore on 20 September 1959, died 12 January 1967. Ex: Rotterdam Zoo.

Paratypes: RMNH.MAM.19897, 20782; ZMA.MAM.5092.

Mohr (1964) examined six specimens; all imported by the Rotterdam Zoo. Of these, one specimen was sent through to Antwerp Zoo and another to NAM. The holotype is in the KBIN (the animal from Antwerp Zoo). ZMA.MAM.5092 is erroneously listed as “Mus. Amsterdam 5292” by Mohr (1964).

***Hystrixbrachyura* Linnaeus, 1758**

*Hystrixmülleri* Marshall in Sclater, 1871: 235 (footnote).

Syntype, RMNH.MAM.19964 (Jentink 1888: 104 *a*), adult female, mounted skin and skull. Loc.: Padang Bessie, Sumatra, Indonesia. Leg.: S. Müller, [1833–1835].

Syntype, RMNH.MAM.19966 (Jentink 1888: 104 *c*), subadult, sex unknown, mounted skin and skull.

Syntype, RMNH.MAM.19993 (Jentink 1887: 232 *a*), adult, sex unknown, skeleton. Loc.: Padang Bessie, Sumatra, Indonesia. Leg.: S. Müller, [1833–1835].

Marshall (1871) published this name in synonymy of *Hystrixlongicauda* in a footnote in an article by Sclater. Later Jentink (1879g: 87) fully described the species and thereby validated the name. Jentink assumed the specimens mentioned by Marshall are all from Naturalis. However, Marshall referred to specimens in “our gallery”, which was in London, and to the specimens in Naturalis collected in Sumatra under the manuscript name *H.muelleri*.

***Hystrixjavanica* Cuvier, 1823**

*Hystrixecaudata* van der Hoeven & de Vriese, 1836: 110 (nomen nudum). Van der Hoeven and De Vriese (1836) published this name without providing a valid description. However, Woods and Kilpatrick (2005: 1544) listed *ecaudata* in the synonymy of *H.javanica*.

*Hystrixtorquata* van der Hoeven & de Vriese, 1836: 110. Syntype, RMNH.MAM.19963 (Jentink 1888: 104.*d*) juvenile, sex unknown, mounted skin, skull in situ. Loc.: Java [Indonesia]. Leg.: H. Kuhl and J.C. van Hasselt [December 1820–September 1821] . Syntype, RMNH.MAM.19989 (Jentink 1887: 232 *g*) adult, sex unknown, skull. Loc.: Java [Indonesia]. Leg.: G. van Raalten [December 1820–1829].

Van der Hoeven and De Vriese (1836) based their description on four specimens in Naturalis. We only list the two specimens which were with certainty present in Naturalis when this name was published. Woods and Kilpatrick (2005: 1544) list *torquata* in the synonymy of *H.javanica*.

#### ﻿﻿Thryonomyidae Pocock, 1922

***Thryonomysswinderianus* (Temminck, 1827)**

*Aulacodusswinderianus* Temminck, 1827b: 248.

Holotype by monotypy, RMNH.MAM.25764 (Jentink 1887: 230 *a*; 1888: 102 *c*), juvenile, sex unknown, skin in alcohol and skeleton. Ex: Academy Groningen, T. van Swinderen.

According to Temminck (1827b) this specimen was received from the collection of the Groningen University without any additional information.

#### ﻿﻿Erethizontidae Bonaparte, 1845

***Coendouprehensilis* (Linnaeus, 1758)**

*Hystrixbrandtii* Jentink, 1879c: 96.

Lectotype, RMNH.MAM.19642 (Jentink 1887: 231 *b*; 1888: 103 *b*), adult female, mounted skin and skull. Loc.: Suriname. Leg.: H.H. Dieperink, 1835.

Paralectotype: RMNH.MAM.19643.

Jentink (1879c) based his description on three specimens of which two are in Naturalis. The third specimen is the skull figured by Brandt (1835: pl. 9, figs 5–9), collected by von Langsdorff in Brazil. According to Abramov and Baranova (2008: 47) this specimen is in the Zoological Institute of St. Petersburg, Russia (O.6593); they erroneously listed it as the holotype for *Hystrixbrandtii*. The lectotype was selected by Husson (1978: 484) and the type locality for *Hystrixbrandtii* is thereby restricted to Suriname.

#### ﻿﻿Chinchillidae Bennett, 1833

***Chinchillachinchilla* (Lichtenstein, 1829)**

*Chinchillabrevicaudata* Waterhouse, 1848: 241.

Syntype, RMNH.MAM.54935 (formerly 39393) (Jentink 1887: 233 *a*; 1888: 104 *a*), sex unknown, mounted skin and skull. Loc.: La Paz, Bolivia. Leg.: A. Dessalines d’Orbigny, [April–June 1833].

Syntype, RMNH.MAM.54936 (formerly 39394) (Jentink 1888: 105 *b*), female, mounted skin, skull extracted but not in collection. Loc.: Chile, no further documentation. Ex: F. Prévost.

The description by Waterhouse (1848) is based on three specimens identified as *Eriomyschinchilla*, one in the MfN (not seen by him) and two in Naturalis, which he measured himself. The spelling *brevicauda* as quoted by Woods and Kilpatrick (2005: 1550) and several earlier authors, is incorrect.

D’Orbigny collected in Bolivia between July 1830 and June 1833; he stayed in La Paz from 19 April to 27 June 1833. Here he arranged and packed his collections amassed during his various expeditions in the country and made some trips around La Paz (Papavero 1971: 139–143). Although the label (not original) of the skull of RMNH.MAM.54935 specifies “de la Paix” (from La Paz), that of the accompanying skin only reads “Bolivie”. Quite possibly, La Paz could indicate the town of acquisition or shipment rather than the collecting locality.

The animal obtained in or from Chile by Prévost is not further documented. Prévost was a dealer based in Paris. Naturalis acquired some mammals from him in 1835 and 1839.

***Chinchillalanigera* Bennett, 1829**

*Eriomyspellionum* van der Hoeven, 1830: 613.

Syntype, RMNH.MAM.63728 (Jentink, 1887: 233 *b*), juvenile, sex unknown, cranium. Loc.: Chile. No further data.

Van der Hoeven (1830) did not specify any specimens in his description, but he referred to a skull, later to be depicted (van der Hoeven 1831, pl. 2). He mentioned the skull lacks its mandibles, as does RMNH.MAM.63728.

***Lagostomusmaximusmaximus* (Desmarest, 1817)**

*Dipusmaximus* “Blainville” Desmarest, 1817a: 117.

*Lagostomustrichodactylus* Brookes, 1828: 102 (nomen novum).

Holotype by monotypy, RMNH.MAM.63731 (Jentink 1887: 233 *a*), sex unknown, skeleton. Loc.: “Chile”. Ex: Brookes Museum.

Desmarest (1817a) based his new species on an account by De Blainville of a living animal in a London menagerie. After its death the remains of this animal ended up in the Brookes collection (Brookes 1828: 102). Desmarest gave Australia as the provenance of this specimen, later changed to Chile by Brookes.

Brookes (1828) introduced the new genus *Lagostomus* and a new name for *Dipusmaximus*, also based on this specimen. In his opinion the epithet *maximus* was unjustified in a monotypic genus as there is no comparison in size to other species.

*Callomysviscacia* d’Orbigny & I. Geoffroy Saint-Hilaire, 1830: 289.

Syntype, RMNH.MAM.64139, (Jentink 1887: 233 *c*; 1888: 105 *b*), juvenile male, mounted skin and skull. Loc.: Patagonia, Chile. Leg.: A. d’Orbigny, January–September 1829.

The locality and collecting date are from Vénec-Peyré (2002: 317).

#### ﻿﻿Caviidae Fischer de Waldheim, 1817

***Kerodonrupestris* (Wied, 1820)**

*Caviarupestris* Wied, 1820b: 43.

Syntype, RMNH.MAM.17928 (Jentink 1888: 107 *b*), subadult male, mounted skin, skull in situ. Loc.: Brazil. Leg.: M. zu Wied, [1815–1817].

According to Wied (1820b) he encountered this species along the “Rio Grande de Belmonte, am Rio Pardo, am S. Francisco u.s.w.”. The type locality was restricted to Rio Grande de Belmonte, Bahia, Brazil by Cabrera (1958: 580).

#### ﻿﻿Dasyproctidae (Bonaparte, 1838)

***Dasyproctaleporina* (Linnaeus, 1758)**

*Musleporinus* Linnaeus, 1758: 1: 59.

*Musaguti* Linnaeus, 1766: 80.

Neotype, RMNH.MAM.20752, adult female, skin and skull. Forest near the Peninika boarding-school near the confluence of the Peninika Creek and the upper Commewijne River, Suriname. Leg.: A. M. Husson and P. Staffeleu, 6 May 1963.

Husson (1978: 462–463) explained the priority of *Musleporinus* (Linnaeus, 1758) over *Dasyproctaaguti* (Linnaeus, 1776) and selected the neotype for *Musleporinus* Linnaeus, 1758. Voss et al. (2001: 144) selected the same specimen as the neotype for *Musaguti* Linnaeus, 1766.

## ﻿﻿Additional information

### Author ORCIDs

Steven van der Mije https://orcid.org/0000-0001-7620-3064

Pepijn Kamminga https://orcid.org/0000-0003-4296-3553

### Data availability

All of the data that support the findings of this study are available in the main text.

## References

[B1] Abramov AV Baranova GI (2008) The Langsdorff’s expedition to Brazil and its mammal collection kept in the Zoological Institute of St. Petersburg, Russia. Russian Journal of Theriology 7(1): 41–50. 10.15298/rusjtheriol.07.1.06

[B2] Adriani MA (1895) Willem Hendrik Woelders. Een levensbeeld uit de Zending op N.-Guinea. Hoenderloo, 35 pp.

[B3] Allen JA (1900) Note on the generic names Didelphis and Philander. Bulletin of the American Museum of Natural History 13: 185–190.

[B4] Allen GM (1939) A checklist of African mammals. Bulletin of the Museum of Comparative Zoölogy at Harvard College 83: 1–763.

[B5] Allman GJ (1866) On the Characters and Affinities of *Potamogale*, a Genus of Insectivorous Mammals. Transactions of the Zoological Society of London 6: 1–16. 10.1111/j.1096-3642.1866.tb00569.x

[B6] Alston ER (1877) On the Rodents and marsupials collected by the Rev. G. Brown in Duke-of-York Island, New Britain, and New Ireland. Proceedings of the scientific meetings of the Zoological Society of London for the year 1877: 123–127.

[B7] Alston ER (1879) On the *Acanthomysleucopus* Gray. Proceedings of the scientific meetings of the Zoological Society of London for the year 1879: 645–646. 10.1111/j.1096-3642.1879.tb02688.x

[B8] Anderson J (1879) Anatomical and zoological researches: comprising an account of the two expeditions to Western Yunnan in 1868 and 1875; and a monograph of the two cetacean genera, Platanista and Orcella. Vol. 1. Bernard Quaritch, London, 984 pp. 10.5962/bhl.title.55401

[B9] Audebert JB (1799) Histoire naturelle des Singes et Makis. Desray, Paris, 61 pls.

[B10] Barnett A (2005) *Cacajaomelanocephalus*. Mammalian species No. 776: 1–6. 10.1644/1545-1410(2005)776[0001:CM]2.0.CO;2

[B11] Bartels Jr M (1937a) A new rat from Java. Treubia 16: 45–46.

[B12] Bartels Jr M (1937b) On two new Muridae from Sumatra and another rat new to the Sumatran fauna. Natuurkundig Tijdschrift voor Nederlandsch Indië XCVII: 121–124.

[B13] Bartels Jr M (1938) A new subspecies of *Rattusbartelsii* (Jentink) from Central Java. Treubia 16(3): 323–324.

[B14] Bauer AM Denzer W Dubois A Entiauspe-Neto OM Frétey T Ohler A Pyron A (2024) The specimens of green anaconda, *Boamurina* Linnaeus, 1758 (Squamata, Serpentes, Boidae, *Eunectes*), from the collection of Albertus Seba, and recommendations for tracing historical specimens. Bionomina 38: 7–29. 10.11646/bionomina.38.1.2

[B15] Bechstein JM (1799) Thomas Pennant’s allgemeine Uebersicht der vierfüßigen Thiere. Aus dem Englischen übersetzt und mit Anmerkungen und Zusätzen versehen von Johann Matthäus Bechstein. Zweyter Band. Verlag des Industrie-Comptoir’s, Weimar, i–xi + 323–768. [pls 35–54]

[B16] Becking JH (1989) Henri Jacob Victor Sody (1892–1959). His life and work. A biographical and bibliographical study. E.J. Brill, Leiden, viii, 272 pp. 10.1163/9789004629707

[B17] Bergmans W (2011) An annotated list of mammal type specimens in the collection of the former Zoological Museum of the University of Amsterdam (1890–2010). Zoologische Mededelingen Leiden, 835–848.

[B18] Blyth E (1875) Catalogue of mammals and birds of Burma. Journal of the Asiatic Society of Bengal 2, extra number, i–xxiv, 1–167.

[B19] Boddaert P (1785) Elenchus animalium. Vol. I. Sistens Quadrupedia huc usque nota, eorumque varietates. Ad ductum Naturae, quantum fieri potuit disposita. C.R. Hake, Rotterodami, xxxviii + 174 pp. 10.5962/bhl.title.40283

[B20] Boeseman M (1970) The vicissitudes and dispersal of Albertus Seba’s zoological specimens Zoologische Mededelingen 44(13): 177–206.

[B21] Bonhote JL (1900) On the Squirrels of the Ratufa (Sciurus) bicolor Group. The Annals and magazine of natural history; zoology, botany, and geology 7(5): 490–499. 10.1080/00222930008678321

[B22] Boschma H (1943) Voorloopig verslag over het verzamelen van dieren gedurende de expeditie van het Koninklijk Nederlandsch Aardrijkskundig Genootschap naar Nieuw-Guinee in 1939. Tijdschrift van het Nederlandsch Aardrijkskundig Genootschap 60: 504–522. [pls 1, 2]

[B23] Bosman W (1704) Beschrijving van de Guinese Goud-Kust. Waar by gevoegt is, een net verhaal van den slavehandel; en een generale beschrijving van de Slave-Kust, of Ardrase Landen, mitsgaders een omlandse reis door den schrijver in den jare van 1698. gedaan, na Rio de Gabon, Cabo-Lopez di Gonsalvez, de eilanden van Sainct Tomé en Annaboa, en wederkomst op de Goud-Kust. Tweede deel. Anthony Schouten, Utrecht, 211 pp.

[B24] Brandt JF (1835) Mammalium Rodentium exoticorum novorum vel minus rite cognitorum Musei Academici Zoologici descriptiones et icones. Mémoires de l’Académie impériale des sciences de St.-Pétersbourg 3(2): 357–442. [pls VIII–X]

[B25] Broekhuizen S Camphuysen K Hoekstra B Van Laar V (2017) Chris Smeenk 1942–2017. Lutra 60(1): 43–54.

[B26] Brongersma LD (1936) On the subspecies of *Rhizomyssumatrensis* (Raffles) with some notes on related species. Zoölogische Mededeelingen Leiden 19: 137–164.

[B27] Brookes J (1828) On a new Genus of the Order Rodentia. Transactions of the Linnean Society 16: 95–104. [pl. IX] 10.1111/j.1095-8339.1829.tb00257.x

[B28] Brookes J (1828–1830) Brookesian Museum: The Museum of Joshua Brookes […] Consists of a Collection of Anatomical & Zoological Preparations, Vols. 1–2. Gold and Walton, London, 20 pp.

[B29] Bullock W (1819) Catalogue of the London Museum of Natural History, which will commence selling by auction, on Thursday, April 29, 1819, by Mr. Bullock, at the Egyptian Hall, Piccadilly. Part first–fifth. London, 165 pp.

[B30] Burbridge AA Short JC (2023) Burrowing Bettong, *Bettongialesueur*. In: Baker Gynther AM Ian C (Eds) Strahan’s Mammals of Australia (4th edn.). Reed New Holland Publishers, Wahroonga, NSW, 291–293.

[B31] Burgin CJ Wilson DE Mittermeier RA Rylands AB Lacher TE Sechrest W (Eds) (2020) Illustrated Checklist of the Mammals of the World. Vol. 1–2. Lynx Editions, Barcelona. 1165.

[B32] Büttikofer J (1884) Mededeelingen over Liberia. Resultaten van eene onderzoekingsreis door J. Büttikofer en C.F. Sala in de jaren 1879–1882. Bijbladen van het Tijdschrift van het Aardrijkskundig Genootschap 12: i–xvi + 1–147. [map]

[B33] Büttikofer J (1885) Zoological researches in Liberia. A list of birds, collected by J. Büttikofer and C.F. Sala in Western Liberia. Notes from the Leyden Museum 7: 129–255. [pls 6, 7]

[B34] Büttikofer J (1888) Zoological researches in Liberia. A list of birds, collected by the author and Mr. F.X. Stampfli during their last sojourn in Liberia. Notes from the Leyden Museum 10: 59–106. [pl. 5]

[B35] Büttikofer J (1890a) Reisebilder aus Liberia. Resultate geographischer, naturwissenschaftlicher und ethnographischer Untersuchungen während der Jahre 1879–1882 und 1886–1887. I. Band. Reise- und Charakterbilder. E.J. Brill, Leiden, xv + 440 pp. [18 pls, map] 10.1163/9789004601239

[B36] Büttikofer J (1890b) Reisebilder aus Liberia. Resultate geographischer, naturwissenschaftlicher und ethnographischer Untersuchungen während der Jahre 1879–1882 und 1886–1887. II. Band. Die Bewohner Liberia’s. Thierwelt. E.J. Brill, Leiden, viii + 510 pp. [table, pls XIX–XXXII] 10.1163/9789004601246

[B37] Büttikofer J (1911) On a probably new species of the genus Cercopithecus. Notes from the Leyden Museum 34: 1–3.

[B38] Büttikofer J (1917) Die Kurzschwanzaffen von Celebes. Zoologische Mededeelingen Leiden 3: 1–86.

[B39] Cabrera A (1957–1961) Catálogo de los mamíferos de América del Sur. 2 vols. Buenos Aires, xxii + 732 pp.

[B40] Calaby JH Richardson (1988) Potoroidae. In: Walton DW (Ed.) Zoological Catalogue of Australia, 5.Mammalia. Australian Government Publishing Service, Canberra, 53–59. 10.1163/9789004611450_014

[B41] Calaby JH Taylor JM (1980) Re-evaluation of the holotype of *Musruber* Jentink, 1880 (Rodentia: Muridae) from Western New Guinea (Irian Jaya). Zoologische Mededelingen 55(18): 215–219.

[B42] Carleton MD Smeenk C Angermann R Goodman SM (2014) Taxonomy of nesomyine rodents (Muroidea: Nesomyidae: Nesomyinae): Designation of lectotypes and restriction of type localities for species-group taxa in the genus *Nesomys* Peters. Proceedings of the Biological Society of Washington 126(4): 414–455. 10.2988/0006-324X-126.4.414

[B43] Cerqueira R Tribe CJ (2007) Genus Didelphis Linnaeus, 1758. In: Gardner AL (Ed.) Mammals of South America, vol.1. Marsupials, xenarthrans, shrews, and bats. The University of Chicago Press, Chicago/London, 17–25.

[B44] Chasen FN (1939a) Four new mammals from Java. Treubia 17: 185–188. 10.14203/treubia.v17i3.2565

[B45] Chasen FN (1939b) Two new mammals from North Sumatra. Treubia 17: 207. 10.14203/treubia.v17i3.2569

[B46] Chasen FN (1940a) The mammals of the Netherlands Indian Mt. Leuser expedition 1937 to North Sumatra. Treubia 17: 479–502. 10.14203/treubia.v17i5.2592

[B47] Chasen FN (1940b) A handlist of Malaysian mammals (A systematic list of the mammals of the Malay Peninsula, Sumatra, Borneo and Java, including the adjacent small islands). Bulletin Raffles Museum Singapore, Straits Settlements 15: xx + 209 pp. [1 map]

[B48] Chasen FN Kloss CB (1928) On some Carnivora, Rodentia and Insectivora principally from Eastern Borneo. Journal of the Malay Branch of the Royal Asiatic Society 6(1): 38–49.

[B49] Collett R (1895) On a new Pseudochirus from N.W. Australia. Zoologischer Anzeiger 18: 464–468.

[B50] Corbet GB Hanks J (1968) Revision of the elephant-shrews, family Macroscelidae. Bulletin of the British Museum (Natural History). Zoology 16(2): 45–111. [1 pl.]

[B51] Corbet GB Hill JE (1992) The mammals of the Indomalay region: a systematic review. Natural History Museum Publications, Oxford University Press, Oxford, vii + 488 pp.

[B52] Cretzschmar PJ (1826–1828) Säugethiere. In: Cretzschmar, P.J. (1826–1830) Atlas zu der Reise im nördlichen Afrika von Eduard Rüppell. Heinrich Ludwig Brönner, Frankfurt am Main, 78 pp. [30 pls]

[B53] Cruz M Bergmans W Takada T Shiroishi T Yoshiki A (2024) Type specimens, taxonomic history, and genetic analysis of the Japanese dancing mouse or waltzer, *Muswagneri* variety *rotans* Droogleever Fortuyn, 1912 (Mammalia, Muridae). ZooKeys 1200: 27–39. 10.3897/zookeys.1200.118823 38736700 PMC11082488

[B54] D’Orbigny AD (1844) Voyage en Amérique méridionale. Mammifères Vol. 3 (1). Bertrand, Paris, 464 pp. [21 pls]

[B55] D’Orbigny AD (1835–1847) Voyage en Amérique méridionale. Mammifères Vol. 4 (1,2). Bertrand, Paris, 362 pp.

[B56] Dammerman KW (1928) On the Mammals of Sumba. Treubia 10(2–3): 299–315.

[B57] Davis DHS (1965) Classification problems of African Muridae. Zoologica Africana 1: 121–145. 10.1080/00445096.1965.11447305

[B58] De Ávila Pires FD (1965) The type specimens of Brazilian mammals collected by Prince Maximilian zu Wied. American Museum Novitates 2209: 1–21.

[B59] De Beaufort LF (1913) Rapport omtrent een onderzoek van door Dr. J. J. van Loghem in 1911 op Java verzamelde ratten. Mededeelingen van den burgelijken geneeskundigen dienst 2(2): 5–14.

[B60] De Beaufort F (1966) Catalogue des Types de Mammifères du Muséum national d’Histoire naturelle, Paris. VI. Monotremata. VII. Marsupialia. Bulletin du Muséum national d’histoire naturelle 38(5): 509–553.

[B61] De Buffon (1789) Histoire naturelle, générale et particulière, servant de suite a l’histoire des animaux quadrupedes. Vol. 13. L’Imprimerie Royale, Paris, 320 pp. [48 pls] 10.5962/bhl.title.51327

[B62] De Selys-Longchamps E (1841) Note sur le *Musagrestis* de Linné. Bulletins de l`Académie Royale des Sciences et Belles-Lettres de Bruxelles 8: 234–237.

[B63] Delessert A (1843) Souvenirs d’un voyage dans l’Inde exécuté de 1834 á 1839. Fortin, Masson et Cie, Langlois et Leclercq, Paris, 107 pp. [35 pls] 10.5962/bhl.title.34148

[B64] Denys C Taylor PJ Aplin KP Burgin CJ Fabre P-H Haslauer R Woinarski JCZ Breed WG Menzies JI (2020) Family Muridae. In: Burgin CJ Wilson DE Mittermeier RA Rylands AB Lacher TE Sechrest W (Eds) Illustrated Checklist of the Mammals of the World.Lynx Editions, Barcelona, 440–541.

[B65] Desmarest AG (1817a) Première Espèce. – La Grande Gerboise, *Dipusmaximus*, Blainv. (Mém. sur une nouvelle espèce de Gerboise, et Illustr. de ce genre.) In: Nouveau dictionnaire d’histoire naturelle, appliquée aux arts, à l’agriculture, à l’économie rurale et domestique, à la médicine, etc. par une société de naturalistes et d’agriculteurs. Nouvelle édition presqu’entièrement refondue et considérablement augmentée; avec des figures tirées des trois règnes de la nature. Tome XIII. Deterville, Paris, 117–119.

[B66] Desmarest AG (1817b) Kanguroo, *Kangurus*, Geoffr. [...]. In: Nouveau dictionnaire d’histoire naturelle, appliquée aux arts, à l’agriculture, à l’économie rurale et domestique, à la médicine, etc. par une société de naturalistes et d’agriculteurs. Nouvelle édition presqu’entièrement refondue et considérablement augmentée; avec des figures tirées des trois règnes de la nature. Tome XXV. Deterville, Paris, 28–44.

[B67] Desmarest AG (1822) Mammalogie ou Description des espèces de mammifères. Vol. 2. Mme. Veuve Agasse, Paris. i–viii + 277–556.

[B68] Desmarest AG (1826) RAT, Mus. (Mamm.). In: Cuvier F. Dictionnaire des sciences naturelles, dans lequel on traite méthodiquement des différens êtres de la nature, considérés soit en eux-mêmes, d’après l’état actuel de nos connoissances, soit relativement à l’utilité qu’en peuvent retirer la médecine, l’agriculture, le commerce et les arts. Suivi d’une biographie des plus célèbres naturalistes. Tome quarante-quatrième. F.G. Levrault, Strasbourg, Paris & Le Normant, Paris, 468–486.

[B69] Dickinson EC (2005) Some bibliographical findings on Notes from the Leyden Museum. Zoologische Mededelingen Leiden 79–2 (1): 1–7.

[B70] Dieterlen F Turni H Marquart K (2013) Type specimens of mammals in the collection of the Museum of Natural History Stuttgart. Stuttgarter Beiträge zur Naturkunde A 6: 291–303. 10.18476/sbna.v6.a3

[B71] Dorai F Low MEY (2021) Diard & Duvaucel: French Natural History Drawings of Singapore and Southeast Asia, 1818–1820. Embassy of France and National Library Board, Epigram Books, 188 pp.

[B72] Dorr LJ (1997) Plant collectors in Madagascar and the Comoro Islands. A biographical and bibliographical guide to individuals and groups who have collected herbarium material of algae, bryophytes, fungi, lichens, and vascular plants in Madagascar and the Comoro Islands. The Trustees, Royal Botanical Gardens, Kew, xlvi + 524 pp.

[B73] Droogleever Fortuyn, AB (1912) Über den systematischen Wert der japanischen Tanzmaus (*Muswagneri varietas rotans* nov. var.). Zoologischer Anzeiger 39(5/6): 177–190.

[B74] Eldridge M Coulson G (2020) Family Macropodidae. In: Burgin CJ Wilson DE Mittermeier RA Rylands AB Lacher TE Sechrest W (Eds) Illustrated Checklist of the Mammals of the World.Lynx Editions, Barcelona, 29–105.

[B75] Elliot DG (1913a) A review of the Primates. Monograph No. 1 vol. 1. Lemuroidea, Daubentonia to Indris. Anthropoidea, Seniocebus to Saimiri. American Museum of Natural History, New York, cxxvi + 317 pp [xxxviii, 11 pls]

[B76] Elliot DG (1913b) A review of the Primates. Monograph series vol. II. Anthropoidea. Aotus to Lasiopyga. American Museum of Natural History, New York, xviii + 382 pp. [xxvi, 8 pls]

[B77] Engelberger S (2010) Annotated catalogue of primate type specimens in the mammal collection of the Museum of Natural History Vienna. Diplomarbeit, University of Vienna. Fakultät für Lebenswissenschaften, 62 pp.

[B78] Engländer H (1995) Die Säugetierausbeute der Ostbrasilien-Expedition des Prinzen Maximilian zu Wied. Fauna und Flora in Rheinland-Pfalz Beiheft 17: 247–280.

[B79] Feiler A (1990) Über die Säugetiere der Sangihe- und Talaud-Inseln der Beitrag A.B. Meyers für ihre Erforschung (Mammalia). Zoologische Abhandlungen aus dem Staatlichen Museum für Tierkunde Dresden 46: 75–94.

[B80] Feiler A (1998) Ausgestorbene Säugetiere, Typusexemplare und bemerkenswerte Lokalserien von Säugetieren aus der Sammlung des Staatlichen Museums für Tierkunde Dresden (Mammalia). Zoologische Abhandlungen des Staatlichen Museums für Tierkunde Dresden 50: 401–416.

[B81] Fischer JB (1829) Synopsis Mammalium. Sumtibus J.G. Cottae, Stuttgardtiae, xlii + 952. [tables I–V]

[B82] Fisher RD Ludwig CA (2012) Catalog of Type Specimens of Recent Mammals: Rodentia (Sciuromorpha and Castorimorpha) in the National Museum of Natural History, Smithsonian Institution. Smithsonian Contributions to Zoology 640: 1–97. 10.5479/si.00810282.640.1

[B83] Flannery TF Groves CP (1998) A revision of the genus *Zaglossus* (Monotremata, Tachyglossidae), with description of new species and subspecies. Mammalia 62(3): 367–396. 10.1515/mamm.1998.62.3.367

[B84] Fooden J (1967) *Macacafuscata* (Blyth, 1875): proposed conservation as the name for the Japanese macaque (Mammalia). Z.N.(S.) 1802. The Bulletin of Zoological Nomenclature 24: 250–251. 10.5962/bhl.part.15410

[B85] Fooden J (1969) Taxonomy and evolution of the monkeys of Celebes (Primates: Cercopithecidae). Bibliotheca Primatologica 10: i–vii + 1–148.

[B86] Fooden J Aimi M (2005) Systematic review of Japanese macaques, *Macacafuscata* (Gray, 1870). Fieldiana: Zoology, new series 104: i–vi + 1–200. 10.5962/bhl.title.3500

[B87] Forbes A (1881) Mammalia. The Zoological Record 16: 1–28. Proceedings of the Biological Society of Washington 19: 51–56.

[B88] Förster F (1913) Neue Marsupialia vom Huongolf-Inland (Deutsch-Neuguinea). Zoologischer Anzeiger 42: 177–180.

[B89] Fransen CHJM Holthuis LB Adema JPHM (1997) Type-catalogue of the Decapod Crustacea in the collections of the Nationaal Natuurhistorisch Museum, with appendices of pre-1900 collectors and material. Zoologische Verhandelingen Leiden 311: i–xvi + 1–344.

[B90] Fraser L (1854) Description of a new species of Hyrax from Fernando Po. Proceedings of the Zoological Society of Londen 20: 99. [pl. XXXIII]

[B91] Gaimard JP (1823) Notice sur les Mammifères et les Oiseaux de la baie des Chiens-Marins et de la Nouvelle-Galles du Sud; sur leurs mœurs et leur distribution géographique. Annales des sciences naturelles 5: 476–491. 10.5962/bhl.part.3592

[B92] Garbino GSTG Costa HC (2015) Some nomenclatural notes regarding authorship and dates of New World monkeys (Primates: Platyrrhini). Sherbornia 2(3): 21–27.

[B93] Garbino GSTG Rezende GC Valadares-Padua C (2016) Pelage Variation and Distribution of the Black Lion Tamarin, *Leontopithecuschrysopygus*. Folia Primatologica 87: 244–261. 10.1159/000450998 27802443

[B94] Gardner AL (1993) Order Didelphimorphia. In: Wilson DE Reeder DM (Eds) Mammal species of the world.A taxonomic and geographic reference. Second edition. Smithsonian Institution Press, Washington/London, 15–23.

[B95] Gardner AL (2005) Order Didelphimorphia. In: Wilson DE Reeder DM (Eds) Mammal species of the world.A taxonomic and geographic reference. Third edition. Vol. 1. The Johns Hopkins University Press, Baltimore, 3–18.

[B96] Gardner AL Dagosto M (2007) Tribe Metachirini Reig, Kirsch, and Marshall, 1985. In: Gardner AL (Ed.) Mammals of South America, vol.1. Marsupials, xenarthrans, shrews, and bats. The University of Chicago Press, Chicago/London, 35–39. 10.7208/chicago/9780226282428.001.0001

[B97] Geoffroy Saint-Hilaire É (1791) Observations sur une petite espèce de maki (*Lemur* Linn.). Bulletin des Sciences, par la Société Philomatique de Paris 1: 89–90.

[B98] Geoffroy Saint-Hilaire É (1812) Tableau des quadrumanes, ou des animaux composant le premier ordre de la classe des mammifères. Annales du Muséum d’Histoire naturelle 19: 85–122.

[B99] Geoffroy Saint-Hilaire I (1850) Notes sur plusieurs espèces nouvelles de Mammifères, de l’ordre des Primates. Compte Rendu des séances de l’Académie des Sciences 31: 873–876.

[B100] Geoffroy Saint-Hilaire I (1851) Catalogue méthodique de la collection des mammifères de la collection des oiseaux et des collections annexes du Muséum D’Histoire aNturelle de Paris. Première partie. Mammifères. Catalogue des primates. In: Muséum d’Histoire naturelle de Paris. Catalogue méthodique de la collection des mammifères de la collection des oiseaux et des collections annexes. Gide et Baudry, Paris, vii + 96 pp.

[B101] Geoffroy Saint-Hilaire I De Blainville HMD (1834) Rapport sur les résultats scientifiques du voyage de M.. Alcide d’Orbigny dans l’Amerique du Sud, pendant les années 1826, 1827, 1828, 1829, 1830, 1831, 1832 et 1833. Nouvelles Annales du Muséum d’Histoire naturelle 3: 84–115.

[B102] Geoffroy Saint-Hilaire I Deville É (1848) Note sur huit espèces nouvelles de Singes américains, faisant partie des collections de MM. de Castelnau et Émile Deville. Compte Rendu des séances de l’Académie des Sciences 27: 497–499.

[B103] Gervais P (1841) Séance du 8 mai 1841. Mammalogie: Ecureuils. Societé philomatique de Paris. Extraits des procès-verbaux des séances 4: 51–52.

[B104] Gijzen A (1938) ‘s Rijks Museum van Natuurlijke Historie 1820–1915. W.L. & J. Brusse’s Uitgeversmaatschappij, Rotterdam, xii + 335 pp.

[B105] Gould J (1840a) Mr. Gould exhibited to the Meeting a new species of *Hypsiprimnus* [...]. Proceedings of the Zoological Society of London 6: 178–179.

[B106] Gould J (1840b) Mr. Gould, who had just returned from Australia [...]. The Athenaeum 670: 685.

[B107] Gould J (1841) Specimens were exhibited of five new species of Kangaroo [...]. Proceedings of the Zoological Society of London 8: 92–94.

[B108] Gould J (1844) Mr. Gould laid before the Meeting specimens of three new species of Mammalia [...]. Proceedings of the Zoological Society of London 12: 31–33.

[B109] Gould J (1845) Descriptions of five new species of mammals. Proceedings of the Zoological Society of London 1845: 77–79.

[B110] Gould J (1841–1842) Monograph of the Macropodidae, or family of kangaroos. Vol. 1. London, by the author, 15 pls and text, no pagination.

[B111] Grandidier A (1867) Mammifères et oiseaux nouveaux découverts à Madagascar et décrits par M. Alfred Grandidier. Revue et Magasin de Zoologie pure et appliquée et de Sériciculture comparée (2) 19: 84–88.

[B112] Gray JE (1830) Spicilegia Zoologica Or Original Figures and Short Systematic Descriptions of New and Unfigured Animals. Vol. 2. Treüttel, Würtz and co., London, 9–12.

[B113] Gray JE (1838) Notes on the above, with descriptions of two new species. In: R. Gunn. Notices accompanying a collection of quadrupeds and fish from Van Diemen’s Land.Annals of Natural History; or, Magazine of Zoology, Botany, and Geology 1: 106–108. 10.1080/00222933809512244

[B114] Gray JE (1843) List of the specimens of Mammalia in the collection of the British Museum. The Trustees [of the British Museum], London, 490 pp.

[B115] Gray JE (1863) Revision of the species of lemuroid animals, with the description of some new species. Proceedings of the scientific meetings of the Zoological Society of London for the year 1863: 129–152. [pls XVII–XIX]

[B116] Gray JE (1867) Synopsis of the Asiatic squirrels (Sciuridae) in the collection of the British Museum, describing one new genus and some new species. The Annals and Magazine of Natural History, including Zoology, Botany, and Geology ser. 3, 20: 270–286. 10.1080/00222936708694131

[B117] Gray JE (1871a) Catalogue of monkeys, lemurs and fruit-eating bats in the collection of the British Museum. The Trustees [of the British Museum], London, viii + 137 pp.

[B118] Gray JE (1871b) On a new species of lemur from Madagascar, and on the changes of *Lemurmacaco*, Linn. The Annals and Magazine of Natural History, including Zoology, Botany, and Geology (4)7: 339–340. 10.1080/00222937108696387

[B119] Gray JE (1873) Notes on *Propithecus*, *Indris*, and other Lemurs (*Lemurina*) in the British Museum. Proceedings of the scientific meetings of the Zoological Society of London for the year 1872: 846–860. [pls LXIX–LXXI]

[B120] Groves CP (2005a) Order Diprotodontia. In: Wilson DE Reeder DM (Eds) Mammal species of the world.A taxonomic and geographic reference. Third edition. Vol. 1. The Johns Hopkins University Press, Baltimore, 43–70.

[B121] Groves CP (2005b) Order Primates. In: Wilson DE Reeder DM (Eds) Mammal species of the world.A taxonomic and geographic reference. Third edition. Vol. 1. The Johns Hopkins University Press, Baltimore, 111–184.

[B122] Groves CP Flannery TF (1989) Revision of the genus *Dorcopsis* (Macropodidae: Marsupialia). In: Grigg G Jarman P Hume I (Eds) Kangaroos, wallabies and rat-kangaroos.Vol. 1. Surrey Beatty & Sons, Chipping Norton, 117–128.

[B123] Groves CP Tattersall I (1991) Geographical variation in the fork-marked lemur, *Phanerfurcifer* (Primates, Cheirogaleidae). Folia Primatologica 56: 39–49. 10.1159/000156526

[B124] Hamilton Smith C (1842) Introduction to Mammalia. In: Jardine W (Ed.) The Naturalist’s Library. Vol. XV. Mammalia. W.H. Lizars, Edinburgh, 313 pp. [30 pls]

[B125] Heaney LR (1985) Systematics of Oriental pygmy squirrels of the genera *Exilisciurus* and *Nannosciurus* (Mammalia: Sciuridae). Miscellaneous Publications of the Museum of Zoology, University of Michigan 170: 1–58.

[B126] Helgen KM Flannery TF (2004) Notes on the phalangerid marsupial genus *Spilocuscus*, with description of a new species from Papua. Journal of Mammalogy 85: 825–833. 10.1644/BER-110

[B127] Herman JS McGowan RY Swinney GN (1990) Catalogue of Type Specimens of Recent Vertebrates in the National Museums of Scotland. National Museums of Scotland Information Series (Vol. 4). Edinburgh, National Museums of Scotland, 34 pp.

[B128] Hershkovitz P (1959) Nomenclature and taxonomy of the Neotropical mammals described by Olfers, 1818. Journal of Mammalogy 40: 337–353. 10.2307/1376558

[B129] Hershkovitz P (1969) The evolution of mammals on southern continents. VI. The recent mammals of the Neotropical Region: a zoogeographic and ecological review. The Quarterly Review of Biology 44: 1–70. 10.1086/405975

[B130] Hershkovitz P (1976) Comments on generic names of four-eyed opossums (family Didelphidae). Proceedings of the Biological Society of Washington 89: 295–304.

[B131] Hershkovitz P (1977) Living New World monkeys (Platyrrhini). With an introduction to Primates. Vol. 1. The University of Chicago Press, Chicago/London, xiv + 1117 pp.

[B132] Hershkovitz P (1979) The species of sakis, genus Pithecia (Cebidae, Primates), with notes on sexual dichromatism. Folia Primatologica 31(1–2): 1–22. 10.1159/000155871 114463

[B133] Hershkovitz P (1984) Taxonomy of Squirrel Monkeys genus *Saimiri* (Cebidae, Platyrrhini): A Preliminary Report with Description of a Hitherto Unnamed Form. American Journal of Primatology 6: 155–210. 10.1002/ajp.1350070212 32160721

[B134] Hershkovitz P (1987) The taxonomy of South American sakis, genus *Pithecia* (Cebidae, Platyrrhini): a preliminary report and critical review with the description of a new species and a new subspecies. American Journal of Primatology 12(4): 387–468. 10.1002/ajp.1350120402 31973491

[B135] Hershkovitz P (1990) Titis, New World Monkeys of the Genus *Callicebus* (Cebidae, Platyrrhini): A Preliminary Taxonomic Review. Fieldiana Zoology, new series 55: 1–109.

[B136] Hershkovitz P (1997) Composition of the family Didelphidae Gray, 1821 (Didelphoidea: Marsupialia), with a review of the morphology and behavior of the included four-eyed pouched opossums of the genus *Philander* Tiedemann, 1808. Fieldiana Zoology, new series 86: i–iv + 1–103. 10.5962/bhl.title.3146

[B137] Hill WCO (1960) Primates. Comparative anatomy and taxonomy. IV. Cebidae, Part A. Edinburgh University Press, Edinburgh, vii + 523 pp.

[B138] Hill WCO (1970) The Primates. Comparative anatomy and taxonomy. VIII. Cynopithecinae. Edinburgh University Press, Edinburgh, 750 pp.

[B139] Hodgson BH (1836) Synoptical Description of Sundry New Animals, enumerated in the Catalogue of Nipálese Mammals. The Journal of the Asiatic Society of Bengal 5: 231–238.

[B140] Hodgson BH (1840) Three new Species of Monkey; with Remarks on the genera *Semnopithecus* et *Macacus*. The Journal of the Asiatic Society of Bengal 9: 1211–1213.

[B141] Hodgson BH (1841a) New species of *Rhizomys* discovered in Nepal. Calcutta Journal of Natural History: and Miscellany of the Arts and Sciences in India 2: 60–62.

[B142] Hodgson BH (1841b) Classified Catalogue of Mammals of Nepal, corrected to end of 1840, first printed in 1832. Calcutta Journal of Natural History: and Miscellany of the Arts and Sciences in India 2: 212–221.

[B143] Hodgson BH (1841c) Notice of the Marmot of the Himalaya and of Tibet. The Journal of the Asiatic Society of Bengal 10(2): 777–778.

[B144] Hodgson BH (1842) Classified catalogue of mammals of Nepal, corrected to end of 1840, first printed in 1832. Calcutta Journal of Natural History: and Miscellany of the Arts and Sciences in India 2: 212–221.

[B145] Hodgson BH (1849a) Brief Notice of several Mammalia and Birds discovered by B.H. Hodgson, Esq., in Upper India. The Annals and Magazine of Natural History, including Zoology, Botany, and Geology, ser. 2, 3: 202–203. 10.1080/03745485909494621

[B146] Hodgson BH (1849b) On the Physical Geography of the Himálayas. The Journal of the Asiatic Society of Bengal 18(2): 761–966.

[B147] Holthuis LB (1968) Biografische notities betreffende verzamelaars voor het Rijksmuseum van Natuurlijke Historie te Leiden. I. Hendrik Severinus Pel (1818–1876). Zoologische Bijdragen Leiden 10: 1–32.

[B148] Holthuis LB (1995) Rijksmuseum van Natuurlijke Historie 1820–1958. Nationaal Natuurhistorisch Museum, Leiden, 172 pp.

[B149] Holthuis LB Sakai T (1970) Ph.F. von Siebold and Fauna Japonica. A history of early Japanese zoology. Academic Press of Japan, Tokyo, 323 pp.

[B150] Hoogerwerf A (1939) Bergen, bosschen en blangs in de Gajo- en Alaslanden. Een tocht door de Gajo- en Alaslanden en iets over het Löserreservaat. Drie jaren Indisch natuurleven, elfde verslag (1936–1938): 242–288.

[B151] Hooijer DA (1948) A new race of the leaf monkey *Presbytisaygula* (L.) from Deli, Northeastern Sumatra. Proceedings of the Koninklijke Nederlandse Akademie van Wetenschappen 51: 234–237.

[B152] Hooijer DA (1962) Quaternary Langurs and Macaques from the Malay archipelago. Zoologische Verhandelingen 55(1): 1–64.

[B153] Hörner L (1839) De beklimming van den berg Ophir. Tijdschrift voor Neêrland’s Indië, 22: 605–622.

[B154] Horsfield T (1849) Brief Notice of several Mammalia and Birds discovered by B.H. Hodgson, Esq., in Upper India. The Annals and magazine of natural history; zoology, botany, and geology Ser. 2, 3: 202–203. 10.1080/03745485909494621

[B155] Horsfield T (1851) A Catalogue of the Mammalia in the Museum of the Hon. East-India Company. J.& H. Cox, London, 212 pp.

[B156] Horst MD De Raadt OLE (1918) De identiteit van *Musdiardii* Jentink. Zoologische Mededeelingen 4(2): 67–69.

[B157] Hose, C., 1893. A descriptive account of the mammals of Borneo. Edward Abbott, Norfolk, 78 pp. 10.5962/bhl.title.37842

[B158] Hummel HC (1994) Johan August Wahlberg Travel Journals (and some letters) South Africa and Namibia/Botswana, 1838–1856. Van Riebeeck Society, Cape Town, second series 23, 249 pp.

[B159] Husson AM (1955) Notes on the mammals collected by the Swedish New Guinea expedition 1948–1949. Nova Guinea, new series 6: 283–306, pls XXV–XXVIII.

[B160] Husson AM (1960) A new species of the rodent *Baiomys* from Aruba and Curaçao. Studies on the Fauna of Curaçao and other Caribbean islands 43: 33–40. [pls VI–VII]

[B161] Husson AM (1963) On *Blarinapyrrhonota* and *Echimysmacrourus*: Two Mammals Incorrectly Assigned to the Suriname Fauna. Studies on the Fauna of Suriname and other Guyanas 5: 34–41. [pls I, II]

[B162] Husson AM (1978) The mammals of Suriname. Zoölogische Monographieën van het Rijksmuseum van Natuurlijke Historie 2: i–xxxiv, 1–569. 10.1163/9789004626652

[B163] Husson AM Holthuis LB (1955) The dates of publication of “Verhandelingen over de natuurlijke geschiedenis der Nederlandsche overzeesche bezittingen” edited by C.J. Temminck. Zoologische Mededelingen Leiden 34(2): 17–24.

[B164] Husson AM Holthuis LB (1968) *Sciurusebii* Pel, 1851 (Mammalia): request for suppression under the plenary powers. Z.N.(S.) 1846. Bulletin of Zoological Nomenclature 25: 125–127. 10.5962/bhl.part.23971

[B165] Husson AM Rappard FW (1958) Note on the taxonomy and the habits of *Dendrolagusursinus* Temminck and *D.leucogenys* Matschie (Mammalia: Marsupialia). Nova Guinea. new series 9: 9–14. [pls I–IV]

[B166] Illiger K (1811) Ueberblick der Säugthiere nach ihrer Verteilung über die Welttheile. Abhandlungen der physikalischen Klasse der Königlich-Preussischen Akademie der Wissenschaften 1804–1811: 39–159.

[B167] International Commission on Zoological Nomenclature (1970) Opinion 945. *Sciurusebii* Pel, 1851 (Mammalia): suppressed under the plenary powers. Bulletin of Zoological Nomenclature 27: 224–225.

[B168] International Commission on Zoological Nomenclature (2001) Opinion 1978. *Myoxusjaponicus* Schinz, 1845 (currently *Glirulusjaponicus*; Mammalia, Rodentia): specific name conserved as the correct original spelling. Bulletin of Zoological Nomenclature 58(2): 159–160.

[B169] Jackson SM Jansen JJFJ Baglione G Callou C (2021a) Mammals collected and illustrated by the Baudin Expedition to Australia and Timor (1800–1804): A review of the current taxonomy of specimens in the Muséum national d`Histoire naturelle de Paris and the illustrations in the Muséum d`Histoire naturelle du Havre. Zoosystema 43(21): 387–548. 10.5252/zoosystema2021v43a21

[B170] Jackson SM Li Q Wan T Li X-Y Yu F-H Gao G He L-K Helgen KM Jiang X-L (2021b) Across the great divide: revision of the genus *Eupetaurus* (Sciuridae: Pteromyini), the woolly flying squirrels of the Himalayan region, with the description of two new species. Zoological Journal of the Linnean Society XX: 1–25. 10.1093/zoolinnean/zlab018

[B171] Jansen JJFJ (2017) An unpublished “Catalogue du Cabinet de C.J. Temminck” (c. 1803–1804). Archives of Natural History 44(2): 352–355. 10.3366/anh.2017.0454

[B172] Jenkins PD (1987) Catalogue of Primates in the British Museum (Natural History) and elsewhere in the British Isles. Part IV: Suborder Strepsirrhini, including the subfossil Madagascan lemurs and family Tarsiidae. British Museum (Natural History), London, x + 189 pp.

[B173] Jenkins PD Carleton MD (2005) Charles Immanuel Forsyth Major’s expedition to Madagascar, 1894 to 1896: beginnings of modern systematic study of the island’s mammalian fauna. Journal of Natural History, 39(20): 1779–1818. 10.1080/00222930400023719

[B174] Jenkins PD Knutson L (1983) A catalogue of the type specimens of Monotremata and Marsupialia in the British Museum (Natural History). London, British Museum (Natural History), 34 pp.

[B175] Jentink FA (1879a) On various species of *Mus*, collected by S.C.I.W. van Musschenbroek Esq. In Celebes. Notes from the Royal Zoological Museum of the Netherlands at Leyden (Notes from the Leyden Museum) 1: 7–13.

[B176] Jentink FA (1879b) On three new squirrels. Notes from the Royal Zoological Museum of the Netherlands at Leyden (Notes from the Leyden Museum) 1: 36–42.

[B177] Jentink FA (1879c) On a new porcupine from South-America. Notes from the Royal Zoological Museum of the Netherlands at Leyden (Notes from the Leyden Museum) 1: 93–96.

[B178] Jentink FA (1879d) On a new species of *Echimys*. Notes from the Royal Zoological Museum of the Netherlands at Leyden (Notes from the Leyden Museum) 1: 97–98.

[B179] Jentink FA (1879e) On a new genus and species of *Mus* from Madagascar. Notes from the Royal Zoological Museum of the Netherlands at Leyden (Notes from the Leyden Museum) 1: 107–109.

[B180] Jentink FA (1879f) On the hedgehogs from Madagascar. Notes from the Royal Zoological Museum of the Netherlands at Leyden (Notes from the Leyden Museum) 1: 137–151.

[B181] Jentink FA (1879g) On the Sumatra porcupine, *HystrixMülleri*, Temminck MS. Notes from the Royal Zoological Museum of the Netherlands at Leyden (Notes from the Leyden Museum) 1: 87–91.

[B182] Jentink FA (1880a) *Arvicolaratticeps*, eene voor de fauna van Nederland nieuwe soort. Tijdschrift van de Nederlandsche Dierkundige Vereeniging 5: 105–110. [pl. I]

[B183] Jentink FA (1880b) A Celebian mouse renamed. Notes from the Leyden Museum 2: 11–12.

[B184] Jentink F.A. (1880c) On some undescribed species of *Mus* in the Leyden Museum. Notes from the Leyden Museum 2: 13–36.

[B185] Jentink FA (1881) On a new squirrel, *Sciurussalae*. Notes from the Leyden Museum 3: 63–65.

[B186] Jentink FA (1882) Revision of the Manidae in the Leyden Museum. Notes from the Leyden Museum 4: 193–209.

[B187] Jentink FA (1883a) List of the specimens of squirrels in the Leyden Museum. Notes from the Leyden Museum 5: 91–144.

[B188] Jentink FA (1883b) A list of species of Mammals from West-Sumatra and North-Celebes, with descriptions of undescribed or rare species. Notes from the Leyden Museum 5: 170–235.

[B189] Jentink FA (1884) On the species of the Phalanger-genus *Pseudochirus*. Notes from the Leyden Museum 6: 108–110.

[B190] Jentink FA (1885) On *Didelphiscaudivolvula* Kerr and *Didelphisvulpecula* Kerr. Notes from the Leyden Museum 7: 21–28.

[B191] Jentink FA (1886a) On two new species of *Cercopithecus*. Notes from the Leyden Museum 8: 55–57.

[B192] Jentink FA (1886b) On a new species of *Hyrax* (*Hyraxstampflii*) from Liberia. Notes from the Leyden Museum 8: 209–212.

[B193] Jentink FA (1887) Muséum d’Histoire Naturelle des Pays-Bas. Tome IX. Catalogue ostéologique des mammifères. E.J. Brill, Leiden, 359 pp. [12 pls]

[B194] Jentink FA (1888) Muséum d’Histoire Naturelle des Pays-Bas. Tome XII. Catalogue systématique des mammifères (rongeurs, insectivores, cheiroptères, édentés et marsupiaux). E.J. Brill, Leiden, 280 pp.

[B195] Jentink FA (1888a) Zoological researches in Liberia. A list of mammals, collected by J. Büttikofer, C.F. Sala and F.X. Stampfli, with biological observations. Notes from the Leyden Museum 10: 1–58. [pls 1–4]

[B196] Jentink FA (1889) On a collection of Mammals from East-Sumatra. Notes from the Leyden Museum 11: 17–30.

[B197] Jentink FA (1890a) Mammalia from the Malay archipelago. II. Rodentia, Insectivora, Chiroptera. In: Weber M (Ed.) Zoologische Ergebnisse einer Reise in Niederländisch Ost-Indien.Erster Band. E.J. Brill, Leiden, 115–130. [pls VIII–XI] 10.1163/9789004596986_008

[B198] Jentink FA (1890b) On two very rare, nearly forgotten and often misunderstood mammals from the Malayan Archipelago. Notes from the Leyden Museum 12: 222–230. [pl. 9]

[B199] Jentink FA (1890c) Observations relating *Eupetauruscinereus*, Oldfield Thomas. Notes from the Leyden Museum 12: 143–144. [pl. 7, figs 1, 2]

[B200] Jentink FA (1890d) *Sciuropterusplatyurus*. On a new flying squirrel from Deli, Sumatra. Notes from the Leyden Museum 12: 145–148. [pl. 7, figs 3–10]

[B201] Jentink FA (1890e) On a collection of mammals from Billiton. Notes from the Leyden Museum 12: 149–154. [pl. 7, figs 11–14]

[B202] Jentink FA (1892) Muséum d’Histoire Naturelle des Pays-Bas. Tome XI. Catalogue systématique des mammifères (singes, carnivores, ruminants, pachydermes, sirènes et cétacés). E.J. Brill, Leiden, 219 pp.

[B203] Jentink FA (1892a) Mammalia from the Malay archipelago. II. Rodentia, Insectivora, Chiroptera. In: Weber M (Ed.) Zoologische Ergebnisse einer Reise in Niederländisch Ost-Indien.Dritter Band. E.J. Brill, Leiden, 77–82. [pl. V]

[B204] Jentink FA (1895) On *Potamogalevelox* du Chaillu. Notes from the Leyden Museum 12: 234–236. [pl. 9]

[B205] Jentink FA (1897) Zoological results of the Dutch Scientific Expedition to Central Borneo. The Mammals. Notes from the Leyden Museum 19: 26–127. [pl. 2, 3]

[B206] Jentink FA (1906) A new squirrel from the Stanley-falls. Notes from the Leyden Museum 28: 139–140.

[B207] Jentink FA (1907a) Mammals collected by the members of the Humboldt Bay- and the Merauke River-expeditions. Nova Guinea 5: 361–374. [pl. XVI]

[B208] Jentink FA (1907b) On *Arvicolaarenicola* de Sélys. Notes from the Leyden Museum 29: 263–266.

[B209] Jentink FA (1909a) Mammals collected by the Dutch New Guinea expedition 1907. Nova Guinea 9(2): 1–14. [pl. I]

[B210] Jentink FA (1909b) Beiträge zur Kenntnis der Fauna von Süd-Afrika, Ergebnisse einer Reise von Prof. Max Weber im Jahre 1894. Zoologische Jahrbücher 28: 239–262.

[B211] Jentink FA (1910) On a new mouse from Java. Notes from the Leyden Museum 33: 69.

[B212] Jentink FA (1911a) New and interesting mammals of the Dutch New-Guinea-expedition to the Snow-Mountains. Notes from the Leyden Museum 16: 234–236.

[B213] Jentink FA (1911b) Mammals, collected by the Dutch New Guinea expedition 1909/10. Nova Guinea 9: 165–183. [pl. VII]

[B214] Joost W (1987) Reise nach Brasilien in den Jahren 1815 bis 1817 von Maximilian Prinz zu Wied-Neuwied. Bearbeitet und herausgegeben von Wolfgang Joost. Edition Leipzig, Leipzig, 240 pp.

[B215] Jourdan C (1834) M. Jourdan, directeur du Musée d’histoire naturelle de Lyon, lit un Mémoire sur un nouveau genre de quadrumanes appartenant à la famille des Lémuriens, le genre *Avahi.* L’Institut 2: 231–232.

[B216] Julien-Laferrière D (1994) Catalogue des types de marsupiaux (Marsupialia) du Museum National d’Histoire Naturelle, Paris. Mammalia 58(1): 1–39. 10.1515/mamm.1994.58.1.1

[B217] Kaneko Y Smeenk C (1996) The author and date of publication of the Sikkim vole *Microtussikimensis*. Mammal Study 21(2): 161–164. 10.3106/mammalstudy.21.161

[B218] Kappeler PM Rasoloarison RM Razafimanantsoa L Walter L Roos C (2005) Morphology, behaviour and molecular evolution of giant mouse lemurs (*Mirza* spp.) Gray, 1870, with description of a new species. Primate Report 71: 3–26.

[B219] Kelaart EF (1852) Prodromus faunae zeylanicae: being contributions to the zoology of Ceylon. The Author, Ceylon, 58 pp. 10.5962/bhl.title.123402

[B220] Kellogg R Goldman EA (1944) Review of the spider monkeys. Proceedings of the United States National Museum 96(3186): 1–45. 10.5479/si.00963801.96-3186.1

[B221] Kerr R (1792) The Animal Kingdom, or zoological system, of the celebrated Sir Charles Linnaeus; Class I. Mammalia: containing a complete systematic description, arrangement, and nomenclature, of all the known species and varieties of the Mammalia, or animals which give suck to their young; being a translation of that part of the Systema Naturae, as lately published, with great improvements, by Professor Gmelin of Goettingen. Together with numerous additions from more recent zoological writers, and illustrated with copperplates. Systematic catalogue of the Mammalia. J. Murray & R. Faulder, London, xii + 400 pp.

[B222] Kloss CB (1921a) Seven new Malaysian mammals. Journal of the Federated Malay States Museums 10: 229–234.

[B223] Kloss CB (1921b) Some rats and mice of the Malay Archipelago. Treubia, 2: 115–124.

[B224] Koprowski JL Goldstein EA Bennett KR Pereira Mendes C (2020) Family Sciuridae. In: Burgin CJ Wilson DE Mittermeier RA Rylands AB Lacher TE Sechrest W (Eds) Illustrated Checklist of the Mammals of the World.Lynx Editions, Barcelona, 582–627.

[B225] Kraft R (1983) Die von J. B. Spix beschreibenen neotropischen Primaten und Chiroptere Verzeichnis der in der Zoologischen Staatssammlung München aufbewahrten Typus exemplare. Spixiana Supplement 9: 429–441.

[B226] Kuhl H (1820a) Tabula synoptica Simiarum Parisiis anno 1820 elaborata. In: Beiträge zur Zoologie und vergleichenden Anatomie. Erste Abtheilung. Beiträge zur Zoologie. Verlag der Hermannschen Buchhandlung, Frankfurt am Main, 1–52.

[B227] Kuhl H (1820b) Beschreibung einiger zum Theil neuer Marsupialien, Gliren und Falculaten des Illiger. In: Beiträge zur Zoologie und vergleichenden Anatomie. Erste Abtheilung. Beiträge zur Zoologie. Verlag der Hermannschen Buchhandlung, Frankfurt am Main, 61–74.

[B228] Kuroda N (1938) A list of the Japanese mammals. Author’s edition, Tokyo, 122 pp. [in Japanese]

[B229] Largen MJ (1985) Taxonomically and Historically Significant Specimens of Mammals in the Merseyside County Museums, Liverpool. Journal of Mammalogy 66(2): 412–418. 10.2307/1381265

[B230] Laurie EMO Hill JE (1954) List of land mammals of New Guinea, Celebes, and adjacent islands, 1758–1952. London, Trustees of the British Museum, 175 pp. [3 pls] 10.5962/bhl.title.112425

[B231] Linnaeus C (1758) Systema naturae per regna tria naturae, secundum classes, ordines, genera, species, cum characteribus, differentiis, synonymis, locis. 10.5962/bhl.title.542

[B232] Lyon MW (1906) The pigmy squirrels of the Nannosciurus group. Proceedings of the Biological Society of Washington 19: 51–56.

[B233] Lyon MW (1911) Mammals collected by Dr. W. L. Abbott on Borneo and some of the small adjacent islands. Proceedings of the United States National Museum 40: 53–146. [7 pls] 10.5479/si.00963801.40-1809.53

[B234] Lyon MW (1913) Treeshrews: an account of the mammalian family Tupaiidae. Proceedings of the United States National Museum 45: 1–188. [pls 1–11] 10.5479/si.00963801.45-1976.1

[B235] Macklot HC (1829) Uittreksels uit brieven van Dr. H.C. Macklot, natuuronderzoeker in Oost-Indië, aan Dr. C.J. Temminck, directeur, en J.A. Susanna, administrateur van ’s Rijks Museum te Leiden. Bijdragen tot de Natuurkundige Wetenschappen 4(1): 298–309.

[B236] Macklot HC (1830) Verslag van het land, de bewoners en voortbrengselen van eenige plaatsen op de kust van Nieuw Guinea, welke in den loop van het jaar 1828, door de Natuurkundige kommissie in Oost-Indië, aan boord van Z. M. korvet Triton, zijn bezocht, alsmede van de voorwerpen van natuurlijke historie, welke gedurende de reis, van den 20 Mei tot den 30 Augustus, op en langs die kust, door gemelde kommissie zijn verzameld. Bijdragen tot de Natuurkundige Wetenschappen 5(1): 142–182. 10.5962/bhl.title.119992

[B237] Major CIF (1896) Diagnoses of new mammals from Madagascar. The Annals and Magazine of Natural History, including Zoology, Botany, and Geology (6)18: 318–325. 10.1080/00222939608680461

[B238] Marsh LK (2014) A Taxonomic Revision of the Saki Monkeys, *Pithecia* Desmarest, 1804. Neotropical Primates 21(1): 1–165. 10.1896/044.021.0101

[B239] Marshall W (1871) Footnote In: Sclater 1871. Notes on rare or little-known animals now or lately in the Society’s Gardens.Proceedings of the scientific Meetings of the Zoological Society of London for the year 1871: 235–236.

[B240] Martin WCL (1841) A general introduction to the natural history of mammiferous animals, with a particular view of the physical history of man, and the more closely allied genera of the order Quadrumana, or monkeys. Wright and co., London, 545 pp. 10.5962/bhl.title.32451

[B241] Matschie P (1901) Die Säugetiere der von W. Kükenthal auf Halmahera, Batjan und Nord-Celebes gemachten Ausbeute. Abhandlungen herausgegeben von der Senckenbergischen naturforschenden Gesellschaft 25: 243–296. [pls XI–XIII]

[B242] McAllen AW Bruce MD (1989) Some problems in vertebrate nomenclature. I. Mammal. Bollettino, Museo Regionale di Scienze Naturali, Torino 7: 443–460.

[B243] McKay GM (1988) Petauridae. In: Bannister JL Calaby JH Dawson LJ Ling JK Mahoney JA McKay GM Richardson BJ Ride WDL Walton DW (Eds) Zoological catalogue of Australia.Vol. 5. Mammalia. Bureau of Flora and Fauna / Australian Government Publishing Service, Canberra, 87–97. 10.1163/9789004611450_017

[B244] Medway L (1963) Mammals of Borneo: Field Keys and an Annotated checklist. Journal of the Malayan Branch of the Royal Asiatic Society 36(3, 203): i–xiv + 1–193.

[B245] Medway L (1977) Mammals of Borneo: Field Keys and an Annotated checklist. (2^nd^ edn.). Monographs of the Malayan Branch of the Royal Asiatic Society 7: i–xii, 1–172.

[B246] Mees GF (1957) Over het belang van Temminck’s “Discours préliminaire” voor de zoologische nomenclatuur. Zoologische Mededelingen Leiden 35: 205–227.

[B247] Mees GF (1986) A list of the birds recorded from Bangka Island, Indonesia. Zoologische Verhandelingen Leiden 232: 1–176.

[B248] Meijaard E Fredriksson GM Kamminga P Frantz L (2018) Rediscovery of a squirrel species overlooked in 177 years. Preprint.

[B249] Melville RV China WE (1970) Opinion 920. *Inuusfuscatus* Blyth, 1875 (Mammalia): validated under the plenary powers. The Bulletin of Zoological Nomenclature 27: 77–78.

[B250] Menzies JI (1996) A Systematic Revision of Melomys (Rodentia: Muridae) of New Guinea. Australian Journal of Zoology, 44: 367–426. 10.1071/ZO9960367

[B251] Mertens R (1925) Verzeichnis der Säugetier-Typen des Senckenbergischen Museums. Senckenbergiana 7: 18–37.

[B252] Mertens R (1949) Eduard Rüppell. Leben und Werk eines Forschungsreisenden. Verlag Waldemar Kramer, Frankfurt am Main 388 pp. [44 pls]

[B253] Meyer AB (1890) Description of a new Squirrel from the Philippine Islands. Proceedings of the scientific meetings of the Zoological Society of London for the year 1890: 599–601.

[B254] Meyer AB (1890) Description of a new Squirrel from the Philippine Islands. Proceedings of the scientific meetings of the Zoological Society of London for the year 1890: 599–601.

[B255] Meyer AB (1896) Säugethiere von Celebes- und Philippinen-Archipel I. Abhandlungen und Berichte des Königlichen Zoologischen und Anthropologisch-Ethnographischen Museums zu Dresden 6: i–viii + 1–36. [pls I–XV] 10.5962/bhl.title.50304

[B256] Meyer AB (1898) Ueber zwei Eichhörnchenarten von Celebes. Abhandlungen und Berichte des Königlichen Zoologischen und Anthropologsch- Ethnographischen Museums zu Dresden 7(4): 1–3.

[B257] Mikan JC (1823) *Jacchus*. Desmarest. (Hapale. Illiger). In: Delectus florae et faunae Brasiliensis jussu et auspiciis Francisci I. Austriae Imperatoris investigatae. Antonius Strauss, Vindobonae: 2 pp. [1 pl]

[B258] Miljutin A (2010) Notes on the external morphology, ecology, and origin of *Megalomysdesmarestii* (Sigmodontinae, Cricetidae, Rodentia), the extinct giant rat of Martinique Island, Lesser Antilles. Estonian Journal of Ecology 59(3): 216–229. 10.3176/eco.2010.3.04

[B259] Milne-Edwards A (1867) Description de quelques espèces nouvelles d’Écureuils de l’ancien continent, par M. Alph. Milne-Edwards. Revue et magasin de zoologie pure et appliquée 19(2): 193–197.

[B260] Milne-Edwards A (1870) Note sur quelques Mammifères du Thibet oriental. Compte Rendu des séances de l’Académie des Sciences 70: 341–342.

[B261] Milne-Edwards A (1872) In: David A. Rapport adressé a mm. les professeurs-administrateurs du Muséum d’Histoire Naturelle. Nouvelles archives du Muséum d’histoire naturelle 7: 92.

[B262] Mohr E (1964) Zur Nomenklatur und Systematik der Quastenstachler, Gattung *Atherurus* F. Cuvier, 1829. Zeitschrift für Säugetierkunde 29(2): 93–116.

[B263] Moraes PLR (2009) The Brazilian herbarium of Maximilian Prince of Wied. Neodiversity 4: 16–51. 10.13102/neod.42.1

[B264] Müller S (1838) Over eenige nieuwe zoogdieren van Borneo. Tijdschrift voor Natuurlijke Geschiedenis en Physiologie 5: 134–150.

[B265] Müller S (1839) Over de zoogdieren van den Indischen Archipel. Verhandelingen over de natuurlijke geschiedenis der Nederlandsche overzeesche bezittingen, door de leden der Natuurkundige commissie in Indië en andere schrijvers. Zoologie, 1–8. [table]

[B266] Müller S (1840a) Over de zoogdieren van den Indischen Archipel. Verhandelingen over de natuurlijke geschiedenis der Nederlandsche overzeesche bezittingen, door de leden der Natuurkundige commissie in Indië en andere schrijvers. Zoologie: 9–57.

[B267] Müller S (1840b) Bijdragen tot de kennis van Nieuw-Guinea. Verhandelingen over de natuurlijke geschiedenis der Nederlandsche overzeesche bezittingen, door de leden der Natuurkundige commissie in Indië en andere schrijvers. Land- en volkenkunde, 1–30. [pls, map]

[B268] Müller S (1841) Over de zoogdieren van den Indischen Archipel. Verhandelingen over de natuurlijke geschiedenis der Nederlandsche overzeesche bezittingen, door de leden der Natuurkundige commissie in Indië en andere schrijvers. Zoologie.

[B269] Müller S Schlegel H (1840) Monographisch overzigt van het geslacht *Semnopithecus*. Verhandelingen over de natuurlijke geschiedenis der Nederlandsche overzeesche bezittingen, door de leden der Natuurkundige commissie in Indië en andere schrijvers. Zoologie, pls 8–10.

[B270] Müller S Schlegel H (1841a) Monographisch overzigt van het geslacht *Semnopithecus*. Verhandelingen over de natuurlijke geschiedenis der Nederlandsche overzeesche bezittingen, door de leden der Natuurkundige commissie in Indië en andere schrijvers. Zoologie, 57–84.

[B271] Müller S Schlegel H (1841b) Over de tot heden bekende eekhorens (*Sciurus*) van den Indischen Archipel. In: Temminck CJ (Ed.), Verhandelingen over de natuurlijke geschiedenis der Nederlandsche overzeesche bezittingen, door de leden der Natuurkundige Commissie in Indië en andere schrijvers. Zoologie. S. en J. Luchtmans & C.C. van der Hoek, Leiden.

[B272] Müller S Schlegel H (1843) Beschrijving eener nieuwe soort van vleeschetende buideldieren, *Phascogaleamelas*. Verhandelingen over de natuurlijke geschiedenis der Nederlandsche overzeesche bezittingen, door de leden der Natuurkundige commissie in Indië en andere schrijvers. Zoologie, pl. 25.

[B273] Müller S Schlegel H (1844) Over de tot heden bekende eekhorens (*Sciurus*) van den Indischen Archipel. In: Temminck CJ (Ed.) , Verhandelingen over de natuurlijke geschiedenis der Nederlandsche overzeesche bezittingen, door de leden der Natuurkundige Commissie in Indië en andere schrijvers.Zoologie. S. en J. Luchtmans & C.C. van der Hoek, Leiden, 85–102.

[B274] Müller S Schlegel H (1845) Beschrijving eener nieuwe soort van vleeschetende buideldieren, *Phascogaleamelas*. Verhandelingen over de natuurlijke geschiedenis der Nederlandsche overzeesche bezittingen, door de leden der Natuurkundige commissie in Indië en andere schrijvers. Zoologie, 149–152.

[B275] Musser GG (1969) Results of the Archbold Expeditions. No. 89 Notes on the Taxonomic Status of *Rattusaspinatus* Tate and Archbold and *Muscallitrichus* Jentink (Rodentia, Muridae). American Museum novitates 2365: 1–9.

[B276] Musser GG (1971a) The identities and allocations of *TaeromysParaxanthus* and *T. Tatei*, two taxa based on Composite Holotypes (Rodentia, Muridae). Zoologische Medelingen 45(11): 127–138.

[B277] Musser GG (1971b) The taxonomic status of *Rattustondanus* Sody and notes on the holotypes of *R.beccarii* (Jentink) and *R.thysanurus* Sody (Rodentia: Muridae). Zoologische Mededelingen Leiden 45: 147–157.

[B278] Musser GG (1971c) The taxonomic status of *Rattusdammermani* Thomas and *Rattustoxi* Sody (Rodentia, Muridae) of Celebes. Beaufortia 18(242): 205–216.

[B279] Musser GG (1981) Results of the Archbold Expediitions. No. 105. Notes on systematics of Indo-Malayan murid rodents, and descriptions of new genera and species from Ceylon, Sulawesi, and the Philippines. Bulletin of the American Museum of Natural History 168: 225–334

[B280] Musser GG (1987) The mammals of Sulawesi. In: Whitmore TC (Ed.) Biogeographical evolution of the Malay Archipelago.Clarendon Press, Oxford, 73–93.

[B281] Musser GG Carleton MD (2005) Superfamily Spalacidae. In: Wilson DE Reeder DM (Eds) Mammal species of the world.A taxonomic and geographic reference. Third edition. Vol. 1. The Johns Hopkins University Press, Baltimore, 894–1532.

[B282] Musser GG Piik E (1982) A new species of *Hydromys* (Muridae) from western New Guinea (Irian Jaya). Zoologische Mededelingen Leiden 56: 153–167. [pls 1–3]

[B283] Musser GG Durden LA Holden ME Light JE (2010) Systematic review of endemic Sulawesi squirrels (Rodentia, Sciuridae), with descriptions of new species of associated sucking lice (Insecta, Anoplura), and phylogenetic and zoogeographic assessments of sciurid lice. Bulletin of the American Museum of Natural History 339: 1–260. 10.1206/695.1

[B284] Napier PH (1985) Catalogue of the Primates in the British Museum (Natural History) and elsewhere in the British Isles. Part III: family Cercopithecidae, subfamily Colobinae. Trustees of the British Museum (Natural History), London, x + 111 pp.

[B285] Norris RW (2020) Family Spalacidae. In: Burgin CJ Wilson DE Mittermeier RA Rylands AB Lacher TE Sechrest W (Eds) Illustrated Checklist of the Mammals of the World.Lynx Editions, Barcelona, 326–329.

[B286] Oates JF (1985) The Nigerian guenon, *Cercopithecuserythrogaster*: ecological, behavioral, systematic and historical observations. Folia Primatologica 45: 25–43. 10.1159/000156189

[B287] Ogilby W (1838a) [On a new phalanger (*Phalangistaviverrina*), from Van Diemen’s Land]. Proceedings of the Zoological Society of London 5: [vi], 131.

[B288] Ogilby W (1838b) Observations upon some recent communications of Mr. J.E. Gray, of the British Museum, to the Annals of Natural History; with descriptions of two new kangaroos from Van Diemen’s Land. Annals of Natural History; or, Magazine of Zoology, Botany, and Geology 1: 216–221. 10.1080/00222933809512271

[B289] Ogilby W (1838c) The library of entertaining knowledge. The menageries. The natural history of monkeys, opossums, and lemurs. Vol. I. Charles Knight & Co., London, vi + 442 pp.

[B290] Ogilby W (1838d) Mr. Ogilby exhibited and characterized, under the name of *Macropusrufiventer* [...]. Proceedings of the Zoological Society of London 6: 23. 10.1111/j.1096-3642.1838.tb01413.x

[B291] Ogilby W (1838e) Mr. Ogilby exhibited and described various species of Kangaroo Rats (*Hypsiprymnus*) [...]. Proceedings of the Zoological Society of London 6: 62–63.

[B292] Oudemans AC (1890) Über zwei seltene und eine neue Art Affen des zoologischen Gartens im Haag, Holland. Der Zoologische Garten 31: 266–269.

[B293] Papavero N (1971) Essays on the history of Neotropical dipterology with special reference to collectors (1790–1905). Universidade de São Paulo, Museu de Zoologica, viii + 216 pp. 10.5962/bhl.title.101715

[B294] Parnaby HE Ingleby S Divljan A (2017) Type specimens of non-fossil mammals in the Australian Museum, Sydney. Records of the Australian Museum 69(5): 277–420. 10.3853/j.2201-4349.69.2017.1653

[B295] Pel HS (1851) Over de jagt aan de Goudkust volgens een tienjarige eigene ondervinding. Nederlandsch Tijdschrift voor Jagtkunde 1: 149–173.

[B296] Péron F (1816) Voyage de découvertes aux terres australes: exécuté par ordre de Sa Majesté l’empereur et roi, sur les corvettes le Géographe, le Naturaliste, et la goëlette le Casuarina, pendent les années 1800, 1801, 1802, 1803 et 1804. Historique: Tome Second. Paris, Imprimerie impériale, xxxj + 471 pp.

[B297] Peters W (1846) Hierauf las Hr. Müller zoologische Mittheilungen von Hrn. Peters aus Tette über neue Säugethiergattungen aus den Ordnungen der Insectenfresser und Nagethiere. Bericht über die zur Bekanntmachung geeigneten Verhandlungen der Königlichen Preussischen Akademie der Wissenschaften zu Berlin im Monat August 1846: 257–259.

[B298] Peters W (1847) Darauf legte Hr. Müller eine zoologische Mittheilung des Hrn. Peters aus Mozambique vom 19. Juni vorigen Jahres vor, betreffend eine neue Säugethiergattung aus der Ordnung der Insektenfresser. Bericht über die zur Bekanntmachung geeigneten Verhandlungen der Königlichen Preussischen Akademie der Wissenschaften zu Berlin im Monat Februar 1847: 36–38.

[B299] Peters WCH (1851) Derselbe machte eine Mittheilung über zwei neue Insectivoren aus Mossambique. Bericht über die zur Bekanntmachung geeigneten Verhandlungen der Königlichen Preussischen Akademie der Wissenschaften zu Berlin im Monat Juli 1851: 467–468.

[B300] Peters WCH (1852) Naturwissenschaftliche Reise nach Mossambique auf Befehl Seiner Majestät des Königs Friedrich Wilhelm IV in den Jahren 1842 bis 1848 ausgeführt. Zoologie. I. Säugethiere. Georg Reimer, Berlin, xvi + 202 pp. [44 pls] 10.5962/bhl.title.53810

[B301] Peters WCH (1872) Hr. W. Peters machte eine Mittheilung über eine neue Art von Indris, *Lichanotusmitratus*, aus Madagascar. Monatsberichte der Königlichen Akademie der Wissenschaften zu Berlin 1871: 360–362.

[B302] Pieters FFJM (1980) Notes on the menagerie and zoological cabinet of Stadholder William V of Holland, directed by Aernout Vosmaer. Journal of the Society for the Bibliography of Natural History 9(4): 539–563. 10.3366/jsbnh.1980.9.4.539

[B303] Pieters FFJM De Visser J (1993) The scientific career of the zoologist Max Wilhelm Carl Weber (1852–1937). Bijdragen tot de Dierkunde 62(4): 193–214. 10.1163/26660644-06204001

[B304] Pohle H (1927) Über die von Prof. Bresslau in Brasilien gesammelten Säugetiere (außer den Nagetieren). Abhandlungen herausgegeben von der Senckenbergischen Naturforschenden Gesellschaft 40: 237–247.

[B305] Pollen FPL (1863) Énumération des animaux vertébrés de l`île de Madagascar. Nederlandsch Tijdschrift voor de Dierkunde 1: 277–345.

[B306] Pollen FPL (1868) Recherches sur la faune de Madagascar et de ses dépendances, d’après les découvertes de François P.L. Pollen et D.C. van Dam. Relation de voyage. Vol. 1. J.K. Steenhoff, Leyde, 240 pp [9 pls, map] 10.5962/bhl.title.46191

[B307] Raymond L-M (1957) Auguste Plée (1787–1825) et la flore américaine, Les botanistes français en Amérique du Nord avant 1850. Colloques internationaux du Centre National de Recherche Scientifique 63: 193–201.

[B308] Reichenow A (1886) Zwei neue Säugethiere aus Inner-Africa. Zoologischer Anzeiger 9: 315–317.

[B309] Reinwardt CGC (1858) Reis naar het oostelijk gedeelte van den Indischen Archipel, in het jaar 1821; uit zijne nagelaten aanteekeningen opgesteld, met een levensberigt en bijlagen vermeerderd; door W.H. de Vriese. Werken van het Koninklijk Instituut voor Taal-, Land- en Volkenkunde van Nederlandsch Indië. Tweede afdeeling. Afzonderlijke werken. Frederik Muller, Amsterdam, xvi + 646 pp. [19 pls]

[B310] Reitsma E (2012) Duizend en meer verhalen op sterk water: 13 miljoen dieren Zoölogisch Museum Amsterdam. Stichting Uitgeverij Noord-Holland, Wormer, 320 pp.

[B311] Reuvens CL (1890) Einiges über die Myoxidae oder Schlafer. Notes from the Leyden Museum 13: 65–85. [tab. V]

[B312] Robinson HC Kloss CB (1916) Preliminary Diagnoses of some New Species and Subspecies of Mammals and Birds Obtained in Korinchi, West-Sumatra, Feb.–June 1914. Journal of the Straits Branch of the Royal Asiatic Society 73: 269–278.

[B313] Robinson HC Kloss CB (1919) On a collection of mammals from the Bencoolen and Palembang Residencies, South West Sumatra. Journal of the Federated Malay States Museums 7: 257–291. [pl. V]

[B314] Rode P (1938) Catalogue des types de mammifères du Muséum National d`Histoire Naturelle. Ordre des Primates, sous-ordre des simiens. Bulletin du Muséum National d`Histoire Naturelle (2)10: 202–251.

[B315] Rode P (1939) Catalogue des types de mammifères du Muséum National d`Histoire Naturelle. Ordre des Primates, sous-ordre des Lémuriens. Bulletin du Muséum National d`Histoire Naturelle (2)11: 434–449.

[B316] Rode P (1943) Catalogue des types de mammifères du Muséum National d`Histoire Naturelle. Ordre des Rongeurs. Bulletin du Muséum National d`Histoire Naturelle (2)15: 275–282.

[B317] Rode P (1945) Catalogue des types de mammifères du Muséum National d`Histoire Naturelle. Ordre des Rongeurs (suite). Bulletin du Muséum National d`Histoire Naturelle (2)17: 24–31.

[B318] Roos C Helgen KM Portela Miguez R Thant NML Lwin N Lin AK Lin A Yi KM Soe P Hein ZM Myint MNN Ahmed T Chetry D Urh M Veatch EG Duncan N Kamminga P Chua MAH Yao L Matauschek C Meyer D Liu Z-J Li M Nadler T Fan P-F Quyet LK Hofreiter M Zinner D Momberg F (2020) Mitogenomic phylogeny of the Asian colobine genus *Trachypithecus* with special focus on *Trachypithecusphayrei* (Blyth, 1847) and description of a new species. Zoological Research 41(6): 656–669. 10.24272/j.issn.2095-8137.2020.254 33171548 PMC7671912

[B319] Rümmler H (1935) Neue Muriden aus Neuguinea. Zeitschrift für Säugetierkunde 10: 105–118.

[B320] Rümmler H (1938) Die Systematik und Verbreitung der Muriden Neuguineas. Mitteilungen aus dem Zoologischen Museum in Berlin 23: 1–296.

[B321] Rüppell E (1835) Neue Wirbelthiere zu der Fauna von Abyssinien gehörig, entdeckt und beschrieben von Dr. Eduard Rüppell Säugethiere. Siegmund Schmerber, Frankfurt am Main, 40 pp. [14 pls] 10.5962/bhl.title.53778

[B322] Rylands AB Mittermeier RA (2020) Family Callitrichidae. In: Burgin CJ Wilson DE Mittermeier RA Rylands AB Lacher TE Sechrest W (Eds) Illustrated Checklist of the Mammals of the World.Lynx Editions, Barcelona, 174–183.

[B323] Rylands AB Mittermeier RA Cornejo FM Defler TR Glander KE Konstant WR Pinto LP Talebi M (2020) Family Atelidae. In: Burgin CJ Wilson DE Mittermeier RA Rylands AB Lacher TE Sechrest W (Eds) Illustrated Checklist of the Mammals of the World.Lynx Editions, Barcelona, 212–219.

[B324] Schinz HR (1845) Systematisches Verzeichniss aller bis jetzt bekannten Säugethiere oder Synopsis Mammalium nach dem Cuvier’schen System. Vol. 2. Jent & Gassmann, Solothurn, 51 pp.

[B325] Schlegel H (1857) Handleiding tot de beoefening der dierkunde. 1e deel. Koninklijke Akademie voor de Zee- en Landmacht / Boekdrukkerij van de Gebroeders Nys, Breda, lii + 530 pp. 10.5962/bhl.title.11648

[B326] Schlegel H (1863) Notices sur les écureuils à ventre rouge et à flances rayés de l’Archipel Indien. Nederlandsch Tijdschrift voor de Dierkunde 1: 24–30. [pls 1–2]

[B327] Schlegel H (1866a) Contributions à la faune de Madagascar et des îles avoisinantes, d`après les découvertes et observations de M.M. François Pollen et M. D.-C. van Dam. Nederlandsch Tijdschrift voor de Dierkunde 3: 73–89.

[B328] Schlegel H (1866b) Observations zoologiques. II. Nederlandsch Tijdschrift voor de Dierkunde 3: 249–258.

[B329] Schlegel H (1866c) Observations zoologiques. III. Nederlandsch Tijdschrift voor de Dierkunde 3: 325–358.

[B330] Schlegel H (1867) [Communication on mammals and birds collected in Madagascar]. Proceedings of the scientific meetings of the Zoological Society of London for the year 1866: 419–426.

[B331] Schlegel H (1876) Muséum d’Histoire Naturelle des Pays-Bas. Revue méthodique et critique des collections déposées dans cet établissement. Tome VII. Contenant: Monographie 40: Simiae: 1–356. E.J. Brill, Leide.

[B332] Schlegel H (1880) On *Dasyurusalbopunctatus*. Notes from the Leyden Museum 2: 51–53.

[B333] Schlegel H (1881) On the zoological researches in West Africa. I. Notes from the Leyden Museum 3: 53–58.

[B334] Schlegel H Müller S (1841) Over drie buideldieren uit de familie der kengoeroe’s. Verhandelingen over de natuurlijke geschiedenis der Nederlandsche overzeesche bezittingen, door de leden der Natuurkundige commissie in Indië en andere schrijvers. Zoologie, pls 19–24.

[B335] Schlegel H Müller S (1843) Over de, op de Oost-Indische eilanden levende soorten van het geslacht Hylogalea. Verhandelingen over de natuurlijke geschiedenis der Nederlandsche overzeesche bezittingen, door de leden der Natuurkundige commissie in Indië en andere schrijvers. Zoologie, pls 26–27.

[B336] Schlegel H Müller S (1845a) Bijdragen tot de natuurlijke geschiedenis der vliegende eekhorens (*Pteromys*). Verhandelingen over de natuurlijke geschiedenis der Nederlandsche overzeesche bezittingen, door de leden der Natuurkundige commissie in Indië en andere schrijvers. Zoologie, pl. 16.

[B337] Schlegel H Müller S (1845b) Over drie buideldieren uit de familie der kengoeroe’s. Verhandelingen over de natuurlijke geschiedenis der Nederlandsche overzeesche bezittingen, door de leden der Natuurkundige commissie in Indië en andere schrijvers. Zoologie, 129–148.

[B338] Schlegel H Müller S (1845c) Over de, op de Oost-Indische eilanden levende soorten van het geslacht *Hylogalea*. Verhandelingen over de natuurlijke geschiedenis der Nederlandsche overzeesche bezittingen, door de leden der Natuurkundige commissie in Indië en andere schrijvers. Zoologie, 159–168.

[B339] Schlegel H Pollen FPL (1868) Mammifères et oiseaux. In: Pollen FPL, Van Dam DC. Recherches sur la faune de Madagascar et de ses dépendances, d’après les découvertes de François P.L. Pollen et D.C. van Dam. 2^me^ partie. J.K. Steenhoff, Leyde, xix + 186 pp. [40 pls] 10.5962/bhl.title.46191

[B340] Schunke AC Hutterer R (2005) Geographic Variation in the West African Scaly-Tailed Squirrel *Anomaluruspelii* (Schlegel and Müller, 1845) and Description of a new Subspecies (Rodentia: Anomaluridae). In: Huber BA (Ed.) African Biodiversity: Molecules, Organisms, Ecosystems: Proceedings of the Fifth International Symposium on Tropical Biology, Museum Koenig, Bonn.Springer Verlag, Berlin, 321–328. 10.1007/0-387-24320-8_32

[B341] Schwaner CALM (1854) Borneo. Beschrijving van het stroomgebied van den Barito en reizen langs eenige voorname rivieren van het zuid-oostelijk gedeelte van dat eiland, door D^r^ C.A.L.M. Schwaner, op last van het Gouvernement van Nederl. Indie gedaan in de jaren 1843–1847. 2e deel. P.N. van Kampen, Amsterdam, xiv + 200 pp.

[B342] Schwarz E (1927) Erythrism in monkeys of the genus *Cercopithecus*. Studies of variation in mammals. The Annals and Magazine of Natural History. Including Zoology, Botany, and Geology (9)19: 151–155. 10.1080/00222932708633582

[B343] Schwarz E (1928) Bemerkungen über die roten Stummelaffen. Zeitschrift für Säugetierkunde 3: 92–97.

[B344] Schwarz E (1931) A revision of the genera and species of Madagascar Lemuridae. Proceedings of the general meetings for scientific business of the Zoological Society of London 1931: 399–428. 10.1111/j.1096-3642.1931.tb01020.x

[B345] Sclater PL (1870) [Mr. P.L. Sclater exhibited, and made remarks on, a specimen of a newly described Lemur…]. Proceedings of the scientific meetings of the Zoological Society of London for the year 1870: 112.

[B346] Sclater PL (1893) List of the Dates of Delivery of the Sheets of the ‘Proceedings’ of the Zoological Society of London, from the commencement in 1830 to 1859 inclusive. Proceedings of the scientific meetings of the Zoological Society of London for the year 1893: 435–440.

[B347] Seba A (1734) Locupletissimi rerum naturalium thesauri accurata descriptio et iconibus artificiosissimis expressio per universam physices historiam. Tomus I. H. Janssonius van Waesberge, & J. Wettstein & W. Smith, Amsterdam, 178 pp. [111 pls] 10.5962/bhl.title.62680

[B348] Sganzin V (1840) Notes sur les mammifères et sur l’ornithologie de l’île de Madagascar (1831 et 1832). Mémoires de la Société d’Histoire naturelle de Strasbourg 3: 1–49.

[B349] Shekelle M Gursky-Doyen S Richardson MC (2020) Family Tarsiidae. In: Burgin CJ Wilson DE Mittermeier RA Rylands AB Lacher TE Sechrest W (Eds) Illustrated Checklist of the Mammals of the World.Lynx Editions, Barcelona, 194–211.

[B350] Sherborn CD (1899) A Note on the Date of the Parts of ‘Humboldt and Bonpland’s Voyage: Observation’s de Zoologie’. The Annals and magazine of natural history; zoology, botany, and geology 3: 428. 10.1080/00222939908678146

[B351] Sherborn CD (1934) Dates of publication of catalogues of Natural History (post 1850) issued by the British Museum Annals and Magazine of Natural History 13(74): 308–312. 10.1080/00222933408654812

[B352] Sherborn CD Griffin FJ (1934) On the dates of publication of the natural history portions of Alcide d’Orbigny’s Voyage Amérique méridionale`. Annals and Magazine of Natural History 13(73): 130–134. 10.1080/00222933408654798

[B353] Shoshani J (2005) Order Hyracoidea. In: Wilson DE Reeder D (Eds) Mammal species of the world.A taxonomic and geographic reference. Third edition. Vol. 1. The Johns Hopkins University Press, Baltimore, 87–89.

[B354] Shultz S Roberts D (2013) *Dendrohyraxdorsalis* Western tree hyrax. In: Kingdon J Happold D Hoffmann M Butynski T Happold M Kalina J (Eds) Mammals of Africa.Vol. I. Bloomsbury Publishing, London, 155–157.

[B355] Smeenk C (2009) Has one of Captain Cook’s possums landed in Leiden? The possible holotype of *Pseudocheirusperegrinus* (Boddaert, 1785). Zoologische Mededelingen Leiden 83: 723–740.

[B356] Smeenk C Kaneko Y (2000) *Myoxusjaponicus* Schinz, 1845 (currently *Glirulusjaponicus*; Mammalia, Rodentia): proposed conservation as the correct original spelling of the specific name. Bulletin of Zoological Nomenclature 57(1): 36–38. 10.5962/bhl.part.20666

[B357] Smeenk C Kaneko Y Tsuchiya K (1982) On the type material of *Musargenteus* Temminck, 1844. Zoologische Mededelingen 56(9): 122–129. [pl. I–IV]

[B358] Smeenk C Godfrey LR Williams FL (2006) The early specimens of the potto*Perodicticuspotto* (Statius Müller, 1776) in the National Museum of Natural History, Leiden, with the selection of a neotype. Zoologische Mededelingen Leiden 80(4): 139–164.

[B359] Smuts J (1832) Dissertatio zoologica inauguralis, enumerationem mammalium Capensium. J.C. Cyfveer, Leiden, 108 pp. [pls I–III]

[B360] Sody HJV (1929a) Naamlijst van de zoogdieren van Java (met korte beschrijving van 2 nieuwe subspecies). Natuurkundig Tijdschrift voor Nederlandsch-Indië 89: 160–166.

[B361] Sody HJV (1929b) Over *Callosciurusnotatus*, Bodd., met beschrijving van 2 nieuwe subspecies. Natuurkundig Tijdschrift voor Nederlandsch-Indië, Batavia 89: 325–332.

[B362] Sody HJV (1930a) Over *Rattusrattusroquei* Sody met beschrijving van een nieuw ras van Rattusrattus van Soemba. Zoologische Mededeelingen 13: 94–99.

[B363] Sody HJV (1930b) Overzicht van de ratten van Java, met beschrijving van een nieuw subspecies. Zoologische Mededeelingen 13: 100–140.

[B364] Sody HJV (1930c) *PithecusAygulaFredericae* n. subsp. De tropische natuur, Weltevreden 19(4): 68.

[B365] Sody HJV (1931a) Two new races of *Rattuslepturus* from Java. Natuurkundig Tijdschrift voor Nederlandsch-Indië 91: 212–215.

[B366] Sody HJV (1931b) Six new mammals from Sumatra, Java, Bali and Borneo. Natuurkundig Tijdschrift voor Nederlandsch-Indië 91: 349–360.

[B367] Sody HJV (1932a) Four new mammals from Bali and Soemba. Natuurkundig Tijdschrift voor Nederlandsch-Indië 92: 334–340.

[B368] Sody HJV (1932b) Six new Indo-Malayan rats. Natuurhistorisch Maandblad, Orgaan van het Natuurhistorisch Genootschap in Limburg 21(12): 157–160.

[B369] Sody HJV (1933) Ten new mammals from the Dutch East Indies. The Annals and Magazine of Natural History, including Zoology, Botany, and Geology 10(12): 430–442. 10.1080/00222933308673706

[B370] Sody HJV (1934) New rats from Java and New Guinea. Natuurkundig Tijdschrift voor Nederlandsch-Indië 94: 170–177.

[B371] Sody HJV (1936a) Seventeen new generic, specific, and subspecific names for Dutch East Indian mammals. Natuurkundig Tijdschrift voor Nederlandsch-Indië 96: 42–55.

[B372] Sody HJV (1936b) A new flying squirrel from Sumatra. Natuurkundig Tijdschrift voor Nederlandsch-Indië 96: 146–147.

[B373] Sody HJV (1936c) A new race of *Callosciurusnotatus* from North Sumatra. Natuurkundig Tijdschrift voor Nederlandsch-Indië 96: 217–218.

[B374] Sody HJV (1937) Notes on some mammals from Sumatra, Java, Bali, Buru, and New Guinea. Temminckia 2: 211–220.

[B375] Sody HJV (1940) On the mammals of Enggano. Treubia 17: 391–401, map. 10.14203/treubia.v17i4.2582

[B376] Sody HJV (1941) On a collection of rats from the Indo-Malayan and Indo-Australian regions. Treubia 18(2): 255–325. 10.14203/treubia.v18i2.2605

[B377] Sody HJV (1949a) On a collection of Sciuridae from the Indo-Malayan and Indo-Australian regions with descriptions of 20 new species and subspecies, and with some remarks on the essential significance and the denomination of subspecies. Treubia 20: 57–120.

[B378] Sody HJV (1949b) Notes on some Primates, Carnivora, and the babirusa from the Indo-Malayan and Indo-Australian regions (with descriptions of 10 new species and subspecies). Treubia 20: 121–190. [table 1]

[B379] Statius Müller PL (1776) Des Ritters Carl von Linné Königlich Schwedischen Leibarztes etc. etc. vollständigen Natursystems Supplements- und Register-Band über alle sechs Theile oder Classen des Thierreichs. Mit einer ausführlichen Erklärung ausgefertiget von Philipp Ludwig Statius Müller. Erste Classe. Säugende Thiere. Gabriel Nicolaus Raspe, Nürnberg, 62 pp. [3 pls]

[B380] Stokes JL (1846) Discoveries in Australia; with an account of the coasts and rivers explored and surveyed during the voyage of H.M.S. Beagle in the years 1837-38-39-40-41-42-43. By command of the Lords Commissioners of the Admiralty. Also a narrative of captain Owen Stanley’s visits to the islands in the Arafura Sea. Vol. 2. London, T. and W. Boone, 543 pp. 10.5962/bhl.title.20334

[B381] Stuhlmann F (1894) Mit Emin Pascha ins Herz von Afrika: ein Reisebericht. Geographische Verlagsbuchhandlung von Dietrich Reimer, Berlin, 901 pp.

[B382] Sundevall CJ (1844) J. Wahlbergs samlingar från Sydafrika. Öfversigt af Kongl. Vetenskaps-Akademiens Föhandlingar 1: 159–161.

[B383] Sundevall CJ (1846) Nya Mammalia från Sydafrika. Öfversigt af Kongl. Vetenskaps-Akademiens Förhandlingar 3: 118–121.

[B384] Susanna JA (1858) Levensschets van Coenraad Jacob Temminck. Handelingen en Levensberichten van de Maatschappij der Nederlandsche Letterkunde 1858: 47–75.

[B385] Swinhoe R (1863) On the Mammals of the Island of Formosa (China). Proceedings of the scientific meetings of the Zoological Society of London for the year 1862: 347–365. [pls XLII–XLV]

[B386] Tate GHH (1933) A systematic revision of the marsupial genus *Marmosa*, with a discussion of the adaptive radiation of the murine opossums (*Marmosa*). Bulletin of the American Museum of Natural History 66: 1–250. [pls I–XXVI, table I: 1–9]

[B387] Tate GHH (1940) Notes on the types of certain early described species of monotremes, marsupials, Muridae and bats from the Indo-Australian region. American Museum Novitates 1061: 1–10.

[B388] Tate GHH (1945) Results of the Archbold Expeditions. No. 54. The marsupial genus *Pseudocheirus* and its subgenera. American Museum Novitates 1287: 1–30.

[B389] Tate GHH (1947) Results of the Archbold Expeditions. No. 56. On the anatomy and classification of the Dasyuridae (Marsupialia). Bulletin of the American Museum of Natural History 88: 97–156.

[B390] Tate GHH (1948) Studies in the Peramelidae (Marsupialia). Bulletin of the American Museum of Natural History 92(6): 313–346.

[B391] Tattersall I (2022) The Itineraries of Alfred Crossley, and Natural History Collecting in Mid-Nineteenth Century Madagascar. American Museum Novitates 3987: 1–28. 10.1206/3987.1

[B392] Taylor JM Horner BE (1967) Results of the Archbold Expeditions. No. 88 The Historical Misapplication of the Name Musfuscipes and a Systematic Re-Evaluation of Rattus lacus (Rodentia, Muridae). American Museum Novitates 2281: 1–14.

[B393] Temminck CJ (1807) Catalogue systématique du cabinet d’ornithologie et de la collection de quadrumanes. C. Sepp Jansz., Amsterdam, 304 pp.

[B394] Temminck CJ (1824a) Première monographie. Sur le genre phalanger. *Phalangista*. (Geoff.) (1). In: Monographies de mammalogie, ou description de quelques genres de mammifères, dont les espèces ont été observées dans les différens musées de l’Europe. Tome premier. G. Dufour & Ed. d’Ocagne, Paris/Amsterdam, 1–20. [pls I–IV]

[B395] Temminck CJ (1824b) Deuxième monographie sur le genre sarigue (1) – *Didelphis*. (Linn.). In: Monographies de mammalogie, ou description de quelques genres de mammifères, dont les espèces ont été observées dans les différens musées de l’Europe. Tome premier. G. Dufour & Ed. d’Ocagne, Paris/Amsterdam, 21–54. [pls V–VI]

[B396] Temminck CJ (1824c) Troisième monographie. Sur les mammifères du genre dasyure, et sur deux genres voisins, les thylacynes et les phascogales. In: Monographies de mammalogie, ou description de quelques genres de mammifères, dont les espèces ont été observées dans les différens musées de l’Europe. Tome premier. G. Dufour & Ed. d’Ocagne, Paris/Amsterdam, 55–72. [pls VII–VIII]

[B397] Temminck CJ (1827a) Tableau méthodique des mammifères In: Monographies de mammalogie ou description de quelques genres de mammifères, dont les espèces ont été observées dans les diférens musées de l’Europe. Tome premier. G. Dufour & Ed. d’Ocagne, Paris, Amsterdam, xiii–xxxii.

[B398] Temminck CJ (1827b) Septième monographie. Sur une espèce nouvelle de la famille des rongeurs, formant le type du genre ulacode. – Aulacodus (V. Swind.). In: Monographies de mammalogie, ou description de quelques genres de mammifères, dont les espèces ont été observées dans les différens musées de l’Europe. Tome premier. G. Dufour & Ed. d’Ocagne, Paris, Amsterdam, 245–248. [pl. XXV]

[B399] Temminck CJ (1828) Blik op de dierlijke bewoners van de Sunda-eilanden en van de overige Nederlandsche bezittingen in Indië. Bijdragen tot de Natuurkundige Wetenschappen 3: 62–78.

[B400] Temminck CJ (1832) Monographie over een nieuw geslacht van knaagdier, onder den naam van Nyctocleptes. Bijdragen tot de Natuurkundige Wetenschappen 7: 1–8. [pl. 1]

[B401] Temminck CJ (1835) Neuvième monographie. Sur un nouveau genre de rongeur sous le nom de Nyctocleptes (1). In: Temminck, C.J. (Ed.) Monographies de mammalogie, ou description de quelques genres de mammifères, dont les espèces ont été observées dans les différens musées de l’Europe. Tome second, C.C. van der Hoek, Leiden & Ed. d’Ocagne & A. Bertrand, Paris, 40–45. [pl. XXXIII]

[B402] Temminck CJ (1836) Coup-d’oeil sur la faune des îles de la Sonde et de l’Empire du Japon. Discours préliminaire destiné à servir d’introduction à la Faune du Japon. Leiden, 30 pp. 10.5962/bhl.title.119899

[B403] Temminck CJ (1842) Aperçu général et spécifique sur les mammifères qui habitent le Japon et les iles qui en dépendent. In: Siebold PF (Ed.) Fauna Japonica sive descriptio animalium, quae in itinere per Japoniam, jussu et auspiciis superiorum, qui summum in India Batava imperium tenent, suscepto, annis 1823–1830 collegit, notis, observationibus et adumbrationibus illustravit Ph.Fr. de Siebold. Conjunctis studiis C.J. Temminck et H. Schlegel pro vertebratis atque W. de Haan pro invertebratis elaborata, Arnz et Socii, Lugduni Batavorum, 1–24. [pls 1–10]

[B404] Temminck CJ (1844) Aperçu général et spécifique sur les mammifères qui habitent le Japon et les îles qui en dépendent. In: Siebold, P.F. (Ed.) Fauna Japonica sive descriptio animalium, quae in itinere per Japoniam, jussu et auspiciis superiorum, qui summum in India Batava imperium tenent, suscepto, annis 1823–1830 collegit, notis, observationibus et adumbrationibus illustravit Ph. Fr. de Siebold. Conjunctis studiis C.J. Temminck et H. Schlegel pro vertebratis atque W. de Haan pro invertebratis elaborata, Arnz et Socii, Lugduni Batavorum, 25–59. [pls 11–20]

[B405] Temminck CJ (1846) Coup-d’oeil général sur les possessions néerlandaises dans l’Inde archipélagique. Tome premier. A. Arnz & Comp., Leiden, xxii + 352 pp.

[B406] Temminck CJ (1847) Coup-d’oeil général sur les possessions néerlandaises dans l’Inde archipélagique. Tome second. A. Arnz & Comp., Leiden, viii + 471 pp.

[B407] Temminck CJ (1849) Coup-d’oeil général sur les possessions néerlandaises dans l’Inde archipélagique. Tome troisième. Arnz & Comp., Leiden, viii + 379 pp.

[B408] Temminck CJ (1853) Esquisses zoologiques sur la côte de Guiné. 1e partie, les mammifères. E.J. Brill, Leiden, xvi + 256 pp. 10.5962/bhl.title.14828

[B409] Thalmann U Geissmann T (2000) Distribution and geographic variation in the western woolly lemur (*Avahioccidentalis*) with description of a new species (*A.unicolor*). International Journal of Primatology 21: 915–941. 10.1023/A:1005507028567

[B410] Thomas O (1880) On the *Myoxuselegans* of Temminck. Proceedings of the scientific Meetings of the Zoological Society of London 1880: 40–41.

[B411] Thomas O (1882) On a small Collection of Rodents from South-Western Africa. Proceedings of the Zoological Society of London for the year 1882: 265–267. 10.1111/j.1096-3642.1882.tb02741.x

[B412] Thomas O (1884) On a Collection of Muridae from Central Peru. Proceedings of the scientific Meetings of the Zoological Society of London 1884: 447–458. [pls XLII–XLIV] 10.1111/j.1096-3642.1884.tb02857.x

[B413] Thomas O (1888a) On *Eupetaurus*, a new form of flying squirrel from Kashmir. The journal of the Asiatic Society of Bengal 57(2): 256–260.

[B414] Thomas O (1888b) Catalogue of the Marsupialia and Monotremata in the Collection of the British Museum (Natural History). The Trustees [of the British Museum], London, 401 pp. [28 pls]

[B415] Thomas O (1892) On the probable Identity of certain Specimens, formerly in the Lidth de Jeude Collection, and now in the British Múseum Proceedings of the Zoological Society of London 60(3): 309–348. 10.1111/j.1469-7998.1892.tb06833.x

[B416] Thomas O (1894) Description of some new Neotropical Muridae. The Annals and Magazine of Natural History, including Zoology, Botany, and Geology (6)14: 346–366. 10.1080/00222939408677813

[B417] Thomas O (1905) The Duke of Bedford’s Zoological Exploration in Eastern Asia. I. List of Mammals obtained by Mr. M. P. Anderson in Japan. Proceedings of the Zoological Society of London, November 14 1905: 331–363. 10.1111/j.1469-7998.1906.tb08400.x

[B418] Thomas O (1915) Notes on the Asiatic bamboo-rats (*Rhizomys*, etc.). Annals and Magazine of Natural History 16(91): 56–61. 10.1080/00222931508693685

[B419] Thomas O (1916) On the Rats usually included in the genus *Arvicanthis*. The Annals and magazine of natural history; zoology, botany, and geology (8)18: 67–70. 10.1080/00222931608693822

[B420] Thomas O (1918) A selection of lectotypes of Indian mammals, from the co-types described by Hodgson, Gray, Elliot and others. Journal of the Bombay Natural History Society 25(3): 368–372.

[B421] Thomas O (1921) Notes on Australasian rats, with a selection of lectotypes for Australasian Muridae. The Annals and magazine of natural history; zoology, botany, and geology (9)8: 425–433. 10.1080/00222932108632602

[B422] Thomas O (1922) A selection of lectotypes of the typical Australian marsupials in the British Museum collection. Annals and Magazine of Natural History (9) 10: 127–128. 10.1080/00222932208632749

[B423] Thomas O (1923a) On some Queensland Phalangeridae. The Annals and Magazine of Natural History. Including Zoology, Botany, and Geology (9)11: 246–250. 10.1080/00222932308632848

[B424] Thomas O (1923b) On some mammals, chiefly bats, from the East Indian Archiopelago. The Annals and Magazine of Natural History. Including Zoology, Botany, and Geology (9)11: 250–255. 10.1080/00222932308632849

[B425] Thomas O (1927) A selection of lectotypes of American rodents in the collection of the British Museum. The Annals and Magazine of Natural History. Including Zoology, Botany, and Geology (9)19: 545–554. 10.1080/00222932708655531

[B426] Thomas O Hartert E (1894) First glimpses of the zoology of the Natuna Islands. III. List of the first collection of mammals from the Natuna Islands. Novitates Zoologicae 1: 652–659. 10.5962/bhl.part.24567

[B427] Thorington RW Hoffmann RS (2005) In: Wilson DE Reeder DM (Eds) 2005.Mammal species of the world. A taxonomic and geographic reference. Third edition. 2 Vols. The Johns Hopkins University Press, Baltimore, 754–818.

[B428] Turni H (2024) Type specimens of Primates (Mammalia) in the collections of the Museum für Naturkunde Berlin. Zootaxa 5543(2): 151–194 . 10.11646/zootaxa.5543.2.1 39646114

[B429] Turni H Hutterer R Asher R (2007) Type specimens of “insectivoran” mammals at the Museum für Naturkunde, Berlin. Zootaxa 1470(1): 1–33. 10.11646/zootaxa.1470.1.1

[B430] van der Boon Mesch AH (1829) Beschrijving en afbeelding van eenen nieuwen eekhoren en eenen nieuwen pastor. Verhandelingen der tweede klasse van het Koninklijk-Nederlandse instituut van wetenschappen, letterkunde en schoone kunsten 2: 241–244. [2 pls]

[B431] van der Hoeven J (1830) Handboek der dierkunde of grondbeginsels der natuurlijke geschiedenis van het dierenrijk. Vol. 2. Wed. J. Allart, Rotterdam, 1068 pp. [24 pls]

[B432] van der Hoeven J (1831) Over de Chinchilla, *Mus Laniger* van Molina. Bijdragen tot de natuurkundige wetenschappen 6: 105–118.

[B433] van der Hoeven J (1844) Bijdragen tot de kennis van de Lemuridae of Prosimii. Tijdschrift voor Natuurlijke Geschiedenis en Physiologie 11: 1–48. [pls I–III]

[B434] van der Hoeven J de Vriese WH (1836) Boekbeschouwing en Letterkundige berigten. Tijdschrift voor natuurlijke geschiedenis en physiologie 3: 107–114.

[B435] van Musschenbroek SCJW (1883) Dagboek van Dr. H.A. Bernstein’s laatste reis van Ternate naar Nieuw-Guinea, Salawati en Batanta 17 October 1864–19 April 1865. Bewerkt door Mr. S.C.J.W. van Musschenbroek. Met aanteekeningen, bijlagen en eene kaart. Martinus Nijhoff, ‘s Gravenhage, 258 pp. 10.1163/22134379-90000433

[B436] van Nouhuys JW (1910) Toelichting bij de overzichtskaart 1: 200.000 der expeditie naar centraal Nieuw-Guinea 1907 en 1909/1910 onder leiding van Mr. H.A. Lorentz. Bulletin Maatschappij ter bevordering van het Natuurkundig Onderzoek der Nederlandsche Koloniën 64: 1–7.

[B437] van Steenis-Kruseman MJ (1950) Malaysian plant collectors and collections being a cyclopaedia of botanical exploration in Malaysia and a guide to the concerned literature up to the year 1950. In: Van Steenis CGGJ (Ed.) Flora Malesiana being an illustrated systematic account of the Malaysian flora, including keys for determination, diagnostic descriptions, references to the literature, synonymy, and distribution, and notes on the ecology of its wild and commonly cultivated plants.Series I. Spermatophyta. Noordhoff-Kolff, Djakarta, 5–639.

[B438] van Wingerden P (2023) Layered loyalties: The Natuurkundige Commissie in the Netherlands Indies (1820–1850). PhD Thesis, University of Leiden, Leiden, The Netherlands, 389 pp.

[B439] Vanzolini PE (1993) As viagens de Johann Natterer no Brasil, 1817–1835. Papéis Avulsos de Zoologia 38: 17–60. 10.11606/0031-1049.1992.38.p17-60

[B440] Vanzolini PE Myers CW (2015) The herpetological collection of Maximilian, Prince of Wied (1782–1867), with special reference to Brazilian materials. Bulletin of the American Museum of Natural History 395: 1–155. 10.1206/910.1

[B441] Vénec-Peyré M-T (2002) Alcide d’Orbigny (1802–1857): sa vie et son œuvre. C.R. Palevol 1: 313–323. 10.1016/S1631-0683(02)00043-X

[B442] Veth HJ (1879) Overzicht van hetgeen in het bijzonder door Nederland, gedaan is voor de kennis der fauna van Nederlandsch Indië. S.C. van Doesburgh, Leiden, viii, 204 pp.

[B443] Voisin C Voisin J-F Tranier M (1999) Note on Grandidier’s lemur collections from Madagascar. Mammalia 63: 535–536.

[B444] von Heuglin T (1863) Beiträge zur Zoologie Afrika’s. Über einige Säugethiere des Bäschlo-Gebietes. Verhandlungen der Kaiserlichen Leopoldinisch-Carolinischen deutschen Akademie der Naturforscher 30(2) Nachtrag: 1–14.

[B445] von Heuglin T (1864) Beiträge zur Zoologie Central-Afrikas, mit Tafeln. Nova Acta Academiae Caesarea Leopoldino Carolinae Naturae Curiosorum 31(7): 3–15.

[B446] von Heuglin T (1877) Reise in Nordost-Africa. Schilderungen aus dem Gebied der Beni Amer und Habab nebst zoologischen Skizzen und einem Führer für Jagdreisende. Vol. 2. Georg Westermann, Braunschweig, 304 pp. 10.5962/bhl.title.104692

[B447] von Heuglin T (1862) Reise der Herren Th. v. Heuglin, Dr. Steudner und H. Schubert im östlichen Theile des Hochlandes von Abessinien, Februar bis Mai 1862. Nach Briefen des Herrn *v. Heuglin*. Mittheilungen aus Justus Perthes’ Geographischer Anstalt über wichtige neue Erforschungen auf dem Gesammtgebiete der Geographie von Dr. A. Petermann 1862: 424–428.

[B448] von Humboldt A (1805) Mémoire sur l’os hyoïde et le larynx des oiseaux, des singes et du crocodile. In: Humboldt A, Bonpland A (1805–1809) (cited from reprint [1811] 1812). Recueil d’observations de zoologie et d’anatomie compareé, faites dans l’Océan Atlantique, dans l’intérieur du nouveau continent et dans la Mer du Sud pendant les années 1799, 1800, 1801, 1802 et 1803. Premier volume. F. Schoell & G. Dufour et Comp., Paris, 1–13. [pls I–IV] 10.5962/bhl.title.43770

[B449] von Humboldt A (1812) Sur les singes du royaume de la Nouvelle-Grenade et des rives de l’Amazone. In: De Humboldt A, Bonpland A., 1805–1812. Recueil d’observations de zoologie et d’anatomie comparée, faites dans l’Océan Atlantique, dans l’intérieur du nouveau continent et dans la Mer du Sud pendant les années 1799, 1800, 1801, 1802 et 1803. Premier volume. F. Schoell & G. Dufour et Comp., Paris, 336–344. 10.5962/bhl.title.43770

[B450] von Pelzeln A (1871) Zur Ornithologie Brasiliens. Resultate von Johann Natterers Reisen in den Jahren 1817 bis 1835. A. Pilcher’s Witwe & Sohn, Wien, lix, 462. [18 pls, 2 maps] 10.5962/bhl.title.3654

[B451] von Pelzeln A (1883) Brasilische Säugethiere. Resultate von Johann Natterer’s Reisen in den Jahren 1817 bis 1835. I, II. Verhandlungen der kaiserlich-königlichen zoologisch-botanischen Gesellschaft in Wien, Beiheft zu Band 33: 1–58. 10.5962/bhl.title.8930

[B452] von Rosenberg CBH (1865) Reistogten in de afdeling Gorontalo, gedaan op last der Nederlandsch Indische regering. Frederik Muller, Amsterdam, 162 pp. [6 pls, 4 maps]

[B453] von Siebold PF (1824) De historiae naturalis in Japonia statu, nec non de augmento emolumentisque in decursu perscrutationum exspectandis dissertatio, cui accedunt specilegia faunae Japonicae. Bataviae, 16 pp.

[B454] von Siebold PF (1897) Nippon. Archiv zur Beschreibung von Japan und dessen Neben-und Schutzländern Jezo mit den südlichen Kurilen, Sachalin, Korea und den Liukiu-Inseln. Verlag der K.u.K. Hofbuchhandlung v. Leo Woerl, Würzburg und Leipzig, 257 pp.

[B455] von Spix JB (1823) Simiarum et vespertilionum Brasiliensium species novae, ou histoire naturelle des espèces nouvelles de singes et de chauve-souris observées et recueillis pendant le voyage dans l’intérieur du Brésil exécuté par ordre de S. M. le Roi de Bavière dans les années 1817, 1818, 1819, 1820. Typis Francisci Seraphici Hübschmanni, München, viii + 72 pp, 38 pls von Spix JB, Von Martius CFP (1823–1831) Reise in Brasilien in den Jahren 1817–1820. Unveränderter Neudruck des 1823–1831 in München in 3 Textbänden und 1 Tafelband erschienenen Werkes. Herausgegeben und mit einem Lebensbild des Botanikers C.F.P. von Martius sowie mit einem Register versehen von Karl Mägdefrau. 3 volumes. M. Lindauer, München, xvii + 1388 pp. [40 pls, map.]

[B456] Voss RS (2022) An annotated checklist of recent opossums (Mammalia: Didelphidae). Bulletin of the American Museum of Natural History 455: 1–76. 10.1206/0003-0090.455.1.1

[B457] Voss RS Lunde PL Simmons NB (2001) The mammals of Paracou, French Guiana: a neotropical lowland rainforest fauna part 2. Nonvolant species. Bulletin of the American Museum of Natural History 263: 3–236. 10.1206/0003-0090(2001)263<0003:TMOPFG>2.0.CO;2

[B458] Voss RS Díaz-Nieto JF Jansa SA (2018) A revision of *Philander* (Marsupialia: Didelphidae), part 1: *P.quica*, *P.canus*, and a new species from Amazonia. American Museum Novitates 3891: 1–70. 10.1206/3891.1

[B459] Wagner A (1842) Diagnosen neuer Arten brasilischer Säugthiere. Archiv für Naturgeschichte 8(1): 356–362.

[B460] Waterhouse GR (1838) Catalogue of the Mammalia preserved in the Museum of the Zoological Society of London. 2nd ed. Richard and John E. Taylor, London, 68 pp.

[B461] Waterhouse GR (1839) Part II. Mammalia. In: Darwin C (Ed.) The zoology of the voyage of H.M.S. Beagle, under the command of Captain Fitzroy, R.N., during the years 1832 to 1836. Smith, Elder and Co. London, ix + 97 pp. [11 pls]

[B462] Waterhouse Gr (1848) A natural history of the Mammalia. Vol. II. Containing the order Rodentia, or gnawing Mammalia. Hippolyte Baillière, London, 500 pp. [21 pls]

[B463] Weber MWC (1890) Einleitung. In: Zoologische Ergebnisse einer Reise in Niederländisch Ost-Indien. Erster Band. E.J. Brill, Leiden xi + 3 maps. 10.5962/bhl.title.52289

[B464] Wied M (1820a) Reise nach Brasilien in den jahren 1815 bis 1817. 2 Vols. Brönner, Frankfurt a.M, 721 pp. 10.5962/bhl.title.85967

[B465] Wied M (1820b) Ueber eine noch unbeschriebenes Säugthier aus der familie der Naher. Isis von Oken 1: 43.

[B466] Wied M (1826) Beiträge zur Naturgeschichte von Brasilien. II. Band. Verzeichniss der Amphibien, Säugethiere und Vögel, welche auf einer Reise zwischen dem 13ten und dem 23sten Grade südlicher Breite im östlichen Brasilien beobachtet wurden. II. Abtheilung. *Mammalia*. Säugthiere. Verlag des Gr. H. S. priv. Landes-Industrie-Comptoirs, Weimar, 620 pp. [5 pls]

[B467] Wied M (1839) Über einige Nager mit äusseren Backentaschen aus dem westlichen Nord-America. Nova acta physico-medica Academiae Caesareae Leopoldino-Carolinae Naturae Curiosum 19: 366–384.

[B468] Williams DF Genoways HH (1979) A systematic review of the Olive-Backed Pocket Mouse, *Perognathusfasciatus* (Rodentia, Heteromyidae). Annals of Carnegie Museum 48(5): 73–102. 10.5962/p.330823

[B469] Wilson DE, Reeder DM (Eds) 2005. Mammal species of the world. A taxonomic and geographic reference. Third edition. 2 Vols. The Johns Hopkins University Press, Baltimore, xxxiv + 2142 pp.

[B470] Woods CA Kilpatrick CW (2005) Infraorder Hystricognathi Brandt, 1855. In: Wilson DE Reeder DM (Eds) Mammal species of the world.A taxonomic and geographic reference. Third edition. Vol. 2. The Johns Hopkins University Press, Baltimore, 1538–1600.

[B471] Woolley PA (2005) Revision of the three-striped dasyures, genus *Myoictis* (Marsupialia: Dasyuridae), of New Guinea, with description of a new species. Records of the Australian Museum 57: 321–340. 10.3853/j.0067-1975.57.2005.1450

[B472] Zinner D Fickenscher GH Roos C Anandam MV Bennett EL Davenport TRB Davies NJ Detwiler KM Engelhardt A Eudey AA Gadsby EL Groves CP Healy A Karanth KP Molur S Nadler T Richardson MC Riley EP Rylands AB Sheeran LK Ting N Wallis J Waters SS Whittaker DJ (2020) Family Cercopithecidae. In: Burgin CJ Wilson DE Mittermeier RA Rylands AB Lacher TE Sechrest W (Eds) Illustrated Checklist of the Mammals of the World.Lynx Editions, Barcelona, 29–105.

